# 2023 International Conference on Life, Health and Modern Medicine

**DOI:** 10.1097/MD.0000000000034187

**Published:** 2023-07-28

## LHMM23001 ALPHA-PINENE INHIBITS BIOFILM FORMATION AND VIRULENCE-FACTOR EXPRESSION OF METHICILLIN-RESISTANT STAPHYLOCOCCUS AUREUS

Yang Yang^a^, Young-Hoi Kim^b^, Kang-Ju Kim^c^, Yong-Ouk You^d^

*^a^ School of Agronomy and Life Sciences, Datong University, Datong, Shanxi, 037009, China, ^b^ Department of Food Science, Chonbuk National University, Jeonju, Jeonbuk, 54896, Republic of Korea, ^c^ Department of Oral Microbiology, Wonkwang University, Iksan, Jeonbuk, 54538, Republic of Korea, ^d^ Department of Oral Biochemistry, Wonkwang University, Iksan, Jeonbuk, 54538, Republic of Korea.*

**Background:** Methicillin-resistant *Staphylococcus aureus* (MRSA) is a typical antibiotic-resistant bacteria and it is a leading cause of nosocomial infections worldwide. *Staphylococcus aureus* is an extracellular bacterium that causes surface to severe hematogenous infections with high infection rate and mortality. Thus, novel antibiotics are urgently required to treat these bacteria. Essential oils (EOs) showed the antibacterial activity and has become the basis of many applications, including alternative medicine, natural therapies, food preservation, raw and processed, and pharmaceuticals. Natural products with EOs can be make use of as raw materials in antibacterial substances.

**Subjects and Methods:** In this study, the ɑ-pinene is a major compound of *Pinus densiflora* Sieb. et Zucc. (PSZ) EOs. The antibacterial activity of the essential oil extracted from PSZ against MRSA was investigated. The inhibitory effect of ɑ-pinene on MRSA growth, acid production, biofilm formation and expression of virulence factors were examined.

**Results:** PSZ EO was analyzed by gas chromatography (GC) and GC coupled for mass spectrometry, which identified 58 constituents, accounting to 90.98% of the total EO. The most components in all the EOs is α-pinene (20.56%). At concentrations of higher than 0.25 mg/mL (p<0.05), the ɑ-pinene was examined to inhibit the growth of MRSA. After using the safranin staining method, ɑ-pinene showed an inhibitory effect against biofilm formation. As well as the experimental results were similar to the scanning electron microscopy. At high concentrations with ɑ-pinene in a concentration dependent manner (of 1–8 mg/mL) also showed a bactericidal effect by the confocal microscopy. According to the real-time polymerase chain reaction, mRNA expression of virulence factor genes, sea, agrA, mecA, and sarA, was observed. All the expression was observed at a concentration of 0.25 mg/mL by ɑ-pinene.

**Conclusions:** These results suggest that ɑ-pinene, a major compound of PSZ EOs, has the antibacterial activity against MRSA, antibiotic resistant bacteria.

### Acknowledgments

This paper was supported by Wonkwang University in 2019.

## LHMM23002 ANALYSIS OF 12 CASES OF FEVER AFTER MODIFIED ELECTROCONVULSIVE THERAPY

Cunbo Wu^a^

*^a^ Chongqing Mental Health Center, Gele Mountain, Shapingba district, Chongqing, China.*

**Objective:** To investigate whether modified electronconvulsive therapy (MECT) causes fever and its causes and feature.

**Methods:** In accordance with the inclusion and exclusion criteria, Data analysis was conducted on 12 patients with mental disorders who were treated with modified electronconvulsive fever after treatment from February to May 2022 at chongqing Mental Health Center.

**Results:** Fever is more common in adolescents after MECT treatment, 4 persons under the age of 20, accounting for 33.3%; 5 persons aged 20-29, accounting for 41.7%; mainly with moderate fever, 7 cases (58.3%), usually occurs in the second time of MECT (50%) and in that afternoon of the day after operation, 11 cases (91.6%). One case was fever after the third time, accounting for 10%; Two cases (20%) developed fever after the eighth MECT. 4 cases (33, 3%) had normal blood routine after MECT. 8 cases (66.7%) had elevated WBC. 4 cases (33.3%) showed normal CRP during fever after MECT. CRP was elevated in 8 cases (66.7%). Among the patients with fever, there were 5 females (41.7%) and 7 males (58.3%). 9 cases (75%) of febrile patients were schizophrenic. 2 cases of affective disorder (16.7%); 1 case (8.3%).

**Conclusions:** The fever caused by MECT should not be ignored and should be treated promptly. Relevant auxiliary examination should be improved to clarify the cause and further treatment. MECT treatment should be suspended if necessary.

## LHMM23003 CLINICAL EVALUATION AND ANTIBIOTIC SELECTION TO TREAT STAPHYLOCOCCUS AUREUS INDUCED INFECTIVE ENDOCARDITIS IN CHILDREN

Xin Guo^a^, Bing Hu^a^, Qinjing Li^a^, Linlin Liu^a^, Zhenzhen Dou^a^, Zhihui Zhao^b^, Liang Zhu^a^, Tianming Chen^a^, Lingyun Guo^a^, Gang Liu^a^

*^a^ Key Laboratory of Major Diseases in Children, Ministry of Education, Department of Infectious Diseases, Beijing Children’s Hospital, Capital Medical University, National Center for Children’s Health, Beijing, 100045, China, ^b^ Department of Heart Diseases, Beijing Children’s Hospital, Capital Medical University, National Center for Children’s Health, Beijing, 100045, China.*

**Background:** Infective endocarditis (IE) is a rare and fatal disease in children, with Staphylococcus aureus (SA) as the leading cause in many regions of the world. Staphylococcus aureus infective endocarditis (SAIE) often presents sudden onset of sepsis and embolism, with multiple organs damage. SAIE with persistent bacteremia is a challenge in clinical practice. Some specific antibiotics such as daptomycin are available as an alternative when conventional treatment fail.

**Subjects and Methods:** The research team retrospectively analyzed the medical records of 7 pediatric patients with infective endocarditis caused by Staphylococcus aureus admitted to Beijing Children’s Hospital from February 1, 2018 to February 28, 2021. The etiology, symptoms and signs, laboratory results, treatment and prognosis were collected, and compared with 13 other cases. Blood culture and echocardiography were used to diagnose the disease, and the efficacy of different antibiotics was analyzed.

**Results:** SAIE accounted for 35% (7/20) of all IE patients, with 7 cases ranging in age from 1 year 5 months to 13 years 8 months. 3 cases (42.9%) had methicillin- resistant Staphylococcus aureus (MRSA) and 4 cases (57.1%) were methicillin-sensitive Staphylococcus aureus (MSSA). A total of 4 cases (57.1%) had congenital heart disease. All patients (100%) had fever and 3 cases (42.9%) had major vessel embolic events. 4 cases (57.1%) had central nervous system complications and 4 cases left heart endocarditis. All patients initially received vancomycin-based treatment regimens. 2 cases (28.6%) with persistent bacteremia were changed to a 9 mg/kg/dose of daptomycin when the conventional treatment failed to vancomycin therapy. The outcomes were good and no significant side effects occurred. Daptomycin in our study was first applied successfully for SAIE with persistent bacteremia in Chinese children.

**Conclusions:** SA was the common major pathogen detected in IE. Appropriate antibiotic therapy needs to be adopted, and evaluation of persistent bacteremia and vascular embolic events is needed. The study also showed that vancomycin and linezolid are still the first-line choices for treatment of the disease, but daptomycin can also be an effective treatment option.

### Acknowledgments

The authors would like to thank Huiyu Liu’s writing support from MSD, Qinjing Li and Tianming Chen’s support during the conduction of this study.

## LHMM23004 THERAPIUTIC EFFECT ANALYSIS OF JIAO’S THE SCALP ACUPUNCTURE COMBINED WITH TASK-ORIENTED TRAINING ON MOTOR FUNCTION OF CHILDREN WITH SPASTIC CEREBRAL PALSY

Xiaosu Jie^a^, Lin Niu^b^, Haijun Shan^a^, Caihong Cao^a^, Yingying Zhang^a^, Yujin Hou^a^

*^a^ Child Rehabilitation Department, Henan Province Hospital of Traditional Chinese Medicine, Zhengzhou, China, ^b^ Department of medical technology, Zhengzhou Railway Vocational and Technical College, Zhengzhou, China.*

**Background:** This study analyzed the effects of Jiao’s scalp acupuncture combined with task-oriented training on gross motor function classification, gross motor function assessment and motor development of children with spastic cerebral palsy. Spastic cerebral palsy is the most common type of cerebral palsy with motor dysfunction among the main clinical manifestations. Even the severe patients can not walk for life. So exercise intervention is the focus of cerebral palsy treatment. This study aims to provide more effective treatment methods for pediatric spastic cerebral palsy.

**Subjects and Methods:** Fifty children admitted to our hospital for spastic cerebral palsy from January 2020 to June 2021 were enrolled, and assigned to the control group (n=25) receiving task-oriented training, and the observation group (n=25) receiving Jiao’s scalp acupuncture plus task-oriented training. Before and at 6 months after treatment, the gross motor function grade, gross motor function assessment score changes and the satisfaction of children’s guardians with rehabilitation treatment were compared.

**Results:** After the treatment, the total gross motor function score was higher in the observation group (P<0.05). The number of gross motor function in patients with decreased grade, the Peabody motor development score, the satisfaction and overall clinical efficacy of the children’s guardians were higher in the observation group (all P<0.05).

**Conclusions:** Jiao’s scalp needle was founded by Professor Jiao Shunfa in 1971. Based on the theory of cerebral scalp needle has been widely used in the clinical treatment of various encephalopathy in China due to its convenient acupoint selection and simple operation, and has achieved positive clinical effect in the treatment of spastic cerebral palsy in children. Jiao’s scalp acupuncture combined with task-oriented training applied in spastic cerebral palsy children has obvious practical effect. They can reduce the disease of children and improve the level of gross motor function of children, improve the quality of life and self-reliance, self-care ability of children at the same time. It also can enhance parents’ confidence in rehabilitation and improve parents’ satisfaction with rehabilitation treatment. As a result, the effect is better than that of task-oriented training routine treatment.

### Acknowledgments

This work was supported by the Scientific research project of traditional Chinese medicine in Henan Province, Project: 2019ZY2056

## LHMM23005 THE DESIGN STRATEGY OF COGNITIVE-BEHAVIORAL AND MENTAL HEALTH TRAINING TOYS FOR CHILDREN WITH AUTISM BASED ON INPD THEORY

Chenlin Wang^a^, Yi Feng^a^

*^a^ School of Art and Design, Beijing Forestry University, Beijing, China.*

**Background:** The incidence of Autism Spectrum Disorder (ASD) in children around the world is on the rise, and the development of mental health in autistic children due to physical defects can lead to cognitive, emotional, behavioral and other disorders. Since the etiology and biochemical abnormalities of ASD have not been fully elucidated, and there is no specific drug to treat ASD pathologically, ASD has increasingly become a worldwide problem facing human society. The importance of toys for the healthy development of children makes it possible to improve the above problems through toy training. The design of cognitive behavioral training toys for children with ASD has received a lot of attention. The INPD method is widely used in many fields, providing the possibility to solve the problem of designing cognitive behavioral training toys for children with ASD.

**Subjects and Methods:** Based on the design method of INPD theory, the design strategy of ASD Children’s cognitive behavioral health training toys is explored. This paper uses INPD, combined with AHP, to guide the toy design ideas for ASD children. Firstly, the design gaps is found from SET analysis method; then, a questionnaire survey is conducted on ASD children’s families to clarify the needs of ASD children’s families for cognitive behavioral health training toys and classify the needs to form a hierarchical diagram of user needs; finally, the nine-scale method of AHP hierarchical analysis is introduced to calculate the important priorities of different levels of needs. Finally, different requirements are sorted into design strategies in priority order.

**Results:** The product concept is then translated into a design strategy. According to the priority order of needs, the design strategy of ASD children’s cognitive behavior training toys should focus on logic and cooperation in content, establish toy IP and develop intelligent training toys. In addition to following the safety principle, the design principle should also pay attention to the simplicity and scalability of toys, so as to fully realize the purpose of training the physical functions of ASD children and letting them grow up healthily.

**Conclusions:** The design method of combining INPD and AHP effectively solves the problem of prioritization of user needs in toy design, provides a new design idea for the design of cognitive behavioral health training toys for autistic children, and also provides a new method for other research on user needs.

## LHMM23006 ON INTEGRATION TRAINING OF CHILDREN’S SPORTS SENSORY: INTERPRETATION, APPLICATION, AND DESIGN

Junwei Wang^a^

*^a^ Wuhan Sports University, Wuhan 430079, China.*

**Background:** With the rapid development of high quality in the education, the aim of this study was to Children sensory integration has become the focus of global education. It has important theoretical and practical significance to improve children’s sensory integration through sports activities or training.

**Subjects and Methods:** Take the development frontier and practice cases of children sports and sensory integration abroad as an opportunity, this paper uses comprehensive analysis methods, such as literature review and data statistics, to comb the meaning, application and design of children’s sport and sensory integration. Meanwhile, this research will focus on the development history, current situation, function and role of sensory integration. Combining the special functions of sports and empirical cases, explain children’s bad behaviors in the environment, life, and learning, and provide a scientific de mainly base sign path.

**Results:** Sensory integration training is based on games to cultivate children’s learning interest, self-consciousness, and teamwork capabilities. They Correct children’s incorrect standing, sitting, walking, running, etc. through specific language environment, body movements and teaching aid guidance and behavior habits. Therefore, children sports is a brand-new training model tailored to the individual needs of children. Studies have found that the early childhood stage is a key stage for the development of the sensory nervous system. It is believed that the sensory system is a sub-component of the brain motor control system, which needs sports activities or training to stimulate the neural linkage, and the linkage is realized through the complementary relationship between static and dynamic components, On the child’s brain growth and limbs and trunk have a healthy role in promoting.

**Conclusions:** Sports activity or training can promote children’s body, vestibular, tactile and other sensory systems to process, improve the flexibility and adaptability of children’s body posture, joints, muscles, eyesight, hearing and other body parts during exercise, and have a positive impact on children’s cognitive and mental health. The purpose is to solve children’s behavioral cognition and mental strengthening training, so that the child’s brain can smoothly integrate and process various sensory information and make correct decisions. Therefore, learning sports activities in childhood is an important functional attribute of promoting the healthy growth of children.

### Acknowledgments

**Foundation Project:** 2019 Scientific Research Program of Hubei Provincial Department of Education (Project number: B2019196); Wuhan Sports University Scientific Innovation team of young and middle-aged funding project.

## LHMM23007 RESEARCH ON THE RELATIONSHIP BETWEEN CHINA’S HEALTH EXPENDITURE STRUCTURE AND PUBLIC HEALTH OUTPUT: AN ANALYSIS BASED ON THE SYSTEM GMM METHOD

Penghui Xu^a,b^, Haili Li^b^, Shi Guo^b^, Guoping Wang^b^ and Hairong Liu^b^

*^a^ Department of Management, Jiangsu University, Zhenjiang, Jiangsu 212013,China, ^b^ Anhui Key Laboratory of Philosophy and Social Sciences for Public Health Crisis Management in Colleges and Universities, Wannan Medical College, Wuhu 241002, China.*

**Background:** To investigate the impact mechanism of health input structure attributes on health development and its regional differences based on the perspective of human capital.

**Subjects and Methods:** Using panel data from 31 Chinese provinces from 2011 to 2018 and using the systematic GMM estimation method, the public health output effects of the health expenditure structure and its regional differences were investigated.

**Results:** Firstly, the empirical results showed that social health expenditure significantly increased public health output. There were regional differences: the eastern region showed the strongest effect in this regard, the central region had the second strongest, and the western region involved the weakest effect. However, government health and personal hygiene investment did not significantly increase public health output. Second, the number of doctors per thousand people had a significant impact on public health output, but there were significant regional differences: the eastern region had the strongest promotional effect, while the central and western regions were not significant. Third, there were significant regional differences in the impact of the number of beds per thousand people on health output, of which the western region was the most significant, followed by the central region, and the promotional effect in the eastern region is not significant. Fourth, the per capita GDP and environmental factors in the three major regions of eastern, middle, and western China had no significant impact on the health level, while there was a significant regional difference in the impact of per capita education level on the health level. The regional promotional effect was relatively high, while the central and western regions were not significant.

**Conclusions:** The conclusions of this study have important policy implications. In order to further improve the health of residents and build a healthy China, we put forward the following policy recommendations: Strengthen the input of health resources in key areas is to ensure basic medical services, effectively ensure the equalization of basic medical services, and improve the accessibility and affordability of medical services; The government should increase support for social health in the central and western regions, implement a variety of tax incentives to promote the continuous growth of social health expenditure, and build a mechanism to maximize the efficiency of health resources.

### Acknowledgments

The authors thank all participants who made an effort in this study. This study was funded by the grants from the Ministry of Education of China (grant 20YJCZH193) and Anhui Provincial Department of Education (grant SK2017A0212).

## LHMM23008 A REGULARITY COMPARATIVE STUDY OF SELENIUM DEPOSITION, ANTIOXIDANT ACTIVITY, GROWTH PERFORMANCE AND HEALTH BETWEEN DIFFERENT PHENOTYPES OF DONGXIANG GREEN-SHELL LAYING HENS (RED AND PURPLE COMB) AFFECTED BY VARIOUS SELENIUM SOURCES

Han Yan^a^, Xiaoyu Chang^a^, Qinglan Liu^a^, Kaiwen Zhao^b^, Songqiang Yi^c^, Yingmei Yu^a^, Sansan Shao^a^, Zhenghua Huang^a^, Ruili Li^d^, Siming Li^a^

*^a^ Key Laboratory for quality and safety control of poultry products, Ministry of Agriculture and Rural Affairs of the People’s Republic of China, Institute for Quality & Safety and Standards of Agricultural Products Research, Jiangxi Academy of Agricultural Sciences, Nanlian Road 602, Nanchang 330200, China, ^b^ Nanchang Institute of Technology, Nanchang 330044, China, ^c^ Agricultural Technology Extension Center of Jiangxi Province, Nanchang 330046, China, ^d^ Yantai Academy of Agricultural Sciences, Yantai 265500, China.*

**Background:** Selenium is a necessary nutrient element in the human body. Because the human body cannot synthesize selenium, it needs to be supplemented from the outside. Moderate supplement of selenium can improve human immunity and resistance, and promote human health. At the same time, with the improvement of people’s living standards, the development of healthy and safe functional selenium enriched eggs has attracted more and more attention. In this study, Dongxiang green-shell laying hens was taken as the experimental object, to investigate the effect of nine different kinds of selenium (Se) feeding methods to Dongxiang green-shell laying hens with different morphological characteristics (red and purple comb) on egg concentration, viscera antioxidant properties, and slaughter index. In order to provide a basis for the production of safe and healthy selenium enriched green shell eggs.

**Subjects and Methods:** The experiment was carried out on two hundred and fifty two 30-week-old Dongxiang black feather laying hens and the red comb ones and purple crests ones equal share of the total number of the test. These hens were allocated to nine dietary treatments and each group was divided into four parallels with 7 hens per replicate and two of the replicates have red crests and the other have purple crests. respectively. The hens were free to eat and drink during the experiment. We controlled the winter feeding temperature between 16 and 18 °C and the lighting in hen house was 14h daily.

**Results:** The result shows that the organic selenium group has higher portion of selenium in all indicators measured than inorganic selenium group significantly (P<0.1). Also the total selenium content is higher in high concentration group than in low ones under the stress of the same selenium source. Moreover, enrichment of selenium content in egg and breast was the most effective (0.477 μg/kg, 0.198 μg/kg) via application of dietary selenomethionine (0.6 mg/kg) in purple crown group. Statistically, there is a significant difference (P＜0.1) in the concentration of selenium deposition in eggs between purple and red cockles in the same selenium source group (selenomethionine, selenium yeast, sodium selenite). And the difference in the color of chicken’s crown had a significant effect on the selenium content deposited in the plasma and visceral of the selenomethionine treatment group (P<0.1).

**Conclusions:** the selenium supplemental concentration was positively correlated with the anti-oxidant capacity in viscera and the effect was significant. In conclusion, adding 0.6 mg/kg selenomethionine in the diet of purple crown laying hens has the best effect on selenium enrichment in eggs and tissues, and the detection indexes are better than those of red crown ones. This study provides a theoretical basis for the development of selenium-rich products.

### Acknowledgments

Study on selenium deposition mechanism and key technologies of Product development and utilization in green shell eggs (Item number: 20202BBFL63029); Study on technology system of nutritional evaluation of agricultural products (JXXTCXNLTS202103); Study and application of key technologies for high value fermentation of Selenium-rich rice (Item number: 20202BBFL63011).

## LHMM23009 IMAGING STUDY ON PRIMARY INSOMNIA PATIENTS WITH ELECTROACUPUNCTURE AT SHENMEN COMBINED WITH NEIGUAN POINT

Ganbin Qiu^a,b^, Weixiong Liao^a^, Zhongyan Wen^b^, Yue Zhao^c^, Yonghui Liu^b^, Jincan Chen^b^

*^a^ Imaging department of Zhaoqing Medical College, Zhaoqing 526020, China, ^b^ Department of Radiology, The First People’s Hospital of Zhaoqing, Zhaoqing 506040, China, ^c^ Department of Radiology, The First Affiliated Hospital of Jinan University, Guangzhou 510000, China.*

**Background:** To compare the changes of brain low-frequency amplitude (ALFF) in patients with primary insomnia (PI) and healthy control group (HC) before and after the treatment of Shenmen combined with Neiguan point, and to explore the neural mechanism of brain function changes in patients with PI by electroacupuncture Shenmen combined with Neiguan point.

**Subjects and Methods:** From November 2019 to May 2022, 23 PI patients (9 males and 14 females) were recruited in Zhaoqing First People’s Hospital as the study group, and 26 healthy volunteers (12 males and 14 females) with age, sex and education level were recruited in society as the control group; All the subjects were scored with the clinical Pittsburgh Sleep Quality Index (PSQI), Hamilton Depression Scale (HAMD) and Hamilton Anxiety Scale (HAMA) before acupuncture, and all the subjects were scanned with resting MRI before acupuncture treatment; Then select Shenmen and Neiguan points of the two groups of subjects for electroacupuncture therapy. After “Deqi”, the electroacupuncture will continue to stimulate for 30 minutes. After another electroacupuncture treatment the next day, the resting MRI scan will be performed again. The low frequency amplitude (ALFF) analysis method was used to calculate the ALFF of brain regions between PI group and HC group before and after acupuncture, and the rest state in PI group before and after acupuncture.

**Results:** There was no significant difference in gender and age between the two groups (p>0.05), but the PSQI score, HAMA score and HAMD score of patients in the study group were significantly higher than those in the control group (p<0.05). Before acupuncture treatment, the brain areas with increased ALFF values in PI group included the right cuneiform lobe, left inferior temporal gyrus, left amygdala, etc., while the brain areas with decreased ALFF values included the right parahippocampal gyrus, right anterior cuneiform lobe, left inferior parietal lobule, and left thalamus; After acupuncture treatment, the brain areas with increased ALFF values in PI group included the left posterior cingulate gyrus, bilateral anterior cuneiform lobe and right cuneiform lobe, and the brain areas with decreased ALFF values included the left amygdala, the right transverse temporal gyrus, and the right posterior central gyrus.

**Conclusions:** The brain regions of default network, significant network and part of emotional cognitive network in patients with primary insomnia (PI) are damaged, which may be the microcosmic manifestation of daily behavior and cognitive abnormalities in PI population. The combination of electroacupuncture at Shenmen and Neiguan points can activate part of the damaged brain areas of PI population, so as to improve the mental state and sleep, which may provide a new auxiliary scheme for clinical treatment of PI population.

### Acknowledgments

This work was supported by the Science and Technology innovation of Zhaoqing City, Guangdong Province, China under Grant (No. 2019N008). This work was supported by Medical Scientific Research Foundation of Guangdong Province, China under Grant (No.B2020225).

## LHMM23010 SHORT-TERM EFFICACY OF MIS-DYNESYS AND MIS-TLIF IN THE TREATMENT OF SINGLE LEVEL LUMBAR DEGENERATIVE DISEASE

Kai Cui^a^, Tian Tian^a^, Shengzhi Tan^a^, Tao Liu^a^, Dong Wang^a^, Yuan Li^a^, Di Wu^a^, Rong Tan^a^

*^a^ Department of Spine Surgery, Strategic Support Force of Chinese PLA, Beijing, 100101, China.*

**Background:** To evaluate the safety and reliability of minimally invasive Dynesys implantation and discectomy (MIS-Dynesys) and minimally invasive transforaminal lumbar interbody fusion (MIS-TLIF) under Quadrant tube in single level lumbar degenerative diseases.

**Subjects and Methods:** From January 2016 to December 2018, a retrospective analysis was carried out on 65 patients with single-segment lumbar degenerative diseases admitted to our hospital. Among them, 21 patients, 12 males and 9 females, with an average age of 64.57 ± 6.8 years, were treated in the MIS-Dynesys group, and 44 patients in the MIS-TLIF group, 19 cases in men and 25 cases in women, with an average age of 64.16 ± 5.71 years old. The general data, duration of surgery, bleeding volume, drainage, and oswestry disability index (ODI), JOA score (Japanese Orthopedic Association), and Visual Analogue Scale (VAS) for preoperative and postoperative and final follow-up were recorded to evaluate clinical efficacy. Before surgery, X-rays of lumbar orthovertebral lateral position and flexion and extension position, lumbar CT, lumbar spine MRI combined with clinical determination of the responsibility gap. 2 days after the operation, X-rays of the lumbar vertebrae in the lateral position and the flexile and extensive position were taken, and the LUM CT was re-determined to determine the internal fixation position and then moved to the ground. At the last follow-up, both patients were taken X-rays of the lumbar orthovertebral lateral position and flexion and extension motility position, the mobility of the surgical segment was measured, and the fusion group reviewed the CT to assess the fusion effect, and the data were collected for comparative study.

**Results:** The follow-up time of the two groups of patients was 6-39 months. The results of the MIS-Dynesys group and the MIS-TLIF group were not statistically significant in terms of general data, and operation time, and bleeding volume (P>0.05), and the former had significantly lower drainage than the latter, and the difference was statistically significant (P<0.05). Preoperative ODI JOA lumbago and leg pain VAS scores in 2 groups were not statistically significant (P>0.05); ODI JOA and lumbago and leg pain VAS scores in 2 groups at the end of 3 months after surgery were significantly improved compared with those before surgery, and the differences were statistically significant (P<0.05); ODI JOA and lumbago and leg pain VAS scores were not statistically significant between the two groups at the end of 3 months after surgery(P>0.05).There was no statistical difference in the activity results of the preoperative target segment (P>0.05) between the two groups, and the postoperative follow-up, the former of which still maintained a certain degree of activity, and the latter was well fused, the mobility was 0, and the difference was statistically significant (P<0.05).

**Conclusions:** For single level lumbar degenerative diseases, MIS-Dynesys can achieve similar result with the MIS-TLIF. The MIS-TLIF group had more drainage and fusion. The MIS-Dynesys group maintain a degree of motion.

## LHMM23011 PRACTICE OF COMPUTER BASIC CURRICULUM REFORM IN MEDICAL COLLEGES WITH THE CORE OF CULTIVATING COMPUTATIONAL THINKING AND HEALTH EDUCATION

Huanqing Xu^a^, Fangliang Huang^a^, Tongping Shen^a^

*^a^ School of Medicine and Information Engineering, Anhui University of Chinese Medicine, Hefei, Anhui 230012, China.*

**Background:** To cultivate new medical talents with high information literacy and healthy mental qualities, the teaching of basic computer courses in medical schools should be made more practical, specialized and useful. The goal of this paper is to study how the characteristics and advantages of the subject matter of basic computer courses in medical schools can be combined with mental health education and permeated in classroom teaching, in order to cultivate good motivation and a healthy personality for students and to establish a positive and active mental quality for later entry into the workplace.

**Subjects and Methods:** This paper is to highlight the professional characteristics of medicine in the teaching of basic computer courses at Anhui University of Chinese Medicine, and to create a computer course with a “medical flavor”. The course integrates computational thinking into the curriculum and further enhances students’ understanding and absorption of knowledge by “dissecting” complex problems at different levels. In addition, the online and offline integration of the SPOC teaching model can fully motivate students to learn on their own. In the teaching of basic computer courses, the overall objective of quality education should be taken as the basis for establishing corresponding mental quality objectives. Through training, counselling, implication and infection, students’ mental quality will be improved, their mental barriers will be solved, their mental health will be promoted and their healthy mental qualities will be cultivated.

**Results:** After one cycle of reform practice, the teaching results of four Applied Psychology classes of different grades taught by the author were remarkable. A comparison of the course formative assessment results shows that the SPOC combined with the computational thinking development teaching model and integrate into mental health education has greatly stimulated students’ interest in learning, with a significant increase in both pass and merit rates.

**Conclusions:** Classroom practice shows the importance of integrating computational thinking and health education into the SPOC teaching model as a ‘new treatment’ to facilitate the development of pedagogical reform in the course.

### Acknowledgements

This study was supported by the Key Project of Scientific Research in Anhui Higher Education Institutions of China under Grant NO. KJ2021A0587 and NO. SK2020A0244, the Provincial Quality Engineering Project in Anhui Higher Education Institutions of China under Grant No.2020jyxm1029 and 2022jyxm857.

## LHMM23012 CLINICAL ANALYSIS OF INTERLOCKED INTRAMEDULLARY NAIL IN TREATMENT OF FEMORAL SHAFT FRACTURES IN ADOLESCENTS

Jun Wen^a^, Feng Shan^a^, Yimeng Wang^b^, Ya Liu^a^, Yao Liu^a^, Yunfang Zhen^a^

*^a^ Department of Orthopaedics, Children’s Hospital of Soochow University, Suzhou, Jiangsu 215000, China, ^b^ Department of Orthopaedics, First Affiliated Hospital of Soochow University, Suzhou, Jiangsu 215000, China.*

WJ and SF contributed equally to this study.

**Background:** To explore the efficacy of interlocking intramedullary nail in the treatment of femoral shaft fractures in adolescents.

**Subjects and Methods:** The clinical data of 10 adolescent femoral shaft fractures treated with interlocking intramedullary nailing in our hospital from January 2020 to December 2021 were retrospectively analyzed. Among the patients, 8 were male and 2 were female. Their ages ranged from 11.5 to 15.3 years (mean, 12.8 years). Their weight ranged from 50.2kg to 70.2 kg (mean, 61.4 kg). The procedure was fixed with a static interlocking intramedullary nail. Periodic follow-up examinations were performed to monitor the occurrence of complications and to record the time of union of the fracture. At the last follow-up, neck shaft angle (NSA), medial proximal tibial angle (MPFA), femoral head diameter (FHD), femoral neck diameter (FND), and articulotrochanteric distance (ATD) were measured on the X-ray. Functional recovery of the affected limb was assessed using Thoresen’s criteria.

**Result:** The mean follow-up time was 14.4 months (10-17 months). All fractures healed, and all incisions healed at the first stage. No infection or incision dehiscence occurred. The average fracture union time was 5.1 weeks (4-8 weeks). The average radiological union time of the fracture was 9.6 weeks (6-12 weeks). There was no nonunion of the fracture,no delayed union, no malunion, no broken nails, no re-fracture. A leg length discrepancy of 1.4cm was observed in one case. One patient had ectopic ossification above the greater trochanter. Pressure ulcers occurred at the ankle in one patient. The NSA, MPFA, FHD, FND, and ATD at the final follow-up were, respectively, [(135.57 ± 4.20)°, (91.86 ± 6.39)°, (47.67 ± 2.70)mm, (32.35 ± 2.61)mm, (31.55 ± 3.30)mm] for the affected side,which were insignificantly different from those for the healthy side [(136.42 ± 6.92)°, (92.29 ± 8.01)°, (47.34 ± 2.79)mm, (32.29 ± 2.27)mm, (29.66 ± 2.42)mm] (*P*>0.05). According to the Thoresen criteria, there were 8 (80%) excellent, 1 (10%) good, and 1 (10%) satisfactory results with an excellent to good rate of 90%.

**Conclusion:** Interlocking intramedullary nailing is clinically effective in the treatment of femoral shaft fractures in adolescents, with excellent fracture union, few complications and no significant effects on proximal femur development in the short term.

## Acknowledgements

This work was supported by funds from National Natural Science Foundation of China (no. 82172520).

## LHMM23013 RESEARCH ON THE APPLICATION OF BLENDED PEDAGOGY IN TRACK AND FIELD CURRICULUM AND ITS TRANSFORMATION OF HEALTH EDUCATION METHODS

Fenglan Guo^a^, Guoshuai Ma^b^, Gandan Si^c^, Xiaoqiang Liu^d^, Lixin Chen^e^

*^a^ School of Physical Education, Xinjiang Normal University, China, ^b^ Xinjiang Institute of Engineering, China, ^c^ School of Physical Education, Changji University, China, ^d^ Urumqi NO.8 middle school, China, ^e^ Xingjiang Vocational Institute of Energy Technology, China.*

**Background:** In the era of “Internet +”, the application of blended teaching method has become an important starting point for the construction of first-class physical education courses in China, which is the direction of the curriculum reform of higher physical education and highlights its unique advantages in the application and development of physical education classroom teaching, namely, blended teaching can not only be applied to the theory and health knowledge classroom, but also can be combined with physical education technology teaching. And then provide services to promote students’ health, health education and its physical and mental health.

**Subjects and Methods:** This study takes blended teaching method of general track and field courses as the research theme, adopts the methods of literature review, questionnaire survey and on-the-spot investigation to carry out the application implementation of the online and offline blended teaching method for the students in Grade 2021 of physical education major, and explores the application effect of online and offline blended teaching practice of general track and field courses.

**Results:** (1) Blended teaching of track and field general courses should include independent learning, cooperative learning, question answering, practice consolidation, summary and evaluation. (2) The application of mixed teaching of track and field general courses has different degrees of influence on the innovation of teaching and learning, the working efficiency of teachers, the improvement of students’ technical skills and abilities, and the evaluation of teaching performance. (3) Combining mixed teaching of track and field general course with ideological and political practice of the course has a certain promoting effect and value on the mining, design and practical application of ideological and political elements of the course. (4) Through the combination of online and offline teaching and practice platform of track and field courses, the concept of physical and medical integration can be integrated into the sports training and rehabilitation education and learning of college students majoring in physical education, so that the sports health education of college students majoring in physical education can develop in a scientific way

**Conclusion:** (1) Use the Chaoxing Learning platform to carry out blended teaching design and practice, expand students’ multiple ways of learning in track and field, meet students’ professional learning needs, and improve students’ self-learning ability (2) By teaching theories and health knowledge about track and field events online, and learning skills online and offline, students can better master the health knowledge about track and field, fitness means and skills, cultivate students’ professional quality of teachers, and enhance students’ comprehensive ability of sustainable development. (3) The referee practice of track and field general training events and the ability training of demonstration and explanation are carried out in the way of cooperative group rotation, so as to stimulate students to give full play to their potential, integrate and expand the application of what they have learned, and cultivate the core quality of physical education teachers in primary and secondary schools. (4) Integrate ideological and political education into the online and offline teaching process, improve students’ comprehensive ability and the effect of ideological and political education in the course, so that the core quality of students’ future teacher career can be improved accordingly, which is in line with the talent training program. (5) The combination of online and offline teaching methods can organically combine skill practice with theoretical learning of sports health education, so as to promote the effectiveness and scientific development of track and field courses.

### Acknowledgements

This work was supported by the First-Class Undergraduate Course of Xinjiang Normal University in 2021 ----Hybrid Online and Offline First-Class Courses (Track and Field), the Social Science Fund of Autonomous Region, Project No.: 22CTY031.

## LHMM23014 THE POSITIVE EFFECTS OF REGIONAL COMPREHENSIVE ECONOMIC PARTNERSHIP (RCEP) ON THE PSYCHOLOGICAL INCENTIVES AND EMOTIONAL REGULATION OF TRADERS AND INVESTORS AND REFLECTION ON THE ROLE OF CHINA EXPORT CREDIT INSURANCE

Jian Xu^a^

*^a^ China Export & Credit Insurance Corporation, Beijing 100033, China.*

**Background:** To illustrate how RCEP offers positive psychological incentives and emotional regulation for the traders and investors, thus promoting trade and investment in East Asia, and to give some suggestions on how China export credit insurance could play an important role in serving China’s opening up and supporting enterprises to develop markets in the contracting parties.

**Methods:** This paper, from the psychological perspective, compares RCEP with the General Agreement on Tariffs and Trade (GATT), the General Agreement on Trade in Services (GATS), the Agreement on Trade-Related Investment Measures (TRIMs), the Trade Facilitation Agreement (TFA), Agreement on Safeguards(AS), the US-Canada-Mexico Agreement (USMCA), the EU-Canada Comprehensive Economic and Trade Agreement (CETA), and the Comprehensive and Progressive Agreement for Trans-Pacific Partnership (CPTPP) to illustrate how RCEP offers psychological incentives and emotional regulation for the traders and investors, thus promoting trade and investment in east Asia. Besides, the paper preliminarily judges the opportunities and challenges of China export credit insurance to support China’s enterprises in conducting trade and investment in the contracting parties, finally, it gives some suggestions on how China export credit insurance could play an important role in serving China’s opening up and supporting enterprises to develop markets in the contracting parties.

**Results:** The analysis of RCEP rules shows that RCEP will give positive psychological incentives and emotional regulation to traders and investors in East Asia, and then expand regional trade and investment in the region. At the same time, RCEP allows the parties to take relevant protective measures, which may reduce the psychological expectations of traders and investors, and thus may have negative effects on trade and investment in the region. Based on the above, from a practical point of view, China export credit insurance could strengthen the positive psychological incentive and emotional regulation of the agreement, stabilize the psychological expectation of China’s traders and investors to develop market in East Asia.

**Conclusions:** RCEP rules could give positive psychological incentives and emotional regulation to traders and investors in East Asia. At the same time, RCEP may have negative effects on trade and investment in the region. China export credit insurance, starting from the following 4 aspects, could strengthen the positive psychological incentive and emotional regulation of the agreement, stabilize the psychological expectation of China’s traders and investors to develop market in east Asia: 1. A deep understanding that opportunities brought by RCEP are conducive to better integration of China export credit insurance into the China’s development; 2. Take the bidding list of the contracting parties within RCEP as the starting point, and clarify the specific support direction from China export credit insurance for China’s enterprises; 3. China export credit insurance shall provide timely support to promote China’s enterprises to build a more mature supply chain layout of foreign trade industry among contracting countries within RCEP.

## LHMM23015 THE IMPACT OF RURAL HEALTH HUMAN RESOURCE ALLOCATION ON THE MENTAL HEALTH OF RURAL RESIDENTS

Dawei Yang^a^, Qiyan Jiang^b^

*^a^ Shandong Polytechnic, Jinan, Shandong, 250104, China, ^b^ Zaozhuang Transportation Service Center, Zaozhuang Shandong, 277800, China.*

**Background:** The purpose of this dissertation is to explore the impact of rural health human resource allocation on the mental health of rural residents. The increasingly prominent problem of health human resource allocation in rural areas affects the quality and coverage of medical services and has an important impact on the psychological health of rural residents.

**Subjects and Methods:** Through collecting relevant literature and field research, this paper analyzes the main problems of rural health human resource allocation, including insufficient number of medical personnel, low level of medical technology, and uneven distribution of medical resources. These problems have led to difficulties in accessing medical services and mental health support for rural residents. This dissertation also explores the relationship between rural health human resource allocation and the mental health of rural residents.

**Results:** An unreasonable allocation of health human resources can lead to rural residents’ dissatisfaction and distrust of medical resources, which in turn affects their mental health status. The lack of professional mental health service personnel and psychological support mechanisms is also an important cause of mental health problems among rural residents. However, some positive practices and countermeasures have been proposed, including strengthening the training and introduction of health human resources, improving the mental health literacy of medical personnel, and establishing a sound mental health service network.

**Conclusions:** Through an in-depth study of the impact of rural health human resource allocation on the mental health of rural residents, we can better understand the root causes of the problem and the mechanisms of influence, and make corresponding policy and practice recommendations. Improving rural health human resource allocation and providing better medical services and psychological support are important for promoting the mental health of rural residents. This study provides useful references for relevant departments and policy makers to better respond to the rural health human resource allocation problem and improve the mental health of rural residents.

## LHMM23016 FORMATION MECHANISM OF DEMAND-SIDE USERS’ CREDIT FRAUD FROM THE PERSPECTIVE OF PLATFORM ECONOMY RESEARCH BY CONSUMERS’ MENTAL HEALTH

Kaigang Yi^a^, Jin Yan^a^, Feiqin Li^b^

*^a^ School of Business Administration, Zhejiang Gongshang University, Zhejiang, 310018, China, ^b^ Zhejiang Gongshang University Hangzhou College of Commerce, Zhejiang, 311508, China*

**Background:** Credit is the key mental health factor to support the economic development of the platform, while demand-side users are the direct “fact implementers” of credit fraud on the network platform, but they are also easily overlooked subjects. Studying the formation mechanism of demand-side users’ credit fraud from the perspective of consumer psychology can provide a new direction for the governance of credit fraud on the network platform. Based on the theory of planned behavior and fraud risk factors, this paper integrates and constructs a theoretical model of the mechanism of credit fraud of platform demand-side users through theoretical applicability analysis.

**Subjects and Methods:** On the basis of theoretical deduction, seven hypotheses were constructed, and 312 valid questionnaires were collected by taking the demand-side users of online shopping platform as the empirical research object. On the basis of reliability and validity test, the structural equation model was used to statistically test the fitting degree and research hypotheses of the whole model, and then the nest model method was used for further verification.

**Results:** (1) attitude of credit fraud, subjective norms of credit fraud, and control of perceived behavior of credit fraud have direct positive effects on the intention to commit credit fraud; (2) Perceived risk indirectly affects the intention to commit credit fraud through the control of perceived behavior of credit fraud; (3) Risk preference plays a moderating role in the influence of perceived risk on the control of perceived behavior of credit fraud.

**Conclusions:** According to consumer psychology, credit fraud committed by platform demand-side users is influenced by personal morality and interests, government supervision and punishment, and important stakeholders in society, which provides a certain direction for innovative research on governance countermeasures. (1) At the regulatory level, the government and the platform main body should first pay more attention to the harmfulness of credit fraud of demand-side users of the platform, and speed up the formulation of special programs or special documents to deal with this problem; (2) At the technical level, we should, on the basis of the relatively mature technical discrimination against credit fraud of platform supplier users, draw lessons and innovate technical means to strengthen the transaction management of demand-side users, and use technology to empower supervision and warn fraud, so as to reduce the implementation space of fraud; (3) At the social level, it is necessary to carry out thematic series of publicity and reports in combination with the special actions of industry and commerce department, public security department and other departments aimed at the governance of online platform credit fraud.

### Acknowledgments

This paper was funded by the Youth Project of the National Philosophy and Social Sciences Foundation (19AGL009) and the Zhejiang Philosophy and Social Science Foundation (17NDJC218YB).

## LHMM23017 THE EFFECTS PHYSIOLOGICAL FUNCTIONS OF DIFFERENT SELENIUM SOURCES ON SLAUGHTER PERFORMANCE, MEAT HEALTH AND ANTIOXIDANT ACTIVITY OF DONGXIANG GREEN-SHELL LAYING HENS

Han Yan^a^, Xiaoyu Chang^a^, Qinglan Liu^a^, Jiewei Liu^b^, Ruili Li^b^, Kaiwen Zhao^c^, Dawen Zhang^a^, Yingmei Yu^a^, Huanhuan Wan^a^, Biaojin Zhang^a^, Siming Li^a^

*^a^ Key Laboratory for quality and safety control of poultry products, Ministry of Agriculture and Rural Affairs of the People’s Republic of China, Institute for Quality & Safety and Standards of Agricultural Products Research, Jiangxi Academy of Agricultural Sciences, Nanlian Road 602, Nanchang 330200, China, ^b^ Institute of Animal Science, Yantai Academy of Agricultural Sciences, Yantai, Shandong 265500, China, ^c^ Nanchang Institute of Technology, Nanchang, Jiangxi 330044, China.*

H Yan and XY Chang contributed equally to this study.

**Background:** The trace element selenium is not only one of the essential nutrients for the human body, but also an indispensable element for animal growth and development that is closely related to many important physiological functions of animals. At the same time, a lack of selenium lowers the body’s immune function, which is a hallmark of depression. This experiment was designed to investigate the effects physiological functions of different selenium sources on slaughter performance, meat health and antioxidant activity of Dongxiang green-shell laying hens.

**Subjects and Methods:** A total of 450 30-week-old healthy Dongxiang green-shelled laying hens with similar body weight were randomly divided into 9 groups. All the animals were fed the same basal diet. The control group was fed a non-supplemented diet. T1 and T2 were added sodium selenite (SS).T3 and T4 were enriched with selenium yeast (SY). T5 and T6 were fed with diet comprising Kappa-Selenocarrageenan (SK). And T7 and T8 were supplemented with selenomethionine (SM). And the concentrations of each type of selenium medium added above were 0.30 mg/kg and 0.60 mg/kg, respectively. The prefeeding period was 3 days, and the formal experiment lasted for 28 days.

**Results:** 1) The carcass weight of T7 and T8 were increased significantly (P<0.05) compared with the control group. The rate of half breast meat was higher in T7 group higher than in other groups (P<0.05). The abdominal fat percentage in groups T1, T2 and T4 was higher than that in control group (P<0.05). 2) The ovarian index of Dongxiang green-shelled laying hens increased significantly (P<0.05) in T7 and T8 groups. The spleen index of T2 group was lower than that of control group (P<0.05). The liver index of Dongxiang green-shelled laying hens was not significantly affected by different levels of selenium sources in additive groups (P>0.05). 3) Chromaticity values of T1 and T3 were lower than those of the control group (P<0.05). PH value in T2 and T3 groups was higher than that in control group (P<0.05), while that in T6 group was lower than that in control group (P<0.05). A higher Se level was detected in all experimental groups (P<0.05). And selenium content increased with the increasing supplemental level, and T8 group had the highest selenium content, which was 0.191mg/kg. 4) The antioxidant values of liver and spleen in experimental groups were significantly higher than those in control group (P<0.05). Serum antioxidant values of experimental groups except T1 were significantly higher than those of control group (P<0.05).

**Conclusions:** Different selenium source levels can regulate physiological and immune functions of Dongxiang green-shelled laying hens. Reasonable supplementation of organic selenium sources, especially selenium-rich yeast and selenium-methionine can improve slaughter performance, meat quality and antioxidant capacity of Dongxiang green-shelled laying hens.

### Acknowledgements

This work was supported by two key research and development projects of Jiangxi Province, their project numbers are 20202BBFL63029, JXXTCXNLTS202103 and 20202BBF63011.

## LHMM23018 INVESTIGATION OF GENDER PSYCHOLOGICAL DIFFERENCES IN DATA LITERACY OF PRIMARY SCHOOL TEACHERS IN THE CONTEXT OF BIG DATA

Yuan Wang^a^, Yiwen Cao^a^

*^a^ Institutes of Education, Dalian University, Dalian, Liaoning, China.*

**Background:** The big data era has changed society, including education, requiring teachers to possess data literacy skills. However, little research exists on primary school teachers’ data literacy skills, particularly regarding gender psychological differences. This study aims to investigate potential gender psychological differences in data literacy to promote gender equity in developing these skills. Understanding gender psychological differences can enhance teachers’ ability to use data to improve teaching decisions.

**Subjects and Methods:** The study collects data on the completion process and results of assignments of pre-service elementary school teachers of different genders through an online teaching platform that assigns data analysis assignments in education and teaching fields. The study also uses a questionnaire to gain insight into the current gender psychological differences in data literacy of pre-service elementary and secondary school teachers in China. The questionnaire is divided into two parts, the first part is the basic information of the respondents, and the second part is a survey on the current situation of data literacy of pre-service elementary school teachers of different genders. The study conducts a big data analysis using descriptive statistics, t-tests, and regression analysis to analyze the data. Understanding gender psychological differences can enhance teachers’ ability to use data to improve teaching decisions and promote gender equality.

**Results:** This study analyzed the data literacy of pre-service elementary school teachers of different genders in terms of their completion of data analysis assignments, four dimensions of data literacy, and gender psychological differences. The results showed that male pre-service elementary school teachers have slightly higher data literacy than female pre-service elementary school teachers. The mean score of data literacy of male pre-service elementary school teachers was high, while the mean score of data literacy of female pre-service elementary school teachers was moderate. Male pre-service elementary school teachers had a significantly higher mean score than female pre-service elementary school teachers in the core skills dimension. The regression analysis showed that gender was a significant predictor of data literacy after controlling for age and educational level. The findings suggest the importance of addressing gender psychological differences in data literacy and providing targeted training programs to promote data literacy among female pre-service teachers.

**Conclusions:** The study found significant gender psychological differences in specific aspects of data literacy among pre-service elementary school teachers in the context of big data. Despite similar overall levels of data literacy between male and female participants, targeted interventions are necessary to address these differences and improve the data literacy skills of all pre-service teachers. However, the study’s limitations suggest the need for larger, more diverse samples and objective measures of data literacy in future research. Overall, the study contributes to our understanding of gender psychological differences in data literacy and highlights the importance of addressing these differences to effectively navigate the era of big data in education.

## LHMM23019 THE INFLUENCE OF KNOWLEDGE AND EXPERIENCE OF CHINESE EXCELLENT TRADITIONAL CULTURE ON THE DEVELOPMENTAL TENDENCY OF PROSOCIAL BEHAVIOR OF 130 CHILDREN AGED 5-6 BASED ON CHILDREN’S NATIVE MENTAL HEALTH EDUCATION

Aihua Liu^a^

*^a^ School of Preschool Education, Changsha Normal College, Changsha, Hunan, China.*

**Background:** To explore the influence of Chinese excellent traditional cultural knowledge and experience level on the development tendency of prosocial behavior of children aged 5-6 based on children’s native mental health education.

**Subjects and Methods:** 260 children from 8 classes of 3 kindergartens in XX Province of China were selected as subjects, including 2 in cities and 1 in township, aged 5-6 years, all of whom were native speakers of Chinese. After conducting the prosocial behavior development tendency evaluation on 260 people, 130 children (N=74, W=56) randomly selected from the main test participated in the interview on the knowledge and experience level of Chinese excellent traditional culture and scored. 7 modules of “social adaptation” of 5-6 years old in the Chinese version of the Guidelines for the Development of Children Aged 3-6 years old were used as the evaluation tool of prosocial behavior development tendency; Compile the knowledge and experience of Chinese excellent traditional culture and the main test questionnaire. Statistical analysis was conducted for the two types of results.

**Results:** There is a statistical correlation between the level of knowing and experiencing Chinese excellent traditional culture and prosocial behavior development tendency; The difference of prosocial behavior development tendency between high level group and low level group was statistically significant; There is a statistically negative correlation between the level of knowledge and experience excellent Chinese traditional culture and the modules of “like and adapt to group life” and “abide by basic rules of behavior”, which lead to the risk of mental health education.

**Conclusion:** There is a statistical correlation between the level of knowledge and experience excellent Chinese traditional culture and the development tendency of prosocial behavior of 5-6 year old children, and there is a statistical correlation with some high-level prosocial behavior modules; The unsuitable education mode of Chinese excellent traditional culture has educational risks, which have a negative impact on the mental health of preschool children.

## LHMM23020 DEPRESSIVE SYMPTOMS AND ACADEMIC BURNOUT AMONG COLLEGE STUDENTS DURING THE COVID-19 PANDEMIC: THE MEDIATING ROLE OF PSYCHOLOGICAL CAPITAL

Yun Luo^a^, Chen Ye^a^, Xiaoyi Hu^b^, Yan Jiang^c^

*^a^ School of Education, Zhaoqing University, Duanzhou District Zhaoqing, 526061, China, ^b^ Faculty of English Language and Culture, Guangdong University of Foreign Studies, 2 North Baiyun Street, Guangzhou, 510420, China, ^c^ Psychological Counseling Center, Shanghai University, 99 Shangda Road, Baoshan District, Shanghai, 20444, China.*

**Background:** Since 2020, great social changes have occurred, and people’s mental health have been affected due to the COVID-19 pandemic. The COVID-19 outbreak changed the lifestyle of college students significantly. One of the changes is in learning experience. The shift from traditional, offline, face-to-face teaching to online teaching often requires the use of smart devices. Studies have shown that incorrect or problematic smartphone use will lead to depressive symptoms. The model of recovery from depressive symptoms states that while facing malignant life events, individuals with depression will experience higher levels of hopelessness. Thus, people prefer to explain and attribute malignant life events in negatively and individual attribution will affect the level of academic burnout. This study explored the relationship between depressive symptoms and academic burnout among university students during the COVID-19 pandemic.

**Subjects and Methods:** We conducted an online survey with 3,123 undergraduates from universities in Shanghai from March to April 2020. The study used the nine-item Patient Health Questionnaire, Maslach Burnout Inventory–Student Survey, and Positive Psychological Capital Questionnaire. In this study, SPSS 26.0 was used to perform an independent sample t test for groups between students whose academic performance was affected by the pandemic and students whose academic performance was not affected, and the results demonstrated that it was statistically significant. Second, descriptive statistics and correlations for the two groups were analyzed for three dimensions. Third, AMOS 23.0 was used to construct the structural equation model and analyze it to test whether the mediation model was valid.

**Results:** The results showed that depressive symptoms predicted academic burnout. Psychological capital played a mediating role between depressive symptoms and academic burnout. The higher the level of depressive symptoms, lower the level of psychological capital and higher the degree of academic burnout. Furthermore, the effects among students whose academic performance had been affected by the COVID-19 pandemic were significantly different from those whose academic performance had not been affected.

**Conclusions:** These findings have potential implications for practical measures while facing a pandemic. Positive psychological capital is conducive for college students and for the establishment and maintenance of a stable mental health state during the COVID-19 pandemic. Furthermore, in this study, the effect of psychological capital on academic burnout manifests not only in its own characteristics but also from its opposite side: psychological inflexibility, and scholars have explained this mechanism. Thus, depressive symptoms are significant negatively predicted academic burnout through psychological capital and its four dimensions.

## LHMM23021 CARNIVAL IMAGES AND REAL LIFE: ON THE CARNIVAL POETIC FEATURES AND PSYCHOTHERAPY EFFECT OF FELLINI’S AMARCORD

Hanyang Li^a^

*^a^ School of Journalism and Communication, Minnan Normal University, Zhangzhou, 363000, China*

**Background:** Federico Fellini’s film Amarcord, released in 1973, presents a vivid and surreal world that captures the essence of a small Italian provincial town in the 1930s. The film combines chaos, absurdity, and ordinary life to create a poetic and humorous narrative. Fellini’s unique aesthetic style reconstructs the dreams, dignity, and poetry of ordinary people during that era. This article explores the carnival poetics theory proposed by Mikhail Bakhtin and its relevance to understanding the imagery in Amarcord.

**Subjects and Methods:** The article applies Bakhtin’s theory of carnival poetics to analyze the film’s imagery. The theory describes carnival as a festival that breaks social norms, allowing for subversion and renewal. It emphasizes the universal, inclusive, festive, and dual nature of carnival laughter. The analysis focuses on the structure of Amarcord, the carnival-like atmosphere throughout the film, and its portrayal of universal laughter, festiveness, and duality.

**Results:** The analysis reveals that Amarcord exhibits the key features of carnival poetics as defined by Bakhtin. The film’s structure is a mosaic-like collection of stories that portray the town’s life in a comical manner. Laughter permeates the film, representing a carnival-style banter that is universal and inclusive, embracing all aspects of life. The festive atmosphere in the film reflects a connection between the cycle of seasons and the cycle of human life, emphasizing rebirth and renewal. The duality in Amarcord is evident in its use of laughter to both reflect and criticize societal norms, ideologies, and institutions. This duality offers a thought-provoking perspective on the world, inviting viewers to challenge rigid and stagnant beliefs.

**Conclusions:** Fellini’s Amarcord effectively captures the spirit of carnival through its imagery and narrative. The film’s carnival poetics, influenced by Bakhtin’s theory, presents a world that is simultaneously ordinary and extraordinary. The universal laughter, festiveness, and duality depicted in the film challenge established hierarchies and ideologies, providing a profound reflection on human existence. Amarcord’s exploration of the carnival spirit offers viewers an opportunity for introspection, renewal, and a deeper understanding of the complexities of life. By engaging with the film’s carnival imagery, audiences can experience a psychotherapeutic effect that encourages critical thinking and reevaluation of societal norms. Ultimately, Amarcord stands as a timeless masterpiece that invites audiences to revisit its poetic images and contemplate the inherent truths of the human experience.

## LHMM23022 APPEARANCE DESIGN OF RICE COOKERS BASED ON KANSEI ENGINEERING AND LIFESTYLE THEORY OF PHYSICAL AND MENTAL HEALTH

Hongsu Chen^a^, Yi Feng^a^

*^a^ School of Art and Design, Beijing Forestry University, Beijing, China.*

**Background:** The increasing abundance of material goods and the rising health awareness of the whole population. Healthy living has become the new trend, and healthy eating is an important part of it. Healthy cooking not only reduces the loss of nutrients in food, but also prevents cancer and can effectively reduce the incidence of diseases such as high blood sugar and high blood cholesterol. Maintaining a healthy diet over time reduces the risk of obesity, cardiovascular disease, diabetes and other chronic diseases. Physical health has a positive effect on mental health. At the same time, healthy eating habits can improve the user’s sense of pleasure and contribute to mental health. With the Chinese eating habit of three meals a day, rice cooker is an essential household appliance. Kansei engineering theory is widely used in many fields and provides new ideas for understanding the shape of the user’s favourite smart rice cooker.

**Subjects and Methods:** Based on the design method of Kansei engineering theory, the product appearance design strategy of intelligent rice cooker is studied. Establish a product sample space through market research, extract product design elements, collect perceptual image vocabulary, and then measure users’ perceptual needs using semantic difference method. Key perceptual image words are obtained through factor analysis. Then perform partial correlation analysis on the results of the above methods to identify the corresponding relationship between factors and perceptual image space. Finally, obtain the corresponding relationship between the user’s emotional needs and the design elements of the intelligent rice cooker.

**Results:** Translate the mapping relationship between emotional needs and design elements into design strategies. The more exquisite the smart rice cooker looks outside, the easier it is to win the favor of users. Users choosing smart rice cookers can effectively improve their nutritional level and dietary health, ensure their physical and mental health can balance development, and achieve the goal of physical and mental health.

**Conclusions:** This research mainly uses the research methods of Kansei engineering to guide the appearance design of smart rice cookers, effectively providing new design ideas for the design and development of smart rice cookers, which is conducive to improving consumers’ satisfaction with the appearance of smart rice cookers.

## LHMM23023 ANALYSIS ON THE REFORM OF INTERNAL EVALUATION IN VOCATIONAL COLLEGES: COMPREHENSIVE QUALITY TRAINING BASED ON IMPROVING STUDENTS’ PHYSICAL AND MENTAL HEALTH

Lixin Bu^a^, Lili Huang^a^, Xinmin Yang^a^, Di Wu^a^, Yan Li^a^

*^a^ Chengde College of Applied Technology, Chengde, 067060, China.*

**Background:** The objective of the study is to establish an evaluation system that can mobilize teachers to devote themselves to education, thus cultivating a generation of the new era with overall development of moral qualities, intellectual ability, physical fitness, aesthetic appreciation, hard-working spirit, and healthy mentality. Guided by establishing scientific education objectives to ensure a correct development direction of education, the establishment of the new system starts from the perspective of guiding the psychological health of teachers and students, and shaping their positive qualities. Aimed at cultivating the positive power and qualities of teachers and students, developing their psychological potential, and promoting a full, vibrant, and happy life for them, the evaluation features “improving result, strengthening process, exploring added value, and improving comprehensiveness”.

**Subjects and Methods:** Firstly, based on modern information technology, the study aims to comprehensively showcase the positive psychological factors that lead teachers’ professional growth and students to possess a scientific career, energetic emotions, optimistic personality, and other positive psychological factors. The experimental goals of the study are ensured by scientifically and accurately identifying the core elements that affect teachers’ educational ability and students’ physical and mental health, developing scientific and effective intervention strategies, increasing positive emotions among teachers and students, shaping optimistic personalities, and tapping into psychological potential, so as to maintain the best state of their lives and to achieve happiness in education. Secondly, strategies and design plans will be formulated in research and practice from the aspects of core goals, consensus on concepts, institutional construction, team building, practical promotion, expected results, and demonstration and promotion. A scientifically sound new evaluation model will be formed ultimately, which will guide the implementation of the fundamental task of cultivating morality and virtues, establish standards for improving students’ physical and mental health education and guide the promotion of “ Educating Six Domains Simultaneously”. This model will further lay a foundation for improving the quality control system, adding impetus to the modernization of governance in vocational colleges.

**Results:** The “Five Ones” goals of the reform: to establish a system; to cultivate a new generation; to generate a batch of achievements; to foster virtue through education; to develop a new evaluation model that guides teachers and students to establish correct values and promote the development of physical and mental health.

**Conclusion:** Guided by the overall development of moral qualities, intellectual ability, physical fitness, aesthetic appreciation, hard-working spirit and healthy mind, the new evaluation system targets at guiding teachers to establish the goal of “ cultivating morality and virtues “ and leading students to coordinated development of thought, body, mind, academic ability, and personality. The model will fully utilize information technology to extract core elements that affect teachers’ professional development and students’ physical and mental health. An internal evaluation system will be formulated through Directionality Principle in psychology, helping teachers and students better unleash their potential and creativity. The system highlights the distinct characteristics of vocational education as a type of education, which will promote the high-quality development of the college and play a leading and exemplary role in evaluation reform in corresponding regions.

### Acknowledgement

Research Project of Social Science Development in Hebei Province in 2022 (Project category: key project. Project number: 20220101003).

## LHMM23024 THE IMPACT OF GOVERNMENT ENVIRONMENTAL PENALTIES ON CORPORATE TAX BURDEN AND EXECUTIVE PSYCHOLOGICAL ANXIETY

Xiaoyan Chen^a^, Han Yan^b^

*^a^ School of Economics and Management, North China University of Technology, Beijing, China, ^b^ School of Management, Xiamen University, Fujian, China.*

**Background:** In the context of China’s green development strategy system, corporate environmental governance and executives’ management mode both face great challenges. How to achieve excellent business performance and environmental performance in the market competition raises many questions about executive psychological anxiety. Corporate executives show many unhealthy psychological problems and psychological anxiety, such as short-termism and utilitarianism, they are eager to pursue short-term corporate value and make decisions that harm the environment. It is urgent for enterprises and the whole society to find correct solutions to achieve personal development, sustainable economic and social development. For example, when corporate environmental violations occur, the government’s environmental penalties and enforcement can timely ease such psychological anxiety problems of executives and improve their correct cognition of sustainable environmental governance, and enhance their positive attitude of other business management behaviors such as corporate tax payment.

**Subjects and Methods:** This paper aims to explore the impact of government environmental penalties on corporate tax burden and executive psychological anxiety. This paper subdivides environmental penalties into two dimensions: environmental penalty frequency and environmental penalty intensity based on the breadth and depth of environmental penalties. Then I take Chinese listed firms in heavy pollution industries as research samples, and use OLS regression analysis to test the impact of environmental penalty frequency and environmental penalty intensity on corporate tax burden. Finally, I conducted field interviews with some executives to analyze the impact of environmental penalties and law enforcement on their psychological anxiety.

**Results:** The study finds that both the frequency and intensity of government environmental penalties have a significant positive impact on corporate tax burden, indicating that the more environmental penalty frequency and the greater environmental penalty intensity, the higher level of corporate tax burden. Further analysis shows that the direct influence path of the above effect is the increase of corporate tax payment rate and corporate income tax burden, and the indirect influence path is the decrease of tax shield effect of corporate new bank loan. In addition, the interview study finds that executive psychological anxiety about short-termism and utilitarianism has been effectively alleviated after the government’s environmental penalties.

**Conclusions:** The results show that environmental law enforcement can help ease executive psychological anxiety problems in terms of corporate sustainable development, and enhance their positive attitude of business management behaviors such as environmental sustainability and tax payment. This study reminds executives to focus on long-termism rather than short-termism. In addition, this study has important practical significance and enlightenment for promoting the multi-party co-governance of environmental governance by the government, tax authorities and firms. I suggest that the government, tax authorities and firms should make joint efforts to create a good environmental governance system.

### Acknowledgements

This research was supported by Research Foundation Project of North China University of Technology (Grant No. 110051360002).

## LHMM23025 ADVERSE CHILDHOOD EXPERIENCES AND DEPRESSIVE SYMPTOMS AMONG UNIVERSITY STUDENTS IN CHINA: THE ROLE OF CHARACTER STRENGTHS

Jun Luo^a^, Dandan Wang^b^, Yulan Yu^b^

*^a^ Guangdong University of Science and Technology, Dongguan, China, ^b^ Department of Psychology/ Research Center for Quality of Life and Applied Psychology, Guangdong Medical University, China.*

JL and DW contributed equally to this work.

**Background:** Adverse childhood experiences (ACEs) are associated with pool mental health, whereas few studies paid attention to the potential buffers. This study aims to: (a) establish predictive relationships between ACEs and character strengths (CS); and (b) exam the joint contribution of ACEs and CS to predict depressive symptoms.

**Subjects and Methods:** We used systematic sampling technic to get samples and performed a self-administered survey among students at a university in southern, China. We analyzed data through descriptive statistics, logistic regression model and mediating effect analysis.

**Results:** Linear regression model in the present study showed that history of ACEs predicted lower relationship (p=0.049), enthusiasm (p=0.009), vitality (p=0.0002) domains of character strengths significantly, while vitality (p=1.23e-08) and justice (p=0.03) domains predicted lower depressive symptoms significantly. Vitality domain of character strengths was a full mediator in the relationship between ACEs and depressive symptoms among university students. Indirect effect was 0.25, 95%CI [0.13, 0.39], and direct effect was 0.21, 95%CI [-0.10, 0.50], and total effect was 0.46, 95%CI [0.17, 0.78]. Proportion of mediation was 54%.

**Conclusions:** Vitality domain of character strength mediated the effect of ACEs on depressive symptoms fully. The finding provided mechanism explanation and empirical evidence for future intervention direction among university students with ACEs. However, caveats regarding recall bias, social desirability bias, and lack of generalizability are also recommended in the interpretation of the study findings.

### Acknowledgments

This research was funded by Guangdong Provincial Philosophy and Social Science Planning Project (GD22XJY25) and Postgraduate Education Innovation Project of Guangdong Province (2022JGXM106).

## LHMM23026 EXPLORATION OF CROSS-REGIONAL COORDINATED DEVELOPMENT OF PRIVATE UNIVERSITIES IN THE CHENGDU-CHONGQING ECONOMIC CIRCLE AND MAINTENANCE OF STUDENTS’ PSYCHOLOGICAL HEALTH

Zemei Shen^a^

*^a^ School of Medicine, Chongqing Institute of Engineering, Chongqing, China.*

**Background:** The purpose of this paper is to explore the feasibility of cross-regional coordinated development and cooperation between private universities in the two cities under the drive of the Chengdu-Chongqing economic circle, and its influence on students’ psychological health, and to sort out the problems of students’ psychological health in this process.

**Subjects and Methods:** Literature research method was used to study and analyze the present situation, theoretical basis, driving factors of cross-regional collaborative development of private universities in Chengdu-Chongqing Economic Circle, as well as the importance, convenience and substantive significance of factors influencing students’ psychological health, main problems, and cross-regional development of professional education on the maintenance of students’ psychological health, in the hope of helping private universities in Chengdu-Chongqing Economic Circle achieve cross-regional coordinated development, and also to alleviate students’ psychological problems such as learning pressure and learning anxiety in the process of cross-regional development of professional education in private universities in both places, so as to maintain the psychological health of students in private universities in both places.

**Results:** The study found that private universities are an important part of higher education in China. Under the drive of the Chengdu-Chongqing Economic Circle, a study on the development status of private universities in the Chengdu-Chongqing economic circle and the promotion of cross-regional coordinated development between private universities in the two cities can better promote the scale and cluster-based management of private universities in the Chengdu-Chongqing economic circle, improve the quality of education, and provide more comprehensive talent and intellectual support for the current construction of the Chengdu-Chongqing economic circle. Meanwhile, discussing the influence of the cross-regional development of professional education in private universities in the two places on the maintenance of students’ psychological health can also provide some help for the training of talents in the two places and escort the smooth cross-regional coordinated development of private universities in Chengdu and Chongqing.

**Conclusions:** Private universities in the Chengdu-Chongqing Economic Circle should strengthen cross-regional coordinated development, learn from each other’s strengths, and progress together. Meanwhile, due to the challenges that students may face in the process of cross-regional coordinated development, such as poor resilience, fear of learning, high learning pressure, and anxiety, it is important to pay attention to and jointly maintain students’ psychological health.

### Acknowledgments

**Fund Project:** “Research on Influencing Factors, Mode Construction and Realization Path of Cross-regional Coordinated Development of Private universities in Chengdu-Chongqing Economic Circle” in Research and Planning Project of Humanities and Social Sciences of Chongqing Education Commission in 2022 (22SKGH502); The offline “golden course” of Chongqing Institute of Technology’s “Golden Courses” Construction Project in 2021—Market Survey (KC20210323).

## LHMM23027 EFFECT OF LOWER EYELID BLEPHAROPLASTY VIA TRANSCONJUNCTIVAL ROUTE COMBINED WITH NANO FAT TRANSPLANTATION IN ORBITAL REJUVENATION

Jie Bai^a^

*^a^ Zi Jie Medical Beauty, Beijing 100006, China.*

**Objective:** This study aims to investigate the effect of lower eyelid blepharoplasty via transconjunctival route combined with nano fat transplantation in orbital rejuvenation.

**Methods:** Retrospective analysis was conducted on 126 orbital rejuvenation surgery patients admitted in our hospital from August 2019 to August 2021, who were divided into combined surgery group and conventional surgery group according to the treatment approach. Patients in the combined surgery group received lower eyelid blepharoplasty via transconjunctival route combined with nano fat transplantation, including a total of 76 patients, and patients in the conventional surgery group received lower eyelid blepharoplasty via transconjunctival route, including a total of 50 patients. The recovery results of the two groups at 6 months after surgery were observed, and the tear trough deformity scale scores, patient satisfaction and postoperative complication rates of the two groups were compared.

**Results:** The postoperative TTRS scores decreased in both groups, with more obvious decrease in the combined surgery group than in the conventional surgery group, showing significant difference between the two groups (P < 0.05). The surgical satisfaction rate was 96.05% in the combined surgery group and 80.00% in the conventional surgery group, showing statistically significant difference between the two groups (P < 0.05). The incidence of postoperative complications was 11.84% in the combined surgery group and 24.00% in the conventional surgery group, showing no statistically significant difference between the two groups (P > 0.05).

**Conclusion:** Lower eyelid blepharoplasty via transconjunctival route combined with nano fat transplantation has good efficacy in orbital rejuvenation, with patients’ symptoms significantly alleviated. The surgery has certain safety, and patients have high satisfaction with the postoperative results.

## LHMM23028 ANALYSIS ON THE CONSTRUCTION AND IMPLEMENTATION OF MUTUAL ENCOURAGEMENT AND INTEGRATION MODE OF THE JOINT TRAINING BASE FOR POSTGRADUATES’ MENTAL HEALTH

Xihuang Lai^a^, Zhihui Yang^b^, Zhifei Song^a^, Xiaobo Tao^a^

*^a^ Graduate School, North China University of Technology, Beijing, China, ^b^ Brunel London School, North China University of Technology, Beijing, China.*

**Background:** Improving postgraduates’ mental health will be implemented as an important reference factor for the layout of degree authorization points. The integration between industry and education is an important way to guarantee postgraduates’ mental health. Those joint training bases built on the mutual collaboration provide strong guarantees for graduates in shaping their mental health, cultivating the application ability of basic theory and expanding the field of vision of industry.

**Subjects and Methods:** This paper proposes a strategy of multi-dimensional mutual encouragement and integration, multi-mode construction approaches, as well as multi-dimensional evaluation and guarantee system, which can better solve the problems of shortages of resources, lack of mutual trust in school enterprise cooperation, imperfect operation mechanism and system, which exist widely in local universities and have significant impact on postgraduates’ mental health. In addition, combined with the operating experience of the joint training base for graduate students in relevant universities, an overall management scheme of the bases is given from the viewpoint of base creation, daily management and sustainable development of bases in order to provide practical implementation reference for the construction of bases among local universities.

**Results:** The construction of the joint training base for graduate students of the school has achieved initial results. Up to now, the number of off-campus joint training bases for graduate students has grown from 8 at the beginning to 180 at present, and the production and education integration joint training base has truly achieved a win-win situation between school and enterprise, both of which improve postgraduates’ mental health to a great extent.

**Conclusions:** To improve postgraduates’ mental health, universities should start from the three aspects of “multi-mode training base”, “multi-faceted training guarantee” and “multi-dimensional evaluation system” to build a joint training base construction mode of “multi-dimensional mutual encouragement”. With the overall goal of “professional knowledge plus ability expansion”, the school has established a graduate ability pool and provided ability reports for graduate students. “Multi-mode training base”, “multi-faceted training guarantee” and “multi-dimensional evaluation system” have formed a new model for the construction of joint training bases for graduate students in local universities, and the three influence and promote each other to achieve pluralism and unity, which can effectively mobilize the enthusiasm of enterprises and participate in the construction of practice bases, and improve postgraduates’ mental health significantly.

### Acknowledgments

This article is a phased research result of the 2021 ideological and political work research topic of Beijing colleges and universities by the Education Work Committee of the Beijing Municipal Committee of the Communist Party of China and Beijing Higher Education Teaching Reform and innovation project, “Construction and Implementation of an Evaluation System for the Comprehensive Development of Postgraduates Oriented by Ability Cultivation” (Project No.BJSZ2021ZC63).

## LHMM23029 THE EFFECT OF THE XIAOZHONG ZHITONG MIXTURE ON SKIN FLAP ISCHAEMIA-REPERFUSION INJURY AND GENE EXPRESSION PROFILING BASED ON THE P38 MAPK-PPARΓ/NF-ΚB SIGNALLING PATHWAY

Tuanzhuang Zhang^a^, Yuan Song^b^, Tao Liu^b^, Jiaxuan Shen^a^, Xudong Liang^a^, Jing Qiao^a^, Keyu Zhu^a^, Shenghua Li^b^

*^a^ Gansu University of Traditional Chinese Medicine, School of Traditional Chinese Medicine, Lanzhou 730000, Gansu Province, China, ^b^ Gansu Hospital of Traditional Chinese Medicine, Hand Surgery, Lanzhou 730050, Gansu Province, China.*

**Background:** This study aims to investigate the effect of Xiaozhong Zhitong mixture on flap ischaemia-reperfusion (I/R) injury and its action mechanism.

**Methods:** Sixty SPF SD rats were randomly divided into six groups (n=10 each): sham operation group, model control group, Xiaozhong Zhigang mixture group, p38MAPK inhibitor group, PPARγ inhibitor group, and NF-κB inhibitor group. Random flaps (8cm×2cm) were constructed on the back of rats except those in sham operation group. After operation, the flaps were sutured in situ with 3-0 suture to establish the flap I/R injury model. At 7 days after surgery, the rats were observed morphologically and the levels of TNFα, IL-6 and intercellular adhesion molecule-1 (ICAM-1) were measured. Western blotting was used to detect the protein expression of p38 MAPK-PPARγ/NF-κB pathway. The whole genome chip was used to screen the differentially expressed genes after treatment, and the expression patterns of the differentially expressed genes were analyzed by clustering, GO and KEGG. The differentially expressed genes and the biological processes and the most important signal transduction pathways in the process of Xiaozhongzhipain mixture in the treatment of flap ischemia reperfusion injury were screened. The highest and lowest temperatures of the flaps of rats were detected by infrared thermography, and the edema and leukocyte infiltration of the flaps were observed by hematoxylin-eosin staining.

**Results:** On POD 7, the survival area of rats in the Xiaozhong Zhitong mixture group, p38 MAPK inhibitor group, and NF-κB inhibitor group increased. The levels of TNFα, IL-6 and ICAM-1 were decreased by Xiaotumorzhidong mixture, p38 MAPK inhibitor and NF-κB inhibitor. Xiaozhong Zhutong mixture can up-regulate the expression of PPARγ and IκBα, and down-regulate the expression of p38MAPK and NF-κB. The whole genome microarray analysis showed that the attenuation of flap I/R injury by Xiaotumoranalgesic mixture was related to inflammatory response, immune system process regulation, cell proliferation, cell adhesion and neutrophil migration. Xiaotumorzhidong mixture can alleviate flap I/R injury in PPAR signaling pathway, MAPK signaling pathway and NF-κB signaling pathway.

**Conclusions:** The Xiaozhong Zhitong mixture may reduce I/R injury in rat flaps by modulating the p38 MAPK-PPARγ/NF-κB pathway.

### Acknowledgments

This work was supported by a grant from the Regional Project of the National Natural Science Foundation of China (Grant No.81860863).

## LHMM23030 ANALYSIS OF CARBON EMISSION INTENSITY AND ITS IMPACT ON RESIDENTS’ HEALTH IN LIUPANSHUI CITY UNDER THE VISION OF “CARBON NEUTRALITY”

Chaoyan Deng^a,b^, Chunyan Dai^a,c,d^

*^a^ Rattanakosin International College of Creative Entrepreneurship, Rajamangala University of Technology, Phuttamonthon, Nakhon Pathom 73170, Thailand, ^b^ School of Chemistry and Materials Engineering, Liupanshui Normal University, Liupanshui, Guizhou 553004, China, ^c^ School of Management Science and Engineering, Chongqing Technology and Business University, Chongqing 400067, China, ^d^ Development Information Management Engineering Technology Research Center in Chongqing, Chongqing 400067, China.*

**Background:** As the social progress and economic development have brought about an increasingly warming climate, countries have promised their carbon neutrality goals, and China has solemnly promised to the world that it will achieve carbon neutrality in 2060. For this reason, the relevant carbon emission accounting and scenario prediction are particularly important to provide the technical and theoretical basis for urging the low-carbon transformation of relevant industries. Under the current local policies of industrial revitalization and characteristic tourism development, it is of great significance to analyze and study the carbon emission intensity and residents’ health under the vision of “carbon neutrality” for Liupanshui as an old industrial city in the former third-line construction, the Coal Capital in the Regions South of the Yangtze River and the Cool Capital of China.

**Subjects and Methods:** In this study, the energy consumption and production carbon emissions of four administrative regions including Zhongshan District, Shuicheng District, Liuzhi Special District, and Panzhou City, which constitute Liupanshui City, were calculated from 2011 to 2020. The carbon emissions increment model of Liupanshui City was constructed by the Logarithmic Mean Divisia Index (LMDI) method, and the impact of carbon emissions on residents’ health in Liupanshui City was studied through qualitative analysis. On this basis, the spatial distribution model of carbon emissions in Liupanshui was constructed by using big data, energy consumption data and infrastructure spatial distribution data. The driving force and influencing factors of carbon emissions in Liupanshui were analyzed by using historical data of Liupanshui, and the internal mechanism of carbon emissions, socio-economic factors and spatial form indicators was explored.

**Results:** The results show that in recent ten years, the overall trend of carbon emissions in Liupanshui is wavy, the trend of carbon emissions per capita is similar to the intensity of carbon emissions, and the intensity of carbon emissions shows a steady downward trend. The empirical analysis of carbon emission increment of energy consumption in Liupanshui shows that the carbon emission increment caused by economic scale effect first increases and then decreases, the carbon emission increment caused by regional structure effect changes little, the carbon emission increment caused by technological progress effect is sometimes positive and sometimes negative, and the carbon emission increment caused by energy consumption structure effect is similar to technological progress effect. Extreme weather caused by greenhouse gases indirectly harms people’s physical and mental health, induces an increase in the incidence of cardiovascular and respiratory diseases, and accelerates the spread and spread of epidemic diseases. Based on people’s recognition, local residents are actively making improvements towards a healthier lifestyle. In addition, the government is also strengthening the introduction of relevant low-carbon policies.

**Conclusions:** The above research results will provide data support and decision-making suggestions for energy conservation and emission reduction, urban planning, and related scientific research, and policy suggestions on how to implement energy conservation and emission reduction planningas well as feasible solutions on how to achieve the goal of “carbon neutrality” in Liupanshui in the future, a good environment for physical and mental health of Liupanshui residents.

### Acknowledgments

The authors gratefully acknowledge the financial support of Guizhou Social Sciences “Theoretical Innovation Project” special fund supported project (No. GZLCZB-2022-3-5-1); Philosophy and Social Science Project of Liupanshui City (No. lpsskllh-2022-76).

## LHMM23031 EFFECTS OF EMBODIED LEARNING METHOD ON CHINESE CHARACTER LEARNING AND STUDENTS’ ANXIETY

Mingmin He^a^, Zhen Ren^a^

*^a^ Sichuan University Jinjiang College, Meishan 620860, China.*

**Background:** The purpose of this study was to investigate the effectiveness of two embodied learning approaches, finger-tracking, and observation-tracking, in Chinese character learning and to explore their relationship with learners’ working memory and students’ anxiety.

**Subjects and Methods:** A total of 31 undergraduate students from non-native Chinese speaking universities, of whom 7 were male and 24 were female, participated in our study. The working memory capacity was measured using the Visual Patterns Test (VPT). The study materials included 36 kanji (12 kanji for each teaching style). We measured the effectiveness of learning through two rounds of tests: immediate post-test and delayed post-test. The effectiveness of finger tracking and observational finger tracking in Chinese language learning was investigated empirically by comparing the performance of the finger tracking group, the observational tracking group and the control group on the recognition and cue recall tasks in the post-test and the delayed post-test. As an indication of experienced anxiety during the learning phase and directly after each of the post-test phases participants were questioned on a 7-point anxiety scale.

**Results:** We found that: 1) the observation tracking learning method could improve the performance on the delayed post-test during the learning of Chinese characters for beginners, resulting in better retention of learning effects and was effective only for learners with low working memory capacity; 2) the finger-tracking learning method had a negative impact on performance in Chinese character learning, especially for learners with low working memory capacity; 3) students in the observation tracking task report significant less anxiety compare to in the finger-tracking learning task and control learning task; students in the finger-tracking learning task report the higher anxiety than in other tasks.

**Conclusions:** The results of this study suggest that when designing instruction for Chinese language learning, instructors should choose a reasonable teaching style. Observational tracking learning as a form of embodied learning helps Chinese language learning and reduces students’ anxiety.The finger-tracking learning method had a negative impact on performance and increased students’ learning anxiety, especially for learners with low working memory capacity.

## LHMM23032 USERS’ PSYCHOLOGICAL ANXIETY AND LEGAL RISK RESPONSE OF BLOCKCHAIN MANAGEMENT OF TRADE SECRETS IN THE METAVERSE FROM THE PERSPECTIVE OF CHINA

Shiyong Song^a^

*^a^ School of Political Science and Law, Qilu University of Technology (Shandong Academy of Sciences), Jinan, Shandong 250353, China.*

**Background:** The management and legal protection of trade secrets is the common goal of all countries. At the same time, however, the issue of trade secrets profoundly reflects the psychological game between obligees, obligors and infringers. In judicial practice, a large number of infringement cases are actually done maliciously by the infringer based on the unhealthy psychology of prying, which also causes psychological anxiety to the business secret manager that he may leak at any time. Metaverse, as a new concept, has produced great social influence in the economic field of various countries. Blockchain protection of trade secrets is considered to be the best mode in the meta-universe. The characteristics of decentralization and traceability of Blockchain technology can limit the infringer’s unhealthy psychology of snooping, reduce the inner anxiety of trade secret managers, improve their psychological satisfaction and professional happiness, and promote the benign development of trade secret protection.

**Subjects and Methods:** Taking Russia and India of the Shanghai Cooperation Organization for reference, through literature review and empirical analysis, this paper probes into various legal problems faced by countries with trade secret laws and countries without special trade secret protection laws in the two-way psychological game between trade secret owners (managers) and infringers who apply this law. Compared with China’s anti-unfair competition law as the main mode of regulating trade secrets, Russia has a special trade secret protection law. India has not included trade secrets in its intellectual property protection system. India’s trade secret protection mainly provides comprehensive protection through its criminal law, contract law and information technology law. In the face of different legal provisions, anyone will always choose his own behavior mode according to the higher and closer standards of his psychological happiness index. The purpose of this study is to explore the psychological obstacles faced by users at all nodes of Blockchain under the cosmic background (strong protection psychology of obligee and malicious snooping psychology of infringer), as well as various legal risks and coping strategies at this time.

**Results:** Although the meta-universe has exerted great social influence in the economic fields of various countries, on the whole, the meta-universe is only a beautiful idea under the kidnapping of various kinds of capital, and it does not have the conditions for special legal regulation. Users of Metaverse can’t completely move freely without Blockchain technology, which has caused their psychological limitations in behavior, which is reflected in the protection of trade secrets. They don’t have a higher sense of happiness and satisfaction psychologically because of Blockchain technology, but they continue to need to guard against infringement at all times. At the same time, due to the transnational characteristics of users under the background of Blockchain technology and meta-universe, this psychological fear will be further amplified.

**Conclusions:** Compared with the traditional environment, the meta-cosmic scene is more likely to cause more psychological obstacles to the trade secret owners, and generally has a distrust of new technologies, thus affecting their normal social communication ability. At present, the manipulation of the Metaverse by capital magnifies people’s abnormal snooping psychology and unhealthy psychology of illegal possession of social public assets, which is extremely unfavorable to the protection of trade secrets.

### Acknowledgments

This work was supported by: Shandong Philosophy and Social Science Planning Project---Study on the Overall Risk of Blockchain Protecting Trade Secrets in Meta-Universe (Grant No.22CFXJ03); The major special project of regional and country studies (Research on the Development of Science, Technology and Industry in Shanghe Region) of Shanghai Cooperation Organization of Qilu University of Technology (Shandong Academy of Sciences) (Grant No.2022-122).

## LHMM23033 ANALYSIS OF INFLUENCING FACTORS OF KNOWLEDGE, ATTITUDE AND PRACTICE MODEL ON QUALITY OF LIFE OF ELDERLY PATIENTS WITH ACUTE MYOCARDIAL INFARCTION AND ITS INTERVENTION

Yuxia Zhu^a^, Wenjuan Yan^a^, Dixia Qi^a^, Hui Yao^a^, Shuxian Li^a^, Juan Zhao^a^, Nan Kang^a^, Rongyan Zhang^a^, Aiqin Xu^a^, Fang Liu^a^, Fang Liu^a^, Juan Zhao^a^, Yinghui Pan^a^, Yongfang Feng^a^, Ningli Sun^a^, Jing Xu^a^, Shunda Liu^a^, Wenjuan Zhang^a^, Guilian Ma^a^

*^a^ Ningxia Hui Autonomous Region People’s Hospital, Yinchuan City, Ningxia, 750002, China*

**Background:** To explore the effect of knowledge-belief-practice model on influencing factors of quality of life in elderly patients with acute myocardial infarction (AMI).

**Subjects and Methods:** 124 elderly patients with AMI were randomly divided into control group and observation group with 62 cases in each group. The control group was given routine nursing through clinical pathway, while the observation group was given questionnaires by patients’ general questionnaire and quality of life rating scale (WHOQOL-BREF) on the basis of the control group, and the results were analyzed by single factor and multivariate analysis.

**Results:** In the observation group, sex, marital status, educational level, heart function at admission, hospitalization times and payment methods of medical expenses were the main factors affecting the quality of life of elderly AMI patients. Logistic regression analysis showed that resilience (CD-RISC), health belief (CHBMS), depression (SDS) and anxiety (SAS) were the influencing factors of comprehensive scores of elderly AMI patients. Taking the above influencing factors as the main problems of intervention research, the KAP model was applied to intervene, and the effects of the two groups before intervention and one week after intervention were compared. After one week of intervention, the scores of CD-RISC and CHBMS in the observation group were 76.89 ± 6.82 and 141.12 ± 22.28 respectively, which were higher than those in the control group (P < 0.05); SDS and SAS scores of patients in observation group were lower than those in control group (P < 0.05). After the intervention, the scores of knowledge, belief and behavior in the observation group were higher than those in the control group (P < 0.01).

**Conclusions:** KAP model can effectively improve the resilience level, health belief and negative emotion of elderly AMI patients.

### Acknowledgments

This work was supported by Ningxia Hui Autonomous Region Science and Technology Benefiting the People Project (2022KJHM0020).

## LHMM23034 SAFE CONSTRUCTION SUGGESTIONS ON MENTAL HEALTH OF CONSTRUCTION PERSONNEL

Jianlei Wang^a^, Qianyu Zhuang^b^, Junyi Duan^c^, Jin Xu^c^, Zhanbiao Zhao^a^, Ping Zhang^a^

*^a^ AECC Beijing Institute of Aeronautical Materials, Beijing, China, ^b^ National Innovation (Qingdao) High speed Train Material Research Institute Co. LTD, Qingdao, China, ^c^ School of Environmental Science and Safety Engineering, Tianjin University of Technology, Tianjin, China.*

**Background:** With the rapid development of the construction industry, the number of safety accidents is increasing. Safety psychology is a science that studies people’s psychological state and its changes in production activities. It can point out the direction for how to mobilize the enthusiasm of construction workers to reduce the occurrence of safety accidents. Therefore, how to analyze the psychological problems of construction workers from the perspective of safety psychology, find out the main conflicts causing psychological problems and put forward safety strategies has become a new research task.

**Subjects and Methods:** Based on safety psychology, this paper analyzes and points out the unsafe psychology and occupational health problems of construction workers in the construction industry, as well as the conflict relationship between individuals, families and enterprises (industries) environment, etc., and puts forward safety strategy suggestions for the application of safety psychology in the construction industry. It can achieve the purpose that construction workers have correct occupational health and safety psychology and reduce safety accidents, thus promoting the healthy development of individuals and enterprises.

**Results:** Before the accident, the construction workers generally had fluky psychology, burnout psychology, rebellious psychology, paralysis psychology and conformity psychology, and the prominent occupational health and safety psychological problem was burnout psychology. There are many factors that cause unhealthy psychological problems, such as individuals themselves, families, and enterprises (industries). All parties should pay more attention to the psychology of construction workers, be good at self-regulation, build a harmonious family relationship, and improve the mechanism of enterprises (industries), starting from the maintenance of mental health and the implementation of safety strategies.

**Conclusions:** With regard to the safety strategy of construction workers in the construction industry, it is necessary to explore the psychological activity rules and contradictions of construction workers under the guidance of the theory of safety psychology, so as to accurately predict the unsafe behaviors of construction workers, adjust and deal with unsafe psychology with scientific methods, prescribe the right medicine, and avoid unsafe psychological problems and safety accidents. The safety strategy based on safety psychology has very important theoretical and practical significance for reducing the frequency of safety accidents in construction.

## LHMM23035 RESEARCH ON THE INDIVIDUALIZED AEROBIC FITNESS PROGRAM FOR COLLEGE STUDENTS BASED UPON ARTIFICIAL NEURAL NETWORK

Rong Huang^a^, Lei Wang^a,b^

*^a^ School of Physical Education, Shaanxi University of Technology, Hanzhong, 723001, Shaanxi, China, ^b^ School of Computer Science and Engineering, Xi’an University of Technology, Xi’an, 710048, Shaanxi, China*

**Background:** The World Health Organization has recently recommended the amount of aerobic exercise for teenagers. However, the guidelines are more instructive than practical, and cannot be directly used as the individualized aerobic fitness program for college students. Under the background of “combination of sports and medicine”, the principle of “suiting the remedy to the case” followed by medical rehabilitation work is also applicable to the sports fitness programs. Meanwhile the manner in which to apply machine learning to individualized aerobic exercise programs has attracted more and more attention. In this research it assumed that the relationship between human body data indicators and aerobic fitness program could be expressed by an artificial neural network.

**Subjects and Methods:** The research aimed to verify the feasibility and effectiveness of constructing the functional relationship between college students’ static body data and aerobic fitness programs based on artificial neural network, and realize the individualized aerobic fitness activities for college students with the assistance of smart bracelets and mobile phones. 60 normal college students (28 females) were recruited from Shaanxi University of Science and Technology to measure their static body data, meanwhile the test for heart rate under the aerobic exercise with different intensity and duration was carried out by smart bracelet. Based on the target heart rate (THR), a 3-layer artificial neural network between the static body data of the experimental object and the aerobic fitness program was constructed.

**Results:** We had 52 participants (29 males and 23 females) to complete the experiment on schedule. The 3-layer artificial neural network was constructed by Back Propagation Algorithm which was iterated 5 times, and the relationship between human body indicators and running program was represented by the artificial neural network with 17 parameters in hidden layer and 10 parameters in output layer. However, because of the neglect of the lifestyle and chronic diseases for participant, 2 of 20 college students who engaged in the final test got unexpected result.

**Conclusions:** The college students could achieve THR in exercise according to motion speed and duration of running provided by the artificial neural network without fitness professional. Considering the effectiveness, convenience and popularity, the running was selected as the aerobic fitness event, but there were many other aerobic exercises we could use. The 3-layer artificial neural network could be further optimized through multi-dimensional information obtained from different channels and multi-objective analysis method, by which it could promote the applicable scope of the 3-layer artificial neural network. At the same time, with the rapid development of cloud computing, big data and 5G technology, more complicated, efficient and convenient intelligent exercise prescription system will be constructed.

### Acknowledgments

This work was supported by a project grant from the Western China project of National Natural Science Foundation of China (Grant No.21XTY012).

## LHMM23036 RISK RESISTANCE AND MOOD-MODULATING: A VITAL EXPLANATORY FRAMEWORK FOR PATIENT TRUST GENERATION MECHANISMS

Rui Tan^a^, Junfan Xiao^a^

*^a^ College of Humanities & Social Sciences, Huazhong Agricultural University, Wuhan, China.*

**Background:** Patients’ trust deficit in doctor-patient relationships in China has caught research attention in recent times. The risk of trust between doctors and patients makes patients often suffer from anxiety, hesitation and other Negative emotions, which leads to the failure of diagnosis and treatment. Therefore, it is of great practical significance to explore the basis of trust between doctors and patients.

**Subjects and Methods:** This study presents a framework for the effects of patients’ risk resistance regarding patient trust, having applied multivariate regression models to analyse the causes of the patient trust deficit, based on data from the China Family Panel Studies (CFPS, 2018).

**Results:** It was shown that in terms of economic resilience, more comprehensive medical insurance and higher income among patients represented their capability to resist the economic risks induced by medical care, and that their willingness to establish good patient trust was stronger. In terms of social resources, higher social status levels and more stable work statuses improved patients’ capability to handle the risk of medical resources and gave them the confidence to receive good medical services, which positively influenced the establishment of patient trust. With respect to health literacy, sound physical health conditions improved patients’ capability to handle life safety risks.

**Conclusions:** The results show that the lack of patient risk tolerance is the deep-seated reason it is difficult to build patient trust, which is reflected in the fact that the level of patient trust decreases when patients’ economic resilience, social resources, and health literacy are insufficient. Therefore the government should embed the improvement of patients’ risk resistance into all aspects of development of the medical field, pay attention to the assessment of risks faced by patients, and promote the sustainable development of patients’ risk resistance.

### Acknowledgement

This study was supported by the Fundamental Research Funds for the Central Universities (Grant numbers: 2662021JC007).

## LHMM23037 SHAPE SCALE AND PSYCHOLOGICAL HEALING VALUE OF ARCHITECTURAL SAPCEOF MOGAO CAVES IN DUNHUANG

Xueyan Jin^a^, Tian Li^a^

*^a^ College of Arts, Gansu University of Political Science and Law, Lanzhou 730070, China.*

**Background:** The construction of the Mogao Caves was influenced by Indian cave temples and embodies the characteristics of traditional Chinese wooden architecture, representing a process of localization of cave architecture. The architectural structure and spatial form exhibit distinct historical and regional features under the influence of geological environment, excavation technology, and subjective psychological perception of cave space by social groups. Forced by the expectation of survival, the lower class people can extricate themselves from the suffering of reality by worshiping Buddha piously, while the grottoes provide people with a space for spiritual practice, chanting and worshiping. The solemn and sacred atmosphere in the space promotes the frescoes of good and evil causality, and the dignified colored sculptures can heal people’s psychological and physiological health, build their values and even promote the renewal of personality changes, help improve their psychological resilience, reduce stress and anxiety, improve their psychological emotions, and place beautiful expectations on life. It has positive significance in mental health and psychological rehabilitation.

Subject and Methods: The shape of the main chamber roof in Mogao Caves is mainly divided into flat top, even Ceiling, herringbone drape and truncated pyramidal ceiling top. The number of caves Pyramidal Ceiling top is the largest, it was found that its spatial volume and top slope value showed an increasing trend. It provides a broad field of vision and enhances fluidity, making it possible to create large-scale three-dimensional sculptures and murals. In addition, the transparent and spacious top of truncated pyramid ceiling of cave forms a perspective effect with algae wells that progressively recede from outside to inside at different levels, extending users’ psychological space while reducing feelings of psychological oppression. This is conducive to creating a solemn, peaceful, profound and sacred heavenly world. Devout believers will be integrated with the cave space and consciously immersed in the quiet and harmonious space atmosphere, so that their body and mind can be relieved and their spiritual and emotional sustenance can be relaxed; So as to alleviate the psychological pressure, anxiety, helplessness and other negative emotions brought by real life, maintain psychological balance and ensure mental health.

**Results:** Although ancient people lacked systematic scientific exploration and analysis in constructing caves, they established relatively mature systems for cave structure shapes, proportional scales and environmental psychological mapping through persistent practical exploration and accumulated experience. The Mogao Caves’ architecture not only reflects the progress of construction technology but also shows the development of users’ psychological needs. As experiencers and users of cave architecture, people’s ideology will penetrate every aspect of cave construction. Quantitative factors, such as cave structure, shape, spatial scale, color pattern, etc., will also guide and sort out people’s negative emotions, which is beneficial to mental health.

**Conclusions:** This paper discusses the construction technology of the caves from the space layout, scale, psychological atmosphere and other aspects, and discusses many influencing factors in the caves construction, which provides reliable information for studying the structural characteristics of the caves and the psychological mapping of users to the caves in vision and even subconscious. It is helpful for us to know the outstanding wisdom of the ancients in underground engineering technology and architectural psychology. Find out the positive influence of cave architecture on people’s mental health.

### Acknowledgments

In addition to the influence of geographical environment and rock structure, the change and development of the structure and scale ratio of cave architecture are also related to the rule of the regime at that time, the life of the common group and the functional needs of the caves. From the perspective of scientific exploration and using computer simulating test, four models were established based on the same conditions, and the deformation and principal stress of Mogao Caves were compared, and it is found that the stability of pyramidal ceiling > even roof > herringbone > flat roof. Although the excavation was limited by cutting equipment and technique at that time, the top structure of caves cleverly utilized the structure and characteristics of rock mass, and the sequence of excavation from top to bottom reduced the influence on the surrounding rock of caves. Under the condition of no supporting lining, the stability and self-supporting ability of surrounding rock were guaranteed, and the demands of culture spreading and exhibition were taken into account in different times, showing the wisdom and engineering experience of ancient Chinese craftsmen, which had certain guiding significance for underground engineering construction. 2021 Young Doctor Fund Project of Gansu Province (Project No.: 2021QB101).

## LHMM23038 A STUDY ON CHARISMATIC LEADERS ON ALLEVIATING EMPLOYEES’ PSYCHOLOGICAL ANXIETY AND IMPROVING WORK PERFORMANCE

Jia-qing Wang^a^

*^a^ School of Economics and Management, Wuhan University of Engineering Science, Wuhan 430200, Hubei, China.*

**Background:** The essence of psychological anxiety is a kind of psychological sub-health, which is externally manifested as negative and anxious emotions of employees’ work attitude and behavior. In enterprise management, there is a basis for psychological anxiety in addition to personal characteristics, it is also related to the job position, job responsibilities and the management style of superior leaders, especially related to the company’s principle of mutual benefit. The principle of mutual benefit is the basic factor for the harmonious coexistence of the organization, leaders and employees, and it is also the pillar of the maintenance of the organizational system, the implementation of leadership activities and the development of work behavior. If the principle of mutual benefit is not well guaranteed, then psychological anxiety will lose its space for existence, and correspondingly, organizational operation, leadership behavior and work behavior can be smoothly implemented. The principle of interest and emotion is the core of psychological anxiety, and it is also the legal principle of relationship performance and production performance. Charismatic leaders can have a positive effect on employee performance by alleviating psychological anxiety as an intermediate variable. Based on the internal logic of charismatic leadership traits and psychological anxiety, the relationship between psychological anxiety and employee performance and the mechanism of charismatic leadership and employee psychological anxiety alleviation were analyzed.

**Subjects and Methods:** The research goal of this paper is to explore the positive leading role of alleviating psychological anxiety on employee performance from the perspective of organizational operation. The article argues that a leadership style that is highly consistent with the behavior of alleviating psychological anxiety is needed, that is, leadership ability and leadership art that is appealing and attractive. At the same time, based on the internal analysis and examination of the characteristics of charismatic leadership style, it is found that this leadership style feature is compatible with psychological anxiety behavior, and can stimulate employees’ work initiative and enthusiasm in the form of alleviating psychological anxiety, thereby improving employee relationship performance and effectively interacting with organizational performance. By using a variety of statistical analysis techniques such as content analysis, literature analysis, exploratory factor analysis, confirmatory factor analysis, structural equation model and regression analysis, the influence of charismatic leadership on employee performance was studied with psychological anxiety as the mediating variable.

**Results:** Through theoretical analysis and empirical research, it is found that the personal charisma traits of leaders constitute the main dimension of charismatic leadership. From the perspective of leadership behavior practice, the dimension of charismatic leadership is specifically manifested in the following aspects, namely: common vision, caring for employees, paying attention to the environment, daring to take risks and innovation, and extraordinary behavior. Charismatic leadership can play a role in alleviating psychological anxiety because of the application of these five elements. Based on a shared vision, charismatic leaders are usually exemplary and benchmarking to reinforce the alignment of employees with their own tasks, and guide employees to make efforts to improve organizational performance. The impact of charismatic leadership on employee performance is manifested as: the role model of charismatic leadership can strengthen employees’ engagement in work; The vision of charismatic leaders can effectively awaken employees’ work motivation; The personality factors of charismatic leaders can better enhance employees’ loyalty to the organization; Charismatic leaders care for their jobs and improve employee satisfaction.

**Conclusions:** This paper proposes a path for charismatic leaders to improve employees’ performance from the perspective of alleviating psychological anxiety: focusing on the consistency between charisma traits and alleviating employees’ psychological anxiety; Organizational commitments and rules for alleviating psychological anxiety construction should be followed; Caring about job satisfaction in the dimension of employee psychological anxiety; Lead and inspire employee loyalty to the organization.

## LHMM23039 THE APPLICATION OF PAINTING PSYCHOANALYSIS IN THE MENTAL HEALTH EDUCATION OF BANGLADESHI FOREIGN STUDENTS IN HIGHER VOCATIONAL COLLEGES

Junjun Chen^a^

*^a^ Student Affairs Office, Shandong Polytechnic, Jinan, Shandong 250104, China.*

**Background:** In recent years, more and more foreign students have entered higher vocational colleges in China. Mental health education of foreign students in higher vocational colleges is facing great challenges.

**Subjects and Methods:** The purpose of this paper is to explore the application of painting psychological analysis in the mental health education of Bangladeshi students in higher vocational colleges, and to explore a new way to promote the harmonious development of physical and mental health of foreign students in higher vocational colleges. A total of 50 Bangladeshi students in Shandong Polytechnic were enrolled in the study. This paper evaluates the reliability and validity through qualitative research.

**Results:** Painting psychoanalysis, which is characterized by its variety, easy operation and freedom from time and space constraints, is mainly used as a means of communication to bring more vitality and enlightenment to the mental health education of college students. The physical and mental health of Bangladeshi students in vocational colleges were fully developed and achieved psychological growth. The classroom atmosphere is more active. The students’ psychological stress was significantly reduced, and their self-identity significantly increased.

**Conclusions:** The psychological analysis of painting is integrated into the mental health education activities of the foreign students in Bangladesh, which is very popular among the foreign students. Through communication, teachers, counselors, students generally agree that painting activity has achieved good results. From the practice, it is found that the psychological analysis of painting is helpful to overcome the negative influence of culture and language, and is suitable for Bangladeshi students to carry out mental health education.

## LHMM23040 EXPLORATION OF INNOVATIVE CULTIVATION OF CRITICAL THINKING ABILITY OF ENGLISH MAJORS FROM THE PERSPECTIVE OF EDUCATIONAL PSYCHOLOGY

Yadong Wang^a^

*^a^ School of Education, Nanchang Institute of Science and Technology, Nanchang, Jiangxi, China.*

**Background:** To explore effective ways of cultivating critical thinking quality of English majors from the perspective of educational psychology, especially the willpower. For willpower plays an important role in the entire process of cultivation of critical thinking quality. Students who possess strong willpower can consciously control and adjust their actions according to the purpose, and can overcome various difficulties to achieve the purpose. Also a strong willpower will usually lead to a strong determination, which is a key factor to success. A strong willpower is also helpful in cultivating students’ healthy psychological mindset and character building.

**Subjects and Methods:** 1. The willpower and motivation method: First, purpose education. To educate the students about the purpose and necessity of cultivating critical thinking quality. Then, turn them into a strong motivation. Third, educate the students about the importance of a strong willpower.When meeting difficulties, encourage them to get over difficulties by their strong willpower. 2. The willpower cultivation method: Besides educating students about the theoretical side of willpower, the teacher also educate them about the practical side, i.e. how to obtain a strong willpower. The teacher adopts various ways to cultivate students’ physical and psychological willpower. To cultivate students’ physical willpower, the teacher requires students to make a very specific plan for physical exercise. For example, running a certain distance every day and stick to it strictly. To cultivate students’ psychological willpower, the teacher requires students to memorize a certain number of English vocabulary every day and stick to it strictly. The teacher controls the process by checking the students’ progress periodically and establish a certain reward mechanism. In this way, help students cultivate a strong willpower as well as a healthy psychological mindset. 3. Integration method: The elements of critical thinking are integrated into classroom teaching activities such as text teaching, writing teaching, reading teaching. Through text analysis, main idea summary and question answering, students’ logical thinking ability is exercised and developed. Through critical writing practice, students’ critical ability is trained and developed.

**Results:** 1. Through teacher’s education of the purpose, the students know the importance and necessity of cultivating critical thinking quality. Through teacher’s willpower and motivation education, the students know the important role the willpower plays in the cultivation process. The students’ learning enthusiasm is greatly aroused with a strong motivation. 2. The logic theory required in the cultivation process is usually abstract, dull and hard to understand. However, through teacher’s willpower education and cultivation, the students’ strong willpower is gradually obtained and further enhanced. Thus, it enables the students to get over various difficulties occurred during the cultivation process. Meanwhile, it also helps the students develop a healthy psychological mindset and a good character.

**Conclusions:** English teaching integrated with cultivation of critical thinking of English majors from the perspective of educational psychology is feasible. For willpower plays an important and irreplaceable role in the cultivation of critical thinking quality and a healthy psychological mindset. In colleges that do not offer a separate course on critical thinking, this method is both realistic and useful.

## LHMM23041 AN EMPIRICAL STUDY ON THE IMPACT OF FINTECH ON RETAIL BANKING: FROM THE PERSPECTIVE OF PSYCHOLOGICAL SATISFACTION

Mingchi Zhu^a^, Huayu Guan^b^

*^a^ Beijing-Dublin International College at BJUT, Beijing 100124, China, ^b^ Finance Office, School of Emergency Technology and Management, North China Institute of Science and Technology, Langfang 065201, China.*

**Background:** From the perspective of psychological satisfaction, fintech can reduce the management costs of commercial banks, improve operational efficiency, meet customer psychological needs, and achieve psychological satisfaction, thereby enhancing their core competitiveness. The Internet can break through the limitations of time and space blockade, increase the customer range of commercial banks, improve their psychological satisfaction level, greatly reduce the service costs of commercial banks, and thus increase the psychological satisfaction and loyalty of commercial bank customers.

**Subjects and Methods:** This article studies the impact mechanism and empirical analysis of financial technology on customer psychological satisfaction from the perspective of customer psychological satisfaction, combined with the characteristics of commercial banks, from both theoretical and empirical perspectives. Based on this, this article uses the “text mining” method to construct the impact index of financial technology, using state-owned large commercial banks, joint-stock banks, and urban commercial banks as samples, and measures the customer psychological satisfaction index, empirically analyzing the impact mechanism of financial technology on customer psychological satisfaction.

**Results:** This article analyzes the impact mechanism of financial technology development on customer psychological satisfaction, and benchmark test results show that the development of financial technology significantly improves the level of customer psychological satisfaction. After a series of robustness tests, the main conclusions of this paper remain unchanged. Mechanism analysis found that the improvement of income level, the development of entrepreneurial activities, and the narrowing of urban-rural income gap are important reasons for the impact of financial technology development on customer psychological satisfaction.

**Conclusions:** Financial technology can improve customers’ psychological satisfaction, increase their happiness index, and achieve psychological satisfaction. According to empirical research results, due to historical and management system reasons, large state-owned commercial banks seem to have a slight lag in responding to external environmental changes, and their impact on customer psychological satisfaction is relatively low. However, equity banks and urban commercial banks are significantly weaker in responding to the impact of financial technology. Therefore, commercial banks should base themselves on their own strength, seize opportunities in the external environment, and enhance psychological satisfaction. At the same time, the government should support the development of fintech, promote the standardization of fintech in China, formulate policies and regulations related to fintech, promote the sustainable and healthy development of the financial industry, and thus improve customer psychological satisfaction and happiness index to a greater extent.

## LHMM23042 RESEARCH ON THE REFORM OF TEACHING MODE OF COMPUTER COMPOSITION PRINCIPLE IN LOCAL COLLEGES ON RELIEVING STUDENTS’ PSYCHOLOGICAL ANXIETY UNDER THE REQUIREMENT OF EMERGING ENGINEERING ERA

Shigan Yu^a^, Fujun Ren^a^, Xiaoling Ru^a^, Hui Guo^a^, Xiuyou Wang^a^

*^a^ Fuyang Normal University Fuyang, 236041, Anhui, China.*

**Background:** The era of emerging engineering has endowed college teachers with a new mission, and training high-quality professionals to meet the needs of emerging engineering has become a new goal. As the core course of computer-related majors, the traditional teaching mode of the course lags behind the requirements of the emerging engineering era, which leads to the lack of learning initiative, low academic performance, backward practical ability, and weak ideological and political consciousness of the students majoring in computer science, thus causing their concerns about their employability after graduation. It makes students worried about their future and causes serious psychological problems. Therefore, the teaching mode of this course needs to be reformed.

**Subjects and Methods:** This paper analyzes the problems in the traditional teaching process, studies the pain points in teaching, faces the strategic requirements of the new engineering era, takes training high-quality talents as the goal, takes students as the center, and makes students busy, teachers strong, management strict and the effect practical. This paper proposes PBL (Problem-based Learning) and “Preview, Bridge in, Discussion, Evaluation, Exercise, Designing” six-step teaching model, Online and offline hybrid teaching ideas, flipped classroom teaching methods, progressive curriculum ideological and political teaching process, experiential learning and Outcome-based Education (OBE) concept combined teaching system and curriculum innovation evaluation system.

**Results:** Through the investigation and statistical analysis of the teaching reform results, it can be found that the innovative teaching reform aiming at the principle of computer composition has enhanced students’ learning initiative, improved their academic performance, increased their practical ability, enhanced their ideological and political awareness, and cultivated a large number of patriotic and high-quality talents required by the new engineering era. It can reduce students’ worries about their future employability and alleviates students’ psychological problems. Conclusions: The new teaching method proposed in this paper has been proved to be efficient in practice, which can improve students’ comprehensive ability, make students full of confidence in the future and relieve psychological anxiety, and preliminarily meet the requirements of the emerging engineering era. The course presenter will continue to promote the reform of teaching mode according to the connotation of the requirements of the emerging engineering, and cultivate a larger team of high-quality professionals.

### Acknowledgments

The work was supported in part by Quality project of Anhui Province with Grant number 2018mooc061, 2020xsjxms182, 2020mooc386, 2020zdxsjg260, 2020jyxm1423, 2021sx117, 2021jyxm1121 and 2021jxtd215, in part by Talent research launch start-up project of Fuyang Normal University under grant 2019kyqd0018, in part by Anhui Province university key research project under grant KJ2018A0669 and 2022AH052820, in part by List of first-class undergraduate programs in Anhui Province under Grant 209 and in part by Ministry of Education industry-school cooperative education project under grant 220601363062820.

## LHMM23043 RESEARCH ON THE STRATEGY OF BUSINESS PROMOTION BASED ON THE SATISFACTION DEGREE OF PUBLIC MENTAL HEALTH: TAKING THE INTERNATIONAL GRAND BAZAAR AS AN EXAMPLE

Huan Wang^a^, Tao Fan^a,b^, Jian Wang^a^, Yan Shen^a^

*^a^ College of Architectural and Civil Engineering, Xinjiang University, Urumqi 830047, China, ^b^ College of Architecture and Urban Planning, Tongji University, Shanghai 200092, China.*

**Background:** Due to the impact of the Internet on traditional business, bazaars in Xinjiang, China, have developed slowly in recent years. In order to activate this traditional commercial form, the government has carried out a series of construction practices of large bazaars. Although the actual operation effect of many bazaars is ideal, the problem of many bazaars is that the degree of public mental health and happiness is not enough, so it is necessary to study the strategy of commercial promotion based on the degree of public mental health and happiness.

**Subjects and Methods:** The research object of this paper is the most famous commercial complex in Xinjiang -- Xinjiang International Grand Bazaar. Firstly, the objective analysis of customer behavior of Xinjiang International Grand Bazaar on the spot investigation of public mental health and happiness, including: walking traffic space status analysis, walking square space status analysis, function and landscape analysis. Secondly, 743 on-site and online questionnaires were used to conduct subjective analysis and evaluation on the status quo of public mental health and happiness in Xinjiang International Grand Bazaar.

**Results:** Based on the objective analysis and subjective evaluation of public mental health and happiness, it is concluded that Xinjiang International Grand Bazaar puts emphasis on the renovation and upgrading of public square public space, gives full play to the synergistic effect produced by the combination of public space and commercial space, and improves the public mental health and happiness. The space has a strong degree of openness and inclusiveness. However, there is still some room for improvement in the degree of public mental health and happiness: (1) The utilization rate of the square is not enough, the lack of public space to meet different mental health needs. (2) Single functional area to increase functional space to meet more mental health needs. (3) Accessibility is not ideal and happiness is reduced.

**Conclusions:** And finally put forward the promotion of mental health and happiness degree strategy: ⑴ rich square space to meet a variety of mental health needs, to create a comfortable and pleasant rest space, interactive space to promote mental health communication. (2) Expansion and reconstruction of functional zoning, reasonable distribution in each area of the International Bazaar; (3) establishment of body traffic, formation of “multiple first floor” mode, and use of air corridor to increase building connectivity. This paper studies the degree of public mental health and happiness in Bazaar, the design strategy proposed in this paper can further activate the vitality of the Bazaar, attract consumers to higher floors or underground Spaces, and contribute to a more benign interaction between the International Grand Bazaar and the city, so as to improve its actual operation effect. It provides theoretical reference for the reconstruction of Xinjiang International Grand Bazaar and similar building design in the future.

### Acknowledgements

This work was supported by the project grant from 2020 Xinjiang Natural Science Foundation Youth Fund “Research on the Design Strategy of Public Cultural Service Function Based on Synergy Theory: A Case Study of Xinjiang Commercial Complex” (Grand No. 2020D01C059).

## LHMM23044 ANALYSIS OF JOINT DISTRIBUTION MODE AND SERVICE PLATFORM ARCHITECTURE DESIGN FOR PHARMACEUTICAL COLD CHAIN LOGISTICS UNDER THE TARGET OF EQUALIZATION OF BASIC MEDICAL SERVICE

Yihua Li^a^, Mengjie Deng^a^, Hao Tian^a^, Wenjing Huang^b^

*^a^ School of Transportation & Logistics, Central South University of Forestry & Technology, Changsha 410004, Hunan, China, ^b^ School of Materials Science and Engineering, Central South University of Forestry & Technology, Changsha 410004, Hunan, China.*

**Background:** In order to effectively cope with urban public health crisis, improve the toughness of the public health system, improve the level of medical health, and ensure the safety of consumers’ medication. This study focuses on the most pressing difficulties and problems that are of great concern to the people in the field of medical and health. It aims to achieve low cost, high efficiency and high safety distribution of pharmaceutical products by conducting research on the joint distribution mode of pharmaceutical cold chain logistics.

**Subjects and Methods:** In this paper, we first compose the development of pharmaceutical cold chain logistics and its infrastructure construction in China in recent years. Secondly, we compare and analyze three mainstream distribution modes in pharmaceutical cold chain logistics industry. Then we summarize the social benefits of carrying out joint distribution of pharmaceutical cold chain logistics. Finally, we enumerate the technical advantages of blockchain.

**Results:** The results show that the development of pharmaceutical cold chain logistics has a bright prospect, and the implementation of joint distribution in pharmaceutical cold chain logistics can improve resource utilization, reduce business risks, help environmental friendliness and promote healthy development of the industry. The technical advantages of blockchain such as decentralization, distributed storage, tamper-evident, traceability and smart contract provide great convenience for the development of joint distribution business.

**Conclusions:** The blockchain-based information service platform for joint distribution of pharmaceutical cold chain logistics can play a role in reducing costs and increasing efficiency, promoting cooperation and facilitating supervision. In addition, the real-time reaches of logistics data can greatly reduce the medical safety incident caused by the quality of medical products deteriorate in transportation process. The establishment of this information service platform has enlightening significance for the information construction of the entire pharmaceutical logistics industry.

### Acknowledgements

Support for this study was jointly provided by General Project of Chinese National Social Science Foundation “Research on the Balanced Path of the Cold Chain Logistics Structure System of Agricultural Products in the Digital Village Background” (22BGL173), Major project of the Hunan Social Science Review Committee “Research on the Implementation Countermeasures for Promoting the Equalization of Basic Public Services in Hunan Province by Medical Cold Chain Logistics Driven by Data” (XSP22ZDA006) and General Project of Hunan Natural Science Foundation (2022JJ31015). The authors would also thank all the institutions and units for providing statistical data, information, and documents used in this study.

## LHMM23045 THE IMPACT OF LIVELIHOOD CAPITAL ON LIVELIHOOD RISK AND MENTAL HEALTH OF FARMERS OUT OF POVERTY IN POOR MOUNTAINOUS AREAS OF SOUTHWEST CHINA

Yi Xiao^a^, Ke Yin^b^, Yumeng Yang^c^

*^a^ College of Public Administration; Chongqing Technology and Business University; Chongqing 400067, China, ^b^ College of Geographical and Travel, Chongqing Normal University, Chongqing 400047, China, ^c^ College of Business Administration, Chongqing Technology and Business University, Chongqing 400067, China.*

**Background:** Poverty alleviation in 2020, farmers still face various types of livelihood risks. In the livelihood risk analysis, livelihood capital is the core of the analysis. Therefore, it is necessary and practical to study the impact of livelihood capital on the livelihood risk and mental health of farmers in poor mountainous areas in Southwest China, so as to provide empirical basis for the government to better formulate relevant policies and consolidate the results of poverty alleviation.

**Subjects and Methods:** Based on this, this study takes the questionnaire of 321 poverty-stricken farmers in the southwest poor mountainous areas as the sample data, analyze the impact of farmers’ livelihood capital on their livelihood risk and mental health.

**Results:** The results show: (1) Livelihood risks of poverty-stricken farmers include natural risks, economic risks, welfare risks and social risks. (2) Economic risk is affected by financial capital and human capital, natural risk is affected by natural capital and material capital, and welfare risk is affected by social capital, financial capital and material capital. Social risk is affected by natural capital and financial capital. It can be seen that human capital and financial capital are the key factors affecting the livelihood risk of farmers.

**Conclusions:** On the one hand, the local government should increase the investment in the human capital of rural households to promote the systematization, diversification and specialization of farmers’ employment training. Secondly, we should increase investment in education, improve the capital stock of farmers’ livelihood and guide their mental health development as a whole; On the other hand, the government should improve the land utilization rate and promote the marketization process of rural land transfer. At the same time, the government should increase its support for farmers’ cooperative organizations, accelerate the construction of a modern rural financial system, develop a diversified rural credit support model, and increase the financial capital stock of farmers, so as to effectively improve the livelihood level of farmers.

### Acknowledgments

This study was funded by National Social Science General Project: Study on Livelihood Risk and Sustainable Guarantee of Poverty Alleviation Farmers in Poor Mountainous Areas (20BJY166).

## LHMM23046 RESEARCH ON TEACHING METHODS OF MULTI INFORMATION FUSION IN HIGHER VOCATIONAL COLLEGES BASED ON STUDENTS’ MENTAL HEALTH

Xiaoke Yin^a^, Yi Yu^a^, Zhiwei Tang^a^, Bo Hu^b^, Dr. Ali Abid^c^

*^a^ Hunan Biological And Electromechanical Polytechnic, Changsha, 410127, China, ^b^ Beiya Middle School of Changsha, Changsha, 410005, China, ^c^ Allied Hospital, Faisalabad, Punjab 54590, Pakistan.*

**Objective:** According to the latest statistics of the World Health Organization, mental health problems are one of the main causes of extreme events (such as suicide). Therefore, the psychological health of college students is an extremely important research topic. The research on college students’ mental health is traditionally based on small-scale questionnaires, scales or experimental data. This paper creatively proposes a diversified information based teaching method that combines online open courses, intelligent teaching platforms and virtual simulation teaching resources, and uses big data technology and concepts to analyze a large range of samples.

**Methods:** The online open courses and virtual simulation teaching resources were organically integrated to solve the problem that students were difficult to understand the real learning situation; Integrate virtual simulation teaching resources and intelligent teaching platform organically to solve the problem that teachers cannot fully understand students’ learning effects; The smart teaching platform and online open courses will be organically integrated to solve the problem that managers are difficult to understand the teaching situation. From the aspects of university economic life, academic performance, comprehensive performance, mental health and school satisfaction, the development of special enrollment plan students in rural and poor areas will be discussed.

**Results:** The online open courses, intelligent teaching platform and virtual simulation teaching resources were integrated organically and promoted as a whole, and teaching methods were innovated, teaching resources were enriched and teaching effectiveness was improved. A machine learning model has been established to predict postpartum depression and depression through social media data, and the accuracy of the latter can reach 70%. Research based on microblog text and Instagram photos has also proved that machine learning methods can be used to identify patients with early depression with high accuracy. Literature systematically analyzed Sina Weibo users based on multi task regression and incremental regression algorithms and used them to predict the five personality traits.

**Conclusion:** It can not only make up for the shortcomings of traditional classroom in colleges and universities, fully mobilize students’ enthusiasm for learning, but also evaluate students’ learning effects and timely adjust teaching strategies, so as to promote the comprehensive application of information and intelligence in education. Inspired by the recent education big data and computational social economics methodology, this paper intends to explore the relationship between students’ mental health problems, especially depression symptoms and students’ social behavior by analyzing students’ behavior data under uncontrolled conditions.

### Acknowledgments

Obtained the “Fourteenth Five Year Plan” of Hunan Education Planning Science in 2022, and “Research on the Path of Multi information Integration Teaching Means in Higher Vocational Colleges” (Project No.: XJK22CGD076).

## LHMM23047 THE THOUGHT OF ECOLOGICAL CIVILIZATION LEADS THE WAY TO REALIZE THE LEGALIZATION OF THE NATIONAL MENTAL HEALTH

Deng Xin^a^

*^a^ Department of Law, School of Political Science and Law, Xinjiang Normal University, Guanjing Road No. 100, Shuimogou District, Urumqi, Xinjiang, China.*

**Background:** With the fierce competition in modern society, various psychological problems have become important factors that seriously affect people’s health and life satisfaction. The National Health Conference held in 2016 and the Outline of the Healthy China 2030 Plan issued after the conference raised the issue of universal health to a national strategic level. The Guidelines on Implementing the Healthy China Action, issued on July 15, 2019, clearly states that mental health promotion actions should be carried out. Thus, it can be seen that guaranteeing the mental health of the whole people plays an important role in the strategy of “Healthy China”. The formation of the national mental health system requires the construction of the top-level mental health coordination mechanism under the guidance of the idea of ecological civilization, and the system innovation of realizing the national mental health from the perspective of legal protection. To build an ecological mental health model for the whole people, this paper discusses the role of ecological civilization thought as an important ideological guidance as well as its application in realizing the legalization of promoting the national mental health.

**Subjects and Methods:** To this end, this paper combs out the internal correlation between the thought of ecological civilization and the legal system of national mental health from the aspects of theoretical basis, logical consistency and historical experience, and analyzes the internal mechanism of realizing the legalization of the national mental health from the aspects of ecological jurisprudence foundation and ecological psychological system construction. From the perspective of legalization, this paper discusses the value, implication and practical path of how to raise the mental health problem of the whole people to the level of legal rights under the guidance of ecological civilization.

**Results:** Based on the above analysis, this paper proposes to construct the legal environment through inheriting the excellent ecological mental health culture and creating the legal atmosphere of ecological mental health; unify the individual value orientation and common consciousness orientation by constructing a mental health ecological legal education system formed by theoretical cognition and practice; and leads the construction of the mental health awareness system of the whole people by optimizing the mental health rule of law under the guidance of ecological civilization.

**Conclusions:** To realize the legal path of the right to mental health of the whole people, it is necessary to establish a right protection system on the basis of clarifying the subject of rights and the relative subject of obligations, and clarifying the content of rights and obligations between subjects. By integrating ecological civilization thought and practice into the process of exploring the legalization of promoting the national mental health, it is possible to promote all nationalities to actively construct the legal coordination mechanism and the relief assistance mechanism of mental health, providing a reference for the theoretical research and practice on promoting the national mental health.

### Acknowledgements

This work was supported by the project of “Research and Reform of Undergraduate Education and Teaching in Colleges and Universities” by the Department of Education of Xinjiang Autonomous Region in 2022, “In-depth exploration and Organic integration of ideological and political Elements in the Course of Environmental and Resource Protection Law”. (Grant No. XJGXPTJG-202227).

## LHMM23048 EXPERIMENTAL STUDY ON THE EFFECT OF DIFFERENT PHYSICAL EXERCISE PROGRAMS ON PSYCHOLOGICAL ANXIETY AND DEPRESSION IN COLLEGE STUDENTS

Yuhang Wang^a^, Hua Zhou^b^, Yingli Li^b^, Mengqi Wang^b^

*^a^ Institute of physical culture, Harbin University, Harbin, Heilongjiang 150076, China, ^b^ School of Marxism Northeast Agricultural University, Harbin, Heilongjiang 150030, China.*

**Background:** As we all know, physical exercise helps to enhance physical fitness and promote physical health. However, the effects of different physical exercise programs on psychological anxiety and depression have not attracted sufficient attention from scholars. This paper adopts the method of exercise intervention to study the impact of different physical exercise programs on anxiety and depression symptoms in college students, providing a theoretical reference for solving the mental health problems of college students, and providing a scientific basis for the formulation of exercise prescription.

**Subjects and Methods:** Using the whole group sampling method, 60 undergraduates of Northeast Agricultural University with anxiety and depression symptom were selected as subjects. The subjects were divided into basketball group, badminton group and aerobics group according to their interest. The three groups of subjects were given 16 weeks of physical exercise intervention training. And Self-rating Anxiety Scale (SAS), Self-rating Depression Scale (SDS) were used for assessment before and after the intervention. SPSS 20.0 statistical software was used for statistical analysis of the obtained data. The data before and after the same exercise program was compared by independent sample t test, and the data before and after the experiment between different exercise programs was compared by single factor ANOVA.

**Results:** The anxiety level of the basketball group, the anxiety and depression level of the badminton group and the depression level of the aerobics group were significantly different before and after the experiment (t = 3.81, 1.36, 2.86, 2.54 respectively, p < 0.05). The anxiety level of the aerobics group was extremely significant, and the depression of the basketball group was extremely significant (t = 5.76, 6.82 respectively, p <0.01). After the intervention, it was also found that SAS scores between basketball group and aerobics group, basketball group and badminton group, badminton group and aerobics group were all statistically significant (t = 5.32, 3.25, 3.88 respectively, p < 005) and SDS scores between basketball group and aerobics group, basketball group and badminton group, badminton group and aerobics group are all statistically significant as well (t = 5.87, 3.22, 2.69 respectively, p < 005).

**Conclusions:** The three kinds of physical exercise program can all significantly alleviate the anxiety and depression symptoms of college students, among them, aerobics program has the most significant effects on anxiety symptoms of college students followed by badminton program and basketball program. And badminton program has the most obvious effect on reducing the symptoms of depression followed by badminton program and aerobics program. This study only considered the effects of physical exercise programs on anxiety and depression of college students. It did not put the mediating variables such as gender factor, physical quality, personal characteristics into consideration, which should be considered in the follow-up study.

### Acknowledgements

Supported by a project grant from GJB1423410; GJE1422083.

## LHMM23049 RESEARCH ON THE PATH OF CARBON REDUCTION AND SEQUESTRATION IN AGRICULTURE AND RURAL AREAS UNDER THE PSYCHOLOGICAL IDENTITY OF LOW CARBON AND ENVIRONMENTAL ANXIETY

Jiangxin He^a^, Yi Wang^a^

*^a^ School of Marxism, Xi’an University of Science and Technology, Xi’an 710600, China.*

**Background:** The public recognition of low carbon psychology and anxiety about the environment are not only the objective requirement of people’s response to climate change, but also the realistic need of the transformation and optimization of agricultural industry. Although low carbon life is a new concept, it does raise the world sustainable development of the old problem, it reflects the human because of climate change and the future environment concerns and anxiety. With agricultural modernizing, a great demand for carbon emission reduction and sequestration in agriculture and rural areas.

**Subjects and Methods:** By identifying and confirming the basis and main types of carbon sources and sinks, as well as searching literature and conducting research, we found in-depth understanding of the existing problems of carbon emission in the Yangtze River Economic Belt, and ensure that the path of carbon emission reduction and sequestration is reasonable and effective. In this way, it can enhance people’s recognition of low carbon psychology and relieve people’s anxiety caused by environmental change.

**Results:** Through studying the path of carbon emission reduction and sequestration in agriculture and rural areas under the condition of low carbon psychological identity and environmental anxiety, some problems existed in the Yangtze River Economic Belt region. For example, in the implementation of carbon reduction and sequestration goals, first of all, we should adhere to the green development of agriculture at the same time; Secondly, we should ensure the safety of agricultural products. While ensuring food production, we should also pay attention to the safety of agricultural products, which is related to people’s health. Finally, we should optimize and innovate the system and mechanism.

**Conclusions:** In order to promote the smooth implementation of carbon reduction and sequestration in agriculture and rural areas under the condition of low carbon psychological identity and environmental anxiety, technological innovation can be promoted and utilized. Developing conservation-oriented agriculture; Optimize agricultural management; Enhancing public awareness and changing lifestyles; Afforestation and other ways to solve the problem of carbon emissions polluting the environment, through these effective and reasonable paths, further improve the environmental state that people rely on for survival, can improve the public’s psychological identification of low-carbon lifestyle, but also can alleviate people’s anxiety about carbon emissions polluting the environment.

## LHMM23050 ANALYZING THE MECHANISM OF THE IMPACT OF DIGITAL ECONOMY ON INCLUSIVE GREEN GROWTH BASED ON THE PERSPECTIVE OF EMOTION REGULATION

Songtao Wang^a^, Yuncai Ning^a^

*^a^ School of Management, China University of Mining & Technology, Beijing, 100083, China.*

**Background:** Inclusive green growth is an important symbol of high-quality economic growth, and emotional regulation is a representative of social equity. The purpose of this article is to explore the impact of the digital economy on inclusive green growth, including emotional regulation, and analyze the important role of emotional regulation in inclusive green growth.

**Subjects and Methods:** From the perspective of emotional regulation, a system dynamics model including emotional regulation was constructed to study the impact of the digital economy on emotional regulation. The system dynamics model includes stocks, variables, constants, etc.

**Results:** The system dynamics model simulation results show that the digital economy can form positive feedback on emotion regulation, and emotion regulation plays a positive role in inclusive green growth. It can be seen from this that emotion regulation can reduce the income gap and promote inclusive green growth. The impact mechanism of the digital economy on inclusive green growth is that it can positively regulate emotions, thereby reducing the wealth gap and promoting inclusive green growth.

**Conclusions:** This article studies the impact mechanism and system operation rules of the digital economy on inclusive green growth from the perspective of emotional regulation, which can provide theoretical support for relevant policy recommendations in the later stage. Therefore, the following policy recommendations are proposed: promoting the development of the digital economy is conducive to public emotional regulation, thereby reducing the wealth gap and improving mental health. Emphasizing the positive mechanism of the digital economy on emotional regulation is beneficial for inclusive green growth and strengthening the application of the digital economy. And utilize the strong vitality of the digital economy to promote inclusive green growth in cities, thereby promoting economic growth, alleviating national psychological pressure, and improving mental health. In the process of developing the digital economy, we should focus on increasing investment in the information industry, encouraging the development of new startups, and strengthening the application of the digital economy to strengthen it.

## LHMM23051 DEVELOPMENT CHARACTERISTICS OF FUNDAMENTAL MOTOR SKILLS OF CHILDREN AGED 3-6 IN ANHUI PROVINCE PROVINCE

Fusheng Liang^a,b^, Jianmei Xu^a^, Xingying Li^b^

*^a^ Huangshan University, Huangshan, 245041 China, ^b^ East China Normal University, Shanghai, 200241, China.*

**Background:** This paper discusses the developmental characteristics of fundamental motor skills of children aged 3-6 in Anhui Province, providing a basis for the creation and implementation of physical activities and games for young children, a reference for the improvement of children’s physical fitness, as well as for the integration of sports, education, and medicine.

**Subjects and Methods:** A total of 240 young children aged 3–6 in Anhui Province were selected as the survey subjects for statistical analysis by using the measurement tool of TGMD-3.

**Results:** The average total scoring rate of fundamental motor skills for 3-6 year-old children in Anhui Province is 48.7%. Specifically, the average total scoring rate for fundamental motor skills is 30.1% for 3-4 year-olds, 47.85% for 4-5 year-olds, and 67.58% for 5-6 year-olds. There is a significant difference (P<0.05) in the Horizontal jump (S5) between boys and girls aged 3-4 years and 5-6 years, but there is no significant difference (P>0.05) in all other items for all age groups. The three items of Run (S1), Skip (S4), and Underhand throw (S13) all show significant differences (P<0.05), while the other items and total scores do not show significant differences (P>0.05). There is a significant difference (P<0.05) between the underweight and overweight groups for 3-4 year-olds and the overweight and normal groups for 4-5 year-olds. There is a highly significant difference (P<0.01) in the obese group for 4-5 year-olds. The multiple correlation coefficients of the comprehensive influencing factors for each age group are R=0.820, R=0.688, and R=0.866, and the significance is P<0.05 for all.

**Conclusions:** Boys between the ages of 3-6 perform better in both locomotor skills and manipulative skills compared to girls of the same age group. There are significant differences in the development of fundamental motor skills between adjacent age groups of 3-6 year-olds, and the scoring rate increases are more pronounced in the 4-6-year-old group, with several individual items showing a greater increase. There are some urban-rural differences in the development of fundamental motor skills in 3-6 year-old children, and BMI has a certain impact on their fundamental motor skills. The family’s exercise atmosphere, crawling experience in infancy, and participation in sports interest classes are positively correlated with children’s fundamental motor skills, with the main effect of the family’s exercise atmosphere being the most significant.

### Acknowledgments

This research was supported by the Young Backbone Teachers’ Domestic Visiting Program of Anhui Province Provincial Education Department (gxgnfx2019036); the College Students’ Innovation and Entrepreneurship Program in Anhui Province Province (S202010024); the Key Research Project in Humanities and Social Sciences in Higher Education Institutions in Anhui Province Province (SK2021A0632); and the Teaching and Research Project of Huangshan University (2021JXYJ12).

## LHMM23052 EXPLORING THE COUNTERMEASURES OF FOREIGN TRADE DEVELOPMENT IN CHONGQING FROM THE PERSPECTIVE OF INVESTORS’ PSYCHOLOGICAL ANXIETY

Zhaochu Qian^a^, Dan Wang^a^, Qiurong Wang^a^

*^a^ Chongqing Business Vocational College, Shapingba, Chongqing, China.*

**Objective:** Investors’ confidence in the market largely affects the trend of foreign trade enterprises and even foreign trade markets. Based on the perspective of investors’ psychological anxiety, this research analyzes the development status and practical constraints of Chongqing’s foreign trade, and puts forward countermeasures to achieve high-quality development of Chongqing’s foreign trade, so as to comprehensively promote the high-quality development of foreign trade in Chongqing

**Methods:** Based on the change of investors’ psychology, this research makes a multi-dimensional exploration of development in Chongqing from the perspective of investors’ psychological anxiety by means of market subject analysis, quantitative analysis and field research.

**Results:** The overall momentum of foreign trade development in Chongqing is good, but there are still some unfavorable factors. In terms of the external environment, the high inflationary pressures around the world is sustaining; the European economy is suffering a double blow; the impact of the mutation of the novel corona virus is continuing; the Sino-US economic and trade frictions is constant and presenting a long-lasting trend; the foreign trade enterprises are suffering survival pressure; and the investors’ psychological anxiety is obvious. In terms of the internal constraints, the survival pressure of foreign trade enterprises is increasing; the structure of foreign trade is still unbalanced; the new drivers of foreign trade are not yet mature; and the ability of investors to withstand pressure is limited.

**Conclusion:** After clarifying the causes of investors’ anxiety, this research combines the law of foreign trade development with the law of investors’ psychology change according to the actual development of foreign trade in Chongqing, and puts forward that the healthy development of foreign trade in Chongqing needs to work together from four aspects: strengthening the rescue of foreign trade enterprises, ensuring the stability and smooth of foreign trade industrial chain and supply chain, expanding foreign trade diversification and developing new business forms of foreign trade.

### Acknowledgments

This research has received the kind care, guidance and help from Chongqing Municipal Commission of Commerce and relevant enterprises in Chongqing. The systematic, forward-looking and practical content of this research has been significantly improved.

## LHMM23053 INFLUENCING FACTORS OF EFFECTIVENESS ON RELIEVING STUDENTS’ PSYCHOLOGICAL ANXIETY UNDER THE WEB-BASED COLLEGE ENGLISH TEACHING MANAGEMENT IN MIANYANG CITY

Zhang Xia^a^

*^a^ School of Foreign Language Mianyang Teachers’ College Mianyang, Sichuan, China.*

**Background:** This study is to investigate internal and external factors that influence on the effectiveness of relieving students’ psychological anxiety under the web-based college English teaching management.

**Subjects and Methods:** The author mainly used quantitative study methodology. Data was collected from 445 students of eight colleges/universities in Mianyang city. Also introduced the phenomenological study by in-depth interview technique to support the quantitative study. The key informants are six college teachers, two leaders and twelve students from eight colleges/universities in Mianyang city. The study instruments were questionnaires and in-depth interviews. The statistics in analyzing the data were percentage, mean, standard deviation, one-way ANONA, T-test, correlation analysis, and multiple regression analysis.

**Results:** The results showed that the internal factors have much more influence than external factors on the effectiveness of relieving students’ psychological anxiety under the web-based college English teaching management.

**Conclusions:** Finally, it concludeed the special management would be like this: “web-based teaching in class and web-based autonomous learning after class”: that is to take full advantages of internet and build a good platform for students’ self-learning, personalized learning and collaborative learning, so as to improve effectiveness on relieving students’ psychological anxiety under web-based college English teaching management.

## LHMM23054 RESEARCH ON INFLUENCING FACTORS OF ENTERPRISE SAFETY CULTURE IN HIGH-RISK INDUSTRY BASED ON THE PERSPECTIVE OF RELIEVING EMPLOYEES’ PSYCHOLOGICAL ANXIETY

Renlin Guo^a^

*^a^ Beijing Enterprise Safety and Environment Information Science Research Institute, Beijing 100048, China.*

**Background:** Psychological anxiety is an important factor that causes safety risks for employees. In order to evaluate the impact of psychological anxiety on safety culture, this article constructs an SEM model to study the quantitative impact of psychological anxiety on safety culture in high-risk industries.

**Subjects and Methods:** Psychological anxiety is influenced by multiple factors. As an important method to measure the impact of one group of multiple factors on another group of multiple factors, this article uses structural equation modeling to study the impact of psychological anxiety on safety culture in high-risk industries based on the obvious factors of psychological anxiety and safety culture.

**Results:** The conclusion indicates that psychological anxiety is an important factor affecting the level of safety culture in high-risk industries, and there is a negative correlation between the two. That is, the higher the psychological anxiety of employees in high-risk industries, the lower the level of enterprise safety culture. Therefore, enterprises in high-risk industries should strengthen the level of enterprise safety investment, try their best to eliminate employee psychological anxiety, thereby improving the level of safety culture, and adopt various methods to alleviate employee anxiety, Enable employees to work in a warm atmosphere, thereby creating a good level of safety culture in the enterprise.

**Conclusions:** The conclusion indicates that psychological anxiety is an important factor affecting the safety culture level of high-risk industry enterprises. Therefore, high-risk industry enterprises should eliminate employee psychological anxiety and improve safety culture level in the following aspects: firstly, strengthen safety investment, cultivate a good corporate safety culture, and minimize employee psychological anxiety; Secondly, carry out various safety activities to reduce employees’ psychological anxiety and create a warm working atmosphere; The third is to correctly view psychological anxiety and safety management level, and organically link the two together.

### Acknowledgments

This research was supported by the Langfang science and technology research and development project (project number: 2021029083).

## LHMM23055 STUDY ON THE INFLUENCE OF ONLINE TRUST ON INVESTMENT DECISION PROPENSITY OF ENTREPRENEURIAL PROJECTS AND INVESTORS’ PSYCHOLOGICAL HEALTH

Kun Zhao^a,b^, Rui Sun^c^, Yuan Yuan^c^

*^a^ Tan Siu Lin Business School, Quanzhou Normal University, Quanzhou 362021, China ^b^ National Academy of Econnomic Strategy, Chinese Academy of Social Sciences, Beijing 100028, China ^c^ School of Business Administration of Huaqiao University, Quanzhou 362021, China.*

**Background:** The article analyzes the impact of online trust of Chinese investment company TMT on investment decision propensity of entrepreneurial projects and investors’ psychological health.

**Subjects and Methods:** Firstly, the article defines the “trust” in the individual behavior attribute as “situation trust” and “quality trust” as the conceptual basis, and defines the trust relationship between the investment enterprise TMT and the entrepreneur. Second, by conducting an inductive longitudinal cross-case analysis on three Internet startup platforms in China, this study summarizes the relationships of the acquisition of situational and quality trust by investment company TMTs with the investment decision-making for entrepreneurial projects made by these TMTs. Thirdly, a research proposition is then proposed for further verification, from which the influence mechanism of investors’ psychological health under the condition of interacting situational trust and quality trust of TMTs are identified.

**Results:** The article finds that TMT online trust have a positive impact on TMT investment decision-making and investors’ psychological health. Online situational trust influences investment decision propensity through the heterogeneity of situational perception, while when TMT shows higher quality trust, it helps investors to take joint action on market, information, and cooperation, as well as to conduct experimental learning to generate investment value, and to improve online investment decision propensity and reduce psychological anxiety. The existence of transactional or value-based trust transfer between online situational trust and quality trust determines different types of relationships between the two trusting subjects and induces TMT to choose the investment strategy corresponding to them.

**Conclusions:** The article has important implications for further deepening the research on TMT online decision propensity of entrepreneurial projects and investors’ psychological health. First, the article fills a gap in the literature regarding the “internal mechanisms” and “trust transfer” of online trust processes in explaining investment decision propensity and investor mental health. Second, by revealing the mechanism of online trust transfer between TMT situational trust and quality trust based on transactional and value-based, the article provides new ideas for in-depth interpretation of investors’ online investment decisions and reduction of psychological anxiety. Thirdly, TMT should fully consider its own trust type and trust level, carefully design online trust investment mechanism, and choose the investment psychological strategy that fits with the trust type and trust level. Consistent with the problems of established inductive case studies, the findings of this study have limited external generalizability, and future research could expand the sample through other methods to improve external validity.

### Acknowledgments

Supported by a project grant from “Cognitive neural mechanism of personalized recommendation users’ privacy response” (Grand No.21FGLB041) and “Research on the formation mechanism and evolution path of intrepreneurial capability of manufacturing enterprises in Fujian driven by digital innovation” (Grand No.FJ2022B055).

## LHMM23056 MODEL COMPARISON OF EPIDEMIC PREVENTION AND CONTROL IN RURAL AREAS IN CHINA AND THEIR IMPACT ON PEOPLE’S MENTAL HEALTH

Dongqiang Wang^a,b^, Shuqin Tian^b^

*^a^ School of Management Science and Real Estate, Chongqing University, Chongqing, 400045, China, ^b^ Chongqing University of Arts and Sciences, Chongqing, 402160, China.*

**Background:** At present, the rural areas become the leading position, key area and weak link of epidemic prevention and control along with people’s mental health in China.

**Subjects and Methods:** This study trys to construct modern governance system for epidemic prevention and control in rural areas and analyse their impact on people’s mental health from the perspective of all aspects, the whole process and all elements. A total of four cases were collected and analyzed according to the database involved epidemic prevention and control in rural areas in China and their impact on people’s mental health built by the text analysis, grounded theory and multi-case analysis.

**Results:** The results show that four typical models of epidemic prevention and control in rural areas in China and their impact on people’s mental health, including legal leading model, local mobilization model, general grid model, and technical service model, are summarized and and a multi-case comparative study is conducted from the aspects of governance orientation, governance way, and governance mechanism. No matter which mode, COVID-19 had an impact on people’s mental health, mainly manifested as depression, anxiety, stress, loneliness, helplessness.

**Conclusions:** From the perspective of holistic management, this paper puts forward some policy suggestions to further strengthen the work of epidemic prevention and control in rural areas and reduce their impact on people’s mental health: functional integration to establish a centralized and efficient leadership and command system; benign interaction to improve the communication and coordination mechanism of effective connection between different departments; demand orientation to take a holistic management and diversification model according to local conditions; crossing borders to build and share the work digital information platform of epidemic prevention and control in rural areas.

### Acknowledgments

This paper was supported by the National Social Science Foundation Project “Research on the Effect Measurement and Path Optimization of Social Forces Participating in Rural Revitalization in Key Assistance Counties” (No. 22ASH014).

## LHMM23057 RESEARCH ON THE ADOPTION OF NEW TECHNOLOGIES FOR TELECOMMUNICATIONS OPERATORS BASED ON PSYCHOLOGICAL STABILITY

Yajun Gu^a^, Can Chen^b^, Yusheng Long^c^

*^a^ Tongji Zhejiang College, Jiaxing 314051, Zhejiang Province, China, ^b^ Jiaxing Nanhu University, Jiaxing 314001, Zhejiang Province, China, ^c^ Beijing University ShenZhen Hospital, ShenZhen 518000, Guangdong Province, China.*

**Background:** Investment decisions should not only consider the investment-return itself, but also analyze the psychological state of the decision-making subject, especially the psychological stability of the decision-making entity. This is a destabilizing factor that is independent of technological drivers. An individual’s psychological stability is subject to biological factors such as disease, psychological factors such as personality traits, sociological factors such as the family environment of origin, and other factors. The result of the decision-making entity’s processing of the above information ultimately becomes the key to the investment decision.

**Subjects and Methods:** 1. The factor analysis method and the comprehensive analysis method are used to obtain the main contents affecting the psychological stability of the decision-making subject. 2. The investment decision-making model is constructed. Based on the assumption that the state of technological progress and the efficiency of new technology adoption are uncertain, the Bellman equation for optimal investment is obtained, and the optimal investment time of the project operator is calculated. 3. Parameter analysis of the investment decision-making model. 4. Using association analysis, relying on the psychological stability analysis of decision-making subjects, the decision-making results under different preferences are obtained according to the risk preferences of decision-making subjects.

**Results:** 1. Psychological stability is subject to a variety of factors, and has strong instability and randomness. 2. The speed of technological progress is positively correlated with the uncertainty of positive benefits brought about by the adoption of new technologies, the positive rate of change in the adoption efficiency of new technologies, and the opportunity value F(V) and investment threshold V* adopted by telecommunications operators, while the negative rate of change in the adoption efficiency of new technologies is negatively correlated with the opportunity value F(V) and investment threshold V* adopted by new technologies adopted by telecommunications operators.

**Conclusions:** In the field of telecommunications, operators make decisions on investment in new technologies, which is not only determined by technology itself, but also by the psychological stability of decision-making subjects is more decisive for the implementation of decision-making behavior. In fact, the psychological stability or broad sense of decision-making subjects is mental health the key to the success or failure of decision-making. In order to avoid the uncontrollability of individual decisions, team decisions can more rationally complete new telecommunications investment decisions. Broadly, similar methods can be used to make other types of decisions.

## LHMM23058 RESEARCH ON LINGUISTIC BEHAVIOR AND MENTAL HEALTH FROM THE PERSPECTIVE OF ARTIFICIAL INTELLIGENCE

Hao Peng^a^

*^a^ School of Foreign Languages, Wuhan Polytechnic University, Wuhan 430024, China.*

**Background:** Language is a tool for human beings to convey their feelings and ideas. Human beings use language not only to convey information, but also to express their inner feelings. Therefore, language is also a window into people’s mental health. Through verbal communication, psychiatrists can diagnose patients with mental disorders; Using big language data, scientists can quantify the special experience of doctors and develop intelligent products for early diagnosis of mental disorders. Linguistic researchers not only need to reveal the information of mental health through language, but also need to explore the neural mechanisms related to it.

**Subjects and Methods:** AI studies how to use computer to simulate the ability of human brain to recognize, understand, participate, reason, learn, think and solve problems, and AI can perceive the external environment, further think, learn, and output corresponding behavior. AI has been described as the use of algorithms and software to approximate human cognition when resolving complex data. All AI involved in this paper belongs to the field of computer science and will adopt a series of technologies and methods: machine learning, natural language processing, speech processing, robotics, and similar automated decision making, etc., for developing and implementing human-specific language cognitive processes and mental health monitoring therapies.

**Results:** Language production involves brain networks that process auditory, somatosensory, and visual input, as well as language perception and production. Mental illness often results in abnormalities in these brain networks, so analyzing language patterns can help diagnose mental disorders. Deficiencies in verbal communication are evident in many severe mental disorders, particularly those involving psychomotor retardation, affective retardation, and poverty of speech content. Language is an effective method to detect the mental state of patients. Existing research using artificial intelligence techniques to analyze voice and language patterns has been highly accurate in screening for mental health problems. Some researchers have developed algorithms into artificial intelligence systems that evaluate speech samples and compare them with previous samples of the same patient to score their mental status. Research has found that AI model scoring can be as accurate as human clinicians.

**Conclusions:** Thanks to the rapid development of machine learning, the accuracy of predicting mental disorders by analysing linguistic data is also increasing. Language is a window through which experienced psychiatrists can assess a patient’s mental state. If artificial intelligence can quantify the doctor’s special experience, it will greatly improve the objectivity and popularity of diagnosis. In addition to the flexibility and universality of the collection, language features as a tool for early screening of mental disorders have unique advantages. In addition, social platform language big data can also be used for monitoring users’ psychological status, and the analysis results can provide objective basis for government departments’ decision-making in mental health. Similarly, rapid advances in neuroscience and biological science have provided scientific tools for exploring the relationship between explicit language and Behavior and internal psychology and spirit. Although artificial intelligence and neuroscience have made initial achievements in exploring brain mechanisms and identifying mental disorders using language information, they are still far from practical clinical application. On the way of monitoring and protecting people’s mental health through the window of language data, multidisciplinary linguistic research still has a long way to go.

## LHMM23059 EXPLORATION OF IDEOLOGICAL AND POLITICAL TEACHING AND STUDENTS’ MENTAL HEALTH MAINTENANCE IN HIGHER MATHEMATICS COURSES UNDER THE BACKGROUND OF NEW MEDICAL SCIENCE

Yuezi Wei^a^, Wen Shi^a^, Hongbo Fu^a^

*^a^ School of Biomedical Engineering, Guangzhou Medical University, Guangzhou, Guangdong, 511436, China.*

**Background:** For the higher mathematics curriculum, it is still a new subject to carry out curriculum ideological and political reform and integrate ideological and political elements into curriculum teaching. Higher emotional intelligence is conducive to the formation of sound personalities. Combining ideological and political education with curriculum reform and teaching practice has an important means and method to cultivate people’s personality.

**Subjects and Methods:** Starting from affairs around us and national events, we internalized ideological and political education into the classroom teaching of higher mathematics, and practiced and explored the actual links. Through the teaching of ideological and political cases with the characteristics of the epoch and infectious, it opens a channel for students to connect mathematics with the outside world, and establishes the sound mentality of students.

**Results:** In this way, students’ enthusiasm and initiative in learning mathematics are improved, knowledge updating and cross-integration are realized, and students get the ability to find and solve problems. Through ideological and political education, psychological education cultivates students’ high-quality emotional intelligence and shapes sound personalities, so that college students can form a healthy psychology.

**Conclusions:** Teaching with ideological and political materials can promote the establishment and perfection of students’ mentality. By integrating ideological and political education with the study of mathematics, it not only educates students in ideology and morality, but also allows students to understand the method of mathematical modeling from the mathematical model, and understands the practicality of mathematics. We will train professionals who have a healthy personality, healthy psychology, ideal and faith, morality, correct value pursuit, ability and responsibility of The Times, and promote the construction of new engineering and new medical sciences.

### Acknowledgments

This work was supported by the Second Batch of Industry-University Collaborative Education Project of the Ministry of Education in 2019 (Project No. 201902120032); the Education Science Planning Project of Guangzhou in 2020 (Project No. 06-410-2113029); and the First-class Major Construction Project of Guangzhou Medical University (Project No.: 02-408-2304-11-010XM).

## LHMM23060 THEORY AND PRACTICE OF ONLINE EXAMINATION OF IDEOLOGICAL AND POLITICAL COURSESIN COLLEGES AND UNIVERSITIES: BASED ON STUDENTS’ ANXIETY

Fengxiang Deng^a^

*^a^ School of Marxism, Hunan City University, Yiyang, Hunan, 413000, China.*

**Background:** During the COVID-19, many universities used remote online teaching methods, and online examination became a research topic. College students have two distinct psychological attitudes towards courses during the pandemic. The first type is a relaxed mentality, believing that loose and remote exams will give them great opportunities, so there is no need to study hard. The second type is anxiety, where some students are worried that the exam format may not be suitable or that they may find it difficult to achieve good grades. Some students may believe that remote exams can lead to inconsistent exam situations for different students, thereby harming the fairness and justice of exams.

**Subjects and Methods:** The first is the feasibility of online exams. The progress of technology and society has put forward new requirements for exams, and the popularization of computers and mobile phones has made online exams possible. The emergence of the epidemic and natural disasters has made online exams necessary. Online exams are not only an improvement in assessment methods, but also a beneficial practice of open and personalized assessment, which meets the basic requirements of course teaching. The second is how effective online exams can be in eliminating exam anxiety among college students. There are three research methods. The first is literature research. We have collected various international achievements on the psychological anxiety caused by online exams and solutions, and screened the correctness and universality of these views in order to obtain the best solution based on our research subjects. The second is to conduct empirical research through online exams, which includes three parts: pre exam preparation, exam implementation, and post exam summary and improvement. The third is a survey of candidate satisfaction.

**Results:** In theory, our colleague Lin Xiaojin et al.‘s research has proven that online exams are feasible. Practitioner Yue Sun also believes that universities should give more consideration to the selection and construction of online platforms, and carefully arrange online teaching. In practice, we have conducted ten years of repeated practice and compared the results of the offline exam in 2018 with the online exam in 2020, demonstrating that the results of the online and offline exams are equivalent. Through public opinion surveys, it is believed that online exams are better than offline exams.

**Conclusions:** Online and offline exams have equivalence, and are superior in terms of convenience and fairness compared to offline exams. College students are very satisfied with online exams, with 66.23% of students highly recognizing computer-based exams, 24.12% of students believing that computer-based exams can be used, and only 9.65% of students still cling to traditional offline exams. This indicates that online exams during the epidemic period based on student anxiety resolution are successful.

### Acknowledgments

This work has been supported by the Education Department of Hunan Province, China (approval number: HNJG-2021-0868). Professor Tu Jianhua of the Principles Teaching and Research Section of the School of Marxism, Hunan City University, China, has made great contributions to this topic, and other colleagues of the teaching and research section have participated in the research of this topic.

## LHMM23061 A CASE OF VP-INDUCED INTRACRANIAL INFECTION WITH AN INTESTINE-PERFORATING SHUNT

Zhiyong Tao^a,b^, Yuan Yuan^a,b^, Yuntao Liu^a,b^, Shunjie Zhong^a,b^, Luotong Liu^a^, Liang Liu^a,c^

*^a^ Department of Neurosurgery, Affiliated Hospital of Southwest Medical University, No.25, Taiping Street, Luzhou City, Luzhou, 646000, China, ^b^ Neurosurgery Clinical Medical Research Center of Sichuan Province, Luzhou, China, ^c^ Neurosurgery Clinical Medical Research Center of Sichuan Province, Neurological Diseases and Brain Function Laboratory, Luzhou, China.*

**Background:** VP shunts provide an important means of treating hydrocephalus, and infection is one of the main complications after a shunt placement operation. Infection can cause intellectual damage and ventricle separation and even lead to patient death. According to the affected site, infections associated with VP shunts can be classified as wound infections, meningitis, peritonitis, and shunt infections. Shunt infection caused by gastrointestinal perforation is a rare complication after VP shunt placement. After gastrointestinal perforation, intestinal bacteria can enter the intracranial system through the shunt, leading to ventriculitis, meningitis and even infection of intracranial nerves and brain parenchyma. At present, the cause of gastrointestinal perforation after VP shunt placement is not clear. We report a case of the intestinal penetration of a traumatic hydrocephalus-associated VP shunt in which intracranial infection resulted.

**Subjects and Methods:** A patient of admitted to the neurosurgery department from October 2011 in Affiliated Hospital of Southwest Medical University was enrolled in the study. A case report.

**Results:** The patient was a 10-year-old male with car accident-related injuries resulting in a consciousness disorder that required hospitalization. The diagnosis at admission was diffuse axonal injury. During hospitalization, the results of a head CT showed that the patient had hydrocephalus, and the patient received a ventricular abdominal shunt. Postoperatively, the patient could open his eyes on his own and had a GCS score of 7 points; 4 months after surgery, the patient developed a high fever. A head CT examination suggested bilateral lateral ventricle posterior angle abnormal density fluid level on imaging, which supported a suspicion of intracranial infection. The patient’s abdominal CT examination showed a drainage tube that visibly ended in the ascending colon. The child underwent laparoscopic exploration, and the abdominal end of the tube was found to have entered the intestine. The patient’s drainage device was removed, and the patient underwent external drainage surgery. Postoperatively, anti-infection and antiepilepsy treatments were given. Two months after surgery, the patient died due to circulatory failure.

**Conclusions:** Infection is one of the main complications after shunt placement; a shunt infection due to gastrointestinal perforation is a rare complication after VP; and neurosurgeons should pay close attention to the development of intracranial infection after VP shunt placement.

### Acknowledgments

Experimental study on the effect of TPO on apoptosis and inhibition of brain edema, Luzhou Science and Technology Bureau, No.2021-JYJ-63.

## LHMM23062 IMPACT OF AGRICULTURAL PRODUCT PACKAGING ON PURCHASE INTENTION AND SENTIMENT BEHAVIOR BASED ON CONSUMER PSYCHOLOGICAL PERCEPTION

Peng Han^a,b^, Zehua Pan^b^, Minhan Xie^a^

*^a^ Xiamen Huaxia University, Xiamen 361024, China, ^b^ Japan and South Korea Creative Entrepreneurship Research Center, Liaoning University, Shenyang 110036, China, ^c^ Jiangxi Industry Polytechnic College, Nanchang 330099, China.*

**Background:** This paper aims to analyze the influence of perceptual elements in agricultural product packaging on the purchase intention and psychological sentiment behavior of consumers from consumer psychological perception.

**Subjects and Methods:** This paper applied a combination of internet content analysis and the IPA model analysis to capture comment information on consumer about agricultural products packaging on the Internet and used the ROST CM6 software to perform high-frequency vocabulary, semantic network, and emotional perception analysis on internet content. This research summarized and encoded the agricultural product packaging elements that affect the purchase intention and emotional behavior of consumers. At the same time, this paper used the IPA analysis to construct the analysis model. It analyzed the influence of the satisfaction and attention of each perceived factor in the packaging of agricultural products on the purchase intention and psychological needs of consumers.

**Results:** The results showed that perceptual factors such as packaging appearance, capacity space, and material significantly impact the purchase intention and emotional behavior changes of consumers. Meanwhile, the aspects of low consumer satisfaction, such as packaging pattern, durability, and bearing capacity, needed to be further optimized.

**Conclusions:** This paper analyzed the influence of perceptual elements on the purchase intention and changes of psychological emotional behavior of consumers from the perspective of consumer psychological perception. It summarized the theoretical ideas of agricultural product packaging design in line with the perceived value and psychological emotional behavior of consumers and enriched the research content of agricultural product packaging.

### Acknowledgements

This work was supported by a project grant from education and research project of young and middle-aged teachers in Fujian Province in 2022, Grant number (JAS22213).

## LHMM23063 TERATOMA RUPTURE WITH HEMORRHAGE IN THE THIRD VENTRICLE: A CASE REPORT

Yuntao Liu^a^, Zhiyong Tao^a^, Yuan Yuan^a^, Shunjie Zhong^a^, Liang Liu^a^

*^a^ Department of Neurosurgery, Affiliated Hospital of Southwest Medical University, Luzhou 646000, China.*

**Background:** Intracranial teratomas, which represent 0.5% of all intracranial tumors and 2–4% of pediatric intracranial tumors, can be classified into mature, immature and malignant types depending on histological variation. Most intracranial germinomas occur in the midline. Teratomas are commonly found in the pineal gland and suprasellar region but rarely in the third ventricle. Rupture of mature teratomas is a rare event. To date, only four cases of intracranial teratoma rupture have been reported: a spontaneous teratoma rupture in a 16-year-old female and a teratoma rupture in a 45-year-old male, while the other two patients died during the fetal period. Intracranial teratoma rupture with hemorrhage, on the other hand, has never been reported. We were the first to report a case of spontaneous rupture and hemorrhage of pediatric teratoma and achieved a good outcome through surgical treatment.

**Subjects and Methods:** This study reports a 9-year-old boy who was hospitalized due to symptoms such as lethargy accompanied by headache, nausea and vomiting. Cranial computed tomography (CT) revealed a mass with hemorrhage in the third ventricle. MRI revealed a mass in the third ventricle with hemorrhage and an intraventricular hematocele.To solve the symptoms caused by hydrocephalus and tumor hemorrhage, bilateral ventriculocentesis and drainage were performed and then tumorectomy was performed through a transcallosal approach. Postoperative pathology showed mature teratoma rupture with hemorrhage.

**Results:** After the operation, the serum AFP decreased to normal, and βHCG was significantly reduced (32.78 mIU/ml). The patient had clear consciousness and little speech when discharged from the hospital. His right upper and lower limb muscle strength was grade 4, his left limb muscle strength was normal. Enhanced MRI reexamination revealed no residual tumor or recurrence 3 months after surgery. Currently, the patient is in good condition and can walk independently without signs of tumor recurrence.

**Conclusion:** To the best of our knowledge, this is the first report of mature teratoma rupture with hemorrhage in the third ventricle. We performed serum βHCG tests on the pediatric patient before and after surgery and found that the preoperative blood βHCG was >1,000 mI/ml, while the postoperative βHCG declined significantly to 32.78 mIU/ml, thus proving the possible diagnostic value of βHCG for teratomas. We proposed a treatment scheme in which the symptoms of tumor apoplexy and hydrocephalus are relieved by ventricular drainage prior to performing tumor resection. With these procedures, the patient had a good outcome, without the presence of residual tumor or recurrence during follow-up.

## LHMM23064 STUDY ON THE PROTECTIVE MECHNISM OF TRADITIONAL MONGOLIAN MEDICINE NAGAB-9 ON ACUTE LUNG INJURY MODEL RATS AND ITS MATERIAL BASE

Sachula Baoyin^a^, Jiuwang Yu^b^, Zhiheng Dong^c^, Dusubilige Wen^d^, Lidao Bao^b^, Lan Wu^d^

*^a^ Beijing University of Chinese Medicine, Beijing, 010030, P. R. China, ^b^ Hohhot Hospital of Traditional Chinese Medicine and Mongolian Medicine, Hohhot, Inner Mongolia, 010030, P. R. China, ^c^ Department of Pharmacy, Affiliated Hospital of Inner Mongolia Medical University, Hohhot, Inner Mongolia, 010059, P. R. China, ^d^ Mongolia Medical School, Inner Mongolia Medical University, Hohhot, Inner Mongolia, 010110, P. R. China.*

S Baoyin and JW Yu contribute equally to this work.

**Background:** In this study, the protective mechnism of traditional Mongolian medicine Nagab-9 on acute lung injury model rats and its material base were discussed.

**Method:** Acute lung injury model were developed using SPF SD male rats by bleomycin thorugh *trachea injection* (3.5 mg/kg*).* Then, model rats randomly devided into Nagab-9 middle dose (126 mg/kg), Nagab-9 high dose (252 mg/kg) and dexamethasone (positive control, 5mg/kg) groups, 12 rat each group. Rats of sham-operated (*the sa*me amount of *physiological saline)* group were given *sa*me amount of *physiological saline*. Drug was given for once a day and continued for 21 days. Lung pathological changes were observed by HE staining method. The level of serum TNF-a and IL-6 were detected by ELISA and qPCR. The differentially expressed genes comparing different groups were examined by transcriptional screening technique and validated by qPCR. Also, related signaling pathways were analyzed through KEGG and GO analysis. The active metabolic compounds of Nagab-9 were tested by UPLC-QE-MS/MS untargeted metabolic assay and the active molecules from Nagab-9 were observed. Furthermore, AutoDock Tools (1.5.6) was used to analyze the detected active molecule of Nagab-9 and its target. Finally, the level of NF-kB in lung tissue was tested by immunohistochemistry and the level of proteins Raf, p-Raf, MEK1/2, p-MEK1/2, ERK1/2, p-ERK1/2, Nur77 was analyzed by western blot.

**Result:** Nagab-9 effectively reduced the level of inflammatory factors TNF-a and IL-6 in serum of ALI rats. *Transcriptomic analysis found the expression level of* NR4A1, Raf, Mek, ERK and NF-kB genes were significantly regulated by Nagab-9. KEGG and GO analysis showed MAPK signaling pathway was significantly mediated, especially, NR4A1 was highly expressed in model. S*erum pharmacology result showed 24 of* Nagab-9 compounds. Among them, small molecule lidocaine showed coorelation with MEK/ERK and NF-kB pathway. Molecular docking result revealed that the the interacting score of lidocaine and Nur77 was -5.0. Lidocaine was also detected and quantified in blood sample. qPCR and western resluts indicated that Nagab-9 can significantly down-regulate NR4A1, Raf/Mek/ERK, NF-kB at protein level in the lung tissue of ALI rats.

**Conclusion:** Nagab-9 can effectively relieve normal symptoms and lung pathalogical changes of ALI rats. The possible molecular mechanism is that the small molecule lidocaine from Nagab-9 inhibit the expression of NR41 and prevent Raf/Mek/ERK pathway, thereby prohibit Raf/Mek/ERK pathway and consequently decrease NF-kB inflammation and reaction.

### Acknowledgments

This research is funded by the following projects: Natural Science Foundation of China (No. 82160794 and 82160703); Natural Science Foundation of Inner Mongolia Autonomous Region (No. 2020MS08106); Project of Health Science and Technology in Inner Mongolia Autonomous Region (No. 202201238); Inner Mongolia Autonomous Region Chinese Medicine (Mongolian Medicine) Young and Middle-aged Leading Talents Cultivation Project; Grassland Yingcai in Inner Mongolia Autonomous Region; Qihuang Scholar.

## LHMM23065 INFLUENCE MECHANISM OF SUPPLIER-CUSTOMER RELATIONSHIP NETWORK TO PROMOTING HIGH-QUALITY TRADE DEVELOPMENT FROM THE PERSPECTIVE OF DIGITAL ECONOMY: FROM THE PERSPECTIVE OF EMOTION REGULATION

Yanhong Li^a^, Fangfang Ma^b^, Gongxing Wu^b^, Huiyuan Zou^b^

*^a^ School of Management, West Yunnan University of Applied Sciences, Dali, 671000, China, ^b^ School of Management Engineering and Electronic Commerce, Zhejiang Gongshang University, Hangzhou, 310018, China.*

**Background:** In recent years, the COVID-19 pandemic, the China-Us trade war and other events have caused an unprecedented impact on globalization. The profound adjustment and restructuring of the global industrial chain have had a profound impact on the supplier and customer relations and trade development of enterprises. At the present time of fluctuating global economic situation, how to build a solid trade relationship network and consolidate the psychological and emotion relationship network of both sides of the trade is worth in-depth discussion in a scientific and effective way. In the context of the digital economy, this study analyzes and discusses the mechanism of the supplier - customer relationship network affecting the high-quality trade development from the perspective of emotion regulation.

**Subjects and Methods:** Using enterprise supplier - customer data to construct enterprise supplier - customer relationship network, calculate PageRank value to depict enterprise supplier - customer relationship strength, and use the data of China’s A-share listed companies from 2010 to 2020 to carry out empirical research. The influence mechanism of supplier - customer relationship network on trade quality development under the adjustment of digitalization degree and marketization degree is analyzed. The methods proposed in this study can also be further developed theoretically from the perspective of emotional regulation. Through the construction of a network of psychological and emotional exchanges and win-win cooperation, it can provide strong support for a more solid and stable e-commerce trade, so as to promote the high-quality development of China’s trade.

**Results:** The results show that both the supplier and customer network relationship and its proportion have a positive and significant impact on the development of trade quality. Both the degree of digitalization and the degree of marketization have a positive interaction effect on the relationship between supplier - customer relationship network and trade quality development. Moreover, in the market environment with weak environmental uncertainty, the supplier - customer relationship network is more significant to the high-quality trade development, and the moderating effect of the degree of digitalization and the degree of marketization of enterprises is also stronger. Under a stable emotional regulation network, both sides of the trade can adjust their trade psychological health and maintain high-quality economic and trade development with positive behaviors and communication trends.

**Conclusions:** Based on the calculation results and theoretical analysis of this study, we can deeply study the influence mechanism of supplier-customer relationship network to promoting high-quality trade development from the perspective of digital economy and emotion regulation, analyze the various characteristics of cross-border e-commerce in modern enterprise trade which are different from traditional trade, and analyze how to regulate supplier-customer emotional network relationship, maintain a positive attitude under the situation of good behavior, high-quality economic and trade development. At the end of the paper, some directional suggestions are given for further research.

### Acknowledgments

Supported by the Digital Trade and Silk Road E-commerce Engineering Research Center of Yunnan Provincial Education Department and Major scientific and technological support projects in Dali prefecture “The Application of Digital Trade and Cross-border E-commerce in Dali Prefecture”.

## LHMM23066 APPLICATION OF CALCIUM POLYCARBOPHIL COMBINED WITH COMPOUND POLYETHYLENE GLYCOL ELECTROLYTE POWDER ON BOWEL PREPARATION FOR COLONOSCOPY

Jiayi Wang^a^, Ri Chen^a^, Qihui Ma^a^

*^a^ Cilin Hospital, Ningbo, Zhejiang Province, China.*

**Objective:** To explore the effect and safety of calcium polycarbophil combined with compound polyethylene glycol electrolyte powder on bowel preparation for colonoscopy.

**Methods:** A total of 539 patients undergoing colonoscopy and treatment in Cixi Cilin Hospital, Ningbo, Zhejiang Province from January 2021 to July 2022 were randomly divided into three groups: A, B and C. Study group A (n=180) adopted calcium polycarbophil tablets within 48 hours before taking compound polyethylene glycol electrolyte powder in preparation for intestinal colonoscopy. Study group B (n=180) adopted calcium polycarbophil tablets within 72 hours before taking compound polyethylene glycol electrolyte powder in preparation for intestinal colonoscopy. Control group C (n=179) only took compound polyethylene glycol electrolyte powder for intestinal cleansing. The intestinal cleaning effect and antifoam effect of the three groups were evaluated by the Boston Bowel Preparation Scale (BBPS), and the adverse reactions during the bowel preparation of the three groups were observed.

**Results:** The Boston scores were corrected by the Bonferroni method. The intestinal cleanliness and antifoam effect of the patients in study group A and study group B were significantly higher than those in control group C and there were significant differences (P<0.001). There were no significant differences in intestinal cleaning effect and antifoam effect of the patients between group A and group B (P=0.241, P>0.05) which points the effects in the two groups were equivalent. No serious adverse reactions occurred in all three groups, and the incidence of adverse reactions was low without statistical difference between groups (P>0.05).

**Conclusion:** The effect of taking calcium polycarbophil tablets combined with compound polyethylene glycol electrolyte powder for intestinal preparation 48 hours and 72 hours before colonoscopy are better than that of using compound polyethylene glycol electrolyte powder alone which are safe and acceptable with low incidence of adverse reactions and no serious adverse reactions.

### Acknowledgments

This work was supported by the [Science and Technology Plan Project of Cixi City, Zhejiang Province] under Grant [number CN2018010].

## LHMM23067 EFFECT OF PREOPERATIVE COMMUNICATION USING CARTOON VIDEO IN POSTOPERATIVE COMPLICATIONS IN CHILDREN UNDERGOING TONSILLECTOMY: A RANDOMISED CONTROLLED TRIAL

Wujun Zou^a^, Qian Li^a^, Yesong Cheng^a^, Fei Peng^b^, Dingqiang Huang^a^

*^a^ Department of otorhinolaryngology head and neck surgery, Chengdu Second People’s Hospital, Chengdu 610000, China, ^b^ Department of Anesthesia, West China Hospital of Sichuan University, Chengdu 610000, China.*

**Background:** Tonsillectomy is one of the most frequent operations performed by otolaryngologists in children. Hence, it is essential to prevent postoperative complications in children undergoing tonsillectomy. A successful preoperative education might prevent postoperative complications. This study aimed to investigate the effect of preoperative education using a cartoon video for the prevention of postoperative complications in children scheduled for tonsillectomy.

**Subjects and Methods:** A total of 154 patients scheduled for elective tonsillectomy met the inclusion and exclusion criteria and were randomly divided into the control (77 cases) and cartoon video (77 cases) groups. Patients’ eline data were carefully recorded. The duration of operation, intraoperative blood loss, postoperative diet recovery time, VAS pain scores on the first, third and seventh day after surgery, tonsillar fossa infection, the incidence of postoperative bleeding, upper respiratory symptoms and digestive symptoms was evaluated.

**Results:** Compared with the control group, the average visual assessment score (VAS) pain scores on days 1 and 3 after surgery were significantly decreased in the cartoon video group (P < 0.05). Moreover, the eating time was markedly reduced in the experimental group (P < 0.05). There was no significant difference in intraoperative blood loss, postoperative pain score (day 7), postoperative bleeding, tonsillar fossa infection, digestive symptoms and upper respiratory tract infections.

**Conclusions:** For children scheduled for tonsillectomy, preoperative communication using the cartoon video can effectively improve postoperative recovery. The cartoon video can help children understand the contents of preoperative education more easily, deserving extensive promotion.

## LHMM23068 RESEARCH ON WAYS TO IMPROVE THE SUBJECTIVE HAPPINESS OF E-COMMERCE EMPLOYEES FROM THE PERSPECTIVE OF EMPLOYMENT QUALITY

Chen Chen^a^

*^a^ School of Business Management, Hangzhou Polytechnic of Science and Technology, Hangzhou, China.*

**Background:** By investigating the employment quality of e-commerce employees and its impact on Subjective Happiness, this paper proposes countermeasures for improving employment quality that can improve the happiness of e-commerce employees, and provides opinions and suggestions for e-commerce employees, governments, universities and enterprises.

**Subjects and Methods:** Taking e-commerce employees of small and medium-sized enterprises in Hangzhou, Ningbo, Wenzhou, Taizhou, Jinhua and Yiwu as the research objects, this paper adopts the subjective and objective evaluation indicators of employment quality and the data of 379 valid questionnaires collected from the survey to study the employment quality and its impact on Subjective Happiness. In the research process, questionnaire survey, field survey, interview and other methods are mainly adopted. The questionnaire designed was answered by Likert’s 5-point scale. In the data processing process, SPSS software and Excel were mainly used to carry out statistical analysis. For example, univariate analysis (frequency and frequency, mode, median, mean, variance), bivariate analysis (correlation coefficient, regression analysis), or multivariate correlation analysis is used.

**Results:** (1) Among the four dimensions of employment quality, the average score of interpersonal relationship is the highest, followed by job adaptation and career development, and the score of work intensity is the lowest. (2) Most people think they can adapt to their current jobs, and as they grow older, they become more adaptable; (3) E-commerce employees work long hours and are under great pressure, but they are still looking forward to their work; (4) Most people pay more attention to whether their job has development prospects and whether doing this job has harm to their health. The happiness index of a job with prospects and good health is high; (5) They think their spare time is colorful, but they don’t have enough time to spend with their families; (6) Career development, job adaptation and interpersonal relationship have a significant positive impact on job Subjective Happiness, and work intensity has a significant negative impact on job Subjective Happiness; Career development, job adaptation and interpersonal relationship have a significant positive impact on Life Subjective Happiness, while work intensity has a significant negative impact on Life Subjective Happiness.

**Conclusions:** In order to improve the Subjective Happiness of e-commerce employees, it is suggested that the government level, enterprise level and individual level make joint efforts to create a fair and just competition environment for the e-commerce industry and promote the realization of higher quality employment of e-commerce employees, including the establishment of supervision mechanism and the improvement of social insurance mechanism. Pay attention to the career development of employees, improve the welfare and compensation system, give full play to the initiative of employees; Do a good job planning, according to interests and professional ability to choose positions and other countermeasures.

## LHMM23069 UTAUT MODEL-BASED RESEARCH ON INFLUENCING FACTORS OF BEHAVIORAL EMOTIONS IN THE MOBILE LEARNING PROCESS OF MEDICAL ENGLISH

Yuxiao Luo^a^, Jun Deng^a^, Zhihua Liao^b^, Si Chen^c^, Rongjun Zhao^a^

*^a^ Department of Foreign Languages, Zhongshan Institute, University of Electronic Science and Technology of China, Zhongshan, Guangdong, 528402, China, ^b^ Department of Humanities, Shanghai University of Finance and Economics, Shanghai, 200433, China, ^c^ Department of Nursing, Southwest Medical University, Luzhou, Sichuan, 646000, China.*

**Background:** Mobile learning has been regarded as an essential scheme to optimize the allocation of educational resources and to ensure the fairness of education. Scholars both domestically and internationally have conducted the extensive research on mobile learning by utilizing the UTAUT model and other related prior models, in order to glean insights and ideas for improving learners’ emotional and behavioral experiences during the mobile learning process. However, compared with the general College English, the influencing factors of behavioral emotions in the mobile learning process of Medical English have rarely been included in the current massive amounts of studies.

**Methods:** Based on the UTAUT model, a schematic model of the influencing factors was constructed, and a quantitative questionnaire was designed to profoundly explore the influencing factors of behavioral emotions in the mobile learning process among Medical English learners. The questionnaire scale includes participants’ moderating factors, critical factors, and behavioral emotions, which involves 50 declarative sentences. The simple random sampling was applied to choose 180 participants from 360 learners in Medical English. The descriptive statistics, Pearson Chi-Square analysis, Pearson correlation analysis, and regression analysis have been employed to validate hypotheses.

**Results:** There are no significant differences in participants’ behavioral emotions including potential intentions and genuine attitudes to embark on the mobile learning behaviors with the aid of modern information technology when participants are grouped into male-science, male-liberal arts, female-science, and female-liberal arts. There is a positive significant correlation between participants’ voluntary and four critical factors. There is also a positive significant correlation between participants’ experience and four critical factors. Participants’ moderating factors positively correlate with their behavioral emotions, including potential intentions and genuine attitudes. The univariate linear regression analysis shows that there is a linear regression relationship between participants’ extent of facilitating condition and their behavioral emotions, between participants’ extent of effort expectancy and their behavioral emotions, between participants’ extent of social influence and their behavioral emotions, and between participants’ extent of performance expectancy and their behavioral emotions.

**Conclusions:** The results of this study imply that participants’ psychological capital can not only help them identify the critical influencing factors, but also mobilize their own behavioral emotions including potential intentions and genuine attitudes, which may finally be transformed into the practical mobile learning behaviors. This research may not only provide some inspirations for administrators and teachers to improve behavioral emotions of learners in the mobile learning process, but also usher in some theoretical and practical implications for researchers who intend to conduct relative studies in the near future.

### Acknowledgements

Supported by projects’ grant from Research on Influencing Factors of Learners’ Stickiness in the First-Class Course from the Perspective of Social Cognition Theory (Grant No. GD22XJY18); Research on the Intervention Mechanism of College Students’ Psychological Health Based on Self-Regulation (Grant No. 2021GXSZ117).

## LHMM23070 THE EFFICACY AND MECHANISM OF POSTSTROKE SWALLOWING DYSFUNCTION BASED ON BOLD-FMRI

Yanxia Qin^a^, Chuna Chen^a^, Pan Chen^a^, Xiaojun Jin^b^, Qingtian Meng^a^, Xuchang Zhang^c^

*^a^ Department of rehabilitation medicine, Second Affiliated Hospital of the Chinese University of Hong Kong, Shenzhen, 518172, China, ^b^ Imaging Department, Second Affiliated Hospital of the Chinese University of Hong Kong, Shenzhen, 518172, China, ^c^ Department of Geriatric Medicine, Second Affiliated Hospital of the Chinese University of Hong Kong, Shenzhen, 518172, China.*

**Background:** It is well known that swallowing is a more complex sensorimotor activity, which includes both the voluntary movement but also the non-voluntary movement. All of these movements require the brain stem, the cortex and the nerves around the brain stem to regulate. Stroke is an important cause of patients with swallowing disorders, in the process of clinical treatment of swallowing disorder patients after stroke, medical staff by taking transcranial direct current stimulation has achieved good experimental results, and transcranial direct current stimulation as a new type of non-invasive brain nerve stimulation technology, is increasingly applied to the clinical after stroke swallowing function rehabilitation. However, the injury mechanism after cerebral hemisphere stroke is yet clear. This article mainly analyzes by taking the stroke patients transcranial direct current stimulation can improve the specific function of swallowing, and contrast into the patients of side, side swallowing cortex transcranial direct current excitatory stimulation after specific curative effect difference, the application of blood oxygen level dependent functional magnetic resonance imaging (BOLD-fMRI) technology in before and after tDCS treatment brain structure and function of imaging data, analysis of tCS on the cortex, clear swallowing function rehabilitation and brain function activation mode, location is relationship.

**Subjects and Methods:** By selecting 60 patients with swallowing disorders who came to our hospital from January 2021 to November 2022, and by using a random allocation of numbers. They were divided into three groups: affected side group, healthy side group and control group, for these three groups of patients are subjected to routine swallowing training, ice stimulation, and functional electrical stimulation therapy, for patients in the healthy group and patients in the affected group. On the basis of swallowing training, ice stimulation, and functional electrical stimulation therapy, give excitatory stimulation on the healthy side and excitatory stimulation on the affected side, respectively.

**Results:** After treatment of the 60 patients, the SSA scores were significantly higher in all three groups than before treatment, Healthy side group> affected side group > control group; swallowing lithography (VFSS) examination: before treatment than after treatment, and healthy side group > affected side group > control group; cranial fMRI examination: blood oxygen level dependent method (BOLD-fMRI) examination showed bilateral anterior central gyrus, posterior central gyrus, cingulate gyrus, insula, prefrontal, cuneus swallowing activation area, respectively, more than and stronger than before treatment, and the healthy side group > affected side group > control group.

**Conclusions:** In conclusion, after treatment, the patients in the healthy group were significantly higher than patients in the affected group and the control group, and the difference between the affected group and the control group was not statistically significant.

### Acknowledgements

This work was supported by the Shenzhen Longgang District Economic and Science and Technology Development Special Fund for Medical and Health Science and Technology Project (LGKCYLWS2020001764).

## LHMM23071 EVALUATION OF RELIABILITY OF ULTRA-FAST BIO-MONITORING METHOD IN MONITORING HIGH-PRESSURE STERILIZATION EFFECTS

Yanqiu Yang^a^, Shuoyang Zhao^b^

*^a^ Director of Hospital Infection-Control Department, Beijing Daxing District People’s Hospital, Beijing 102600, China, ^b^ Beijing Jishuitan Hospital, Beijing 100096, China.*

**Background:** To evaluate the reliability of ultra-fast fluorescence bio-monitoring method in high pressure steam sterilization monitoring.

**Subjects and Methods:** The software program SPSS 20.0 (IBM, Chicago, USA) was used to conduct the statistical analysis. Continuous variables were expressed as mean ± standard deviation (SD). Discontinuous variables were expressed in percentage (%). P<0.05 was considered statistically significant. We compared two methods (ultra-fast fluorescence bio-monitoring and traditional bio-monitoring) under three conditions: 134°C/70 S, 134°C/160 S and 134°C/250 S. The sterilization effect of pressure steam was monitored by these two methods, and the reliability of the ultra-fast fluorescent biological method was evaluated by comparing this with the results obtained through the conventional bio-monitoring method.

**Results:** The one-hour ultra-fast fluorescence test result obtained by the biological reader was highly consistent with the result of the 48-hour incubation with ultra-fast bio-indicators, as well as the incubation result after seven days using the traditional biological monitoring method. It was found that there was no significant difference between these. Using the traditional biological monitoring methods, the cultivation needs a long time, which is not good to the turnover and effective use of surgical instruments. The purchase of more surgical instruments for turnover will need more money, which is generally not approved by the hospital. A better solution is speeding up the cleaning, disinfection and sterilization of surgical instruments. Biological monitoring is the gold standard for surgical instruments, especially those with implants or lumens, and sterilization is mainly a process of killing pathogenic microorganisms. Therefore, the parameters of accelerated biological monitoring were selected for verification, to ensure the safety of surgery and medical treatment while ensuring the speed. When the ultra-fast bio-indicator was self-cultured, 36 of these turned yellow at 48 hours, and 36 of these turned yellow at 168 hour. There was no increase in positive results, and the results detected within one hour were consistent with the final biological culture results. The color change results were identical with those of the traditional bio-monitoring sterilization indicator at the seven-day culture.

**Conclusions:** The one-hour ultra-fast fluorescence bio-monitoring method is extremely accurate and reliable. This method can be widely used to improve the time-efficacy of biological monitoring. In conclusion, the results obtained by this kind of ultra-fast fluorescence biological monitoring method are both accurate and reliable, and can be used in the biological monitoring of high-pressure steam sterilization. This can fundamentally solve the limitation of the long monitoring of traditional biological indicators, realizing a seamless workflow process of cleaning, packaging, sterilization and distribution, and thereby improving the turnover rate and work efficiency of equipment.

## LHMM23072 THE INFLUENCE OF INTERGENERATIONAL SUCCESSION AND PSYCHOLOGICAL ANXIETY FOR FAMILY FIRM INTERNATIONALIZATION: AN EMPIRICAL STUDY BASED ON THE MEDIATING EFFECT

Shaoyu Dong^a^, Pengfei Li^b^

*^a^ School of Business Administration, Northeastern University, Shenyang, China, ^b^ School of Computer Science and Engineering, Northeastern University, Shenyang, China.*

**Background:** Chinese family firms have entered a peak period of inheritance, development, transformation, and upgrading; intergenerational succession is bound to affect family firms substantially. The smooth inheritance of a family firm is not only related to the longevity of the firm itself but also to the healthy and sustainable development of the national private economy. Therefore, seeking global-scale development opportunities has become essential for sustainable development for family firms. However, current studies mainly focus on the influence of family firms’ internationalization based on the intergenerational succession scale. Moreover, psychological anxiety is another important factor that can impact family firm internationalization. Starting or expanding a business in a foreign market can be a daunting task, and psychological anxiety can make this process even more challenging. Few studies focus on family firm successors’ psychological anxiety at different stages of succession, and few focus on the intrinsic logic and action mechanism of second-generation successors’ impact on family firm internationalization. In this paper, we innovatively combined the concept in cognitive psychology to understand better the influence of intergenerational succession and psychological anxiety on family firm internationalization.

**Subjects and Methods:** In this paper, we explored the internal mechanism of intergenerational succession that affects the degree of internationalization of family firms and further proposed a series of theoretical hypotheses based on the characteristics of China’s trade policy environment and the actual situation of the intergenerational succession of family firms. This paper takes 1219 listed family businesses in China from 2010 to 2018 as a sample to study intergenerational succession’s impact on the degree of internationalization.

**Results:** By providing comprehensive empirical tests, we found a positive correlation between family firm intergenerational transfer and the degree of internationalization. Further tests, such as instrumental variables, Tobit regression, and robustness tests, show that: (1) intergenerational succession has a significant positive impact on the degree of family firm internationalization, and the results still hold after considering potential endogeneity issues and conducting a series of robustness tests.; (2) through analyzing of mediating effect test results, we find that increases in long-term borrowing and corporate social responsibility is an important means for intergenerational succession to enhance internationalization; (3) the heterogeneity analysis results reveal that family firms belonging to the high-tech industry can promote the degree of internationalization in the stage of intergenerational succession, and compared with the period of parent-child co-governance, “second-generation autonomy” can promote the degree of internationalization because it eliminates the psychological anxiety of managers.

**Conclusion:** Our study enriches the research in intergenerational succession on the degree of internationalization of family firms, and we also provide a reference for exploring family firm inheritance and maintaining long-term competitiveness by providing four aspects of suggestions.

### Acknowledgments

This work was supported by National Natural Science Foundation of China (No. 71873027); Humanities and Social Science Foundation of Ministry of Education of China (18YJA790063).

## LHMM23073 RESEARCH ON EVALUATION OF ENTERPRISE SAFETY CULTURE EFFICIENCY BASED ON DEA-TOBIT MODEL AND MANAGEMENT PSYCHOLOGY: FROM THE PERSPECTIVE OF EMPLOYEE MENTAL HEALTH

Renlin Guo^a^

*^a^ Beijing Enterprise Safety and Environment Information Science Research Institute, Beijing, China.*

**Background:** Psychological health is the foundation of safety culture. This article uses the DEA-tobit model to evaluate the safety culture of enterprises, and proposes policy recommendations to improve the level of psychological health.

**Subjects and Methods:** Mental health is the focus and important aspect of safety culture construction. Based on clarifying the concept of mental health, this article reviews the literature on mental health and evaluates the efficiency of safety culture from the perspectives of industry and enterprises using the DEA-Tobit model. The level of mental health is calculated, and policy recommendations are proposed based on the research conclusions.

**Results:** Research based on the measurement of mental health and safety culture shows that there is a positive correlation between mental health and safety culture levels in coal mines. The higher the level of mental health, the higher the level of safety management in coal mining enterprises. Therefore, coal mining enterprises must clarify the importance and necessity of mental health, in order to build a strong level of safety culture, reveal the relationship between safety culture and mental health, and have significant implications for improving the level of coal mine safety management. This will enable coal mines to increase safety investment, establish long-term mental health concepts, and comply with various safety production and investment regulations.

**Conclusions:** The research conclusions of this article include improving the mental health level of coal mines, increasing safety investment, establishing a soft culture of coal mine safety, enabling employees to establish correct mental health concepts, accelerating enterprise modernization, improving safety management level, raising the entry threshold for frontline employees from the perspective of mental health, strengthening investment in safety training and education, improving and improving labor union organizations, and increasing investment in safety funds and scientific research funds, Effectively improving the level of mental health and safety culture.

### Acknowledgments

This research was supported by Shandong Social Science Planning and Research Project: Research on the comprehensive allocation level measurement and optimization strategy of urban innovation factors in Shandong Province under the digital economy (22DGLJ23).

## LHMM23074 A STUDY ON THE INFLUENCE OF TRADITIONAL DANCE REPRODUCTION ON DANCERS’ PSYCHOLOGICAL HEALTH BASED ON DANCE PSYCHOLOGY

Wei Qin^a^, Changgan Fang^b^

*^a^ Youth League Committee of Wuzhou University, Wuzhou, Guangxi 543002, China, ^b^ Department of Scientific Research, Wuzhou University, Wuzhou, Guangxi 5430002, China.*

**Background:** With the continuous deepening of global modernity, traditional dance must undergo new adaptation and reproduction under the influence of modernity. Dancers have psychological problems such as stage fright, excessive excitement, and ineffective emotional expression in the process of traditional dance reproduction.

**Subjects and Methods:** From the perspective of dance psychology, this study takes dancers in the reproduction process of the Zhuang traditional board shoe dance “run for the sun” as the research object, and uses qualitative research methods to conduct the research. By using the researcher as the research instrument, the non-scale methods such as personal in-depth interviews, focus group interviews, state observation, and psychoanalysis were employed to investigate the dancers’ psychological phenomena as a whole in a natural context. The author collected and recorded data in various aspects, analyzed the data using inductive methods to form theories, gained an interpretive understanding of the dancers’ movement behaviors and meaning constructions through interaction with the research objects.

**Results:** Based on the choreographer’s requirements of neatness, upper-air technical difficulty, and character portrayal for the dancers in the traditional dance reproduction, the psychological problems that the dancers encounter or exhibit in the traditional dance reproduction and the adverse effects on the dancers’ physical and psychological health are analyzed. On this basis, by observing the dancer’s psychological quality and character traits, it was analyzed that the dancer’s psychological problems are caused by unsound psychological quality, which trigger unstable behaviors such as nervousness and stiff limbs. Based on the analysis of psychological problem images and causes, effective methods are proposed to overcome the psychological problems in the traditional dance reproduction, and finally the reproduction of traditional dance and the construction of dancers’ psychological health are completed.

**Conclusions:** The results show that psychological intervention based on dance psychology can achieve the effects of stabilizing self-emotions, improving self-confidence and enhancing cultural quality, which plays an important role in the cultivation of dancers’ physical and psychological health.

### Acknowledgments

This work was supported by the 2019 Annual Stage Art Creation Founding Project of National Arts Foundation under Grant 2019-A-02-(079)-0211.

## LHMM23075 UREAL MASS OF MISDIAGNOSED VAGINAL VEGETATION

Yuan Wen^a^, Tiantian Wang^a^, Li Zhu^a^, YanQiong Gou^a^, Hui Tan^a^, JinZhi Ma^a^

*^a^ Yellow River Sanmenxia Hospital, Henan Province, Sanmenxia 472000, China.*

**Background:** Objective Analysis found that the young urethral orifice mass as the main manifestation, and misdiagnosed as vagina, clinical diagnosis and treatment of vegetation, in order to improve the understanding of the disease.

**Subjects and Methods:** Reviewing a case admitted to Sanmenxia Hospital in Henan Province, the clinical and laboratory examination data from January 27, 2023 to February 2, 2023, reviewed relevant documents, analyzed the causes of the misdiagnosis and summarized the literature.

**Results:** In this case, the child was misdiagnosed as the vaginal burden because the child could not cooperate with the examination, and the final intraoperative exploration was hysteroscopy after anesthesia, and after detailed examination, it was confirmed as the uroid mass, and the pathology was confirmed as condyloma at the urethral mouth after resection. The main reasons for delayed diagnosis and misdiagnosis include: lack of awareness of the disease. Failed to grasp the exclusion features of diagnosis, the medical history was not detailed, and the children could not cooperate with the physical examination. The incidence of vaginal and urethral mass was low. Young period, vaginal and urethral anatomical position, when long vegetation larger, mass root through simple physical examination is not easy to find, children fear and fear for detailed physical caused difficult.

**Conclusions:** the conclusion of seemingly young vaginal oral redundant cases for clinical characteristics, because of the young vaginal, urethral opening distance close, difficult to identify, need to find the urethral opening anatomical location if failed to find obvious urethral entrance or children completely cannot cooperate, need to consider the urethral opening mass, and urethral opening mucosal valgus may be, need further examination under anesthesia. In order to avoid misdiagnosis and mistreatment. Condyloma acuminata is less rare, condyloma acuminata growing around the urethral opening is rarer, friction tissue edema, easy to misdiagnosed vaginal tumor and urethral opening mucosa valgus. Therefore, young girls to prevent such diseases pay attention to improve their own immunity and resistance, but also to prevent other ways of infection.

## LHMM23076 EXPLORING THE IMPACT OF CONNOTATIVE DEVELOPMENT ON HIGHER EDUCATION DEVELOPMENT IN CHINA: REGIONAL DISPARITIES AND STUDENTS’ COLLEGE-GOING ANXIETY

Qian Li^a^, Dawei Liu^a^

*^a^ College of Public Management, Guizhou University of Finance and Economics, Guiyang 550025, China.*

**Background:** Whether the regional disparities in the development of higher education in China have changed since the implementation of the connotative development has received widespread attention from the society. However, few studies have focused on this issue based on statistical data. The regional disparities in higher education development have a direct impact on the difficulty and quality of students’ college admission, which in turn can affect the psychological needs of students from different regions for entering their ideal universities and their anxiety towards college admission.

**Subjects and Methods:** Using a panel data approach covering the period of 2013-2020, this study comparatively analyzed the regional disparities in higher education development in the Eastern, Central, Western, and Northeastern regions of China as well as across provinces. Furthermore, the study explored the possible impact of these disparities on students’ anxiety about college admission.

**Results:** From 2013 to 2020, the number of regular institutions of higher education per million people in China increased from 1.82 to 1.94. Notably, the densities of institutions of higher education in Beijing and Tianjin were much higher than in other provinces, at 4.20 and 4.04 institutions per million people, respectively. With regard to high-level educational resources, in 2020, the number of doctoral students in Beijing exceeded that of undergraduate students by 20%, while the number of master’s students exceeded that of undergraduate students by 55%. Meanwhile, in some western provinces, the number of doctoral students was still less than 1% of the undergraduate student population. The proportion of GDP allocated to higher education increased from 1.33% in 2013 to 1.41% in 2020 on a national level.

**Conclusions:** Since the implementation of the connotative development, the disparity in the scale of higher education among different regions in China has gradually narrowed. The distribution of high-level educational resources in the central and western regions lags significantly behind that in the eastern regions. The regional differences in high-level education have a direct impact on students’ psychological expectations for college entrance, which in turn leads to varying degrees of college-going anxiety.

### Acknowledgments

This paper was supported by the Humanities and Social Sciences Project of the Ministry of Education of China (22XJA790005) and Research Initiation Projects for Introduced Talents of Guizhou University of Finance and Economics (2018YJ44).

## LHMM23077 CUSTOMER KNOWLEDGE SHARING INCENTIVE MECHANISM BETWEEN ELECTRONIC RETAILERS AND MANUFACTURERS IN THE BIG DATA ENVIRONMENT: BASED ON EMOTIONAL BEHAVIOR

Nali Shen^a,b^; Yunru Si^c^; Jian Xiao^d^

*^a^ Business School, Southwest University of Political Science and Law, Chongqing 401120, China, ^b^ China Region Public Brand Research Center at Southwest University of Political Science and Law, Chongqing 401120, China, ^c^ School of Public Management, Chongqing University, Chongqing 400044, China, ^d^ School of Mathematics and Statistics, Chongqing University, Chongqing 401331, China.*

**Background:** In this paper, we study the incentive mechanism of customer knowledge sharing and revenue sharing designed by the manufacturer as the core firm in the supply chain based on emotional behavior in big data environment. The value of customer knowledge sharing and potential barriers were analyzed, then by designing an effective incentive mechanism to create a positive emotional climate that fosters customer knowledge sharing and ultimately leads to better cooperation benefits for both retailers and the manufacturer.

**Subjects and Methods:** Considering retailers’ emotional behaviors, a principle-agent theory method was used to build mathematical models to analyze behaviors of manufacturers and retailers. The organization emotional behavior theory about incentive was also used to analyze the original organizational psychological states and changes after executing incentive mechanism. And a numerical method was applied to describe change trends of the main factors for knowledge sharing between electronic retailers and manufacturers.

**Results:** there is a positive correlation between a single e-retailer’s effort level of customer knowledge sharing to maximize its expected revenue based on its emotional behavior in big data environment, and the proportion of revenue sharing and the number of e-retailers owned by the manufacturer. The optimal revenue sharing ratio obtained by e-retailers is negatively correlated with the manufacturer’s customer knowledge innovation ability and the number of e-retailers owned by the manufacturer, but positively correlated with the e-retailers’ customer knowledge sharing ability.

**Conclusions:** Revenue sharing incentive is an effective mechanism based on organization emotional behavior. The number of manufacturer-owned e-retailers have a positive effect on e-retailers’ efforts to improve customer knowledge sharing. The optimal revenue-sharing ratio was related with the effort elasticity coefficient the manufacturer and retailers, and emotional behaviors would affect their efforts in the process of knowledge sharing. The incentive mechanism for customer knowledge sharing has changed the organizational emotional behavior of retailers, affected the emotional behavior of manufacturers, would promote knowledge sharing between both parties, and ultimately improved cooperation performance. Therefore, in the knowledge sharing cooperation between retailers and manufacturers in big data context, analyzing the organizational emotional behavior before cooperation, designing incentive mechanisms to affect their emotional behaviors, motivating both parties to make efforts to share customer knowledge, which can promote cooperation benefits for both parties.

### Acknowledgments

This work supported by a project grant from National Social Science Fund of China (18BGL102).

## LHMM23078 CONTRASTIVE ANALYSIS OF INDOOR AIR QUALITY OPTIMIZATION SCHEMES FOR CLASSROOMS IN WINTER UNDER THE GUIDANCE OF PUBLIC HEALTH

Wenjing Dong^a^, Runzhao Qi^b^, Xue Bai^c^, Ke Huang^a^

*^a^ School of Architecture and Urban Planning, Chongqing Jiaotong University, Chongqing, China, ^b^ School of Architecture and Environmental Art, Sichuan Fine Arts Institute, China, ^c^ Institute of Architecture & Planning Design Co., Ltd., Chongqing University, Chongqing, China.*

**Background:** Public health is a public service that is related to the public health of a country or region, and the prevention of major infectious diseases is the top priority. Driven by the public health affairs of the global COVID-19 outbreak, people pay more and more attention to the indoor air quality in public places. Indoor air quality in public places plays a decisive role in public health, and is an important factor affecting the physical health, work efficiency and mental health of users. As a crowded place, creating good indoor air quality is the necessary guarantee to ensure the physical and mental health of teachers and students and improve the efficiency of teaching activities.

**Subjects and Methods:** Taking public health as the guide, this paper combines many technical measures for the reconstruction of existing classrooms to form different reconstruction schemes, and uses numerical simulation method to compare and analyze the indoor air environment quality of a university classroom in Chongqing under different schemes. The article puts forward built-in thermal insulation, closing the gaps of doors and windows, and setting fresh air as the transformation measures. At the same time, taking into account the use conditions of opening and closing doors and windows, the paper selects five factors that affect the indoor air quality: temperature, humidity, CO2 concentration, O2 concentration, and PM2.5 concentration for comparative analysis.

**Results:** The results show that the increase of thermal insulation layer has the greatest impact on the temperature in the classroom when the doors and windows have gaps; Fresh air has the best effect on reducing CO2 concentration in the classroom, followed by fully open doors and windows; After strengthening the treatment of door and window gaps, closing the doors and windows and ventilating fresh air have the best effect on the control of PM2.5 concentration in the classroom.

**Conclusions:** The effect of continuous fresh air in reducing the concentration of indoor pollutants is better than that of natural ventilation. The internal thermal insulation setting can effectively ensure the indoor temperature. The research results can provide a basis for putting forward a reasonable classroom reconstruction plan, controlling the quality of indoor air environment, and providing a certain reference for creating a comfortable and healthy indoor thermal environment in public places and ensuring public health.

## LHMM23079 RESEARCH ON FINANCIAL PERSONNEL MENTAL HEALTH IN FINANCIAL DIGITAL TRANSFORMATION

Xiaocheng Gu^a^, Shanshan Zhou^a^

*^a^ Department of Accounting, Anhui Business & Technology College, Hefei, Anhui, China.*

**Background:** To verify that Power Business Intelligence (Power BI) tool is more efficient than traditional Excel tool in processing financial data through typical financial cases, and can improve the efficiency of financial personnel data processing. At the same time, using questionnaire survey method, this paper explores the impact of Power BI on the mental health of financial workers in Chinese enterprises from a micro perspective, which is helpful to understand the relationship between the development of financial digitalization and individual behavior at the micro level. To find out the psychological needs of financial workers, and make timely intervention and adjustment, effectively resolve the contradiction, to ensure the efficient and orderly progress of financial work.

**Subjects and Methods:** Excel and Power BI were used to solve financial problems in typical financial cases, and the complexity of the two calculation processes was compared and analyzed to show the high efficiency of Power BI tools in financial number processing. Through a questionnaire survey, we compared and analyzed the work and psychological impact of using Excel and using Power BI to solve financial problems, and discussed the impact of digitalization on financial practitioners from the perspective of psychology.

**Results:** Power BI tools also provide financial workers with more efficient choices in data processing. Our study answers the question of whether the development of financial digitization affects individual psychological health, which has important theoretical and practical significance. In addition, based on China, a typical developing country, the research results have important practical significance for the development of financial digitization in developing countries.

**Conclusions:** Financial digital transformation is an inevitable trend in the digital era. Power BI tool can improve the efficiency of financial workers’ data processing and is an important tool in digital transformation. However, the process of digital transformation will also bring certain psychological pressure to financial workers, including digital overload and cognitive load, which requires timely intervention and adjustment. At the same time, under the background of digitalization, financial personnel need to constantly learn and update their skills, adapt to the changes and challenges of the digital environment, and improve their competitiveness. In order to better participate in the financial business in the digital era.

### Acknowledgments

This work was supported by the provincial Quality Engineering Teaching and Research Project of Anhui Province [grant number 2021JYXM0161]; the provincial Quality Engineering Teaching and Research Project of Anhui Province [2022JYXM152]; Anhui Province Teaching and Research Project [2021XJJY19]; Anhui Province Teaching and Research Project [2019XJJXTD01].

## LHMM23080 STUDY ON MASS MENTAL HEALTH AND ADJUSTMENT AFTER THE EMERGENCY PUBLIC HEALTH EVENT: TARGETING PEOPLE AGED FROM 18-22

Junjie Zhang^a^, Tieliang Guo^b^, Qing Li^c^

*^a^ Academic Affairs Office, Wuzhou University, Guangxi 543002, China, ^b^ College of Electronics and Information Engineering, Wuzhou University, Guangxi 543002, China, ^c^ Psychological Counseling Center, Wuzhou University, Guangxi 543002, China.*

**Background:** Since the outbreak of new coronavirus pneumonia, it has attracted extensive international attention. In order to understand the impact of this public health emergency on the mental health of young people, The subjects of this survey are all young people aged 18-22..

**Subjects and Methods:** Through the network questionnaire survey, a total of 4305 students were surveyed, including 1222 boys and 3083 girls. This study adopts the self-made general situation questionnaire and the norm of symptom checklist (SCL-90) as well as interviews for factors.

**Results:**In the early and later stages of the epidemic, students’ emotions changed. Most of the students had a good psychological state, while a small number of students had bad emotions such as nervousness, fear and irritability. College students, SCL-90 the difference in the detection rate of symptoms of each factor was not statistically significant. The trend of the factors of different gender students was different before and after the epidemic, and the factors of different levels at different levels, and different data were obtained after the variance.

**Conclusions:** Through mental health testing of 18-22 year olds, comparative analysis of the gap between negative and positive effects, the differences in young people’s personal health adjustment in the face of public health emergencies are studied from the aspects of health philosophy, health knowledge and healthy lifestyle, Explore low-cost paths that can alleviate the lack of medical resources, release the pressure of the medical system, and promote the sustainable development of public health. According to research, since the outbreak of the epidemic, young people with a calm mind have mostly adopted art related adjustment methods. Through promotion, the test was conducted again among young people, and the influencing factors of some young people’s mental health decreased significantly. Therefore, it is suggested that such methods can be used for reference in the adjustment of public health after public health emergencies.

### Acknowledgments

The work was supported by the 2022 Guangxi Higher Education Undergraduate Teaching Reform Project (Grant No. 2022JGZ167), and the 2020 Guangxi Higher Education Undergraduate Teaching Reform Project (Grant No. 2020JGB369).

## LHMM23081 INVESTOR PSYCHOLOGICAL ANXIETY AND MARKET REACTION TO MANAGEMENT EARNINGS FORECASTS DURING THE CORONAVIRUS PANDEMIC

Xiaobei Huang^a^, Caixia Wu^a^

*^a^ School of Economics and Management, North China University of Technology, Beijing, 100144, China.*

**Purpose:** The purpose of this paper is to investigate whether investors who develop psychological anxiety will rely more on the management earnings forecast of Chinese listed firms during the coronavirus pandemic.

**Methods:** Based on the quarterly earnings forecasts of all A-share listed companies in China from 2018 to 2021, this paper examines the market reactions of investors to management earnings forecasts before and after the coronavirus pandemic using difference-in-difference methods.

**Results:** The results show that uncertainty due to the coronavirus pandemic can affect investors’ psychological expectations of the future economic situation and induce psychological anxiety, which will trigger investors’ vague aversion, the irrational sentiments lead to pessimistic judgment of investors on the future operation of listed companies, resulting in a significant decline in the market reaction to management earnings forecast during the pandemic. The quality of the management earnings forecast is further controlled in the regression model and the research conclusion remains unchanged, excluding the impacts of information quality on the research conclusion.

**Conclusions:** The coronavirus pandemic has caused unprecedented uncertainty, which also triggers psychological anxiety of investors and affects investment decisions. In the pandemic, investors have demonstrated ambiguity aversions, and less trust in earnings forecasts of corporate management. The results are not driven by the changes in the quality of management earnings forecasts. This research helps the market learn more about the behavioral logic of corporate management and the influence of investors’ decision-making during the pandemic, which can also provide evidence for regulators to further standardize the information disclosure of listed companies and improve the information environment of the Chinese capital market.

### Acknowledgement

The paper was supported by National Natural Science Foundation of China (71602004), the Social Science Foundation of Beijing (19GLC059).

## LHMM23082 THE VALUE OF CHINESE EXCELLENT TRADITIONAL CULTURE IN COLLEGE STUDENTS’ MENTAL HEALTH EDUCATION AND ITS REALIZATION

Qun Wang^a^

*^a^ University of Jinan, Jinan, Shandong 250022, China.*

**Background:** The excellent traditional culture of China is the inexhaustible motive force for the Chinese nation’s endless and sustainable development. Integrating the excellent traditional culture of China into the mental health education in colleges and universities, stimulating the vitality and vitality of the excellent traditional culture of China, and highlighting the value of the times are not only important measures to improve the effectiveness of mental health education for college students, but also important measures to inherit the national spirit blood, absorb rich historical and cultural nourishment, and enhance the national cultural soft power and international discourse power, It is a realistic need to build a socialist cultural power with Chinese characteristics and comprehensively improve the cultural literacy of the people. Given that there are still many problems with the excellent traditional Chinese culture in college students’ mental health education, we must strengthen efforts to promote the creative transformation and innovative development of the excellent traditional Chinese culture.

**Subjects and Methods:** Mental health education in colleges and universities is related to the major issue of cultivating qualified builders and successors of socialism with Chinese characteristics for the new era. We must take scientific and effective measures, adhere to the principle of making the past serve the present, bring forth the new, treat them differently, inherit the useful and discard the useless, draw essence from the excellent traditional Chinese culture, and strive to use all the spiritual wealth created by the Chinese nation to educate people with culture.

**Results:** Do a good job in the creative transformation and innovative development of Chinese excellent traditional culture in college mental health education, and build a harmonious campus culture by exploring the harmonious view of Chinese excellent traditional culture; Pay attention to the space concept of the excellent traditional Chinese culture and do a good job in its “three entry” work; Develop the dynamic view of Chinese excellent traditional culture, innovate teaching methods, and pay attention to educational effectiveness; Highlight the characteristics of Chinese excellent traditional culture and enrich the carrier of Chinese excellent traditional culture education; Strengthen the people-oriented view of Chinese excellent traditional culture and improve the teaching level of teachers; Adhere to the concept of knowledge and action of Chinese excellent traditional culture, and build a “trinity” education model of Chinese excellent traditional culture of school, society and family.

**Conclusions:** The excellent traditional Chinese culture is both historical and contemporary; It is both national and global. We should more consciously and actively promote the adaptation of the excellent traditional Chinese culture to the contemporary society and the coordination with the modernization process, better promote the creative transformation and innovative development, and promote the mental health education of college students.

### Acknowledgments

This paper was supported by a project from the foundation “ Research on the Integration of Excellent Traditional Chinese Culture into the Education and Teaching of Ideological and Political Theory Course”—2015 Shandong Provincial Social Science Planning Project (15cszj04).

## LHMM23083 A HOLISTIC PERSPECTIVE-BASED UNDERSTANDING OF TRANSCORTICAL MOTOR APHASIA: THREE-MODULE MODEL

Yinchou Zhou^a^, Tao Xiao^b^, Meihong Zheng^c^

*^a^ Department of Foreign Languages and Literatures, Tsinghua University, Beijing 100084, China, ^b^ Institute of Industrial Development and Governance Innovation, Zhejiang Guangsha Vocational and Technical University of Construction, Jinhua, 322103, Zhejiang, China, ^c^ School of Social Sciences, Tsinghua University, Beijing 100084, China.*

**Background:** Current treatments of transcortical motor aphasia are based on the attribution of typical symptoms and brain regions. However, the large individual differences between patients and the uncertainty of stroke injury areas make it difficult to adapt to the dynamic changes of post-stroke aphasia recovery. In order to make the symptom analysis of transcortical motor aphasia more dynamic and better fit for patients, this study attempts to reexamine the specific symptom representations with the perspective of a holistic process from verbal intent to speech behavior.

**Subjects and Methods:** Based on the neural basis of language processing as well as the results of the PDC model and P-chain model, we have proposed a three-module model consisting of linguistic expression, coordinated connectivity, and motor output (labeled as part 1, part 2, and part 3 respectively)——after generating the expressive intention in part 1 and then projects the expressive intention and content to the motor control system in part 3 through the coordination of part 2 and the motor output of the language after integrating the articulatory organs, thus completing the complete language expression.

**Results:** Language expression can be understood as a process from intention to behavior, in which “language production” at the beginning and “motor output” at the end of the process are linked in a loop, while the aSPPO area is specific for speech-motor connectivity and there is overlap with brain areas damaged in patients with transcortical motor aphasia. Thus transcortical motor aphasia can be at least partially explained by the fact that disruptions in the coordinated connectivity pathway involving the aSPPO area may lead to the inability of individuals to produce effective language output even when the speech production and vocalization sites are functional.

**Conclusions:** Based on brain plasticity and the fact that the aSPPO area is between the hand action area and the oral-facial action area, there may be practical value in two behavioral intervention treatment options for transcortical motor aphasia: assistance in functional overlap areas, which means after training individuals to conceptualize verbal content in the brain (without expressing it) while performing non-verbal actions such as licking their lips and smiling, the originally damaged coordination connectivity pathways involved in aSPPO can be reactivated to some extent; and compensation in adjacent areas, referring to individuals who perform right-handed hand action or facial action training will have significantly improved verbal motor output.

## LHMM23084 A STUDY ON THE INFLUENCE OF ACADEMIC PERFORMANCE ON THE MENTAL HEALTH OF CHINESE LIBERAL ARTS STUDENTS

Xiaoxue Zhang^a^, Shizheng Li^b^

*^a^ School of Management Science and Engineering, Anhui University of Finance and Economics, Bengbu 233030, China, ^b^ School of Finance, Anhui University of Finance and Economics, Bengbu 233030, China.*

**Background:** Despite the multitude of studies exploring the causes of mental health problems among college students in China, the impact of academic performance on mental health has not been adequately investigated. Purpose: Analyzing the relationship between student self-evaluation comprehensive academic performance score, as well as their scores on 7 different sub-dimensions of academic performance, and the mental health status of college students.

**Subjects and Methods:** A 41-item scale was developed to evaluate participants’ academic performance. 10 items from the PSS-14 were used to measure the mental health status of participants. Descriptive statistics were initially employed to analyze the distribution and numerical characteristics of academic performance and perceived stress among the participants. Seven logistic regression models were constructed to investigate the predictive effect of different dimensions of academic performance on the mental health.

**Results:** Online teaching prompted by the COVID-19 pandemic did not yield a significant impact on the mental health of the respondents. Gender did not emerge as a predictive factor for mental health status. Stress related to academic performance is not limited to students who struggle with their studies, as academic achievers may face higher risks. Academic performance scores obtained through the self-evaluation scale demonstrated better predictive power for mental health status than traditional GPA scores.

**Conclusions:** The threshold for safeguarding the mental health of college students must be shifted to an earlier stage of education. It is imperative for China to pursue a gradual and sequenced approach to the implementation of mental health education in primary, secondary, and tertiary schools, with a focus on enhancing the professionalism and expertise of mental health education practitioners.

### Acknowledgments

This work is supported by Anhui University of Finance and Economics’ Teaching Research Project (acjyzd2020004).

## LHMM23085 EXPRESSION OF GABAA RECEPTOR SUBUNITS A1 AND A2 IN AUTISM

Linyin Luo^a^, Weiqing Zhao^a^, Ye Liu^b^, Yaozhou Wang^a^, Caiyun Xiong^a^, Hao Zhou^a^

*^a^ Department of Pediatrics, Guizhou Provincial People’s Hospital, Guiyang, 550002, China, ^b^ Department of Otolaryngology, Guizhou Provincial People’s Hospital, Guiyang, 550002, China.*

LL and WZ contributed equally to this research.

**Background:** This study examined changes in γ-aminobutyric acid type A (GABAA) α1 (GABAAα1) and α2 (GABAAα2) receptor expression in autism model rodents and children with autism spectrum disorders (ASD) with the aim of providing insights about causes and treatment of autism.

**Subjects and Methods:** Neuronal expression of GABAAα1 and GABAAα2 in the frontal cortex of control rats (saline) and gestational valproic acid (VPA)-induced ASD rats was measured by immunohistochemistry (IHC), western blot, and real-time quantitative PCR (RT-qPCR). Serum GABAAα1 and GABAAα2 expression in 37 autistic children and 32 healthy children were determined by enzyme-linked immunosorbent assay (ELISA).

**Results:** Immunohistochemical staining showed that the VPA-induced ASD rats had significantly higher mean GABAA α1 (0.023 ± 0.012 vs. 0.000 ± 0.000, P<0.05) and GABAAα2 (0.036 ± 0.018 vs. 0.013 ± 0.01, P<0.05) expression than controls. Western blot detection showed that the relative grayscale values of GABAAα1 (0.52 ± 0.05 vs. 0.44 ± 0.04, P>0.05) and GABAAα2 (0.79 ± 0.028 vs. 0.75 ± 0.97, P>0.05) protein were slightly higher in the VPA group than in controls, although the differences were not statistically significant. The VPA group also had higher GABAAα1 (P>0.05) and GABAAα2 (P<0.05) mRNA expression in the frontal cortex compared to controls. Similar to the findings in rodent models, autistic children had higher serum GABAAα1 (11.15 ± 3.19 pg/ml vs. 12.40 ± 4.34 pg/ml, P>0.05) and GABAAα2 (1183.48 ± 374.17 pg/ml vs. 1306.07 ± 326.04 pg/ml, P<0.05) levels than controls.

**Conclusions:** Abnormal expression of GABAA receptor subunits may contribute to autism pathogenesis.

## LHMM23086 MACHINE LEARNING APPROACH FOR SCREENING KEY GENES OF ATHEROSCLEROSIS AND PERFORMING PAN-CANCER ANALYSIS

Zhenrun Zhan^a,b^, Xiaodan Bi^a,b^, Xingqiang Huang^a,b^, Xu Tang^a,b^, Jinpeng Yang^a,b^, Tingting Zhao^a,b^

*^a^ Heping Hospital Affiliated to Changzhi Medical College, Changzhi, Shanxi 046000, China, ^b^ Changzhi Medical College, Changzhi, Shanxi 046000, China*

**Background:** Atherosclerosis, an immunoinflammatory disease caused by lipids, is a significant factor in coronary heart disease and stroke. Researchers worldwide are working to develop more effective ways of diagnosing and treating it. This article introduces a machine-learning algorithm that screens biomarkers and performs pan-cancer analysis as a reference.

**Subjects and Methods:** We first downloaded the GEO dataset containing information on atherosclerotic patients for differential gene screening and analyzed differential mRNAs using KEGG and GO enrichment analysis. Then, we combined WGCNA, protein interaction network analysis, and mechanistic learning algorithms to screen for core genes, perform basic experimental validation, and immuno-infiltration analysis. Finally, we used TCGA and GTEx databases for pan-carcinogenic analysis of differentially expressed genes.

**Results:** After screening differentially expressed genes from the GSE28829 dataset, we performed WGCNA and obtained 122 key genes by combining module genes and differential expression genes. We used four machine learning algorithms (XGBoost, RandomForest, SVM-REF, and GLM) to calculate the critical genes and obtain the junction of the results, which led us to identify four hub genes (SLAMF8, TLR2, VAMP8, and VSIG4). Next, we built a diagnostic model and assessed its capabilities. The ROC curves of the four genes suggested their critical role in the development of atherosclerosis. We then performed gene correlation analysis, immune infiltration analysis, and RT-PCR verification of the four core genes and finally screened out TLR2 for pancarcinoma. Our analysis found that TLR2’s expression in patients with various tumors differed from that in healthy individuals, and it was strongly associated with the prognosis of patients with diverse cancers.

**Conclusions:** TLR2 may be a target for intervention in developing diseases such as atherosclerosis and tumors.

### Acknowledgments

This study was supported by the Shanxi Province Graduate Education Innovation Project (2022Y726/737), the Basic Research Program of Shanxi Province (202203021212010), and the Youth Start-up Fund of Heping Hospital affiliated to Changzhi Medical College (HPYJ202225).

## LHMM23087 RESEARCH ON THE EVALUATION OF GRAIN PRODUCTION EFFICIENCY IN CHINA AND SOCIAL ANXIETY BASED ON FUZZY SUPER-EFFICENCY DEA AND BOOTSTRAP DEA METHODS

Yongcai Zhang^a^, Zhong Li^b^, Weibai Liu^c^

*^a^ School of Law and Business, Shaoyang University, Shaoyang 422000, China, ^b^ School of Business Administration, Hunan University of Finance and Economics, Changsha 410205, China, ^c^ School of Economics and Management, Hunan University of Science and Technology, Yongzhou 425199, China.*

**Background:** Food satisfaction can solve the problem of hunger, alleviate social anxiety and improve people’s mental health. Food is an important source of food, Food security is the most important ballast for economic development and social stability. Therefore, the social status of food is decisive and its yield and quality is particularly important. Food security lies not only in output growth, structural adjustment and optimization, but more importantly in further improvement of grain productivity and alleviate social anxiety.

**Methods:** Fuzzy logic theory, super-efficiency DEA and Bootstrap DEA methods are used to measure the grain productivity of China from 1978 to 2018. Fuzzy logic adopts “Fuzzy ToolkitUoN” to make a fuzzy logic control, with its implicit boundaries of the qualitative knowledge and experience, the aid of concept of subordinate degree function, fuzzy set and relation, the fuzzy logic can simulate the human brain implementation of rule-based reasoning, deal with the uncertainties created by logic deficiency of “law of excluded middle. The super-efficiency DEA model includes several models such as CCR, BCC and SBM, which is to make the same type of unit (called decision making units) evaluation based on the decision making unit of “input” and “output” data. Bootstrap-DEA method is to introduce Bootstrap into DEA model. Bootstrap sample data are constructed repeatedly through the method.

**Results:** The above data indicate that the traditional DEA method has defects, and more accurate super efficiency DEA and Bootstrap-DEA are needed to calculate the efficiency. Show that the grain yield efficiency is closely related to national policies and social development. China’s grain productivity is relatively stable on the whole, but the return to scale fluctuates greatly, and the return to scale even decreased for 13 consecutive years. Therefore, how to improve the production of food and its productivity in scientific ways to alleviate social anxiety. Since 2013, the comprehensive efficiency has been on a steady rise, and the efficiency value gradually increased from 0.8370 to 1. In addition, its technical efficiency has been stable with an efficiency of 1 for four consecutive years since 2015, indicating that technological progress has promoted the improvement of comprehensive efficiency. At the same time, the scale efficiency value is also above 0.9, and the comprehensive effect of technological progress and scale efficiency got a higher comprehensive efficiency value, improve people’s mental health.

**Conclusions:** To ensure the sustainable development and food security of China’s grain industry and alleviate social anxiety, it is necessary to strictly observe the minimized reserve of cultivated land, strengthen the efforts for the protection of cultivated land, establish and improve policies and incentive mechanisms for the introduction of agricultural scientific and technological personnel, strengthen efforts to make grain more market-oriented, and promote the integrated development of the grain industry with the secondary and tertiary industries. The efficiency of food production will be improved and when society is active, improve people’s mental health, maintain family and social security.

### Acknowledgements

Research and Planning Fund for Humanities and Social Sciences of the Ministry of Education of China (22YJA790038);Projects of Natural Science Foundation of Hunan Province, China (2020JJ5122, 2021JJ30069, 2021JJ30295, 2021J30635, 2019JJ50557); Project of Education Department of Hunan Province, China (19A445, 21B0733).

## LHMM23088 YANGTZE RIVER CULTURAL FESTIVAL ACTIVITIES FROM THE PERSPECTIVE OF URBAN CULTURAL SUPPLY AND SOCIAL PSYCHOLOGICAL HEALTH RESEARCH

Mingzhou Wang^a^

*^a^ Shanghai Urban Construction Vocational College, Shanghai, 200438, China.*

**Background:** Cultural festivals and events refer to a series of cultural activities or events held in cities, which have strong local, cultural, concentrated, and interesting characteristics. They can meet the psychological needs of citizens, enhance their sense of happiness, help alleviate social anxiety, and promote social stability and harmony. By analyzing and summarizing the key factors that can successfully host world-renowned cultural festivals, this paper proposes innovative measures to host the Yangtze River Cultural Festival.

**Subjects and Methods:** Using literature review and data collection methods, we selected world-renowned cultural festivals such as the London Mayor’s Thames Riverside Festival, Seoul’s “Han River Celebration”, and Dalian International Beach Cultural Festival as research objects to analyze the positive effects of cultural festivals on citizens and tourists, such as joy of participation, stress relief, relaxation, and psychological pleasure, and discussed creativity, brand The role of participants and other factors in cultural festival activities.

**Results:** Currently, with the rapid development of society, the pace of life has significantly accelerated, and with the rapid growth of the economy, there are also tremendous negative emotions such as stress, anxiety, and depression, which pose a great threat to people’s psychological and physical health. By participating in cultural festivals and experiencing lively and interesting activities, citizens can gain more opportunities for interpersonal communication, increase individual social interaction, resist negative emotional influences, eliminate depression and anxiety, and promote mental health. A successful cultural festival event must rely on the city’s existing resource endowment, use innovative thinking for top-level design, pay special attention to setting up distinctive and interesting activities, mobilize the participation of diverse subjects, especially young people, and actively meet their daily entertainment and mental health needs. In addition, it is necessary to create a market-oriented, government assisted operating mechanism.

**Conclusions:** As one of the cultural supply products in cities, cultural festivals and events have a positive impact on enriching citizens’ daily cultural life and promoting their mental health. Research has shown that participating in cultural events can help people maintain good interpersonal relationships and self understanding, maintain a positive mental state, and effectively reduce the incidence of mental illness. Cities along the Yangtze River have rich resources to carry out Yangtze River cultural festivals. By learning from the successful experience of world-renowned cultural festivals and holding colorful Yangtze River cultural festivals, it is possible to explore a new path for urban cultural supply.

## LHMM23089 RESEARCH ON THE MECHANISM OF CULTURAL SYMBOL COGNITION IN TOURIST AREAS TO ALLEVIATE TOURISTS’ PSYCHOLOGICAL ANXIETY AND IMPROVE TOURISM WILLINGNESS

Yongping Li^a,b^, Xiaodong Zheng^a,c^, Miao Wang^a^, Fan Zhang^a^

*^a^ Faculty of Humanities and Arts, Macau University of Science and Technology, Macau 999078, China, ^b^ Ningbo University of Finance and Economics, Ningbo, Zhejiang 315175, China, ^c^ School of Computer and Information Engineering, Nantong Institute of Technology, Nantong, Jiangsu 226002, China.*

**Background:** Under the background of consumption era, more and more tourist areas begin to create unique cultural symbols as an important basis for their market attraction and brand effect. However, in the past few years, influenced by COVID-19, people are generally anxious and have no strong desire to travel, which leads to a sharp drop in tourist flow in scenic spots, a continuous downturn in tourism consumption, and a huge impact on tourism. With the gradual disappearance of the epidemic, the global tourism industry has regained its confidence and gained great opportunities. How to accurately grasp and construct cultural symbols, so as to alleviate tourists’ anxiety and enhance tourists’ willingness to travel, has become an important proposition to be solved urgently in every tourist area.

**Subjects and Methods:** Taking Putuo Mountain Tourist Area in Zhoushan City, Zhejiang Province as the research object, this paper discusses the relationship between cultural symbol cognition, tourism psychology and tourism willingness, aiming at clarifying the difficulties faced by Putuo Mountain Tourist Area, helping to establish and strengthen its unique cultural symbols, thus effectively improving the popularity and passenger flow of the tourist area. Firstly, based on the theory of symbolic interaction and symbolic consumption, the corresponding question hypothesis and questionnaire are put forward. Secondly, the questionnaire survey was carried out through the network platform, and the questionnaire results were processed and analyzed by SPSS software. Finally, the corresponding results are obtained by regression analysis to verify the hypothesis.

**Results:** A total of 1000 questionnaires were distributed and 833 questionnaires were collected. After eliminating 172 invalid questionnaires, 661 valid questionnaires were obtained. Using SPSS software to process and analyze the questionnaire data, the results show that tourists’ cognition of Putuo Mountain cultural symbol is positively correlated with their willingness to travel, and the degree of tourists’ psychological anxiety is also positively correlated with their willingness to travel. In addition, the analysis results show that personal education level, personal age, personal income level, image perception of tourist destination and hardware facilities are also important factors affecting tourism willingness.

**Conclusions:** Zhoushan is the main maritime gateway for China to open to the outside world, and it is the only place for Chinese and foreign ships to sail from south to north. There are unique mountain and sea landscapes and island fishing customs here, and tourism has become one of the local pillar industries. As an important scenic spot in Zhoushan, Putuo Mountain attracts a large number of tourists every year with its beautiful scenery and unique cultural heritage. In the future, Putuo Mountain must vigorously publicize its unique cultural symbols, enhance its cultural heritage and speed up the construction of tourism infrastructure to maintain its sustainable attraction as a tourist destination.

### Acknowledgements

This paper is supported by 2021 Nantong Science and Technology Plan Project (JCZ21074), 2020 Jiangsu University Philosophy and Social Science Research Project (2020SJA1612), Young and Middle-Aged Key Teachers Training (Scientific Research) Project of Nantong Institute of Technology (ZQNGGJS202120), Key Scientific Research Project of Zhejiang Provincial Department of Culture and Tourism (2021KYZ006), Key Project of Ningbo Education Science Planning (2021YZD019).

## LHMM23090 STUDY ON THE MAIN CAUSE, FORMATION MECHANISM AND PREVENTION COUNTERMEASURES OF HYPERURICEMIA IN CHINESE RESIDENTS FROM THE PERSPECTIVE OF NUTRITIONAL MEDICINE

Jianping Liu^a,b^, Yongtian Wu^b^, Zhiheng Chen^c^, Xuekai Wei^b^

*^a^ College of Civil and Environmental Engineering, Hunan University of Science and Engineering, Yongzhou, Hunan, 425199, China, ^b^ Bama Hetai Longevity Industry Co., Ltd., Bama, Guangxi, 547500, China, ^c^ Department of Health Management, Xiangya Third Hospital, Central South University, Changsha, Hunan 425099, China.*

**Background:** The high incidence of hyperuricemia has become a major medical and social problem affecting the health of Chinese residents. The existing medical system has not yet explored the formation mechanism of hyperuricemia, resulting in little effect on the prevention and treatment of hyperuricemia.

**Subjects and Methods:** From the perspective of nutritional medicine, the development and changes of nutrient intake and dietary preferences of Chinese residents in the past three decades have been analyzed in depth by using the time series analysis method, and the formation process and mechanism of hyperuricemia have been systematically studied by using the whole-cycle theory.

**Results:** The research shows that excessive uric acid production and too little uric acid excretion are the two causes of high incidence of hyperuricemia. The excessive production of uric acid is caused by the preference for high-purine food, the structural nutrient intake imbalance caused by the overall insufficient intake, the excess intake of some nutrients and the uneven intake of nutrients of long-term nutrients, and the metabolic abnormalities of human related enzymes. Too little uric acid excretion is caused by the structural imbalance of long-term nutrient intake, which is the main cause of hyperuricemia.

**Conclusions:** The basic mechanism of hyperuricemia is the excessive re-absorption of kidney caused by the long-term imbalance of nutrients intake, especially the insufficient intake of water nutrients. We can effectively prevent and treat hyperuricemia by controlling the intake of high-purine exogenous foods, balanced intake of daily nutrients, taking disease-based strategies, strengthening daily independent health management and other measures. The research provides a new theoretical perspective and specific implementation methods for the prevention and treatment of hyperuricemia in China, which has great theoretical value and practical significance.

### Acknowledgments

This work was supported by a project grant from Natural Science Foundation of Hunan Province (Grant No.2019JJ50915) and Hunan University of Science and Technology Applied Characteristic Discipline Construction Project (Grant No.18TSXK-001).

## LHMM23091 RESEARCH ON THE PSYCHOLOGICAL MINING AND MENTAL HEALTH OF THE HERO IN THE CHARACTER DOCUMENTARY “A FEMALE ANCHOR WHO SELLS GOODS THROUGH LIVE STREAMING”

Huansong Yang^a^, Youyou Zhang^a^, Yuehan Qiao^a^

*^a^ Hangzhou Normal University, Hangzhou City, Zhejiang Province 310000, China.*

**Background:** According to the 2019 Chinese Women’s Confidence Report, 40 percent of Chinese women underestimate themselves. In other words, two out of every five Chinese women are not confident. A survey of 2,000 women by a UK institute found that 42 percent of women never give themselves a good compliment and criticize themselves at least eight times a day. This paper takes the method of psychological mining and performance of the hero of character documentary as the research content, explores the influence and contribution of streaming media anchor, a new profession, on the development of female mental health, and strives to find out a set of methods of psychological mining and performance of documentary characters.

**Methods:** Based on psychological principles such as threshold effect, seesaw law, congruence effect, external behavior and manner of characters reflecting internal psychology, and research methods, this paper explores the psychological mining methods of film and television character documentaries, and explores the positive influence of the rise of “a female anchor who sells goods through live streaming” on women’s mental health according to specific character interviews and surveys. The research on the psychology of the protagonist in the character documentary of “a female anchor who sells goods through live streaming” can provide experience and reference for the creation of character documentary in the future, so as to better shape the character image in the documentary creation.

**Results:** The research shows that: 1. In professional documentaries, the use of audio-visual language can excavate the psychological changes of characters to a certain extent. For example, the use of long lens and depth of field lens can capture and amplify the psychological and emotional changes of characters.2.the color tone of the picture, simultaneous sound processing and music sound can play a key role in the portrayal of the character image and character psychology.3. The rise of “a female anchor who sells goods through live streaming” has greatly enhanced women’s self-confidence and has a positive impact on women’s mental health.4. As women have found broader career paths, their social status and recognition have also increased along with their economic level, which has a significant effect on their mental health.

**Conclusions:** With the development of the e-commerce industry, a large number of women have found their own value in the work, thus blooming the charm and brilliance of different age groups. Influencing and inspiring others while maximizing their own worth is part of the magic that careers confer on women. Through the psychological mining and mental health research of the heroine of “a female anchor who sells goods through live streaming”, the author hopes that in the future career development and career planning, there will be more emerging industries to help women realize their self-worth.

### Acknowledgements

This research was supported by Fund program: Zhejiang Province digital cultural and creative Industry Production and education integration engineering base project (Zhejiang development reform society [2020] No.319)

## LHMM23092 STUDY ON THE UNIVERSAL DESIGN OF COUNTRY PARK RECREATION FACILITIES BASED ON THE PSYCHOLOGICAL NEEDS OF USERS

Hongqi Zhou^a^, Li Li^b^

*^a^ Xianda College of Economics and Humanities, Shanghai International Studies University, Shanghai 200000, China, ^b^ Hunan University of Technology, Zhuzhou, Hunan 412000, China.*

**Background:** This study focuses on the behavioral and psychological needs of users and advocates the use of universal design concepts to improve the design of open space facilities in country parks, so as to better reflect the “people-oriented” purpose of design and meet the needs of users for respect, comfort, equality and care. The research provides reference for the design of public space facilities.

**Subjects and Methods:** Firstly, through literature research method, the basic theories related to the study were summarized. Secondly, through on-site investigation, the current status of recreational facilities was understood, and the needs of users’ physiological and psychological fairness, respect, safety and other characteristics were understood. Finally, through investigation and analysis, strategies for the universal design of recreational facilities in the countryside park were proposed.

**Results:** According to the characteristics of country parks, the physical and psychological needs of users, the design of country park recreation facilities should follow the principles of fairness, systemization, safety, comfort and aesthetics, and embody the “people-oriented” design purpose, so that urban recreation facilities can better meet the needs of everyone.

**Conclusions:** Recreational facilities are an important part of the recreational space in the countryside park, and their design should fully respect users’ behaviors and psychological needs. The design should aim to provide equal, safe, and comfortable universal functions for as many users as possible, effectively reducing conflicts between environmental facilities and individual characteristics. This approach can better embody social care and equity.

## LHMM23093 A PREOPERATIVE NOMOGRAM TO PREDICT EARLY DEATH AFTER SURGERY IN PATIENTS WITH RESECTABLE HEPATOCELLULAR CARCINOMA

Yabing Shan^a^, Yan Lu^a^

*^a^ The Third Affiliated Hospital of Zhejiang Chinese Medicine University, Hangzhou, China.*

**Background:** We aim to develop a preoperative nomogram to evaluate the risk of death within a year of the operation in patients with resectable hepatocellular carcinoma.

**Subjects and Methods:** Retrospective study of 490 hepatocellular carcinoma patients who were treated with hepatectomy. Multiple logistic regression analysis was performed to identify factors associated with early death in these patients. A nomogram was constructed based on the independent risk factors, and predictive accuracy of nomogram was valued.

**Results:** Tumor size, portal vein tumor thrombus, fibrinogen to albumin ratio and geriatric nutritional risk index were found to be independent predictors of death at 1 year after surgery in resectable hepatocellular carcinoma patients and were finally entered into the nomogram. The calibration curves for the possibility of one year mortality showed optimal agreement between nomogram prediction and actual observation. The concordance index and the area under curve of receiver-operating characteristic showed that the nomogram in current study was superior to the Barcelona Clinic Liver Cancer classiﬁcation, Child-Pugh score, and Okuda stage in predicting early death of hepatocellular carcinoma patients with sugery.

**Conclusions:** Our nomogram got a better accuracy in predicting mortality within a year after operation in patients with hepatocellular carcinoma.

### Acknowledgments

The authors disclose the receipt of financial support for the research, authorship, and/or publication of this article from the Planned project of Zhejiang Administration of Traditional Chinese Medicine approved by Zhejiang Provincial Health Commission (2011ZB059).

## LHMM23094 INTERPRETATION OF CHARACTERIZATION IN O’CONNOR’S NOVELS BASED ON THE THEORY OF JUNG’S PSYCHOLOGICAL PERSONA

Zhenfeng Xiao^a^, Wenhui Zhang^a^, Jin Xu^a^, Yiwen Qi^a^

*^a^ Tianjin University, Tianjin 300350, China.*

**Background:** Flannery O’Connor is a representative writer of postmodernism in the American Southern literary genre. She lived at a time when the American South was in the midst of intense turmoil, with the rise of the feminist movement, the rapid expansion of the capitalist economy, increasing social conflicts, and serious distortions in human relationships and the inner spirit of man. With her own unique life experience and keen insight into the darkness of human nature, O’Connor was deeply influenced by Jung and consciously used the psychological perspective in her creation to penetrate the hidden inner world of the characters and present their true spiritual state, shaping a group of characters who abandoned their faith and had deformed personalities. The externalized expression of individual thoughts and mental activities is a direct mapping of character. In modern society, the reason why human beings live in an orderly and harmonious society lies in the regulation of law and ethics, while in the American South at the early stage of development and even in the period of seclusion, the root of the stability of social relations lies in the internal control of individuals, i.e., self-control, and psychological health and personality perfection are the basic conditions for the existence of self-control. This paper analyzes the O’Connor series of novels from a psychological perspective. The purpose of this article is to study the results of the influence of psychological and external factors on the development of individuals and society.

**Subjects and Methods:** Psychological analysis is not only a method of literary criticism, but also a way and perspective to see the world. Based on Jung’s personality theory, this paper takes five short stories of O’Connor: A Good Man is Hard to Find, Good Country Man, Greenleaf, and To Save a Man is to Save Himself as the objects of textual research, aiming to analyze the characters in O’Connor’s novels from a psychological perspective through a close reading of the text, with the intention of studying the influence of psychological factors and external factors on personal development and even social. O’Connor draws a rich and diverse portrait of a monstrous group of characters in his short stories. In the development of the characters’ personalities, the external personality masks play a key role. Through the external personality masks, the distorted life caused by the unbalanced mask and shadow of the main character is analyzed, thus revealing the indifference and alienation between people in a sick society.

**Results:** Jung’s theory of analytical psychology explains the madness hidden in the depths of the collective unconscious and reveals the complexity and universality of human psychological contradictions. The purpose of this study is to examine the influence of psychological factors and external factors on individual development and even on society. From the outside to the inside, the potential implications are explored to reveal the rich connotation and great tension of the characters in O’Connor’s novel under Jung’s archetypal theory. O’Connor’s unique artistic style, her use of dynamic language, the expression of the grotesque and death, gives the novel a different kind of aesthetic interest. At the same time, the alienated society and the radical changes of the times make us see the goodness, evil, beauty and ugliness of human nature through the archetypal characters, and understand the fate of the characters, while more than anything else, it arouses our concern for life, our desire for the sublime, and our persistent pursuit of faith.

**Conclusions:** In human history, society is often in a state of constant change, and with it, the transformation of human psychology, in other words, the course of human psychological development is closely related to the continuous development of society. In other words, the course of human psychological development is closely related to the continuous development and change of society, which is a huge collective of individuals with different personalities and psychological states. The society is a huge collective of individuals with different personalities and psychological states, and being in the collective, the universal or majority will is regarded as the unified goal and premise of development in the society. The intersection of psychology and literature is adapted to the needs of the times. Understanding a work is not only limited to knowing people, but also based on understanding the psychology of the writer, in order to better enter into the main character of the work. O’Connor was deeply influenced by Jung, and looking at O’Connor from the perspective of Jungian analytical psychology undoubtedly opens a new door for us to know O’Connor and her novels.

### Acknowledgments

This work is supported in part by the Tianjin Philosophy and Social Sciences Research Program Funding Project under Grant TJWW21-006.

## LHMM23095 MONKEYPOX WARNING AND VISUALIZATION SYSTEM BASED ON LSTM

Wei Liu^a^, Zhenzhao Wang^a^, Jing Su^a^

*^a^ School of Mathematics and Computer Science, Guangdong Ocean University, Zhanjiang, Guangdong, China.*

**Background:** As of September 17, 2022, the World Health Organization has reported 59147 laboratory confirmed cases of monkeypox virus and 489 possible cases, including 22 deaths. The rampant spread of the monkeypox virus poses a grave threat to the lives and well-being of people worldwide. In order to reduce the large-scale spread of monkeypox in the world and reduce the impact of monkeypox on the public health system and international trade economy, it is essential to timely warn monkeypox through artificial intelligence.

**Subjects and Methods:** We integrate 131 days of monkeypox infection data (May 7, 2022 to September 14,2022) into the Susceptible- Exposed-Infection-Removed (SEIR) model and the Long Short Term memory (LSTM) model to obtain the epidemic curve. We also propose a four-tier architecture system that can visualize data and predict epidemic situation. The system submits the predicted data to the module within the corresponding system to form a clear and concise visual page, which is displayed on the IOT device.

**Results:** We compare the prediction performance of SEIR and LSTM models for monkeypox virus, and found that LSTM neural network performs well in the prediction of monkeypox epidemic. The LSTM model used by the ten countries most affected by the epidemic has good fitting results (R2=0.995155, RMSE=33.098713). We predict the United States, Spain, Brazil, France, the United Kingdom, Germany, Peru, Canada, Colombia and the Netherlands, the top 10 countries in the list of infections. The goodness of fit R2 of several countries exceeded 0.99 (United States of America 0.991251, Brazil 0.994753, Peru 0.997037). According to the model, the threat of monkeypox virus will basically end in December 2022, while the United States will end early around November 19, 2022 (in the LSTM model).

**Conclusions:** This paper proposes a monkeypox virus early warning and visualization system based on LSTM neural network. The results show that it is feasible to use LSTM neural network to predict monkeypox epidemic. The visualization system can assist decision makers to make decisions and minimize the harm of infectious diseases, which is worthy of our promotion.

## LHMM23096 INFLUENCE OF COUNTER-STEREOTYPE INFORMATION ON REGIONAL STEREOTYPES: A MENTAL HEALTH PERSPECTIVE

Liuliu Sheng^a^, Jun Ren^b^

*^a^ Taizhou Vocational and Technical College, Zhejiang 318000, China, ^b^ Zhejiang Normal University, Zhejiang 321000, China.*

**Background:** With the development of the network society and the wide spread of negative cases, some regions have been labeled as “stigmatized”, and regional stigma has been constantly constructed, thus forming a negative stereotype of the region. Negative stereotypes not only affect people’s mental health, but also lead to the formation of bad coping styles, thereby affecting their social functions. To explore whether regional stereotypes exist in North and South China and what characteristics they have at the explicit and implicit levels. Subjects were randomly selected from the southern population and the northern population, with the Qinling-Huaihe Line as the boundary.

**Methods:** In the process of research, it is necessary to pay special attention to the mental health of the subjects. The existence of regional stereotype may induce the psychological stress and anxiety of the subjects, thus affecting their emotional and behavioral performance. Therefore, it is necessary to give full informed consent in advance, introduce the main content and purpose of the experiment to the subjects, and inform them that they have the right to refuse to participate in the experiment without any punishment, gain or loss. It is necessary to pay full attention to and protect the mental health of the subjects in the process of research to ensure the scientific and reliability of the experimental results. In the whole process of the study, free response method and trait corresponding behavior were used to collect questionnaires to verify the existence of regional stereotypes. Then explicit regional stereotypes were measured by behavior rating questionnaire and implicit regional stereotypes were measured by stereotype explanatory bias questionnaire.

**Results:** The measurement, collection and analysis of regional stereotypes can help people understand their own psychological conditions, including emotions, thinking, behavior and other aspects, and identify the factors causing negative regional stereotypes, so as to take corresponding actions, measures and interventions to improve their mental health. Among the findings: (1) At the explicit level, the subjects had negative regional stereotypes. The main effect of gender on negative stereotypes was not significant, while the main effect of household registration was significant. The interaction between gender and household registration was not significant. Both Southern and Northern subjects showed in-group preference and out-group rejection. (2) At the implicit level, the subjects had negative regional stereotypes. The main effects of gender and household registration were not significant, and the interaction was also not significant. Both Southern and Northern subjects showed more attributions and external attributions for inconsistent situations, and fewer attributions and internal attributions for consistent situations. (3) Presenting a larger amount of counter-stereotype information can reduce negative stereotypes of the target group more effectively. The research results support the Thin Slice Model. Presenting moderately abnormal counter-stereotype information can reduce negative stereotypes of the target group more effectively than presenting extremely abnormal counter-stereotype information, which is inconsistent with the content of the Transformation Model. Presenting moderately abnormal and a larger amount of counter-stereotype information is most effective in reducing negative regional stereotypes.

**Conclusions:** At the explicit and implicit levels, regional stereotypes exist in both the north and south groups in China, showing the characteristics of in-group preference and out-group rejection. Medium anomaly and large amount of counter-stereotype information can suppress negative regional stereotypes more effectively. In the future, while applying the research results to promote social harmony and stability, it is necessary to pay attention to and protect the mental health, and adopt reasonable means and methods to reduce the negative emotional experience of the subjects such as anxiety and pressure. For example, researchers can use psychological intervention methods to stimulate positive emotions, guide cognitive reconstruction, relieve stress and anxiety during the experiment to help subjects adjust their mental state and mental health, and promote their positive emotions and physical and mental health. In addition, the results show that the medium anomaly and large amount of counter-stereotype information can effectively suppress the negative regional stereotype. Therefore, in real life, relevant departments and individuals can adopt some scientific and rational means and measures to encourage diversification and promote exchanges and cooperation, present moderate abnormal and large amount of positive information, reduce the negative impact of regional stereotypes, and promote the harmonious and stable development of society. In conclusion, while promoting the application of research results, we need to pay full attention to and protect the mental health of the participants, promote social stability and people’s happiness, and achieve the goal of healthy, harmonious and sustainable development.

### Acknowledgements

This work was supported by the [2022 Professional Development Project for Domestic Visiting Scholars and Teachers in Colleges and Universities] under Grant [number: FX2022130], and by the Taizhou Aien Kangze Health Management Co., Ltd under Grant [number: HX2022237].

## LHMM23097 ANALYSIS OF BLOOD TRANSFUSION-RELATED INFECTIOUS DISEASE MARKER TESTING RESULTS AMONG VOLUNTARY BLOOD DONORS IN SHAOXING REGION FROM 2019 TO 2022

Xingxiang Xing^a^, Lu Zhang^a^, Miaoyu Wang^a^

*^a^ Shaoxing Central Blood Station, Shaoxing City 312000, China.*

**Background:** To understand the blood safety situation in the Shaoxing area and propose effective measures to prevent and control transfusion-related infectious diseases.

**Subjects and Methods:** A retrospective analysis was conducted on the results of 8 transfusion-related infectious disease markers from 214,474 voluntary blood donation samples collected between 2019 and 2022. Five of these markers were compared with results from 190,264 voluntary blood donation samples collected between 2006 and 2010. An empirical study was also conducted on the blood testing strategy for voluntary blood donation.

**Results:** The overall rate of unqualified test results for the 8 markers was 1.25%, with the unqualified rates for ALT, HBsAg, Anti-HCV, Anti-HIV, Anti-TP, HBV-DNA, HCV-RNA, and HIV-RNA being 0.42%, 0.17%, 0.15%, 0.08%, 0.18%, 0.19%, 0.01%, and 0.01%, respectively. These rates were at a relatively good level and showed a significant decrease compared to the rates from 2006 to 2010. The analysis of population characteristics showed that the unqualified rates were relatively higher for males, young people, farmers and workers, those with low education levels, and first-time donors. A total of 83.38% of infection markers were detected as unqualified in all ELISA tests (including Anti-TP). The total detection rates of HBV, HCV, and HIV infection in ELISA testing after the first and second tests were completed were 39.24%, 59.61%, 85.94%, 100%, 39.89%, and 99.44%, respectively. The proportion of ELISA first test results that match the ELISA second test results was 21.32%. There were 16 cases of Anti-HIV positive, accounting for 94.12% of WB confirmed cases. All Anti-TP cases were screened by ELISA, and the detection rate of the first test was 41.42%. NAT could detect 65.85% of HBV-infected individuals (40.39% of which were supplemented by screening based on ELISA), 3.75% of HCV-infected individuals (12 individuals in total), and 100% of confirmed HIV-infected individuals (17 individuals, 1 of which was in the “window period”). The proportion of NAT and ELISA results that are consistent was 16.53%

**Conclusions:** ELISA is the basic means to ensure blood safety and can detect most transfusion-related infectious disease markers. NAT can detect hidden, “window period” infected individuals, etc., reducing missed diagnoses and is an important means of blood screening. Under the current context of mature and routine NAT, it is still necessary to continue with ELISA testing twice. Blood testing is an important step in preventing the transmission of transfusion-related infectious diseases, but the role of recruiting blood volunteers and pre-blood collection screening cannot be ignored.

### Acknowledgements

We would like to express our gratitude to Director Sang Liyong and Vice Director Fu Liqiang of the Shaoxing Central Blood Station, as well as Director Chen Jinkun of the Shaoxing Center for Disease Control and Prevention, for their significant assistance, guidance, and support in this study.

## LHMM23098 THE EFFECT OF THE COST OF MALIGNANT TUMOR TREATMENT ON THE DEGREE OF PSYCHOLOGICAL ANXIETY OF PATIENTS

Ying Du^a^, Mingxiu Yang^a^, Xinbin Xia^a^, Zhaojie Wang^a^, Jiale Li^a^, Zheming Tian^a^

*^a^ School of Humanities and Management, Hunan University of Chinese Medicine, 300 Xueshi Road, Yuelu District, Changsha, Hunan Province, 410208, China.*

**Background:** To calculate and analyze the treatment costs of malignant tumors in Hunan Province in 2021, to investigate the depression and anxiety of tumor patients, so as to clarify the influence of patients’ disease economic burden on their psychological anxiety, and to provide data support for the formulation and improvement of policies by the health department.

**Subjects and Methods:** Referring to the “2021 Hunan Province Health Finance Annual Report” and “2021 Hunan Province Health Statistics Summary”, based on the “System of Health Account 2011”, we calculated and analyzed the disease types, beneficiaries, institutional distribution and financing status of malignant tumors diseases. Then we investigated the status quo of depression and anxiety in patients with malignant tumor and analyzed its dynamic changes.

**Results:** In 2021, the total cost of malignant tumor treatment in Hunan Province was 440,596,800 yuan. The top five were malignant tumors of digestive organs (40.10%), malignant tumors of respiratory and intrathoracic organs (17.62%), and malignant tumors of breast (12.24%), female genital organ malignant tumors (9.88%) and lip, oral cavity and pharynx malignant tumors (6.87%). The 35 to 79-year-old age group has higher treatment costs. The costs are concentrated in general hospitals. Funding sources mainly come from government financing and family health expenditure. Among them, family health expenditure accounts for a relatively high proportion. The main influencing factors of malignant tumor hospitalization expenses are gender, length of stay, age, drug proportion, institution level and medical institution type.

**Conclusions:** The disease burden of malignant tumors is relatively serious, and it has a positive effect on patients’ anxiety. The primary medical and health institutions lack health resources. Because the household health expenditure accounted for a relatively high proportion, the economic burden of patients with malignant tumor Further aggravated. As a result, the patients’ psychological anxiety was promoted. This directly influenced the quality of life and life expectancy of patients. Therefore, hierarchical diagnosis and treatment should be promoted reasonably, key diseases and populations should be focused, medical security policies should be improved. These initiatives are expected to reduce the economic burden of disease in patients with malignant tumors and their families’, reduce their psychological anxiety and improve their quality of life and life expectancy.

## LHMM23099 A STUDY ON THE INFLUENCE OF PERCEIVED OVERQUALIFICATION ON TURNOVER INTENTION BASED ON EMOTION REGULATION THEORY

Kunxiu Lu^a^, Fu Jiang^b^, Yanyan Liu^a^

*^a^ Guangdong University of Science & Technology, Guangdong, 523083, China, ^b^ Guangdong Peizheng College, Guangdong, 510830, China*

**Objective:** Under the background of the new era, the turnover rate of the new generation of employees is high, which brings some problems to the enterprise development. From the employee’s point of view, the intention to leave must also have its own internal psychological factors based on the antecedent influences. In view of this, this study takes new employees who have worked for less than three years after 90 years as the investigation object, focuses on the inner evolution process of employees’ psychological feelings based on emotion regulation theory, and examines the research on the influence path of new generation new employees’ perceived overqualification on turnover intention.

**Methods:** Based on the theory of emotion regulation, the structural equation model was used to analyze the questionnaire of 357 new employees.

**Results:** Perceived overqualification had a positive effect on turnover intention, the adjustment effect of working years on perceived overqualification and turnover intention was not significant.

**Conclusion:** This study explores the direct influence of perceived overqualification on turnover intention and the influence factors of boundary. From the results of the study, perceived overqualification is one of the sources of stress for new employees, which in turn leads to negative emotional attitudes such as intention to leave, so companies need to be concerned about the psychological perceptions (e.g. perceived overqualification) of new generation employees. In addition, enterprises should pay more attention to employees who have left their jobs and understand the reasons for leaving so as to alleviate the talent shortage caused by employees leaving.

### Acknowledgements

This research received partial financial support from Planning Office of Philosophy and Social Sciences of Guangdong Province, China (Grant Number: GD21CGL07); Guangdong Education Department, China (Grant Number: 2021ZDJS113); and Guangdong University of Science & Technology, China (Grant Number: GKY-2022 KYZDW-6).

## LHMM23100 RESEARCH ON THE MOTIVATION FOR FRISBEE PARTICIPATION FROM THE PERSPECTIVE OF EMOTION REGULATION

Daogang He^a,b^

*^a^ School of Leisure Sports and Tourism, Beijing Sport University, Beijing, China, ^b^ Chengdu Aeronautic Polytechnic, Chengdu, China.*

**Background:** Ultimate Frisbee is a popular recreational sport that combines physical exercise and recreation, and is emerging as a lifestyle choice for young people. Previous studies have shown that participating in leisure-time physical activity is linked to improved mental well-being, but few studies have investigated the relationship between motivation and mental health. This study aimed to segment and analyze the motivations for participating in Ultimate Frisbee and explore their association with mental health.

**Methods:** A mixed research approach was used, based on self-determination theory, to investigate the drivers of participation in Frisbee sports. This included the Physical Activity and Leisure Participation Motivation Scale, observational data from interviews, and field surveys of disc participants (N=207), as well as focus group interviews with 18 participants. Data were analyzed to identify motivations for disc sports participation and their relationship with mental health, using the basic psychological needs theory.

**Results:** The study identified six dimensions of motivations for disc sports participation, including entertainment, appearance, physical condition, others’ expectations, competition/ego, and social interaction. These dimensions were related to the need for autonomy, competence, and relatedness, represented by health, recreation, and socialization. All motivations for participation were related to mental health, in addition to physical condition. Qualitative analysis identified three motivational factors, including gender equality, trend following, and additional skill level, which were related to the six dimensions. Psychological need satisfaction was primarily related to changes in autonomy and competence, as well as physical activity and mental health.

**Conclusions:** The study identified six distinct motivations for participating in disc sports, including recreation, appearance, physical conditioning, expectations from others, competition/ego, and social interaction. These dimensions were further elaborated upon, and three additional motivational factors - gender equality, following trends, and skill level - were also identified. Motivators related to mental health, such as social interaction, recreational motivation, appearance, and expectations of others, were confirmed as significant factors for participating in Frisbee. Releasing psychological stress through participation in Frisbee, thus promoting mental health and enhancing well-being, is also an important motivation for people to participate in Frisbee. The following trends and gender equality as motivational factors of disc participants can firmly explain the widespread phenomenon of disc sports. It also provides a new perspective for assessing disc sports and their participation motivation from a sociological perspective. The resulting framework provides a comprehensive understanding of the various motivations that drive individuals to participate in disc sports.

### Acknowledgments

This study was supported by a grant of the Youth Project of National Social Science Foundation of China (to Xi CHEN) (21CTY007) and Sichuan Province Philosophy and Social Science Key Research Base Tianfu International Sports Events Research Center (YJY2020010).

## LHMM23101 AN EMPIRICAL STUDY ON STRATEGIES TO IMPROVE THE QUALITY OF CHILDCARE SERVICES FOR CHILDREN UNDER 3 YEARS OLD FROM THE PERSPECTIVE OF REDUCING PARENTING ANXIETY OF CHINESE PARENTS

Weiwei Huang^a,b^, Shuyue Zhang^a^, Qiuju Wang^c^

*^a^ Faculty of Education, Guangxi Normal University, Guilin 541004, China, ^b^ School of Education, Zhaoqing University, Zhaoqing 526061, China, ^c^ Normal College, Eastern Liaoning University, Dandong 118003, China.*

**Background:** The healthy physical and mental growth of children is a major concern for parents, and childcare services for children under the age of 3 are an important form of early childhood education in China. High-quality childcare services are a basic guarantee for the healthy physical and mental growth of children and an important way to reduce parents’ parenting anxiety. Therefore, in response to the current situation that child care services in China fail to meet parents’ psychological needs, this study explores how to improve and optimize the quality of child care services for children under 3 years old from the perspective of enhancing parents’ satisfaction and reducing parents’ parenting anxiety

**Subjects and Methods:** In this study, on the basis of in-depth understanding of parents’ psychological needs and the factors affecting the parenting anxiety of parents through interviews, the author compiled the Survey Scale of Parents’ Satisfaction with Childcare Service Quality for Children Under the Age of 3.The Survey Scale of Parents’ Satisfaction with Childcare Service Quality for Children Under the Age of 3 was used to survey parents in a province in East China, and 516 valid questionnaires were collected, and the data were analyzed using the Kano-IPA integrated model.

**Results:** The analysis results showed that in order to reduce parents’ anxiety and increase their willingness to send their children to childcare institutions, childcare institutions need to improve the five areas parents are most concerned about: “Childcare service institution could provide safety inspection reports for their decoration materials, facilities, equipment, toys, etc.,”, “Service personal of the childcare institution has first aid knowledge”, “Specific sleeping area in the childcare institution”, “Independent and safe outdoor space for activities in the childcare institution”, and “The childcare institution has sufficiently large indoor activity space for children”.

**Conclusions:** Whether the materials used in the hardware facilities of childcare institutions for children under the age of 3 are safe or not is an issue that parents are very concerned with, and it is a basic attribute that affects the satisfaction of parents. The rationality and adequacy of the space setting of childcare institutions for children under 3 years old are factors parents attach importance to, and they are very easy to be perceived and evaluated by parents due to obvious comparability. The qualifications of the staff of the childcare institution constitute a factor that ensures the quality of the service. Only by paying close attention to the psychological needs of parents and improving the quality of childcare services, can childcare institutions institutions improve the satisfaction of parents, reduce the anxiety of parents in raising children, improve the willingness of parents of child-bearing age to have children, and alleviate the problem of population aging.

### Acknowledgments

This study was supported by a research grant from the National Natural Science Foundation of China (32060197).

## LHMM23102 TEACHING MODELS FOR ART MAJORS WITH THE CHARACTERISTICS OF INTANGIBLE CULTURAL HERITAGE LIVING TRANSMISSION FROM THE PERSPECTIVE OF PSYCHOLOGICAL HEALTH

Jia Zheng^a^

*^a^ Xi’an Polytechnic University, Xi’an, Shaanxi 710048 China.*

**Background:** The psychological health of individuals is of paramount importance in the university context, where there is a constant demand for innovation and inheritance of both traditional culture and modern developments. This study proposes an innovative model of instruction for art majors, with a focus on the living transmission of intangible cultural heritage (ICH) as its defining characteristic. The study underscores the positive interaction between psychological health education and the inheritance of ICH in arts courses.

**Subjects and Methods:** Four paired models for the “living transmission” of ICH - namely, “theoretical teaching”, “practical teaching”, “talent cultivation base construction”, and “Internet teaching”- that incorporate diverse cultural elements, enrich students’ innovative thinking abilities and enhance their confidence, thereby providing potential avenues for independent entrepreneurship were proposed.

**Results:** The study finds that learning ICH skills enriches the content of talent cultivation, improves an individual’s manual skills, and has a positive effect on concerning issues such as stress relief and anxiety emotions discharge in modern society.

**Conclusions:** This study highlights the necessity of integrating “living transmission” elements of ICH into theoretical teaching, practical teaching, talent cultivation base construction, and internet teaching, and establishes the innovative teaching model of the “living transmission” of ICH in art teaching.

## LHMM23103 RESEARCH ON THE EFFECT AND CONDUCTION MECHANISM OF TECHNOLOGY INNOVATION IN CROSS-BORDER INVESTMENT BASED ON PSYCHOLOGICAL NEEDS

Yang Li^a^

*^a^ School of Public Finance and Taxation, Central University of Finance and Economics, Beijing, China.*

**Background:** Cross-border investment is a strategic choice for enterprises to test their comprehensive capabilities, which requires comprehensive measurement of their own financial capabilities, management levels, mental health of enterprises managers, market sensitivity and technology absorption capabilities. Based on the data of enterprises with overseas connections among China’s A-share non-financial listed companies from 2009 to 2019, this paper attempts to construct a theoretical framework of technological innovation effect and transmission mechanism of cross-border investment of Chinese enterprises.

**Subjects and Methods:** Based on the theory analysis, this research uses econometric methods, using enterprise-level microdata for data analysis and model regression. Meanwhile, in the process of analyzing the innovation spillover effect of cross-border investment of enterprises, this research adopts the two-stage least squares method and introduces instrumental variables for endogenous problem analysis.

**Results:** The results show that cross-border investment by Chinese enterprises has a positive effect on technological innovation, and the leap in technological innovation capability is the most significant through the reverse technology spillover mechanism, followed by the optimization effect of resource allocation, and the transmission effect of financing constraint mitigation is not significant. In the process of cross-border investment, entrepreneurs show their desire and demand for technological innovation, market sensitivity and personality and psychological needs determine the effectiveness of cross-border investment innovation.

**Conclusions:** Based on the research conclusions, this paper further puts forward policy suggestions to optimize government support to guide more enterprises to promote technological innovation through cross-border investment.

## LHMM23104 A TONE-3 SANDHI STUDY OF MANDARIN-SPEAKING COCHLEAR IMPLANTED

Zhiyue Zou^a^, Qiuchen Zheng^a^

*^a^ Guangzhou Xinhua University, Guangzhou 510520, China.*

**Background:** To investigate whether the T3 sandhi of Mandarin in adults with cochlear implantation has the same computational mechanism as in hearing adults.

**Subjects and Methods:** 6 cochlear-implanted adults and 6 hearing adults were tested for mandarin T3 sandhi. After collecting the corpus, the analysis is carried out in two ways: first, three teachers of speech correction for the hearing impairment score all the corpus subjectively, and compare the correct rate of pitch shift; second, extract the fundamental frequency through the Praat (a speech analysis software) and convert it into a semitone value, make a contrast curve and observe.

**Results:** From the perspective of the correct rate of tone-shifting in the two groups, the correct rate of the three types of words in the experimental group was much lower than that in the control group, the correct rate of true words was higher than that of the other two types of words, and the correct rate of false words was the lowest. From the analysis of the comparison curve, it was found that the curve of some subjects in the experimental group was close to that of the control group, but the fluctuation was small, and it could still be regarded as a correct tone change. The curves of other subjects were flat or fluctuated greatly. When combined with non-T3, the tail tuning was up, which has the meaning of the original tuning. Therefore, it shows that adults with cochlear implants have mastered a certain T3 tuning mechanism.

**Conclusions:** The T3 sandhi of adults with cochlear implantation is far behind that of hearing adults. If they want to master the T3 sandhi more proficiently, they should first change the phonetic environment, and control the low point of the fundamental frequency by listening to the correct pronunciation. Hearing equipment is upgraded to the best or adjusted to expand the range of hearing thresholds.

### Acknowledgements

This work was supported by a project grant from Guangzhou Xinhua University (Grant No. 2019KYQN17).

## LHMM23105 REVIEW OF CHINA’S MODERN TOURISM DEVELOPMENT LEVEL AND RESIDENTS’ MENTAL HEALTH LEVEL IN THE FOURTY YEARS OF REFORM AND OPENING-UP POLICY (FROM 1978 TO 2018)

Xing He^a,b^

*^a^ China West Normal University, College of Management, Nanchong, Sichuan 637002, China, ^b^ Sichuan Research and Study Travel Development Research Center, Nanchong, Sichuan 637002, China.*

**Background:** Since the reform and opening up for 40 years, my country’s tourism industry has experienced a development process from a rise, rapid to a comprehensive, and has gradually become the most dynamic industry in the national economic system.

**Subjects and Methods:** According to the tourism research and residents’ mental health level situation in the past 40 years of my country’s reform and opening up, the development of my country’s modern tourism industry is divided into four development stages: commercialization, marketization, industrialization and socialization, and summarizes eight major turning points in the development of my country’s modern tourism industry. This study chooses worry, perceived control and desire to stimulate to represent emotion, cognition and motivation respectively, and constructs an impact model of psychological factors on tourism risk prevention behavior. Taking actual tourists as the research object, it uses questionnaire method to conduct empirical tests to verify the deep influence mechanism of emotional factors, cognitive factors, motivation factors and risk prevention behavior, as well as the condition boundary of this mechanism channel.

**Results:** It also sorts out, compares and reviews related literatures with qualitative and quantitative research methods, which usefully supplements the theoretical and methodological results of modern tourism research in my country since the reform and opening up, and also conducts research on the shortcomings of modern tourism development in my country Reflection. The study found that the impact mechanism of psychological factors on tourists’ risk perception and prevention behavior is very complex. Worry will not directly affect risk prevention behavior, but will significantly affect prevention behavior through perceived risk; Perceived control directly and indirectly affects risk prevention behavior through perceived risk; Stimulating desire will have a significant negative impact on risk prevention behavior. This mechanism path is regulated by tourism experience. Compared with the first tourists, the revisiters’ concerns, perceived control and perceived risk have a greater impact on risk prevention behavior. Tourism experience also regulates the intermediary role of perceived risk, while the intermediary role of perceived risk of first-time tourists is not significant; The intermediary effect of perceived risk of revisiters is significant.

**Conclusions:** Therefore, more attention should be paid to the impact of psychological factors such as emotion, cognition and motivation on tourists’ tourism risk perception and prevention behavior, and more accurate risk management strategies should be adopted for newcomers and revisiters. At the same time, it is proposed that the combination of modern information technology and “tourism+” industrial research, in the face of unknown public safety incidents, strengthening international cooperation, and building a world tourism community with a shared future will be the development trend of modern tourism in my country.

### Acknowledgements

This paper is Supported by the 2022 project of Sichuan Social Science Planning (No.: SC22C026), the Sichuan Ethnic Mountain Economic Development Research Center Project (No.: SDJJ202224), the Sichuan Ethnic Mountain Economic Development Research Center Project (No.: YX22-38), the Sichuan Provincial Key Research Base for Humanities and Social Sciences of Colleges and Universities, the 2022 project of Sichuan Research Travel Development Research Center (No.: YX22-38) The 2022 Project of Sichuan Tourism Development Research Center, a key research base of social sciences in Sichuan Province (No. LY22-40), the Innovation Team for Studying Travel Theory and Practice of West China Normal University (No. SCXTD2022-6), the 2021 Doctoral Initiation Project of West China Normal University (No. 21E002).

## LHMM23106 STUDY ON THE BEHAVIOR AND INFLUENCING FACTORS OF CHINA’S RESIDENTS’ CHOICE OF MEDICAL PLACES: EMPIRICAL EVIDENCE FROM CHINA

Jun Chen^a,b^, Panzhen Zhao^c,d^, Long Shi^a^

*^a^ Youjiang Medical University for Nationalities, Baise, China, ^b^ Philippine Christian University Center for International Education, Manila, Philippines,1004, ^c^ Faculty of Educational Studies, Universiti Putra Malaysia, Selangor, Malaysia, ^d^ College of Foreign Languages, Baise University, Baise, China.*

**Background:** The first consultation at the grass-roots level is the key link to promote the construction of hierarchical diagnosis and treatment system, which is conducive to promoting the sinking of public health resources and increasing the accessibility of residents’ medical services. Research on residents’ choice behavior and its influencing factors, which has important practical significance for establishing the first diagnosis system at the grass-roots level and guiding residents to form good medical habits.

**Subjects and Methods:** Based on the data of household follow-up survey in China in 2018, based on Andersen’s behavior model as the theoretical analysis framework, the article analyzed the influence of predisposition factors, energy-promoting factors and demand factors on the behavior of Chinese residents’ choice of medical facilities by using the method of disorder and multi-classification Logistic regression.

**Results:** The study found that the grass-roots medical institution is the place with the highest possibility for Chinese residents to go to hospital when they are ill. Meanwhile, due to the unbalanced distribution of medical resources in China, there is a certain degree of difference in the medical level of different levels of medical institutions. There are obvious differences in the behavior of Chinese residents in choosing medical institutions for medical treatment when they are ill. Still, more than one third of respondents prefer to go to general hospitals when they are ill. The study also found that (1) in terms of propensity factors, gender, job nature, education level, social frequency and satisfaction with medical conditions had a significant impact on residents’ choice behavior of medical places; (2) in terms of energy promoting factors, personal income, medical insurance participation, etc. had a significant impact on residents’ choice behaviors of medical places; (3) in terms of demand factors, Health self-assessment and chronic disease have significant influence on residents’ choice behaviors of medical places.

**Conclusions:** The improvement of the system of first diagnosis at the grass-roots level shall take into full consideration of the influencing factors of Chinese residents’ choice of medical facilities, guide the residents to choose basic medical institutions for medical treatment through improving the incentive mechanism of basic medical insurance, and encourage residents to cultivate the reasonable habit of first-time diagnosis and two-way referral system. so as to promote the optimal allocation of medical resources.

### Acknowledgments

This study was supported by the Project of the Planning Project of Guangxi Province Philosophy and Social Science (2021CGL002), National Natural Science Foundation of China (72164036), National Natural Science Foundation of Guangxi Province (2019JJA180060).

## LHMM23107 THE APPLICATION AND PRACTICE OF DESIGN TEACHING REFORM FOR MATERIALIZATION OF CREATIVE IDEAS FROM THE PERSPECTIVE OF PSYCHOLOGICAL HEALTH

Huizhen Xu^a^

*^a^ Fair Friend Institute of Intelligent Manufacturing, Hangzhou Vocational& Technical College, Zhejiang, China*

**Background:** With the continuous development of the times and the deepening reform of university education, the engineering education model is also changing, which presents new challenges and opportunities for the cultivation of design talents. The transformation and upgrading of enterprises have intensified the challenges of design talent cultivation, and how to effectively carry out design education and cultivate innovative design talents with complex technical skills has put design teachers under increasing pressure. However, these pressures may lead to psychological problems such as anxiety and emotional disorders among teachers, which will affect students’ learning outcomes and teachers’ teaching reforms if not addressed effectively and timely. The purpose of this study is to explore the current situation of talent cultivation in design disciplines and the reform practice of design teaching from the perspective of psychological health.

**Methods:** This study adopts a problem-oriented approach, introduces the concept of health psychology and TRIZ theory, and uses the cause-and-effect analysis method to conduct an in-depth analysis of the pain and difficulties in design teaching to find the deep-seated reasons for the difficulty in implementing teaching reform. Combining the deep-level reasons, the expectation model of physical-field is constructed, the design teaching reform strategy with psychological health as the starting point is proposed, and the design teaching reform scheme of creative ideas materialization is practically applied to solve the problems of difficult creative innovation, difficult implementation and difficult technical realization in design teaching, and the feasibility of its scheme is verified.

**Results:** For design majors, a four-stage “TRIZ+ Core Curriculum” training system was designed, a “TRIZ+ Design” resource case platform of interest to students was created, a “studio-style” teaching environment and an interdisciplinary teaching team were formed, and precise assessment and evaluation were designed. The five-step project-driven teaching method is implemented, including “initiation, teaching and learning, practice and enlightenment, evaluation, and expansion”, which effectively solves the problems in design teaching, transforms virtual concepts into physical objects, enables students to understand design ideas more intuitively, and cultivates students’ understanding of design. Students and teachers have achieved fruitful teaching results, and the satisfaction of enterprises has been improved, and the effect of teaching reform has been significantly improved.

**Conclusion:** From the perspective of psychological health, the teaching reform strategy of creative ideas materialization can transform the pressure of teachers and students into motivation and relieve the psychological pressure of teachers and students in design disciplines. This teaching reform strategy can not only help teachers improve their teaching ability, but also help students enhance their innovation and engineering realization ability, and promote the rapid development of design education. Through the successful practice of this study, teachers and students will get a broader space to play and a more genuine sense of achievement.

## LHMM23108 RESEARCH ON THE INFLUENCE OF STRATEGIC HUMAN RESOURCE MANAGEMENT ON ENTERPRISE ORGANIZATIONAL EFFICIENCY AND EMPLOYEES’ MENTAL HEALTH

Jiaqi Zhang^a^, Fan Zhang^b^, Siti Rohaida Binti Mohamed Zainal^c^

*^a^ School of Management, Universiti Sains Malaysia (USM), GelugorPulau Penang, Malaysia, ^b^ School of Innovation and Entrepreneurship, Guangdong Polytechnic Normal University (GPNU), Guangzhou, China, ^c^ School of Management, Universiti Sains Malaysia (USM), GelugorPulau Penang, Malaysia.*

**Background:** Based on the perspective of competitive value architecture, this paper discusses the impact mechanism of strategy on organizational effectiveness of enterprises, and proposes corresponding application strategies.

**Subjects and Methods:** In the increasingly competitive environment, enterprise employees are facing increasing psychological pressure. Enterprise employees are prone to problems such as low mood, physical and mental exhaustion, lack of work enthusiasm, decreased awareness of corporate culture, and lack of innovative spirit. Therefore, this puts forward higher requirements for enterprise human resource management. From the perspective of competitive value architecture, this article discusses strategic human resources, competitive value architecture theory, and strategic human resource management models. It analyzes the impact mechanisms of different strategic human resource management models, such as win-win cooperation, open innovation, control and coordination, and performance evaluation models, on enterprise organizational effectiveness, reveals the role of strategic human resource management in enterprise organizational effectiveness, and propose corresponding application strategies.

**Results:** The impact carriers of strategic human resources on organizational effectiveness include organizational culture and organizational commitment. In order to protect and enhance the mental health of employees, it is very valuable to pay attention to the psychological empowerment of employees. Employee mental health is the key to improving organizational effectiveness. Among them, organizational commitment also affects employees’ psychological empowerment, which in turn affects their level of loyalty to the organization. Employees feel empowered when they feel influential and meaningful within the organization. Therefore, psychological empowerment is of great significance to both employees and organizations, such as improving employee motivation and engagement, which in turn affects organizational performance. Building on the concept of psychological empowerment, this paper finds that, under the win-win cooperation model, a scientific, reasonable, fair and impartial performance contribution evaluation system is used to improve employee compensation satisfaction, enhance employee evaluation fairness, and generate a sense of belonging to the enterprise, thereby promoting the achievement of organizational efficiency goals. Under the open innovation model, both professional quality and innovative creativity affect organizational effectiveness through organizational culture and organizational commitment, stimulate enterprise innovation and creativity, promote enterprise development and innovation, promote the integration of internal and external resources, build resource synergy, and improve organizational efficiency. The control and coordination model enhances organizational effectiveness through a fair, just, and coordinated organizational culture and standardized and institutionalized organizational commitment. The performance evaluation model adopts a quantitative evaluation method to directly evaluate the contribution of strategic human resources to the organizational effectiveness of enterprises, providing a basis for optimizing the structure of strategic human resources and improving the quality of strategic human resources.

**Conclusions:** This study found that, strategic human resource management mainly affects organizational effectiveness through organizational culture and organizational commitment. It is undeniable that there is often a positive correlation between employees’ perceptions of psychological empowerment and mental health. The significant impact of organizational commitment on psychological empowerment of employees will further affect organizational effectiveness by affecting factors such as employee loyalty and enthusiasm.

## LHMM23109 VALIDITY OF PULL-UPS INDICATING PHYSICALLY EXCELLENT CONDITIONS IN MALE COLLEGE STUDENTS FROM NORTHEAST REGION, CHINA

Hongzhao Wang^a^, Landong Xu^b^, Sixiang Tao^a^, Jingtang He^a^

*^a^ Department of Physical Education, Huainan Normal University, Huainan 232038, China, ^b^ School of Physical Education, Changchun Guanghua University, Changchun 130033, China.*

**Background:** Fitness and health (FH) testing in universities in China involved several health related components, such as BMI, vital capacity, flexibility, speed, aerobic endurance, power and muscle strength by National Fitness and Health Standards of Students (NFHSS). These components with specific reference values are considered markers of physical and mental health, and has been shown to be positively associated with cognition, weight status, and academic performance. Evaluation physical condition is a matter of concern of in public physical education (P.E) not only for conforming prevalence of FH conditions but also for implementing strategy of effective teaching. Monitoring physical fitness indicators is therefore considerable importance for social medicine, health education and health management, but it requires specialized equipment. Consequently, FH is rarely measured at the population level in public P.E teaching due to time, budget, complicated procedures and logistic limitations. A single-index of physical fitness components is a simple and cost-effective method to estimate FH status, which is an alternative to integrated testing system. Therefore, the purpose of the observational study was to examine the validity of pull-ups’ cut-off score screening FH status among physically excellent male college students from Northeast, China.

**Subjects and Methods:** The study was based on NFHSS, which was performed in 3 dimensional conducts consisting of morphology, function and fitness. A total of 5669 male college students aged 18-21 years were selected from Changchun Guanghua University. All participants’ FH status were categorized into trichotomy groups as excellent, sound and no-pass by NFHSS. The difference of FH were analyzed by one-way ANOVA with components of the dependent variable and FH status as the factor. Regression analyses indicated whether the pull ups was able to predict the FH status of male college students among physically excellent and sound. Receiver operating characteristic (ROC) curve was applied to evaluate pull ups cut-off score indicating FT status and to discriminate the prediction ability of male college students among physically excellent and sound.

**Results:** Mutually adjusted analysis showed a significant association between FH status and performance of pull ups in male college students who are physically excellent and sound. The cut- offs of pull-ups assessed by the ROC curve can discriminate the FH status between excellent and sound.

**Conclusions:** Pull-ups was significantly associated with FH status and contributed to the excellent/sound model’s prediction for FH status among physically excellent and sound students. Single index of pull-ups could be a useful prediction tool to identify and monitor variation in FH status of male college students between physically excellent and sound.

### Acknowledgments

This work was supported by a project grant from Scientific Research Foundation of Education Department of Anhui Province of China (Grant No.SK2021A0560).

## LHMM23110 A STUDY ON CHINESE EFL TEACHERS’ CLASSROOM EMOTIONS FOR FORMATIVE ASSESSMENT UNDER THE DOUBLE REDUCTION POLICY: A MENTAL HEALTH PERSPECTIVE

Yuchang Hou^a^, Lan Ding^a^

*^a^ School of Foreign Languages, Henan University, Kaifeng 475001, China.*

**Background:** Under the current situation of the “double reduction” policy in China, formative assessment in EFL (English as a foreign language) classroom is receiving increasing attention. According to Positive Psychology, emotions have direct influence during language learning process. For achieving satisfactory result of formative assessment, the present paper aims to investigate the EFL teacher’s emotions in formative assessment and put forward practical strategies and skills to better use teacher’s positive emotions and reduce their negative emotions, such as anxieties, gloominess and self-doubt.

**Subjects and Methods:** To study the current level of emotions of EFL teachers towards formative assessment, questionnaires and interviews were administered randomly to 20 EFL teachers from 4 major junior high schools in China. To study the influence of EFL teacher’s positive emotions on the effect of formative assessment, an 18-week positive psychology-based professional development program was conducted with a series of methods for result analysis including interviews, class observations, discussions and exams. To make the above-mentioned two studies reasonable, logical and scientific, all the experiments in the present paper were tightly grounded on the relevant theories and models to positive psychology.

There are two limitations in the present study. First, the number of participants is not large. Second, the professional development program does not last long enough. Despite these limitations, this is a new study showing the emotion factor of EFL teachers during the formative assessment, a more emphasized kind of assessment under the “double reduction” policy newly and currently implemented in China.

**Results:** Study 1 of the present paper found that the current level of classroom emotions of Chinese Junior School EFL teachers is not satisfactorily high. Data collected from Study 2 of the present paper showed that the professional development program was greatly beneficial. All the 20 participants have demonstrated an increased level of empathy toward students, which in turn brings motivation, enjoyment and confidence to themselves.

**Conclusions:** Chinese teachers in junior high school have already noticed the importance of classroom emotions and teachers’ positive emotions can make students ease in mind and actively engage in class tasks. Moreover, teachers’ positive emotions can enhance their feedback that moves students forward. In general, teachers’ positive emotions can act as instructional resources for building a closer and friendlier relationship with students and boost their teaching of English eventually.

As we are trying to improve English teaching effectiveness and coming across more difficulties under the “double reduction” policy, it would probably be a reliable solution to shift from the summative assessment to the formative assessment, from the learner to the teacher, and from the teaching and learning of knowledge to the classroom emotions.

### Acknowledgments

This study was supported by the Teacher’s Development Research Fund of Henan University (Grant No.: YB-JFZX-2022-19) and the Education Reform Project for Postgraduate Program of Henan University (Grant No.: SYLAL202201) and the Undergraduate-oriented Project of Henan University Research Laboratories (Grant No: 20221701024).

## LHMM23111 LIFE EDUCATION FOR UNIVERSITY STUDENTS IN THE POST-PANDEMIC ERA FROM THE PERSPECTIVE OF PSYCHOLOGICAL HEALTH

Jialin Cheng^a^

*^a^ Zhuhai College of Science and Technology, Zhuhai 519041, China.*

**Background:** In the post-pandemic era, life education for university students has become particularly necessary and important, as the COVID-19 pandemic has not only caused huge losses to people’s lives and property, but also had a significant impact on the knowledge production mode and talent cultivation methods of higher education. In this context, life education for university students has acquired a richer connotation and significance.

**Subjects and Methods:** It should cover three aspects: life consciousness education, survival ability education, and life value sublimation education. To achieve this, it is necessary to further broaden the concept of life education, enrich its content, innovate its methods, build its platforms, and ensure the effectiveness of the education process and its components.

**Results:** The serious problems of narrow educational concepts, knowledge-based educational contents, fixed educational methods, limited educational levels, and closed educational platforms in life education for university students have seriously affected the formation of their life consciousness, the improvement of survival ability, and the sublimation of life values. Apparently, focusing on the physical and mental health and life development of university students, strengthening life education for university students in the post-epidemic era is not only a timely choice to adapt to the external environment of higher education but also an active choice to implement the fundamental task of moral education and talent cultivation.

**Conclusions:** Strengthening life education for university students can help them establish a correct view of life and practice a positive outlook on life.

## LHMM23112 STUDY ON THE INHERITANCE AND DEVELOPMENT OF TRADITIONAL ETHNIC SPORTS IN COLLEGES AND UNIVERSITIES IN THE GUANGDONG-HONG KONG-MACAO GREATER BAY AREA FROM THE PERSPECTIVE OF “COMBINATION OF SPORTS AND MEDICINE”

Ming Wang^a^, Zehui Jiang^a^

*^a^ Zhuhai College of Science and Technology, Zhuhai, Guangdong 519041, China.*

**Background:** With the change of times, the rapid development of economy and society, medical and health undertakings and sports industry is undergoing a period of rapid development; The decline of physical health has gradually become the main public health problem faced by human beings, and people’s pursuit and need for health has become a rigid need. The Outline of the Healthy China 2030 Plan has been issued, and the combination of physical medicine has quickly become a new model of health intervention, emphasizing the need to add more means of physical fitness to modern medical treatment and rehabilitation.

**Subjects and Methods:** By looking up literature related to the combination of physical medicine and its model, traditional national sports on CNKI platform, and looking up books about sports promoting health in the library, data were collected for the writing of this paper. At present, we advocate the organic combination of sports and medicine as well as the health intervention of non-medical means, and aim to establish the promoting mechanism of health education in schools with a focus on colleges and universities, and analyze the differences in the implementation of national traditional physical education courses under the concept of combination of physical medicine. From the school, physical education teachers, families and students four aspects try to analyze the relevant influencing factors, and put forward countermeasures.

**Results:** The combination of physical medicine is a long-term method to enhance physical fitness and immunity. Based on the construction of “first-class undergraduate courses”, this paper starts from three aspects: general education courses, professional education courses and practical education courses, establishes the educational concept of “integration of physical and medical”, optimizes the construction of the content system of “integration of physical and medical” ethnic traditional sports courses, and cultivates ethnic traditional sports guidance talents to ensure public health. It can also build up cultural confidence in colleges and universities and promote the inheritance and development of traditional national sports.

**Conclusions:** The “Guangdong-Hong Kong-Macao Greater Bay Area” strategy has been put forward and implemented, and the exchanges and coordination in the economy, science and technology, education, culture and quality life circle of the “9 + 2” urban agglomeration are constantly expanding. Traditional ethnic sports, as an important way of cultural exchange, play an important role in promoting the coordinated development of sports and industries in the Guangdong-Hong Kong-Macao Greater Bay Area. By means of literature research, field research, SWOT analysis and other methods, this paper analyzes the strengths, weaknesses, opportunities and threats of the coordinated development of ethnic traditional sports in colleges and universities in the Guangdong-Hong Kong-Macao Greater Bay Area. It is suggested to promote the coordinated and integrated development of traditional ethnic sports in higher education institutions in the Greater Bay Area by implementing national strategies and policies, building exchange platforms for higher education institutions, cultivating academic exchange talents, building ethnic sports events, and combining sports and medicine.

### Acknowledgments

This work was supported by 2021 Annual Education Science Planning Project (Higher Education Special Project) “Research on the inheritance and development of traditional ethnic sports culture in universities in the Guangdong-Hong Kong-Macao Greater Bay Area” (Project No.: 2021GXJK080) and “Three Levels” talent Construction Project of Zhuhai College of Science and Technology.

## LHMM23113 RURAL RESIDENTS’ DIGITAL LITERACY, DIGITAL IDENTITY PSYCHOLOGICAL ANXIETY DILEMMA AND PROMOTION PATH

Jingjing Cao^a^

*^a^ School of Culture and Media, Xi’an Eurasia University, Xi’an, Shaanxi, China.*

**Background:** With the continuous development of emerging digital media technology and the continuous expansion of the application scenarios of digital media technology in daily life, building a digital country has become a global consensus, and promoting the digital village strategy has become an inherent requirement and inevitable choice for building the network powerful nation,digital China and smart society. However, according to the survey *Investigation and Analysis Report on Digital Literacy in Rural China under the Background of Rural Revitalization Strategy* released by the Center for Informatization Study of the Chinese Academy of Social Sciences, the digital literacy gap between urban and rural residents is 37.5%. The digital literacy score of rural residents is significantly lower than that of other professional groups. Rural residents’ digital identity anxiety began to emerge.

**Subjects and Methods:** The psychological identity of digital identity and digital literacy of rural residents has a direct impact on the digital village, rural revitalization and digital China construction. How to improve the digital literacy of rural residents in the strategic perspective of comprehensively promoting rural revitalization has become an urgent problem to be solved. Therefore, based on the existing research, the author discusses the value connotation, current situation, existing problems and reasons of rural residents’ digital literacy, and then puts forward a practical and feasible way to improve rural residents’ digital literacy.

**Results:** The author puts forward four feasible suggestions according to the problems and reasons of rural residents’ digital literacy: solve the rural residents’ digital gap based on the problem of using digital products, solve the idle problem of digital products based on the use demand of rural residents, provide training on digital literacy and cultivate digital village culture, introduce digital technology cultivation projects and enhance the participation of rural residents.

**Conclusions:** Rural residents’ psychological anxiety about digital identity comes from the application of digital identity in individual virtual practice in the digital era. Improving the digital literacy of rural residents has a direct impact on alleviating this problem. Improving the digital literacy of rural residents is a long-term and arduous task, which requires a large amount of human, material and financial resources.

### Acknowledgements

This research is under the scientific research project “Research on the promotion path of digital literacy of rural residents in Shaanxi Province from the perspective of Rural Revitalization”, which is supported by the Shaanxi Provincial Education Department (Grant No. 22JK0133), and the project of the 14th Five-Year Plan “Research on the Practice of Mixed Teaching Mode for News and Communication Courses in the Digital Era”, which is supported by Shaanxi Academy of Educational Sciences.

## LHMM23114 ANALYSIS ON THE CULTIVATION AND MAINTENANCE OF COLLEGE STUDENTS’ HEALTHY PERSONALITY FROM THE PERSPECTIVE OF HUMAN RESOURCE MANAGEMENT

Wenqu Xu^a^

*^a^ School of business Minnan Normal University, Zhangzhou, Fujian 363000, China.*

**Background:** The healthy personality of college students is a kind of self-consciousness gradually built up by college students in their life experience with their lifestyle and life style. It is a comprehensive embodiment and important symbol of people’s world outlook, psychological quality and moral cultivation, and it is the psychological basis for college students to accurately grasp themselves, find a social position suitable for their own development, and obtain respect and goodwill from others. College students are the mainstay of the country’s future development and the most valuable human resources of the country. The research on the shaping of college students’ healthy personality and guiding college students to establish healthy and optimistic psychological quality have important practical enlightenment for cultivating college students to become talents with all-round development of morality, intelligence and physique and realizing the prosperity of the country.

**Subjects and Methods:** The subjects of this study are college students in a university in Fujian. The scale used in this study was the Big Five Personality Inventory simplified version (NEO-FFI). The Big Five personality trait model divides personality into five dimensions, including neuroticism, extraversion, openness, agreeableness and conscientiousness, which are measured by five sub-tests of 12 questions per dimension, with a total of 60 questions. The simplified Big Five Personality Inventory (NEO-FFI) is a personality measurement tool for Chinese college students. Its α reliability coefficient is 0.77 for neuroticism, 0.78 for extraversion, 0.63 for openness, 0.72 for agreeableness, and 0.74 for conscientiousness, which has good reliability and validity. Stratified sampling was used in the questionnaire survey, and 309 valid questionnaires were collected. In terms of statistical processing, SPSS22.0 Chinese version was used for statisticalanalysis of the survey data, mainly using specific statistical analysis methods such as descriptive statistics, independent sample T test and variance test.

**Results:** Through the analysis of the NEO-FFI questionnaire, the research found that: first, there is no significant difference in personality traits between different genders and different grades; Second, the personality traits of different majors, whether they are student leaders, future planning types and self-promotion types have significant differences in a certain dimension. Specifically, in terms of professional categories, the neuroticism of liberal arts is significantly higher than that of science; The conscientiousness and extraversion of student cadres are significantly higher than that of non-student cadres, while neuroticism is significantly lower than that of non-student cadres. In terms of future planning type, the extroversion and conscientiousness of students with clear goals were significantly higher than those with uncertain goals. In terms of self-improvement types, conscientiousness and extraversion were significantly higher in students with ideas and practices than in students with no ideas and practices, while neuroticism was significantly lower than in students with no ideas and practices.

**Conclusions:** College students in the new era are the hope and future of the country. The healthy personality of college students is related to the prosperity and prosperity of the country. It is suggested that the theory and method of human resource management should be integrated into the whole process of cultivating healthy personality of college students. Recruitment and configuration, training and development, performance management, salary welfare management and labor relations management six modules as the research basis, on college students’ healthy personality training targeted Suggestions, so as to cultivate good social adaptation ability and stable sense of self, and good quality, independent thinking, good ability to life positive and optimistic attitude, emotion regulation and interpersonal harmony friendly health, etc Excellent college students with personality characteristics will provide qualified builders and successors for realizing the prosperity of the country.

### Acknowledgments

This work was supported by a project grant from2020 Fujian Young and Middle aged Teacher Education Research Project (JAT200333) and 2019 General Project of Fujian Research Center for Socialism with Chinese Characteristics (2019ZTD19).

## LHMM23115 QUANTITATIVE RESEARCH ON PMC-E MODEL EVALUATION OF URBAN SPORTS INDUSTRY POLICY: BASED ON THE PERSPECTIVE OF EMOTION REGULATION

Xuejun Wang^a,c^, Yun Li^b^, Shanxian Li^a^

*^a^ College of Sports Economics and Management, Guangxi University of Finance and Economics, Nanning, Guangxi 530001, China, ^b^ School of Big Data and Artificial Intelligence, Guangxi University of Finance and Economics, Nanning, Guangxi 530001, China, ^c^ College of Education, Jose Rizal University, Manila, Philippines.*

**Background:** To speed up high-quality economic development and further give play to the sports industry’s role in promoting green development, expanding domestic demand and cultivating new economic driving force cannot be separated from the support of local policies. The introduction of local policies will promote economic development, expand employment, and alleviate pressure through internal emotional regulation to generate positive emotions such as joy and happiness.

**Subjects and Methods:** Taking the sports industry development of Guangxi as an example, the sports industry-related policies issued by 14 cities since 2018 were studied, focusing on the correlation between the policy issuance of each city and the development of the sports industry. By constructing the PMC-E quantitative policy evaluation system, the quality index of Six Aspects of Emotional Regulation local policies promoting the development of sports industry is quantified from a multi-dimensional perspective, and the quality index of local sports industry policy development is measured according to the weight, and the effect of various local policies promoting the development of sports industry is tested.

**Results:** Nanning, Liuzhou and Guilin had strong local policies. Good effect on emotional regulation, Urban sports industry policy is reasonable. Other cities are in the acceptable range, the city sports industry policy is generally applicable.

**Conclusions:** The average structure level of sports industry policies in Guangxi is reasonable, which promotes the release of vitality of sports market, Good emotional regulation, generating positive emotions. The PMC-E value of sports industry promoted by urban policies is consistent with the overall objective data, and the optimization suggestions are put forward for improving the local policies to promote the development of sports industry in Guangxi.

### Acknowledgements

This work was supported by the 2022 Guangxi Philosophy and Social Science Planning Project: Research on Artificial Intelligence Empowering High-Quality Sports Development in Guangxi (22FTY019); funded by the 2022 Guangxi First-class Discipline Applied Economics (Sports Economics and Management) Project.

## LHMM23116 FROM THE PERSPECTIVE OF PSYCHOLOGICAL IDENTITY: REFORM AND PRACTICE OF TRAINING APPLIED TALENTS IN SOCIAL SPORTS GUIDANCE AND MANAGEMENT UNDER THE BACKGROUND OF PRECISE EMPLOYMENT

Wenxing Tan^a,b^, Zhibing Zou^a^, Shiqi Liu^a^, Ruhai Fei^a^, Wang Zhang^a^, Licheng Qin^a^, Jiaxiang Fan^a^, Guosong Wu^a^, Liwei Zheng^a^, Nianmao Li^c^

*^a^ School of Sports and Health Science, Xiangsihu College of Guangxi Minzu University, Nanning, 530008, China, ^b^ Graduate School, University of Baguio, Baguio City 2600, Philippines, ^c^ School of Sports and Health Science, Guangxi Minzu University, Nanning, 530006, China.*

**Background:** From the perspective of mental health, the identification of their own identity is also an important evaluation index. Students’ recognition of their major affects their choice of future career and attitude towards future work, which is professional cognition from the level of consciousness. The employment of college students directly reflects the quality of talent training in colleges and universities, which is related to the selection and training of high-quality talents. Based on the perspective of psychological identity, Combined with the background of targeted employment, this paper analyzes various practical problems that affect the training of social sports guidance and management professionals.

**Subjects and Methods:** Subjects and Methods: Based on a common perspective of mental health and professional identity. This paper takes the major of social sports guidance and management as the research object, and adopts the research methods of literature review, interview and logical analysis.

**Results:** The research shows that there are some problems in the training process of applied talents in social sports guidance and management: the absence of courses and teachers, the lack of social sports industry experience of teachers; The professional boundary is not clear, and the characteristics of applied professional skills training are not significant; The separation of specialty and occupation makes it difficult to cultivate and design applied talents. The disconnection between the profession and the society and the failure to train professional talents according to the needs of the society. The fundamental goal of talent training in colleges and universities is to cultivate healthy professional talents for the society, and the health here should be a comprehensive health state including mental health and moral health. Psychological identity is the basis of students’ identification of professional learning, recognition from the internal recognition of the professional, is also the basis of learning motivation, and has a close relationship with their academic achievement. Application-oriented talents should be cultivated in an educational environment conducive to students’ psychological identity, which can help students have a deeper understanding of their major. This paper puts forward the construction of application-oriented courses based on professional requirements to realize the effective diversion of talents; puts forward practical skills training-oriented by professional characteristics to link vocational qualification certificates; puts forward social need-oriented service-oriented teaching practice to accurately meet employment needs; puts forward the cultivation of core competitiveness oriented by targeted employment to improve the reform and innovation ways of talent provision mechanism.

**Conclusions:** The major of social sports guidance and management is an application-oriented talent trained for social sports service and a professional talent characterized by practical maintenance group. Considering the students’ mental health and professional cognition, the formation of their own professional identity is a prerequisite for the establishment of long-term career development planning. Professional identity directly affects the learning attitude, motivation and performance of students majoring in physical education, as well as their career development direction. Based on students’ psychological identification with the major of social sports guidance and management, a four-in-one application-oriented talent training system of “theoretical knowledge-sports skills-teaching ability-comprehensive literacy” is constructed, and the exploration and practical experience of “high-grade, targeted and advanced” talent reform are summarized in combination with the background of targeted employment.

### Acknowledgments

This work was supported by 2022 key project of Guangxi Higher Education Undergraduate Teaching Reform Project [number 2022JGZ187]; 2023 Guangxi Young and Middle-aged Teachers Basic Research Ability Improvement Project [number 2023KY1668]; 2023 Annual Project of the “14th Five-Year Plan” of Guangxi Education Science [number 2023A122].

## LHMM23117 BEYOND LITERAL MEANING: NON-LITERAL LANGUAGE COMPREHENSION AND GENERATION IN CHILDREN WITH AUTISM SPECTRUM DISORDER

Hui Li^a^, Jinsan Qi^b^, Hui Wang^c^, Zhixiang Gao^d^

*^a^ School of Foreign Languages, Zhejiang University, Hangzhou 310058, China, ^b^ School of Foreign Languages, Anyang Normal University, Anyang 455000, China, ^c^ College of Humanities, Ningbo University of Finance & Economics, Ningbo, 315175, China, ^d^ School of Foreign Languages, Institute of Disaster Prevention, Sanhe 065201, China.*

**Background:** Non-literal language is a typical pragmatic expression, and the comprehension and production of non-literal language involve advanced cognitive activities. Children with autism spectrum disorder (ASD), however, are pervasively impaired in processing non-literal language. Therefore, non-literal language comprehension and production ability can be important means to investigate pragmatic competence in autistic children. Recently, with the shift of language pathology to pragmatics, non-literal language processing in autistic children has become a research hotspot in clinical pragmatics worldwide. This paper intends to systematically formulate the comprehension and production characteristics, and the developmental patterns of non-literal language in autistic children, and then to provide worthwhile insights for future clinical language research and intervention.

**Subjects and Methods:** According to the Convention on the Rights of the Child, this paper concerns autistic children under the age of 18. This review paper first retrieves empirical studies in irony/metaphor/idiom/proverb/pun/metonymy in autistic children from the Web of Science in the past two decades. And literature is further selected by refining the titles and abstracts, examining the comprehension and production of non-literal language in autistic children, and then expanding the search via literature-tracking. Finally, 34 empirical research papers are chosen.

**Results:** This paper summarizes that research in non-literal language comprehension and production in autistic children roughly follows three pathways, that is, the characteristics of acquisition and development, the cortical activation patterns, and the influencing factors. To be exact, compared with their typically developing counterparts, not only are autistic children developmentally delayed in comprehending and producing non-literal language, significant differences exist among autistic children, but also they exhibit unique neural activation patterns. Moreover, performances in autistic children are constrained by their structural language competence, general cognitive mechanisms and deficient capacity of online semantic integration, and the complexity or diversity of the experimental tasks. Even so, autistic children do possess some capabilities to comprehend and produce non-literal language and even exhibit creativity to some extent.

**Conclusions:** Three research threads sorted out by this paper presents a comprehensive picture of research in comprehension and production of non-literal language in autistic children. Overall, typically impaired and delayed in comprehending and producing non-literal language, autistic children demonstrate their unique developmental patterns and characteristics. Moreover, due to the complex nature of non-literal language, the nature of experimental tasks, and autistic children’ cognitive abilities, their social environments, structural language capacities as well as pragmatic skills need to be coordinated. However, there exists a debate over the specific role these factors play. Heterogeneous participants and lack of standardized methodologies make the experimental results lack comparability and inclusiveness. Accordingly, several implications for future research and intervention are advocated such as the standardization of experimental research, the implementation of qualitative analysis, the setting of a specific target for individuals, etc.

### Acknowledgments

This study is supported by the key project of Social Science Foundation of China “A Comparative Study on Multimodal Reference Strategies of Chinese Children with Autistic and Typical children” (Project No: 17AYY009).

## LHMM23118 THE RELATIONSHIP BETWEEN THE DEGREE OF PSYCHOLOGICAL ANXIETY OF COLLEGE STUDENTS AND THE INTENTION TO CONTINUE USING MOBILE TRAVEL APPS

Xiaomeng Lyu^a^

*^a^ School of Economics and Management, Shenyang Institute of Technology, Shenyang, Liaoning, 113122, China.*

**Background:** With the development of mobile internet technology and the popularity of smart phones in the world, mobile apps has rapidly entered the daily life of people, which has a profound impact on people’s thoughts, emotions and consumer behavior. It is generally believed that the cost of acquiring new customers is much higher than retaining existing customers. However, app users still have varying degrees of concern during the using process. More specifically, the level of psychological anxiety can to some extent affect users’ use behavior and intention to continue using. This study aims to investigate the relationship between the degree of psychological anxiety of college students and the intention to continue using mobile travel Apps.

**Subjects and Methods:** In order to achieve the purpose of the study, literature analysis method, questionnaire survey method and statistical analysis method were used in this research. In order to ensure the diversity of sample sources, different major groups of college students were selected from different universities in China. An online questionnaire platform “Questionnaire star” has been used to distribute and collect questionnaires. Finally, 489 questionnaires were collected. Before data analysis, strict data filtering is performed to check whether there is invalid data or abnormal values. Questionnaires with incomplete and missing items and the questionnaire with the same answer on 5 consecutive questions were considered invalid, and 445 valid samples were obtained. After data collection, SPSS 22.0 and AMOS 22.0 were adopted as tools for data analysis. The data analysis of this study was conducted using structural equation modeling (SEM) technique and followed the two-step approach of for assessing the measurement and structural models respectively.

**Results:** By referencing the results of significance analysis of path coefficients, the eight hypotheses proposed in the study were supported. These findings in this study are also consistent with those in Bhattacherjee’s study, the five original hypotheses of ECM model are supported. And this study further supports that ECM has the ability to interpret users’ continuance intention of mobile travel apps. Besides, the variable of perceived interactivity was also verified in the study to have an impact on satisfaction and continuance intention. The revised model can be used to guide research on post-acceptance behavior. Increased perceived usefulness, confirmation and perceived interactivity can reduce the degree of psychological anxiety of college students and improve users’ satisfaction and continuance intention. The results extend the findings of ECM model to the context of mobile travel apps and the hypothesis testing indicate that the ECM model is applicable to and can be stretched to the study of travel apps continuance intention.

**Conclusions:** Improved perceived interactivity can reduce psychological anxiety of college students and enhance consumers’ intention to continue using. E-commerce platforms should attach importance to enhancing perceived interactivity.

### Acknowledgments

This work was supported by my thesis advisor Professor Youngkyu Kim from Keimyung University.

## LHMM23119 THE PSYCHOLOGICAL MECHANISM OF WELLNESS HOMESTAY LANDSCAPE SPACE ATMOSPHERE CONSTRUCTION AND ITS HEALING FUNCTION

Rong Xie^a,b^, Qinghua Li^c^

*^a^ School of Architecture and Engineering, Suqian University, Suqian City 223800, China, ^b^ School of Fine Arts, Qiongtai Normal University, Haikou City 571100, China, ^c^ School of Arts and Communications, Suqian University, Suqian City 223800, China.*

**Background:** With the increasingly prominent problems of modern diseases, urban diseases and sub-health, the wellness industry, as an important supplement to modern medical and old-age care institutions, has been booming, and the wellness homestay is one of the important forms of business.

**Subjects and Methods:** The development of the healing function is inseparable from the elaborate design and construction of its landscape space and atmosphere. The article introduced Herman. Schmitz’s new phenomenological concept of body economics to study the psychological mechanism of the healing function of landscape space in wellness homestays. In that case, the carefully created landscape space and atmosphere can effectively adjust and intervene in the structural and dynamic characteristics of the occupant’s body economics, so that the occupant’s body economic structure and dynamic characteristics can maintain a good state of tension, and can achieve a smooth transition between narrow atmosphere and wide atmosphere.

**Results:** In an ideal state, not only the indoor and outdoor environment and landscape space of wellness homestay are carefully designed, organic agriculture, fishery, gardening and traditional handicraft practices are carefully organized and carried out, but also the rich regional culture, traditional festival folk custom, lifestyle, as well as the rich ethnic culture play a vital role in the atmosphere of wellness homestay space.It also has an important impact on the health function of homestays.

**Conclusions:** All these colorful original and present elements, through the effective adjustment and intervention of the economic structure and dynamic characteristics of the body in the process of the restoration and liberation of the subject personality of the residents, can help them to restore a good balance and tension state, complete the smooth transition between the restoration and liberation of the subject personality, and finally realize the healing function of the landscape space of the residential health.This paper is an in-depth study of the complex psychological mechanisms in this process.

### Acknowledgments

This work was supported by Philosophy and Social Science Planning Project in Hainan Province (HNSK(ZC)21-163).

## LHMM23120 THE VALUE-ORIENTED FUNCTION OF MENTAL HEALTH EDUCATION UNDER THE IDEOLOGICAL AND POLITICAL IDEA FOR PATHOPHYSIOLOGY

Qiong Wang^a,b^, Yanhua Zhang^c^, Chengfan Du^a^, Shijun Xi^a,d^, Xiaochun Peng^a,e^

*^a^ Department of Pathophysiology, School of Basic Medicine, Health Science Center, Yangtze University, Jingzhou, Hubei 434023, China, ^b^ Department of Nursing, Health Science Center, Yangtze University, Jingzhou, Hubei 434023, China, ^c^ Department of Physiology, School of Basic Medicine, Health Science Center, Yangtze University, Jingzhou, China, ^d^ Shanghai Zhaxin traditional Chinese western medicine hospital, Jing’an District Shanghai, 200435, China, ^e^ Laboratory of Oncology, Center for Molecular Medicine, School of Basic Medicine, Health Science Center, Yangtze University, Jingzhou, Hubei 434023, China.*

QW and YZ contributed equally to this study.

**Background:** The ideological and political education (IPE)as an integral part of higher education is faced with different challenges amid this modern era of globalization. Therefore, the integration of IPE into the whole subject teachings, the so-called curriculum ideological and political education (CIPE)is an effective way to solve this problem. This study is the first to properly introduce the concept and history of curriculum ideological and political education in China. Additionally, it also analyzes the evaluation of students’ psychological state and the importance of mental health education for the implementation of the curriculum IPE.

**Subjects and Methods:** For those purpose, a total of 211 research subjects from Yangtze university are investigated to report the students’ motivation and behavior toward the ideological, political, and pathophysiology courses Furthermore, a questionnaire survey is used to explore the relationship between pathophysiology and ideological and political courses, and their satisfaction with the implementation of ideological, and political courses in pathophysiology and mental health education importance.

**Results:** The results demonstrate that there are significant differences between educational background and the degree of interest in ideological and political courses, gender differences, and the feedback data on the learning of professional courses. Postgraduate students are more interested in the ideological and political courses, whereas the females report that it is difficult to study pathophysiology; In addition, the results of one-way ANOVA show that the implementation effect of IPE in the pathophysiology curriculum depends on the degree of interest in IPE and pathophysiology curriculum, the degree of considering the importance of professional courses, the professional harvest after learning pathophysiology, and the degree of understanding the relationship between IPE and CIPE. A total of 81.04% of the students believe that the teachers telling stories themselves is the most popular way to spread education in the process of CIPE. This reflects the importance of mental health education from the perspective of CIPE. Additionally, this study also implies that PBL and flipped classroom teaching modes are the popular modes of teaching for CIPE.

**Conclusions:** This study is conducive to promoting the improvement and implementation of CIPE with mental health education in higher education courses; thereby, providing valuable insights for the educational decision-makers.

### Acknowledgements

This work was supported by a project grant from National innovation and entrepreneurship training program for College Students (grant no. 202010489017), Hubei Province Natural Science Foundation of China (grant no. 2017CFB786), the Scientific Research Project of Education Department of Yangtze university (grant no. JY2020134), the Curriculum Ideology and Politic Project of Graduate School of Yangtze University (grant no. YSZ202111), and Yangtze University 2022 Full-English Teaching Course Construction Project(grant no. 202215).

## LHMM23121 THE AESTHETIC PSYCHOLOGICAL NEEDS OF COLLEGE STUDENTS: AN EXAMPLE OF RELIEVING STUDENTS’ ANXIETY IN READING AND ANALYZING THE SPATIAL IMAGES IN BAI XIANYONG’S NOVELS

Yongli Tan^a^

*^a^ Department of English, Chengdu University of Technology, Chengdu, Sichuan 510059, China.*

**Objective:** To investigate the efficacy of aesthetic education in reading and writing through the spatial image description reading of a famous writer’s novels, and to analyze the college students’ aesthetic psychological needs when they are in face of anxiety.

**Methods:** First, close-reading is used in the interpretation. Bai Xianyong’s novels have their own narrative discourse aesthetic feeling. The artistic characteristics are mainly elegant, light and gentle, delicate and sensitive, and peeling off impurities. There is a sense of loneliness in the works, showing a unique aesthetic style. From his works to observe the presentation of aesthetic style, spatial image is an indispensable means of expression. Second, observation is used in the classroom teaching. During a semester, the author systematically observed, recorded and analyzed students’ mood changes in reading Bai Xianyong’s novels in order to obtain the relationship of aesthetic reading and relieving the students’ reading and writing anxiety. The author tried to keep objective and maintain the natural exposure of the observed students, and the obtained results were relatively effective.

**Results:** In the reading and writing, there were a comparatively focused time out of interference and the participation of the students was full. And through the close-reading and observation, the author found the students’ anxiety was relieved when the teacher served them novels with beautiful descriptions, and their aesthetic psychological needs were largely satisfied. Since novels have integrated the author’s observer perspective, creative expression and the aesthetic consciousness of the readers’ imagination, aesthetic reading before writing is an effective way to overcome writing anxiety.

**Conclusions:** I suggest that the aesthetic psychological needs are students’ innate motive for reading and writing, so meeting the aesthetic psychological needs of students have potential to assist educators to become more reflective about their full development and to assist the students to relieve the anxiety and to articulate their own reading and writing schedule. In this paper’s example, through the aesthetic interpretation of the three types of spatial images with the highest frequency in Bai Xianyong’s novels, the residence living room, the midnight park and the ballroom and the bar, the students understand the space-time dislocation, identity pursuit and uncertain fate. The students were led to discuss the relationship of psychology and metaphor, and to explore the specific aesthetic space elements such as body perception, emotion and thought in the novels. After the aesthetic analysis of this novel, the students were more active about their own readings and writings.

### Acknowledgments

This research was supported by Chengdu University of Technology “Double First-Class” Initiative Construction Philosophy and Social Sciences Key Construction Project (ZDJS202318).

## LHMM23122 SUPPLY CHAIN MANAGEMENT AND EMPLOYEES’ MENTAL HEALTH: VIEW OF GROUNDED THEORY

Xiaobo Tao^a^, Zhifang Han^b^

*^a^ Graduate School, North China University of Technology, Beijing, China, ^b^ School of Economics and Management, North China University of Technology, Beijing, China.*

**Background:** With the development of economy and society, the personal quality of employees has been generally improved. However, many employees feel more pressure on how to achieve the best results in the competition with rising tide. Some near-morbid competition phenomena have caused many unhealthy psychological problems, such as high psychological pressure and heavy depression tendency. It is urgent for enterprises and the whole society to find correct solutions to achieve personal development, sustainable economic and social development. Sustainable supply chain management seems to be a reasonable choice. It is a modern management mode that comprehensively considers and balances economic, social, and environmental benefits. As an important part of social benefits, mental health can be maintained in a reasonable range through sustainable supply chain management.

**Subjects and Methods:** Through interviews with three companies, namely company A, company B and company C, which all belong to the manufacturing industry, this paper analyzes the interview results with View of Grounded Theory. The reason why the selected companies belong to the manufacturing industry is that the popularization of automation in the manufacturing industry has become the trend of the times, which makes employees face the major problem of how not to be eliminated in the performance appraisal. Generally speaking, it is difficult for employees to maintain a healthy psychological state under such circumstances, which makes the manufacturing industry a very suitable research object for this article. In the analysis of the interview results, this paper divided the experiment into three parts according to the grounded theory: open coding, spindle coding and selective coding.

**Results:** Based on the grounded theory, this paper sorted out 108 open coding results, 24 spindle coding results and 6 selective coding results in the interview contents. This paper described and analyzed the spindle coding results and the selective coding results to ensure that the sources of the results are true and credible, and finally proposed possible research issues and research directions.

**Conclusions:** Research and analysis based on grounded theory shows that sustainable supply chain management will effectively improve the psychological health of employees. This will require enterprises to consider and discuss multiple aspects when formulating sustainable supply chain management strategies, and develop more diversified sustainable supply chain management strategies, so as to achieve the improvement of multiple benign indicators and benefits, and ultimately help employees and society achieve healthy and sustainable development.

## LHMM23123 THE INTEGRATION OF INNOVATION AND ENTREPRENEURSHIP EDUCATION AND PROFESSIONAL EDUCATION IN CHINESE APPLICATION-ORIENTED UNIVERSITIES AND ITS INFLUENCE ON STUDENTS’ MENTAL HEALTH

Mingzhu Zhu^a^

*^a^ Innovation and Entrepreneurship Development Center, Tianjin Sino-German University of Applied Sciences, Haihe Education Park, Tianjin 300350, China.*

**Background:** To realize the development of innovation and entrepreneurship education in Chinese application-oriented universities, the integration with professional education is an important way to cultivate the entrepreneurship of students and promote the higher quality employment of graduates. In recent years, outstanding achievements have been made in the theoretical research and practice of innovation and entrepreneurship education in Chinese universities. However, China’s innovation and entrepreneurship education is still in the exploration stage, especially in the integration with professional education. The separation has a negative impact on students’ innovation and entrepreneurship education. Moreover, it is unknown to what extent the impact on students’ mental health. This paper tries to comprehensively analyze the current situation, problems and results of the integration of innovation and entrepreneurship education and professional education. The corresponding solutions are proposed to help students complete better innovation and entrepreneurship education and to play a positive role in students’ mental health.

**Subjects and Methods:** This paper adopts the case analysis method and the questionnaire survey method. Taking an application-oriented university as a case, this paper analyzes the status in the integration of this university to find the phenomena and problems in the practice of innovation and entrepreneurship education in universities. Students are the important subjects and direct beneficiaries of education. Students’ subjective feelings and objective needs are both important factors influencing education. The actual impact on the mental health of the students is analyzed through the questionnaire survey of the students in this university.

**Results:** The integration model of innovation and entrepreneurship education in application-oriented universities is proposed from the perspective of universities, government and enterprises based on the symbiosis theory. An application-oriented university is taken as a case to explore the mechanism of innovation and entrepreneurship education, and the successful experience in the integration of education and its impact on students’ mental health. Finally, three suggestions are put forward for the effective integration of innovation and entrepreneurship education and professional education in Chinese application-oriented universities. This also can play a positive role in students’ mental health.

**Conclusions:** The development of innovation and entrepreneurship education in universities should be deeply integrated with professional education. The universities, government and enterprises, all three should participate in the whole development of innovation and entrepreneurship education. The integration of innovation and entrepreneurship education and professional education in application-oriented universities can cultivate students’ entrepreneurship better, help students to complete employment better, and help students’ to improve in mental health better.

### Acknowledgments

This work was funded by achievements of the key research project of Tianjin education (JYDY-20223006) and the scientific research project of Tianjin Sino-German University of Applied Sciences (zdkt2021-013).

## LHMM23124 AN ANALYSIS OF THE MORAL JUDGEMENT ABILITY OF PSYCHOPATHS BASED ON PSYCHOLOGICAL AND NEUROSCIENTIFIC EXPERIMENTAL RESULTS

Xinyi Zhang^a^

*^a^ School of Marxism, China University of Political Science and Law, Beijing, China.*

**Background:** To investigate whether the psychopathic agents who have deficits in emotional processing and inhibitory control are really competent with moral concepts and can make genuine moral judgements as non-psychopathic agents do, and to defend a specific kind of ethical view based on the analysis of relevant results came from psychological and neuroscientific experiments.

**Subjects and Methods:** A certain number of representative existing moral psychological experiments were discussed and analyzed with an eye to arguing for and against relevant competing views about moral concepts and judgements, i.e. the judgmentalist view and the motivationalist view. These experiments including roughly three types, among which the first type is the experiments that using the moral/conventional distinction to test whether psychopaths have the ability to distinguish between moral transgressions and conventional transgressions, the second type is the experiments that using moral dilemmas such as the trolley problem constructed by philosophers to test whether psychopaths can properly distinguish personal harms from impersonal harms. Two phenomena could be observed from these empirical tests, i.e. firstly, psychopaths and non-psychopaths make different judgements with regard to personal aggressions; secondly, both psychopaths and non-psychopaths show the capacity to distinguish personal aggressions from impersonal aggressions. Since the judgmentalist view and some sophisticated version of motivationalist view could accommodate the two phenomena equally well, a third type of psychological experiments was appealed to, i.e. the neuroscientific experiments that using brain imaging techniques such as functional magnetic resonance imaging (fMRI) to reveal patterns of activation in cognitively-relevant areas and emotionally-relevant areas when psychological and non- psychological subjects are presented with moral dilemmas and make moral judgements as well as judgements about how to act. The data from fMRI scans were then used to support the judgmentalist and emotionist hypothesis about moral judgements.

**Results:** The brain imaging experiments showed that, with regard to judgements about the moral properties of relevant actions and judgements about degrees of severity of moral violations, there was some kind of symmetry in psychopaths’ mental mechanisms as well as in non-psychopaths’ when they make moral evaluations. Non-psychopaths’ judgments about moral properties and judgments about severity are both associated with emotion-related brain regions, while both types of judgments of psychopaths are connected to cognition-related brain regions. These two types of judgements are not actually generated by different mental mechanisms and processes.

**Conclusions:** Although there is no significant difference between psychopaths and non-psychopaths in various moral judgment results, psychopaths lack the ability to make genuine moral judgments. This gives us a good reason to support the judgmentalist and emotionist view.

### Acknowledgments

The work on this paper was supported by the Humanities and Social Science Foundation of Ministry of Education of China (Grant No. 22YJC720022).

## LHMM23125 ANALYSIS OF TOURISTS’ ANXIETY PSYCHOLOGY AND THE TREND OF DIGITAL TOURISM TRANSFORMATION AMID HUGE CRISIS

Haixia Bai^a,b^

*^a^ Tourism and Economic Management Department, Lijiang Teachers College, Lijiang 674199, China, ^b^ School of Business, Yunnan University of Finance & Economics, Kunming 650221, China.*

**Background:** Huge Crisis always brings unprecedented impact to the tourism industry, leading the theme of resilience which aims for recovery become a hot topic in tourism research. However, the tourists’ psychological resilience has been largely ignored, the mechanism of how tourists give play to their psychological resilience when facing the destination crisis is still ambiguity, and the impact on tourism industry is also unclear, forming a huge research gap. By exploring tourists’ psychological resilience, this paper aims to enrich the literature on tourism resilience theoretically, and in practice, explores the enlightenment of tourism recovery under the crisis context from the perspective of demand.

**Subjects and Methods:** Based on SOR theory and Maslow’s Needs theory, this paper adopts the longitudinal research method to collect data to track the diachronic changes of the COVID-19 pandemic situation from January 2020 to December 2022 in Lijiang, a small tourist city in southwest China that is popular with domestic and foreign tourists. The Focus group discussion (FGD) method is used to summarize the stages of tourists’ psychological changes, analyze the important role played by technology forces in this process, and predict the trend of digital transformation in the tourism industry.

**Results:** In the early stage of the pandemic, due to the risk perception of external policies and internal loss aversion, tourists show travel fear or travel avoidance behavior out of the motivation of seeking safety. In this stage, they think highly of digital based countermeasures to ensure health and safety. In the middle stage, the depressed tourism demand seeks to release. On the one hand, tourists flow to local tourism markets such as travel around, suburban tourism, rural tourism and micro vacation, increasing the stable closed-loop circulation capacity of the local market. On the other hand, they pursue new forms of digital tourism, which can relieve the suffering of “lovesickness” for tourism to some extent. In the later stage, the pandemic situation tends to ease, some tourists show a tendency of compensatory tourism and start taking “an instant trip” mode, while more tourists remain cautious and planning long distance travel. At this stage, tourism psychology shows a strong preference for social needs and shows dependence on digital technology.

**Conclusions:** Amid the crisis, tourists follow the psychological mechanism model of “Environment-perception- motivation -behavior”, showing the evolution and change law of three stages, namely, early period-travel fear, middle period-demand depression, late period-differentiation and transformation. Besides, digital technology plays an important role in each stage. From the perspective of tourists’ psychology, this paper reveals that after the crisis, the recovery of tourism industry needs a process of gradual adaptation and restart. This research also gives some advice on the research agenda of tourists’ psychological resilience in the future.

### Acknowledgements

This work was supported by a project grant from The National Social Science Fund of China (Grant No.22BGL151).

## LHMM23126 PREDICTION MODEL OF PUBLIC HEALTH EMERGENCIES BASED ON BIG DATA TO EASE PEOPLE’S PANIC

Shiying Wang^a^, Shizhen Bai^a^

*^a^ School of Management, Harbin University of Commerce, Harbin 150028, China.*

**Background:** In recent years, as COVID-19, Ebola, monkeypox and other infectious diseases continue to spread around the world, public health emergencies have become another important factor affecting people’s work and life. Public health emergencies are unexpected, unpredictable and irresistible, and they will cause devastating consequences and damage people’s body, mind, spirit and other aspects. When people face these stressful events, once they cannot solve or deal with them, serious psychological imbalance will occur, which is called crisis. Acute psychological disturbance (anxiety, depression, irritability) may occur during a crisis. The main reason for this is that people are afraid of the uncertainty and opacity of the event itself. Therefore, this paper uses the infectious disease model to establish the prediction model of public health emergencies. Through the model, the development trend of the events, mortality and other indicators are effectively predicted, so as to alleviate people’s panic.

**Subjects and Methods:** In this paper, a prediction model of public health emergencies based on SEIHD was established by improving the traditional infectious disease model and using three indicators, namely viral infection rate, virus incubation period and recovery rate. The SEIR model was improved and the parameters were modified to simulate the external intervention measures to predict the development trend of the epidemic.

**Results:** In this paper, the law of epidemic transmission in Hubei Province was taken as an example to predict the number of confirmed cases and the number of deaths in two stages. The cumulative number of confirmed cases in Wuhan from January 11, 2020 to February 18, 2020, predicted by the prediction model, and the real data during this period was fitted. The two curves were compared, showing good fitting. The data of the COVID-19 epidemic in Hubei Province from January 11, 2020 to February 18, 2020 were input into the model as the basic data to predict the death toll during this period. The data were fitted to the real data respectively. At the same time, it was assumed that quarantine measures were taken ten days in advance to predict the death toll. The same method was used to predict the number of confirmed cases and deaths from February 18, 2020 to April 14, 2020, and the results were correct.

**Conclusions:** The prediction results are basically consistent with the real data, which confirms the validity of this prediction method. The research proves that early detection, early prevention and control of public health emergencies can control their development trend, curb their widespread spread, and reduce the harmful impact on social life and economy. At the same time, it can alleviate people’s panic about the epidemic to a certain extent. So as to avoid the acute psychological disturbance caused by insufficient cognition of public health emergencies.

### Acknowledgments

This work is supported by Humanities and Social Science Project of Ministry of Education (18YJCZH175); the National Grant Natural Science Foundation of Heilongjiang Province (YQ2019G004); University Nursing Program for Yong Scholars with Creative Talents in Heilongjiang Province (UNPYSCT-2020081).

## LHMM23127 FINANCIAL SUPPORT FOR AGRICULTURAL TRANSFER POPULATION’S SOCIAL INTEGRATION TO CHINA’S NEW-TYPE URBANIZATION: A PATH DESIGN FROM THE PERSPECTIVE OF MENTAL HEALTH

Xueqing Kang^a^, Yuanxin Zhang^b^

*^a^ School of Economics, Sichuan University, Chengdu 610065, China, ^b^ Chengdu Institution of New Economic Development, Chengdu 610042, China.*

**Background:** New-type urbanization is not only the urbanization of population, but also the citizenization of agricultural transfer population and realize social psychology integration in aspects of lifestyle, cultural types, psychological resources and corresponding social structure. The psychological health level of the agricultural transfer population is a key component of promoting social integration of agricultural transfer population into cities, which can improve new-type urbanization. Compared with the expanding capital demand of these related projects, it is far from enough to rely on fiscal revenue, therefore optimizing investment and financing mechanism for urbanization and strengthening financial support for new urbanization is urgent.

Subject and Method: This study aims to analyze the agricultural transfer population’s demand of social integration to improve their psychological health level and conduct to optimize the allocation of supporting financial resources in process of new-type urbanization. By analyzing the financial support mechanism of agricultural transfer populations’ social integration from the perspective of environmental psychology, we establish a new financial support path design containing general path as well as specific path, tested by structural equation model based on panel data 31 provinces of mainland China from 2008 to 2017.

**Result:** The results show that the effect of financial support for agricultural transfer population’s social integration to China’s new-type urbanization is more significant than fiscal revenue support, and the supporting function is realized through general path and specific path respectively to ensure migrant people’s equal access to infrastructure and public services to help them to form place connection to gain social integration, which is beneficial for the agricultural transfer population to obtain a sense of happiness from urban life, thereby improving their psychological health status.

**Conclusion:** This study recommends that the enterprises engaged in agricultural transfer population employment and governments need to pay attention to their psychological health status and enhance related infrastructure and equalization of social security to help them achieve social integration. Meanwhile, it is indispensable to build a diversified financial support system in paths of entity economy development, infrastructure construction, employment, education, housing, social security to form place connection from physical environment dimension and solve the recent financial support system’s problem of unitary structure, insufficient innovation and scale, which will enhance agricultural transfer population’s security sense of social security and local trust through place connection to raise their psychological health level to realize social integration.

### Acknowledgements

This paper is supported by the National Social Science Fund of China (grant number 14BJY055).

## LHMM23128 ANALYSIS OF PROMOTIONAL STRATEGIES FOR UNIVERSITY LIBRARY READING BASED ON USER PSYCHOLOGICAL NEEDS: A CASE STUDY OF SHANDONG NORMAL UNIVERSITY LIBRARY READING PROMOTION PROJECT

Jing Chi^a^, Tae-Won Kang^b^

*^a^ Department of Global Entrepreneurship, Kunsan National University Gunsan 54150, Republic of Korea, ^b^ Department of Supply Chain and Logistics, College of Social Sciences, Kunsan National University, Gunsan 54150, Republic of Korea.*

**Background:** To explore the promotional strategies for university library reading and summarize the experience gained in the course of disseminating Chinese traditional culture in the context of national reading, based on user psychological needs and relying on college student volunteers.

**Subjects and Methods:** In 2009, the Library of SDNU analyzed user needs, refined its collection advantages, and took the dissemination of traditional Chinese culture as a starting point to bring college student volunteers into the role of reading promoters, gained support from the university as well as the social forces, uniformly planed marketing development and focused on the impact assessment. In this article, the specific case of the SDNU Library Reading Promotion Project “Promoting Volunteerism and Reading to the N-th Power” was analyzed, and the origin, goals, products, evaluation and main processes of project were introduced.

**Results:** The reading marketing project “Promoting Volunteerism and Reading to the N-th Power” was planned and organized by the Library of SDNU from 2009. The inspiration for the project derived from the classic book A Single Spark Can Start A Prairie Fire by Mao Zedong, the great leader of China. Since the launch of the project, the Library of SDNU has been awarded the national-level “Demonstration Base for National Reading” (at the team level); the university student volunteers in the library have won three gold awards in the three major categories of university student reading promotion nationwide (at the individual level); the cooperation between the Library of SDNU and public welfare organizations and social enterprises has become increasingly close (at the marketing level); the satisfaction survey of readers among the faculty and students at SDNU Library has ranked first for consecutive years, and the number of social readers following their new media platform for marketing projects has been increasing year by year (at the level of user psychological needs). The volunteer reading promotion project for college students in the Library of SDNU has been reported by many media outlets such as Renmin.com, Dazhong.com, Fenghuang.com, Qilu Evening News, and Life Daily, and has aroused strong social repercussions (at the social level). The “Promoting Volunteerism and Reading to the N-th Power” reading marketing project of SDNU Library is gradually realizing the potential of starting a prairie fire with a single spark, taking university libraries as the front line.

**Conclusions:** This project, based on users’ psychological needs, leveraging college student volunteers to promote traditional Chinese culture in university libraries, is a positive manifestation of the views and attitudes of the International Federation of Library Associations and Institutions (IFLA) strategic plan. The project is conducive to the internationalization and branding development of Chinese library reading marketing projects and the establishment of a marketing system for the global dissemination of traditional Chinese culture through university library systems. The design and implementation of the project’s working methods have a close social, school, and personal background. The Library of SDNU has actively responded to the National Reading initiative by cultivating excellent reading promotion projects that can be used for reference and easy to replicate, spreading the permanent charm of Chinese culture and the style of the times, which is conducive to enhancing the reading happiness of people at all levels of society, including entrepreneurs, students, farmers, and other groups; and by cultivating excellent reading promoters, giving full play to the advantages of peer partners, and achieving positive self-development through mutual assistance and cooperation, which is conducive to the unification of the “self-value” and “social value” of the personality of the Chinese youth generation.

## LHMM23129 RESEARCH ON THE INFLUENCE OF OUTWARD-BOUND TRAINING ON THE WILL QUALITY AND MENTAL HEALTH OF CUBA ATHLETES

Lancang Wang^a^

*^a^ Beijing University of Chemical Technology, Beijing 100029, China.*

**Background:** According to a survey of 126000 college students in China, it was found that 20.23% of college students have psychological disorders, 11.7% of college students have different degrees of psychological diseases, and 75% have experienced psychological crisis. Will quality is an important part of athletes’ competitive ability, a necessary guarantee for their excellent performance, and also the embodiment of sports spirit and sports culture, especially for college athletes at the growing stage. At the same time, mental health is of great significance for college athletes to resist mental diseases, improve learning efficiency, complete training tasks, and improve interpersonal communication and quality of life.

**Subjects and Methods:** This paper uses the methods of literature, experiment and mathematical statistics to study the CUBA players of several universities in Chaoyang District of Beijing. The experiment was divided into two groups. The experimental group added outward bound training to the training plan, while the control group carried out according to the original training plan. Before and after the experiment, the professional psychological scale was used to measure and record the various indicators of the subjects for analysis and research.

**Results:** Through theoretical analysis and experimental research, outward bound training plays a positive role in the cultivation of CUBA athletes’ mental health. The experimental research shows that the addition of outward bound training in the training has a positive effect on the cultivation and improvement of the will quality of college students’ athletes. Although this improvement is unbalanced, there is no significant difference in the three aspects of hardiness, tenacity and goal clarity from the statistical point of view, but it is very obvious in the three aspects of decisiveness, self-control and self-confidence (P<0.05).

**Conclusions:** It is suggested to pay more attention to and cultivate the mental health level of college athletes. It is suggested that university sports teams should selectively integrate part of outward-bound training into the training of sports teams according to their own conditions, so as to improve the mental health level and willpower quality of athletes. And gradually establish and improve the curriculum system in line with the training of various projects of the university based on their own needs.

## LHMM23130 FRANCHISING WITH CHINESE CHARACTERISTICS: CULTURAL AND INSTITUTIONAL DIMENSIONS AND MENTAL HEALTH OF PRACTITIONERS

Xiaoxi Sun^a^, Ling Miao^b^, Yanjie Wang^c^

*^a^ The CPC Henan Provincial Party Committee Party School, Zhengzhou, Henan 451464, China, ^b^ The CPC Henan Provincial Party Committee Party School, Zhengzhou, Henan 451464, China, ^c^ College of Humanities and Development Studies, China Agricultural University, Beijing 100089, China.*

**Background:** This article analyzes the development of franchising in China, its growing role in the Chinese economy, and how its development differs from that of developed economies. And the article attempts to analyze from three dimensions: system, culture, and practitioners’ mental health. The results show that these three dimensions will have a significant impact on franchising in China, and it is necessary to focus on these three aspects when formulating policies and implementing them in the future. In particular, the psychological health of practitioners is closely related to the future sustainable development of franchising operations to a certain extent. Therefore, it is necessary to conduct necessary intervention and guidance on the psychological health of practitioners to ensure that they can maintain a good psychological state to carry out their daily work. Franchising in China has shown great advantages and taken a crucial role in the rapid growth of the Chinese economy. While few scholars have explained the unique practices of franchising in the Chinese context. Franchising has become a significant business model in China after reform and opening up as in Western countries.

**Subjects and Methods:** This article is organized as follows. First provide a brief review of the franchising around the world and then discuss the trends of franchising development in China. With a comparison of law and regulation, economy, and culture, the institutional and cultural differences in China are analysed. After that, this article summarize the unique characteristics of franchising in China and discuss how franchising works differently in China. Finally, the authors identify special issues and shed new light on the existing franchising literature in the emerging business environment.

**Results:** Present the growth of franchising in China as a business model relative to the West. The cultural and institutional characteristics compared with the West make franchising in China different. Successful franchises in China have less reliance on contracts and more on trust and personal ties between franchisor and franchisee.

**Conclusions:** In general, franchising will become increasingly accepted and encouraged because the arrival of franchises contributes to the growth of the economy in China. Since the first pilot franchise regulations were adopted in the late 1990s, domestic franchises have taken off with the growing purchasing power gained by Chinese consumers. In addition, universal cultural trends allow many international franchises to enter China. As in Western economies, both international and domestic franchise chains have become a significant business model in China and have grown substantially over the past twenty years. Although this competitive business model has received a surge of scholarly interests, research in this area has sought to provide a franchising model from the Western perspective. The public’s understanding of franchising in China is low. This research focuses on the differences in franchising in China. Franchising in China has developed specific characteristics compared with the West, which is by driven institutional and cultural differences, as well as China’s level of development. Built on trust, a franchisor-franchisee relationship has the requisite guanxi for business success in China. The implications can be applied to some Asia countries with similar cultural backgrounds. Future studies are encouraged to compare the development of franchising in emerging business landscapes, which accounts for most of the world’s population and over half of the world’s natural resources.

### Acknowledgements

Soft Science Project: Challenges and Countermeasures for the Digital Transformation of Traditional Industries in Henan Province under the “Double Carbon” Goal (232400410201).

## LHMM23131 IMPACT OF GRATITUDE ON COLLEGE STUDENTS’ MORAL JUDGMENT: A STUDY FROM THE PERSPECTIVE OF EMOTIONAL REGULATION

Xiang Li^a^, Min Zhang^a^

*^a^ School of Education, Huaibei Normal University, Huaibei 235000, Anhui, China.*

**Background:** The emotion of gratitude reflects the level of individual mental health by affecting moral judgment. This study aims to investigate the impact of gratitude on college students’ moral judgment ability.

**Subjects and Methods:** In experiment 1, the psychological experimental programming software E-prime was used to compile moral word judgment task for pre-post test. Experiment 2 adopted a 2 × 2×2 experiment design between subjects, which revealed the influence mechanism of gratitude on college students’ moral judgment by examining three variables: gender, gratitude degree (Level of mental health) and whether one word is moral word or not.

**Results:** It was found that: (1) the short film used in the study could effectively induce the subjects’ gratitude (t=3.53, df=31, p<0.01); (2) College students displayed significant gender difference in moral word judgment task (F=4714254.5, p<0.05); (3) College students’ gratitude degree exerted significantly different impact on moral word judgment task (F=8730198.546, p<0.001); (4) College students’ gender and gratitude degree exerted significant interaction effect on moral word judgment task (F=2842006.648, p<0.001). Conclusions: Gratitude (level of mental health) has a significant impact on college students’ moral judgment. High gratitude (High level of mental health) individuals have more active cognitive processing, resulting in greatly improved quality in thinking and decision-making. Also, such individuals have higher moral judgment level, showing greater positivity than low gratitude (Low level of mental health) individuals in moral judgment.

### Acknowledgments

The paper was supported by Study on Mental Health Promotion Model for Special Education Teachers Based on Mental Resilience (Project No.: 23500024).

## LHMM23132 EFFECT OF ACYCLOVIR ON PROTEOMICS OF SH-SY5Y CELLS INFECTED WITH HSV-2

Yangfan Wu^a^, Lin Kuang^a^, Tingyu Xu^a^, Wei Lu^a^, Jialing Shi^a^

*^a^ Hunan University of Chinese Medicine, Changsha, Hunan, China*

**Background:** Genital herpes (GH) is a common sexually transmitted disease mainly caused by herpes simplex virus type 2 (HSV-2), which lurks innerve roots and causes recurrent disease. Currently, there is no cure for GH, but nucleoside antiviral drug can inhibit viral replication,and acyclovir is one of the drugs commonly used in clinic to improve its symptoms.

**Subjects and Methods:** The effect of acyclovir on HSV-2 infected human neuroblastoma cells (SH-SY5Y) was studied by label-free quantitative proteomics in order to explore the action mechanism of acyclovir on HSV-2 virus lurking in neurons. In this study, we treated SH-SY5Y cells infected with HSV-2 virus with acyclovir drug-containing serum. After modeling, extracted cell proteins, detected protein expression differences using label-free quantitative proteomics, and analyzed the GO functional enrichment, domain enrichment and transcription factor annotation of these differential proteins.

**Results:** The results showed that 1011 proteins were involved in the intervention of acyclovir against HSV-2 virus lurking in neurons, of which 513 were up-regulated and 498 were down-regulated. These proteins were mainly concentrated in biological processes, cell composition and molecular function. The results of GO functional enrichment analysis, domain enrichment analysis and transcription factor analysis of differential proteins showed that, the potential targets of acyclovir are mainly concentrated in the nucleus and mitochondria, acyclovir mainly acted on mitochondrial carrier domain, ATPase conserved sites and annexin sites, and acyclovir mainly interfered with HMG-box, zf-C2H2, CSD, Homeobox and other transcription factors to inhibit the replication of HSV-2 virus.

**Conclusions:** Acyclovir interferes with the replication of HSV-2 virus in cells by affecting the catabolic process of nuclear transcription mRNA, mitochondrial respiratory function and ATPase activity.

### Acknowledgments

This study was supported by grants from Project supported by the National Natural Science Foundation of China (Grant No.82174391); Chinese Medicine Scientific Research Foundation of Hunan (Grant No.2021058).

## LHMM23133 FINGERTIP TIBETAN FLOWER OF RELIEVING ANXIETY AND IMPROVING MENTAL HEALTH: PRACTICAL RESEARCH ON TRADITIONAL CRAFTS IN THE ORGANIC STREET CONNECTION OF POVERTY ALLEVIATION AND RURAL REVITALIZATION

Yulan Zhou^a^

*^a^ Academy of Fine Arts, Sichuan Minzu College, Kangding, Sichuan 626001, China.*

**Background:** Tibetan embroidery of Relieving anxiety and improving mental health is facing the inheritance crisis such as the fault of the inheritance population, the increase of Tibetan embroidery substitutes and the decrease of public acceptance. Promoting the industrialization development of Tibetan embroidery skills with the integration of culture and tourism; drive the personalized development of Tibetan embroidery skills with innovative ideas; drive the intensive development of Tibetan embroidery skills with capable craftsmen; promote the socialized development of Tibetan embroidery skills with targeted poverty alleviation.

**Subjects and Methods:** Conduct empirical research in Tibet-related areas.

**Results:** How Tibetan embroidery can help poverty alleviation and rural revitalization under the consciousness of building a Chinese national community.

**Conclusions:** Carefully summarize and promote the experience of poverty alleviation, and continue to consolidate the stable employment of the helpers; fully implement the revitalization of Tibetan embroidery, focusing on strengthening the integrated development of Tibetan embroidery tourism; focus on building the development of the whole industry chain of Tibetan embroidery, and promote the revitalization of Tibetan embroidery industry to empower rural areas; adhere to innovation to lead the living inheritance of Tibetan embroidery and expand the market consumption space of Tibetan embroidery products; guide college students with relevant ambitions to tap the cultural and creative resources of Tibetan embroidery, highlight the advantages of manual creation, and finally realize that college students can promote employment through entrepreneurship and alleviate the pressure of social employment. Tibetan embroidery plays an increasingly important role and shoulders the mission and responsibility from poverty alleviation to rural revitalization. The internal logical mechanism between the two requires Tibetan embroidery to take the initiative to take advantage of the development of the rural revitalization strategy. It is necessary to strengthen the inheritance education of Tibetan embroidery, enhance the consciousness and self-confidence consciousness of the inheritance subjectivity, reshape the self-respect and self-confidence of its own historical and cultural characteristics, cultivate the endogenous motivation of rural revitalization, and awaken the road consciousness and road self-confidence of Tibetan compatriots to conspire with their own national characteristics. We should also adhere to innovative development, live inheritance, fully tap the profound cultural heritage and unique capitalization transformation advantages of Tibetan embroidery, enhance the potential industrial development ability of Tibetan embroidery in various forms, and promote the integration of Tibetan embroidery into modern life on the basis of maintaining the national style and local characteristics of the original process.

### Acknowledgments

This work was supported by 2021-2023 Sichuan Higher Education Talent Training Quality and Teaching Reform Project: Theoretical and Practical Research on Teaching Reform of Building the Chinese National Community Consciousness Education in Universities in Ethnic Areas (JG2021-1417); National Ethnic Affairs Commission’s Teaching Reform Project “Research on Building Chinese National Community Consciousness into the Ideological and Political Curriculum Construction of Ganzi Universities” (ZL21064);Sichuan Minzu College 2021-2023 Higher Education Talents Training Quality and Teaching Reform Project ‘ Research and Practice of Diversified and Innovative Talents Training of Tibetan Folk Crafts’ (JG202111).

## LHMM23134 ANALYSIS OF THE IMPACT OF PERFORMANCE APPRAISAL ON CROSS-PLATFORM SERVICE PREFERENCES FROM THE PERSPECTIVE OF IMPROVING EMPLOYEE PSYCHOLOGICAL QUALITY

Wei Shang^a^, Feng Luo^b^, Zhe Wang^b^

*^a^ School of Management, Chengdu University of Information Technology, No.24 Block 1, Xuefu Road, Chengdu, China, ^b^ School of Economics and Management, Sichuan Tourism University, No.459, Hongling Road, Chengdu, China*

**Background:** As a new form of team joint, cross-platform service emerges at the moment and gets good feedback from the market. In recent years, performance appraisal as an environmental cue has been widely concerned by the academic community. However, the impact of performance appraisal on cross-platform services is unknown, especially given the psychological awareness and stress of employees, which is undoubtedly an urgent issue for enterprises to understand.

**Subjects and Methods:** Based on the two-factor theory, this paper explores the influence of performance appraisal on cross-platform service preference and its internal mechanism, and explores the moderating effects of different types of performance appraisal (individual appraisal and team appraisal) in this process. Through 4 experiments, experiment 1 is a field experiment between single factor groups, experiment 2 is a laboratory experiment between single factor groups, experiment 3 is an online questionnaire between single factor groups, experiment 4 is a 2 × 2 field experiment, the research method of combining field experiment and laboratory experiment is adopted, and the research design supported by actual service evaluation and questionnaire measurement is adopted. The variance analysis and bootstrap method were used to examine the internal mechanism of performance appraisal’s preference for cross-platform services through psychological stress and anxiety.

**Results:** The results show that experiment 1 verifies that compared with non-performance appraisal state, employees in performance appraisal state show lower preference for cross-platform services. In experiment 2, other competitive explanation mechanisms were excluded and it was again verified that, compared with non-performance appraisal state, employees were less likely to prefer cross-platform services in appraisal. Experiment 3 verifies that the two-factor theory plays a psychological mediating role in the relationship between performance appraisal and cross-platform service preference, and clarifies the internal two-factor psychological cognition mechanism of this relationship. In experiment 4, the experimental situation was changed, that is, the performance appraisal level was divided into assessment and non-assessment, and the assessment type was divided into individual assessment group and class assessment group, which verified the rationality of the two-factor theory as the internal mechanism of performance appraisal for cross-platform service preference. At the same time, it is found that the performance appraisal type (individual and team) has a moderating effect on the influence of performance appraisal on cross-platform service preference and the mediating effect of the two-factor theory.

**Conclusions:** The research results theoretically expand the relevant theoretical research on job assessment, employee psychology, service support and manufacturer association. At the same time, in practice, it not only provides feasible suggestions for the work arrangement and psychological pressure relief of employees in manufacturing enterprises, but also provides valuable suggestions for service providers to carry out manufacturer association and cross-platform service recommendation. Therefore, this paper has important theoretical contribution and practical significance.

## LHMM23135 THE ROLE OF CHILD-PUGH GRADING IN THE SURGICAL TREATMENT OF LIVER CIRRHOSIS WITH GALLSTONES IN ELDERLY PATIENTS

Yuxia Zhu^a,b,c^, Nan Kang^a,b,c^, Wenjuan Yan^a,b,c^

*^a^ People’s Hospital of Ningxia Hui Autonomous Region, Yinchuan, Ningxia 750001, China, ^b^ Ningxia Eye Hospital, Yinchuan, Ningxia 750001, China, ^c^ The First Affiliated Hospital of Northwest University for Nationalities, Yinchuan, Ningxia 750001, China.*

**Background:** To investigate the effect of Child-Pugh grading standard in the surgical treatment of liver cirrhosis with gallstones in elderly patients.

**Subjects and Methods:** This retrospective analysis selected 68 elderly patients of liver cirrhosis with gallstones treated in Ningnan Hospital (One Branch of People’s Hospital of Ningxia Hui Autonomous Region, China) from February 2021 to February 2022. According to Child-Pugh grading criteria and the individual liver reserve function, all patients were divided into three grades, grade A, B and C. Then, patients were classified into different groups according to varies surgical methods. 33 patients were in the first group which following laparoscopic cholecystectomy (named LC), 28 patients were in the second group which following traditional laparotomy for cholecystectomy (named OC), while 7 cases were in third group for mixed ways (named LC + OC). All the patients were confirmed by CT and MR. The effectiveness results of 68 elderly patients with cirrhosis complicated with gallstones after different surgical treatment were analyzed.

**Results:** Child-Pugh grading on a scale of 5 to 7 is the safest range for the three types of surgery. When the score is higher than 10, it implicates a great risk, and surgical treatment should be carefully selected according to the patient’s condition in these cases. The 33 patients in LC group recovered quickly and were discharged from hospital on the 5th day after surgery. Among the 28 patients in the OC group, the condition recovery was slower than that in the LC group, and 4 patients had mild complications and extended hospitalization for 3 days. Among the 7 patients in LC+OC group, due to their serious condition, 1 patient had complications during the recovery period and was hospitalized for 5 days longer. Another 1 patient’s condition deteriorated and died 2 days after surgery in LC+OC group. The study also conducted a followed up process, 67 patients were discharged and followed up, 11 of which were re-hospitalized.

**Conclusions:** Child-Pugh grading criteria can help doctors effectively evaluate the liver reserve function of patients, and has a guiding effect on the rehabilitation of elderly patients with liver cirrhosis complicated with gallstone after surgical treatment. Our research finds that Child-Pugh grading can reduce the risk of surgery, shorten the length of hospitalization, reduce the re-hospitalization rate, prevent complications and improve the quality of life of the patients.

### Acknowledgments

This work was supported by a project grant from Major R & D Plan Project of Ningxia Hui Autonomous Region (Grant No. 2017 BY 036)

## LHMM23136 THE IMPACT OF STRENGTHENING COST CONTROL ON ALLEVIATING THE PSYCHOLOGICAL ANXIETY OF ENTERPRISE MANAGERS IN THE PEARL RIVER DELTA

Deyu Chen^a^, Yiru Yang^a^

*^a^ School of Accounting, Guangzhou College of Commerce, Guangzhou 511363, China.*

**Background:** With the rapid upgrading of the industrial structure in the Pearl River Delta (PRD), the competition in the manufacturing market is becoming more and more fierce? It not only causes the psychological anxiety of the managers and employees of the enterprises in the Pearl River Delta, but also directly affects the economic benefits of the enterprises and their sustainable development in the future. At present, an important obstacle to the construction of a harmonious socialist society is the pressure and psychological anxiety of employees. The manufacturing industry in the Pearl River Delta region is developed with a large number of employees. Due to the employees’ educational background, skills and income being in a weak position, they bear more pressure and psychological anxiety than other organizations, reduce corporate profits, while bringing serious family and social problems to society.

**Subjects and Methods:** This paper discusses the current situation and causes of cost management of manufacturing enterprises in the Pearl River Delta, taking kub pen company as an example, this paper analyzes the psychological anxiety of enterprise cost managers in the Pearl River Delta by means of activity-based costing (ABC), which includes the lack of cost awareness, the lack of cost management, the lack of material management and the lack of basic work.

**Results:** The various problems in cost management of manufacturing industry in the Pearl River Delta (PRD) have resulted in the decline of the profits of manufacturing enterprises. The manufacturing enterprise has no core competitive power, and the low-cost comparative advantage is losing, the crisis is not only the production crisis, but the survival crisis, which also causes the enterprise manager’s psychological pressure to be extremely big, even part of the enterprise management because can not stand the pressure to choose to jump off the building to end life. To solve these problems, we must make a reasonable response strategy to enhance the sustained competitiveness in the whole market economy.

**Conclusions:** According to the psychological anxiety of enterprise cost managers in the Pearl River Delta, the author puts forward some countermeasures: strengthening the management of activity-based costing, establishing the management mechanism of responsibility cost, improving the quality of basic data; Develop preventive measures and strict cost accounting. By establishing a scientific and effective cost management system, the manufacturing enterprises in the Pearl River Delta can alleviate the psychological anxiety of the managers and employees, and improve the economic benefits of the manufacturing industry, thus enhance the competitiveness of enterprises in the Pearl River Delta in the regional market.

## LHMM23137 INVENTORY MANAGEMENT STRATEGY OF EMERGENCY MEDICAL SUPPLIES BASED ON MEDICAL BIG DATA

Kaige Zhou^a^, Lili Zhou^b^, Xin Xu^c^

*^a^ New Economic Reseach Institute, Ningbo University of Finance&Economics, Ningbo, Zhejiang 315175, China, ^b^ Nanchang Institute of Technology, Nanchang 330511, China, ^c^ Library of Huaiyin Normal University, Huai’an, Jiangsu, 223001, China.*

**Background:** Emergency medical supplies are a necessary material guarantee for dealing with public health emergencies. Good emergency medical supplies inventory management is an important part of emergency medical management mechanism, which will provide strong support for effective response to public health emergencies.

**Subjects and Methods:** Supply chain management of emergency medical supplies has become an important factor to reduce sudden disasters and emergency medical accidents. The optimization of medical supplies inventory can improve the level of emergency medical treatment, control the spread of infectious diseases and reduce the losses caused by infectious diseases. Medical big data shows that the formulation of emergency medical supplies inventory strategy is affected by demand, price and other factors. This paper proposes and analyzes the dynamics of a supply chain system under inventory and price factors with the feature of piecewise linearity. This switched linear model composed of four subsystems extends the classic inventory models under inventory. Based on properties of switched model, some analytical stability results are derived, and the global dynamic behavior is theoretically examined. Simulation experiments are designed to show the stability results and observe other nonlinear phenomena. It is illustrated that the inventory-dependent demand function and the forecasting inventory rate combine to bring about the stability result or arise more complicated nonlinear dynamics, such as chaotic and periodic fluctuations of inventory and order.

**Results:** These results are suitable for understand the dynamic complexities of supply chain system and present the direction for choosing the decision parameters to improve overall performance. Some illustrative examples demonstrate the effectiveness of the obtained scheme.

**Conclusions:** Efficient inventory management based on medical big data can flexibly respond to the rapidly changing demand of medical supplies in an emergency epidemic, so as to improve the accuracy and timeliness of medical assistance, promote the early control of the emergency, shorten the spread time of the emergency, and reduce the harm caused by the emergency to the society. It has positive significance for the rapid elimination of major emergency after the outbreak, and is worthy of promotion.

### Acknowledgements

This work was supported by a project grant from the 2021 Annual Project of “the Chinese Society for Technical and Vocational Education” & “New-Era TVET Institute of China”, Historical Mission and responsibility of vocational education in the construction of Healthy China (Grant No.SZ21A003).

## LHMM23138 BODY FAT PREDICTION ALGORITHM OF EXTREME LEARNING MACHINE BASED ON PARTICLE SWARM ALGORITHM OF TENT MAPPING

Guangwei Zhang^a^, Yuanhui Cui^a^

*^a^ School of Information Science and Engineering, Dalian Polytechnic University, Dalian, Liaoning 116034, China.*

**Background:** To explore methods of body fat measurement and improve the accuracy of body fat measurement. It is of great significance for body shape management, monitoring human health, and preventing diseases such as hypertension and hyperglycemia.

**Subjects and Methods:** An impedance measurement system was designed based on a human body impedance measurement model. Sixty college students were randomly selected to measure their body fat ratio, body impedance, height, weight, age, and gender to form 60 sets of data sets. Use MATLAB software to build an extremum learning body fat prediction model based on tent mapping and particle swarm optimization (TPSO-ELM). The traditional extremum learning machine (ELM) model and standard particle swarm optimization extremum learning machine (PSO-ELM) model were set as control experimental groups. Then select 50 sets of data from 60 sets of data as the training dataset, and the remaining 10 sets of data as the test dataset.

**Results:** Observe the extremum learning body fat prediction model based on tent mapping and particle swarm optimization and the broken line chart of the body fat prediction results of the control group. The predicted results of the extremum learning body fat prediction model based on tent mapping and particle swarm optimization have the best fit with the true value curve. Calculate the absolute error of the extremum learning body fat prediction model based on tent mapping and particle swarm optimization and the control group. Then calculate the average of the absolute errors of 10 sets of data. The average absolute error of the predicted value of the extremum learning body fat prediction model based on tent mapping and particle swarm optimization was 0.0085, compared to 0.22 and 0.023 in the control group. Therefore, the body fat prediction algorithm of extreme learning machine based on particle swarm algorithm of tent mapping proposed in this paper improves the accuracy of body fat measurement.

**Conclusions:** This paper proposes an extremum learning body fat prediction model based on tent mapping and particle swarm optimization. Human impedance, height, age, weight, and gender were used as input parameters for the body fat prediction model. The simulation experiment proves that the new body fat prediction model proposed in this paper improves the accuracy and efficiency of body fat prediction. Body mass index (BMI) is currently the standard recommended by the World Health Organization (WHO) to evaluate body fat and lean. The experimental data were validated according to the obesity and obesity standards established by the World Health Organization, and the results showed good consistency. The fitness prediction algorithm proposed in this paper improves the accuracy of body fat prediction to a new level. The predicted body fat by this method is more useful in monitoring human health and effectively preventing diseases.

## LHMM23139 DESIGN OF FOOD SPOILAGE DETECTION CIRCUIT BASED ON HEALTH MANAGEMENT

Guangli Long^a^

*^a^ School of Physics and Telecommunication Engineering, Shaanxi University of Tecnology, Hanzhong City 723001, China.*

**Background:** The purpose of health management is to prevent and control the occurrence and development of diseases, to reduce the medical cost and to improve the quality of life, and health risk factors associated with their lifestyle, through health information collection, health testing, health assessment, personalized monitoring management programs, health interventions and other means to continue to improve the process and methods. The problem of food safety is a key issue concerning human health, the most important part of which is the detection of food spoilage, spoilage and deterioration of food, regardless of sensory or physical and chemical properties, it is very important to detect the level of food spoilage quickly and accurately.

**Subjects and Methods:** In view of the current problems is people’s concern about the relationship between food and human health, as well as the harmful effects of rotten food on the human body, etc., a kind of food spoilage detection circuit based on health management is designed, The circuit is based on STC12C5A60S2 microcontroller using resistance detection network and moisture sensors to collect data,DHT11 temperature sensor is used to measure the temperature, test results are displayed by the LCD12864 and broadcast by the voice broadcast module SYN6288, and several kinds of normal food standard values are stored in the microprocessor, kinds of food is replaced by pressing button.

**Results:** The experiment proved that the circuit can realized the normal measurement of food. Measurement time, food resistance and moisture as well as the current room temperature can be displayed on the display, the test results whether the food consumed can be broadcasted.

**Conclusions:** Health management is the use of information and medical technology, a complete, thorough and personalized service procedures are built on health care, medical science, its aim is to help healthy people and sub-healthy people to establish an orderly and healthy life style, reduce risk state and keep away from disease by maintaining and promoting health, through the arrangement of medical services, as soon as possible to restore health. In this paper, the mechanism of food spoilage was studied. The resistance and moisture content of food were detected by using water detection circuit and resistance detection network, and compared with the standard value of fresh food, to judge the extent of spoilage of food spoilage, to make food edible or not. Spoilage and deterioration of food can cause poisoning or potential harm to the human body. People with mild poisoning often have symptoms of acute gastroenteritis, such as vomiting, diarrhea or abdominal pain, fever, etc., severe poisoning in the respiratory, nervous, circulatory and other system symptoms, a slight delay may appear life-threatening. Therefore, food spoilage and deterioration has a great impact on human health, detection of food spoilage and deterioration of human health management is of great significance.

### Acknowledgments

This work was supported by Ministry of Education industry-university Cooperation Project (project number: 220604307070644), and this paper is supported by the college-level first-class specialty Communication Engineering Construction Project of in Shaanxi University of Technology.

## LHMM23140 PSYCHOLOGICAL ANALYSIS AND ADJUSTMENT OF STUDENTS’ ANXIETY IN COLLEGE ORAL ENGLISH TEACHING

Sha Li^a^, Xinmeng Ke^b^

*^a^ Department of Foreign Language, Zhengzhou University of Science and Technology, Zhengzhou, Henan 450064, China, ^b^ Office of Academic Affairs, Zhengzhou Railway Vocational and Technical College, Zhengzhou, Henan 450064, China.*

**Objective:** In recent years, in college oral English teaching, it has been found that more and more students are feeling anxious and afraid of opening their mouth to speak English, which hinders the improvement of their oral communicative ability. This is not in line with the policy of cultivating application-oriented talents. Therefore, this paper aims to analyze the reasons that lead to students’ anxiety in oral English class and then proposes strategies that could help to adjust students’ anxiety. It is hoped that teachers could get some suggestions to ease students’ anxiety and help them maintain a healthy mentality in oral English learning, which is conducive to cultivate students’ communicative ability to meet the standards of application-oriented talents cultivation.

**Method:** This paper mainly adopts qualitative analysis based on the analysis of previous literature. In order to effectively solve the problem of students’ anxiety in oral English learning, this paper first makes a brief summary of anxiety from psychological perspective, and then analyzes in depth the important impact of anxiety on college students’ oral English learning. Then, the main subjective and objective factors influencing the degree of students’ anxiety are analyzed. Based on the problems found in previous section, effective strategies to ease students’ anxiety are put forward.

**Results:** It is pointed out in this paper that anxiety has an negative influence on students’ oral English learning. Then the subjective and objective factors contributing to anxiety are analyzed. The subjective factors are ideal level, learning motivation and learning ability while the objective factors are teachers’ teaching ability and region differences. At last, strategies to help students reduce anxiety in oral English learning are proposed. The main strategies are building a good classroom atmosphere for oral English teaching, organizing group activities appropriately, timely guiding students to correct oral mistakes, scientifically designing classroom questioning, enriching language and culture input.

**Conclusions:** Based on the analysis of the negative impact of anxiety on oral English learning and its major influencing factors from both the subjective and objective perspective, the strategies proposed in this paper are aimed to help students adjust their anxiety, overcome the psychological stress during the learning process so as to fundamentally promote students’ oral English learning efficiency and effect, exercise and develop their oral expression and English application skills, which can ensure the effect and quality of oral English teaching to truly meet the needs for oral English professionals for the development of the society in the context of globalization.

## LHMM23141 EFFECT ANALYSIS OF PROVINCIAL COMPREHENSIVE HEALTHCARE REFORM POLICY IN CHINA - BASED ON SYNTHETIC CONTROL METHOD

Ting Zhang^a^, Weirong Wang^a^, Wen Qiao^a^, Xinyi Li^a^

*^a^ Department of Public Policy and Management, Northwestern Polytechnical University, Xi’an, Shaanxi 710072, China.*

**Background:** The provincial comprehensive medical reform is an important pilot policy to improve the efficiency of China’s basic medical and health services. It is of great theoretical and practical significance to systematically explore the impact of policy implementation on optimizing resource allocation and improving the service efficiency of primary medical and health institutions.

**Subjects and Methods:** Based on this, we first adopted the super-efficient DEA model to evaluate the medical service efficiency in primary healthcare institutions in China from 2010 to 2019, then analyzed the effect and the influencing factors of policy implementation in the first pilot areas through the synthetic control method.

**Results:** 1. the service efficiency of primary healthcare institutions in China ranges from 1.061 to 1.01, the average values of the efficiency in the eastern, central and western regions were 1.259, 0.835 and 0.962 in that order. The eastern region shows an inverted “N” shape, the central region shows a “U”-shaped cycle, and the western region has shown a fluctuating downward trend. 2. In the pilot area, the gap between Jiangsu and synthetic Jiangsu is widening after the implementation of the policy, it shows that the provincial healthcare reform pilot policy effect in Jiangsu is obvious. The probability of the ineffectiveness of that policy is less than 10%. The placebo test was not effective in the pilot areas other than Jiangsu.

**Conclusions:** 1. The service efficiency of grass-roots medical and health institutions in China presents an Eastern > Western > Central trend. The service efficiency shows a decline in different degrees, with a large gap between provinces. 2. Only Jiangsu province has an apparent positive policy effect among the first batch of pilot regions. After the policy implementation, the changing trends of the Anhui, Fujian and Qinghai provinces cannot reflect the effect of the policy. To improve the efficiency of medical and health institutions in China, this paper puts forward some suggestions in terms of strengthening the structural investment of medical resources in the central and western regions and promoting the implementation of a graded diagnosis and treatment system.

### Acknowledgments

This work was supported by a project grant from General Project of National Social Science Fundation (Grant No.20BJY076).

## LHMM23142 INFLUENCE OF NOISE ON WORKERS’ SAFETY BEHAVIOR AND PHYSICAL HEALTH IN UNDERGROUND CONFINED SPACE

Mingwei Xu^a,b^, Yufei Qiu^a^, Sitong Ji^a^, Jianwu Chen^b^, Lu Yu^c^, Shengnan Ou^d^, Xiaoya Xie^d^

*^a^ Department of Safety Engineering, School of Safety Engineering,Beijing Institute of Petrochemical Technology, Beijing 102617, China, ^b^ China Academy of Safety Science and Technology Beijing 100083, China, ^c^ Department of Safety Engineering, School of Traffic and Transportation Engineering, Dalian Jiaotong University Dalian 116021, China, ^d^ School of Civil and Resource Engineering, University of Science and Technology Beijing 100083, China.*

**Background:** In modern workplaces, noise pollution has been identified as a significant risk factor that poses a threat to the safety productivity and workers’ occupational health. However, the impact of sound level and frequency on workers’ attention and behavior in confined spaces is not fully understood. Therefore, this study aimed to investigate the effects of sound level and frequency on attention concentration ability, reaction time, and discrimination time in a confined space environment.

**Subjects and Methods:** A human experiment was conducted with 13 participants. The experiment design included three sound levels (0 dB, 60 dB, and 70 dB) and three frequency levels (100 Hz, 1000 Hz, and 10,000 Hz). Participants were exposed to different sound levels and frequencies in a confined space to investigate the impact of sound level and frequency on their attention concentration ability, reaction time, and discrimination time.

**Results:** The study results showed that sound level and frequency had different mechanisms of influence on attention. Sound level had a more significant impact on workers’ attention concentrating ability, indicating that the greater the sound level, the less attention was paid to safety. On the other hand, frequency primarily affected attention concentration level, with intermediate frequency noise significantly reducing the attention concentration level. When the sound level was 70 dB or the frequency was 1000 Hz, the simple reaction time and discrimination time increased significantly, and the concentration ability was the weakest. When the sound level was 60 dB or the frequency was 100 Hz, the attention level of the subjects was the lowest.

**Conclusions:** This study’s findings demonstrate that noise pollution in confined spaces can have detrimental effects on workers’ attention concentration, reaction time, and discrimination time. Sound level and frequency were found to have different impacts on attention, with sound level being more significant in reducing attention concentration ability. These findings highlight the need to control noise pollution in confined spaces and to consider both sound level and frequency when developing noise control strategies to enhance occupational safety and efficiency. Therefore, appropriate noise control measures must be implemented to minimize the risk of noise exposure and protect workers’ health and well-being.

### Acknowledgements

This work was supported by a project grant from Beijing Municipal Commission of Education (Grant No. KM202310017002) and National Natural Science Foundation of China (Grant No. 5230041812).

## LHMM23143 PRE/ POST COVID NONVERBAL COMMUNICATION IN EDUCATION IN AND OUT OF CHINA: FROM THE PERSPECTIVE OF EMOTION REGULATION

Qingzhao Li^a^, Farzaneh Haratyan^a^, Fei Jiang^b^

*^a^ School of Foreign Languages, Suzhou University, Suzhou, Anhui, 234000, China, ^b^ School of Information Engineering, Suzhou University, Suzhou, Anhui, 234000, China.*

**Background:** Emotional regulation refers to a psychological process in which individuals change the intensity, quality, and duration of emotions through self-regulation in an environment that stimulates emotions. Emotional regulation is closely related to nonverbal communication, as nonverbal communication is often the main way to express emotions. In nonverbal communication, individuals can convey their emotional state by adjusting their own facial expressions, gestures, and sounds, while also interpreting others’ nonverbal signals to obtain information and infer others’ emotional state. Both verbal and nonverbal communications are involved in the teaching and learning activities. Verbal behavior is the main channel through which teachers impart knowledge to students, while nonverbal behavior is the main channel through which teachers convey their emotions, simplify teaching instructions, activate the student’s learning interest and enhance teaching effectiveness.

**Subjects and Methods:** This paper reviewed the findings of twenty recently related research inside and outside Chinese contexts and discussed how they converged or diverged in their achieved results about this concept of nonverbal communication in pre covid and post covid era. This paper also included the way it has been defined, practiced, and evaluated considering various variables that interact and affect nonverbal communication.

**Results:** The changes in emotional regulation and nonverbal communication pre covid and post covid are mainly reflected in the attitudes of positive expression, negative emotions and moderate expression. There are close links between the nonverbal signals and the emotional expressions, and the intention of the sender of the information can be revealed frequently from non-verbal cues. Both students and teachers can benefit from practicing nonverbal communication skills.

**Conclusions:** The changes in emotional regulation in nonverbal communication before and after the epidemic reflect people’s psychological state and coping strategies in the epidemic environment. In nonverbal communication, people can communicate and obtain information by adjusting their emotions, and they can also infer the emotional state of others by interpreting their nonverbal signals. This paper maintains how students’ attention, initiative and participation beside other psychological mechanisms in education can be influenced and how to focus on emotional regulation and nonverbal communication strategies to promote both students and teachers’ emotional health. Effective non-verbal communication skills have a positive effect on both teaching and learning. This paper is beneficial to teacher trainers to enhance the effectiveness of college English teaching.

### Acknowledgements

This work was supported by a project grant from Anhui Provincial Department of Education, (Grant No. 2020jyxm2196) and a project grant from Anhui University Humanities and Social Sciences Research Project (Grant No. SK2021A0714).

## LHMM23144 APPLICATIONS OF INTELLIGENT ENVIRONMENTAL MONITORING IN MODERN MEDICAL TREATMENT

Zhecong Wang^a^, Xiao Tan^b^, Siyang Yu^b^, Xilong Qu^b^

*^a^ Software Academy, Changsha Commerce & Tourism College, Changsha, Hunan 410116, China, ^b^ School of Information Technology & Management, Hunan University of Finance and Economics, Changsha, Hunan 410205, China.*

**Background:** Environmental monitoring plays a crucial role in medical facilities, impacting patient outcomes and staff safety. Manual monitoring methods are time-consuming and prone to errors. However, with advancements in sensor technology, ZigBee communication protocols, and mobile devices, intelligent environmental monitoring systems offer efficient and accurate real-time monitoring. In hospitals, monitoring temperature, humidity, air quality, and other environmental factors is essential for maintaining a sterile and safe environment for both patients and healthcare providers. Traditional manual detection and recording methods are labor-intensive and error prone. Intelligent environmental monitoring systems, on the other hand, offer several benefits that can enhance the quality of care in medical facilities.

**Subjects and Methods:** Wireless communication between sensing devices, using ZigBee technology, and a central monitoring system allows easy deployment and scalability. Sensing devices placed throughout the hospital monitor critical areas such as operating rooms, patient rooms, and laboratories. The data collected from these sensors transmits quickly to the central monitoring system, alerting healthcare providers if there are any deviations from the desired environmental conditions. The integration of mobile devices such as smartphones or tablets enables remote access to the monitoring system, facilitating real-time monitoring of different areas of the hospital. Healthcare providers can monitor environmental conditions, even when they are not physically present in the hospital, ensuring timely intervention if required.

**Results:** Moreover, intelligent environmental monitoring systems have expanded beyond temperature and humidity control. They can also monitor gas levels, radiation, and dust particles to ensure a safe environment for patients and healthcare providers. Additionally, they can monitor power consumption, contributing to energy-efficient management in medical facilities. Considering the complex environment of hospital storage, we attach the monitoring system to a mobile tool (such as a mobile car), which can make the detection method of the entire system more flexible, with a wider detection radius, and enable the system to be used for temperature and humidity monitoring in these diverse environments.

**Conclusions:** When ZigBee technology, sensing technology, and mobile devices are combined to form a temperature and humidity monitoring system, it has the advantages of low cost, rapid installation, and high availability compared to common solutions in the industry. As technology continues to advance, further research and development in environmental monitoring systems will lead to more effective applications in modern medical treatment.

### Acknowledgments

This research was supported by Scientific Research Key Project of Hunan Education Department[21A0592], Education Reform Project of Hunan Province[HNJG-2021-1378], Hunan Natural Science Foundation Project (2023)[Research on Resource Scheduling Strategy for Energy Efficiency Optimization in Mobile Cloud Computing].

## LHMM23145 STUDY ON THE ALLEVIATION OF FARMERS’ PSYCHOLOGICAL ANXIETY IN THE STRATEGY EVOLUTION GAME OF SICHUAN-SHAANXI OLD REVOLUTIONARY AREAS UNDER THE BACKGROUND OF RURAL REVITALIZATION

Qiang Zhou^a^

*^a^ School of Finance and Economics Management, Sichuan University of Arts and Sciences, Dazhou, 635002, China.*

**Background:** In the implementation process of the rural revitalization strategy in the Sichuan Shaanxi Revolutionary Old Area, due to the relative uncertainty of policy responses from multiple parties, this article takes the alleviation of psychological anxiety in the process of changing the livelihood of farmers in the revolutionary old area during the government governance process as one of the research subjects. In analyzing the effectiveness of the rural revitalization strategy with the participation of social capital, the strategic evolution game between the government, social capital, and farmers is taken as the research goal, Defining farmers in old areas as units that examine the effectiveness of social capital and government policies is in line with the implementation units and fields of the current rural revitalization strategy. Reflect the stability, continuity, and balance of interests of all parties in the long-term dynamic strategy process.

**Subjects and Methods:** Evolutionary game theory was used to study the dynamic process of governance, select a mechanism with dynamic incentive factors, and combine the social capital participation in the process of rural revitalization with the measures taken by villagers to alleviate life anxiety and psychological counseling in the process of rural revitalization, reflecting the relative stability and long-term nature of the government decision-making process and the process of people’s livelihood.

**Result:** As the government actively promotes rural revitalization and the development of red tourism resources in revolutionary old areas, and improves the effectiveness of incentive subsidies, the probability of social capital and farmers choosing cooperation strategies in the short term increases. However, due to the long-term existence of subsidies, incentive subsidy policies can only stimulate cooperation between the two in a short period of time. In addition, with the increase in subsidy amounts, the convergence speed actively promoted by the government due to incentive subsidy expenditures has also slowed down. Similarly, numerical simulations can simulate the impact of punishment costs on the level of psychological anxiety during changes in social capital, farmer behavior, and livelihood status. Overall, in regulating non cooperative behavior between social capital and farmers, the increase in punishment costs is more effective than incentives, and all three are more sensitive to incentive returns. Thus, in the process of promoting rural revitalization policies, the anxiety state of farmers’ livelihood changes caused by policy promotion has been effectively improved.

**Conclusion:** The stability of the evolutionary game model established at the governance level of rural revitalization strategy will directly have a significant impact on the implementation of rural revitalization policies. And it plays a certain helping role in improving the livelihood status of regional farmers and alleviating psychological anxiety. Therefore, this article has certain theoretical innovation significance and practical value. The countermeasures derived from the tripartite evolutionary game model have certain guiding significance for policy and theoretical decision-making departments.

## LHMM23146 STUDY ON THE EFFECT OF ENZYMATIC HYDROLYSIS PEPTIDE OF ANTLER PLATE OF SIKA DEER-RED DEER HYBRID ON IMMUNE FUNCTION OF MICE

Mei Yang^a^, Zitong Sun^a^, Xiwen Xu^a^, Ling Su^b^, Yuting Li^a^, Xiuran Wang^a^, Wei Hu^a^

*^a^ College of Life Sciences, Jilin Agricultural University, Changchun, Jilin 130118, China, ^b^ Engineering Research Center of Edible and Medicinal Fungi Ministry of Education, Jilin Agricultural University, Changchun, Jilin 130118, China*

**Objective:** enzymatic hydrolysis peptide of antler plate is an ossified substance formed in the spring of the second year after deer antler is cut off or sawed. Modern pharmacological research shows that anlter plate has many pharmacological activities. Protein and peptides account for about 40% of the total components of antler plate. They are rich in amino acids, including essential amino acids. According to the content and types of amino acids, it is speculated that the bioactive protein or polypeptide contained in the enzymatic hydrolysis peptide of antler plate of sika deer-red deer hybrid deer has immune activity. The active peptide is a protein peptide segment with biological function after protein is hydrolyzed by protease. The peptide segment not only has certain biological activity, but also retains its original biological activity.

Patients and Methods: In this study, the enzymatic hydrolysis peptide of antler plate of sika deer-red deer hybrid was used as raw material. The enzymatic hydrolysis peptide of enzymatic hydrolysis peptide of antler plate was obtained by enzymatic hydrolysis technology, and the immune activity of the enzymatic hydrolysis peptide was evaluated by its influence on the immune function of mice. Firstly, the total protein was extracted from the enzymatic hydrolysis peptide of antler plate of sika deer-red deer hybrid deer by alkali extraction and acid precipitation, and the effects of several common proteases (alkaline protease, neutral protease, trypsin and papain) on the total proteolytic efficiency and immune activity of enzymatic hydrolysis peptide of antler plate were investigated, so as to obtain the enzymatic hydrolysis peptides with the best immune activity. The gastric administration doses of enzymatic hydrolysis peptides of mice in each group were 100 mg/kg in low dose, 200 mg/kg in medium dose and 400 mg/kg in high dose respectively for 14 days. Then cyclophosphamide was used to induce mice to establish immunosuppressed mouse model. Finally, the changes of body weight, immune organ index, proliferation ability of mouse spleen lymphocytes and phagocytic activity of peritoneal macrophages were detected, and the changes of cytokine and immunoglobulin expression levels in serum were detected by ELISA sandwich method.

**Results:** The Results showed that the proliferation ability of RAW264.7 macrophages treated with trypsin was the best, so trypsin was selected to hydrolyze the total protein of anlter plate. The enzymatic hydrolysis conditions were as follows: temperature 40℃, time 3h, E/S 3%, pH 7 and enzyme dosage 3000 U/g. The immune activity experiment showed that compared with the model group, the experimental group could significantly increase the weight of mice, improve the index of immune organs, enhance the proliferation of spleen lymphocytes and the phagocytosis of peritoneal macrophages, and also improve the expression levels of serum cytokines (IL-2, IFN-γ) and immunoglobulins (IgM, IgG, IgA).

**Conclusions:** enzymatic hydrolysis peptide of antler plate has obvious immunoregulation effect on immunosuppressed mice, and effectively improves the suppressed humoral immune function of mice.

### Acknowledgments

This study was supported by Jilin Science and Technology Development Plan Project (Nos.20200404024YY), and Special Fund Project for Provincial Industrial Innovation in Jilin Province (Nos. 2018C048-4).

## LHMM23147 A RESEARCH ON THE NECESSITY OF AESTHETIC EDUCATION TO ALLEVIATE COLLEGE STUDENTS’ PSYCHOLOGICAL HEALTH PROBLEMS AND CULTIVATE AESTHETIC VALUES

Suitai Sun^a^, Guoxia Hu^b^, Shuling He^c^

*^a^ College of Art of Guangxi Minzu Normal University, Chongzuo, Guangxi 532200, China, ^b^ College of Marxism of Guangxi Minzu Normal University, Chongzuo, Guangxi 532200, China, ^c^ Institute of Applied Technology of Honghe University, Honghe, Yunan 661199, China.*

**Purpose:** College students’ mental health, aesthetic taste and aesthetic ability have been deeply concerned by all social strata in recent years. Therefore, the ultimate purpose of this study is to explore the necessity of carrying out aesthetic education in universities to alleviate college students’ mental health problems and cultivate aesthetic values.

**Methods:** By means of questionnaire survey and interview, the necessity of aesthetic education in alleviating the mental health problems and cultivating aesthetic values of college students of different majors, grades, genders and sources were investigated and analyzed.

**Results:** The mental health index of male students and female students before and after aesthetic education from freshman to senior year were significantly different. The mental health indexes after aesthetic education were 97%, 94%, 90% and 86%, respectively, and the decline trend was slow. Science, engineering and sports students mostly considered beauty to be natural beauty, while music and art students, on the other hand, considered beauty to be exotic dresses or a fashion trend. The proportion of freshmen who considered beauty is natural beauty was the highest 85%, the proportion of junior college students who think beauty is fashion trend and exotic clothes is the same as that of sophomore college students, accounting for 56%. The proportion of male students and female students in the fourth grade of university who thought beauty as natural beauty was 32%, 31% respectively. The proportion of freshmen who considered beauty is natural beauty is the highest at 42%, the proportion of urban sophomores who prefer fashion trends is the highest at 44%, the proportion of urban juniors who think beauty is fashion trends is the highest at 48%, and the proportion of urban seniors who think beauty is physical beauty is the highest at 32%. Among parents of college students from different walks of life, 70.84% of the parents of farmers and 50.58% of the parents of workers think that college education is the main front and effective way to cultivate college students’ aesthetic education. Teachers of different subjects in colleges and universities think that the average values of aesthetic education in colleges and universities are respectively 55.4%, 26.0%, 12.3% and 6.4%. Students of different grades participate in different activities in different numbers, types and ways. According to the average number of college students taking part in different practice channels, the number of students taking part in summer three trips to the countryside accounted for 18% at most, and the number of students visiting red bases accounted for 13.5% at least.

**Conclusion:** It is necessary to carry out aesthetic education in colleges and universities to alleviate college students’ psychological health and cultivate aesthetic values.

### Acknowledgements

This is the Class B Project of Undergraduate Teaching Reform Project of Guangxi Higher Education in 2021 “The Construction and Teaching Research of Red Resources in Left and right river area Region into the Courses of Art Major in Local Colleges and Universities” (Project No.: 2021JGB378); 2022 School-level Curriculum Ideological and Political Teaching Team of Guangxi Normal University for Nationalities “Inheriting Classics: Ideological and Political Teaching Team of Chinese Painting Course” (Project No.: KCSZJXTD202204); 2022 School-level Curriculum Ideological and Political Demonstration Course of Guangxi Normal University for Nationalities “Creation of Chinese Landscape Painting” (Project No.: KCSZSFKC202207) Phased results. The completion of this thesis is inseparable from the valuable suggestions and suggestions put forward by your colleagues. Thank you again for giving me a lot of encouragement and help. Recall the whole writing process of the thesis, although it is not easy, but let me get rid of impetuous, a lot of gains.

## LHMM23148 RESEARCH ON THE INTEGRATION BETWEEN LIBERAL ARTS EDUCATION AND IDEOLOGICAL AND POLITICAL THEORY COURSES IN PRIVATE UNIVERSITIES FROM THE PERSPECTIVE OF HEALTHY PSYCHOLOGY AND CULTURAL STUDIES

Aihui Jin^a^, Lili Tang^a^

*^a^ Zhuhai College of Science and Technology, Zhuhai 519041, China.*

**Background:** Chinese culture, since ancient times, has been instrumental in nurturing the refinement, cognitive skills, and ethical virtues necessary for building and maintaining a healthy society, thus serving as the “identity card” and spiritual glue of the nation. With the advancement and progress of society, propelled by modern science and technology, and the ongoing process of economic globalization, new cultural elements have emerged. These include network culture, information culture, competition culture, and leisure culture, which are increasingly diverse and prominent. In the current cultural milieu, university students not only engage in knowledge acquisition but are also exposed to a diverse range of cultures that affect their values, which may give rise to various psychological and cognitive issues. The psychological and value-oriented issues among university students caused by cultural problems have prompted widespread attention.

**Subjects and Methods:** Private universities, as an essential constituent of universities, share a responsibility for imparting the core socialist values, promoting the excellent traditions of Chinese culture, guiding students’ healthy psychological development and assume significant influence on education.

**Results:** Exploring the convergence between ideological and political education and Liberal Arts Education in the context of contemporary healthy psychological culture can establish students’ accurate worldview, life philosophy, and values, which can help in enhancing their overall psychological well-being.

**Conclusions:** The significance of cultural psychology in achieving cultural inheritance, resisting the invasion of decadent culture, and establishing student beliefs is instrumental, especially in the context of cultural integration. Therefore, in the education and teaching of private universities, schools must prioritize a healthy psychological and cultural perspective and pay attention to the role of cultural psychology in the integration of Liberal Arts Education and ideological and political theory courses. This will enable schools to exploit their specific educational advantages, which will foster better development and progress of students in private universities.

### Acknowledgments

Supported by the 2020 research project of the “Thirteenth Five-Year Plan” for Guangdong Province’s Education Science (Ethical Education Project)/ Office of the Leading Group for Guangdong Education Science Planning: “In-depth Research on the Integrated Development of Liberal Arts Education and Ideological and Political Education in Private Universities from the Perspective of Cultural Power”. (Project No.: 2020JKDY056).

## LHMM23149 AN ANALYSIS OF THE IMPACT OF BTM COSTS ON CONSUMER SURPLUS IN THE P2P RENTAL MARKET FROM THE PERSPECTIVE OF CONSUMER PSYCHOLOGICAL ANXIETY

Fuhong Guo^a^

*^a^ City Institute, Dalian University of Technology, Dalian 116600, China.*

**Background:** Consumer psychological anxiety is a negative emotion that occurs when there is a BTM cost in the P2P leasing market, resulting in a decrease in total consumer surplus. When perceived threats or risks of the elasticity suppliers and renters exceed their personal control, it leads to negative emotions such as unease, tension, and distress, resulting in psychological anxiety for both elasticity suppliers and renters. This is manifested as the concern for the new elasticity suppliers whether they should continue to rent out their unused products and the concern of renters whether they should rent products in the P2P leasing market. Although theoretically elastic suppliers can rent out their unused assets for free in the P2P rental market, in practice, there is at least some cost associated with providing a rentable product. The rise of the “sharing economy” has been driven by a significant reduction in these costs, and the P2P rental market has brought an increase in consumer surplus and social welfare. However, in the long run, the cost of entering the market, or the BTM cost, has reduced the total consumer surplus in the P2P rental market. This paper analyzes the mechanism through which BTM costs affect consumer surplus in the P2P rental market and finds that they reduce the total consumer surplus, leading to a decrease in profits for elastic suppliers and renters, and causing them psychological anxiety about whether to exit the P2P rental market. Based on this, the paper proposes recommendations for reducing consumer anxiety and promoting the operation and development of the P2P rental market.

**Subjects and Methods:** Firstly, this paper reviews the literature on P2P rental market, BTM costs and consumer psychological anxiety. Secondly, this paper compares short-term consumer surplus between the existence and absence of BTM costs in P2P rental market and draws conclusions. Thirdly, when BTM costs exist, this paper compares long-term and short-term consumer surplus in P2P rental market and draws relevant conclusions. The last part is the conclusion and inspiration of this paper.

**Results:** This paper concludes through the comparative analysis of short-term and long-term consumer surplus in P2P rental market with and without BTM costs: BTM costs reduce total consumer surplus in P2P rental market and cause psychological anxiety for elastic suppliers and renters. Short-term consumer surplus is higher than long-term consumer surplus, and even when long-term consumer surplus reaches the lowest point, psychological anxiety for elastic suppliers and renters reaches its highest point, which leads to their unwillingness to participate in P2P rental market.

**Conclusions:** Based on the analysis results, P2P rental platforms should reduce BTM costs, as much as possible in the long term. This can increase the income of elastic suppliers and renters, thereby reducing the psychological anxiety of both parties and lower psychological anxiety for elastic suppliers and attract more elastic suppliers who can offer more idle products in P2P rental market. There are two main ways to reduce BTM costs: one is for P2P rental platforms to find renters and achieve the best match between elastic suppliers and renters, while monitoring the entire process of product rental use, protecting the rights and interests of elastic suppliers, and reducing their psychological anxiety. The other is to achieve economies of scale and scope in P2P rental market, which can reduce unit cost for elastic suppliers and rental price for renters, and lower their psychological anxiety, thus increasing the willingness of elastic suppliers and renters to participate in P2P rental market.

### Acknowledgments

This work was supported by a project grant from the Funding Project for Teaching and Research of Liaoning Provincial Department of Education. (Grant No. LJKR0679) and Project funded by the Liaoning Provincial Social Science Fund (Grant No. L18BJY014).

## LHMM23150 NETWORK-BASED PHARMACOLOGICAL STUDY ON THE MECHANISM OF SCUTELLARIA BARBATA POLYSACCHARIDE IN THE TREATMENT OF COLON CANCER

Sitong Zhou^a^, Jingyun Yu^b^, Zhaoru Zhou^b^, Haotian Wang^b^, Shuo Han^a^, Hao Sun^a^, Zhipeng Zhao^a^, Pengda Sun^a^

*^a^ Department of Gastrointestinal Nutrition and Hernia Surgery, The Second Hospital of Jilin University, Changchun, Jilin, China, ^b^ The School of Pharmaceutical Sciences, Jilin University, Changchun, Jilin, China.*

SZ and JY contributed equally to this work.

**Background:** Increasing incidence of colon cancer, the role of Scutellaria barbata polysaccharides (SBP) in tumors has drawn widespread attention. In order to further investigate its mechanism of action in the treatment of colon cancer, this paper try to use network pharmacology approaches to predict its possible mechanism of action.

**Methods:** The literature was reviewed to understand the active components of SBP; the Swisstarget database was used to obtain the potential targets of the active components of SBP; the GeneCards and DisGeNET databases were used to screen the disease-related targets of colon cancer, set the relevant parameters and summarize the disease targets, and finally the intersection of the potential targets of SBP and the disease targets was taken to plot the Venn. The STRING database was used to construct the target protein interaction (PPI) network to obtain the core target proteins; gene ontology (GO) analysis and Kyoto Encyclopedia of Genomes (KEGG) pathway enrichment analysis were performed on the potential genes, and the key signaling pathways of SBP in colon cancer were obtained by using the DAVID6. 8 online tool.

**Results:** The results showed 12 monosaccharide components were obtained from some magazines and journals, such as Rhamnose, Fucose, Arabinose, Xylose and Mannose etal. The Swisstarget database records 23 potential targets of Scutellaria barbata polysaccharides (SBP), and there are 1391 colon cancer disease targets, which were summarized from two databases, GeneCards and DisGeNET. There were 8 intersecting targets of Scutellaria barbata polysaccharides and colon cancer, namely VEGFA, HSP90AA1, LGALS3, FGF2, CDK1, HPSE, LGALS4, and FOLH1. Enrichment analysis showed that MAPK signaling pathway, PI3K-Akt signaling pathway, and HIF-1 signaling pathway were the main affected pathways. We predict that the process of colon carcinogenesis may be regulated by acting on intersectional targets as well as signaling pathways to alleviate disease symptoms.

**Conclusions:** Scutellaria barbata polysaccharides is a kind of polysaccharide formed by the compound of many monosaccharides. It can exert the mechanism of action for the treatment of colon cancer through multiple components with multiple targets and pathways.

### Acknowlegdments

This work was supported by project grants from Natural Science Foundation of Jilin Province (Grant No.20200201014JC) and Finance Department Health Special Project of Jilin Province (Grant No. 3D5204921429).

## LHMM23151 STUDY ON THE CONTENT AND EFFECTIVENESS OF EPIDEMIC PREVENTION SLOGANS FROM THE PERSPECTIVE OF EMOTION MANAGEMENT

Xutao Zheng^a^, Zhiyao Li^a^

*^a^ China University of Mining and Technology (Beijing), Beijing 100083, China.*

**Background:** To investigate the dissemination effects and audience attitudes towards various types of epidemic prevention slogans, with the aim of better safeguarding the positive emotions of the public during public health emergencies.

**Subjects and Methods:** In this study, the linguistic styles of epidemic prevention slogans were divided into serious, friendly, rude and other categories, and 831 epidemic prevention slogans were quantitatively analyzed. At the same time, 55 people were interviewed to study the influence of different linguistic styles of slogans on the attitudes of the audience. In particular, this paper focuses on the impact of rude slogans on the mood of the audience.

**Results:** The study found that there were significant differences in linguistic styles among epidemic prevention slogans released in urban and rural areas, as well as those released by different regions or publishers. Even in the early stages of the pandemic, rude slogans were not the majority among epidemic prevention slogans. The center region has a larger percentage of crude slogans as compared to other areas. Villages have a larger percentage of crude slogans than towns do. The township (subdistrict office), public organizations, and village (neighborhood) committees are more likely to print rude and friendly slogans as compared to other levels of public management organizations. The severity of the pandemic, the publisher’s understanding of the target audience, the publisher’s role in epidemic prevention, and legal awareness were all factors that affected the content of the slogans. Different audience attitudes were observed towards various linguistic styles of the slogans. Slogans that can bring positive feelings to the audience are more likely to be observed by the audience. Serious and friendly slogans were more likely to receive positive evaluations and compliance. Although rude slogans could elicit some degree of compliance, they were not easily accepted by the audience and may even prompt negative emotions such as resistance, dissatisfaction, anger, fear, and other adverse responses. The reasons why rude style slogans are followed by some people may be related to the potential punishment of the publisher, the authority of public management organizations and the warning effect of such slogans.

**Conclusion:** In terms of the dissemination effects of epidemic prevention slogans, public managers and researchers should not only focus on whether the public is complying with the epidemic prevention policies and following the requirements of the slogans, but also pay attention to the impact of epidemic prevention slogans on public emotions and psychology. During public health emergencies, public managers must pay close attention to emotional management, respect the emotions of audience, protect citizen privacy, and use more friendly slogans rather than crude ones.

## LHMM23152 RESEARCH ON THE INFLUENCING OF RURAL TEACHER LOSS AND PSYCHOLOGICAL ANXIETY: AN EMPIRICAL STUDY BASED ON PROVINCE H

Xia Tao^a^, Wenjing Duan^a^

*^a^ Wuhan Institute of Technology, School of Marxism, Wuhan 430200, China.*

**Background:** The youth of today are the backbone of social development and the leaders of the progress of the times. In the context of the new era, the key to achieving comprehensive rural revitalization lies in education, and the key to education development lies in teachers, especially young teachers. Through research and studies, it is found that the phenomenon of rural young teachers’ attrition is obvious, and teachers’ mental health problems are prominent, especially young teachers in rural areas with lower economic development levels have relatively high psychological anxiety index. Therefore, it is urgent to provide high-quality education services in rural areas, strive to build an excellent teaching force, further narrow the gap between urban and rural education, alleviate teachers’ psychological anxiety, and stabilize the development of rural basic education.

**Subjects and Methods:** For rural schools in province H with different levels of economic development, 16 rural young teachers who joined in 2015 and had leaving experiences were selected through a targeted sampling method, and the texts of the interviewees were coded and analyzed using the rooted theory research method, and after open coding, spindle coding, and selective coding, a theoretical model was constructed for the influencing factors of rural young teachers’ attrition and their psychological anxiety.

**Results:** Through the analysis of the rooted theory research method, the four highest dimensional categories were finally summarized and concluded that the factors affecting rural young teachers’ attrition and their psychological anxiety are mainly school factors, parental factors, social factors, and personal factors, and specific contents were collated in each factor, containing a total of 14 aspects.

**Conclusions:** Based on the current situation of rural young teachers’ attrition and their psychological anxiety, this study endeavors to improve teachers’ psychological quality, enhance their sense of belonging and happiness at work, and devote themselves to education with full motivation from the perspective of paying attention to teachers’ psychological health. Based on this, reducing rural young teachers’ attrition rate and alleviating teachers’ psychological anxiety are mainly based on improving school infrastructure, focusing on joint home-school education, accelerating rural economic development, and meeting career development needs, which have practical implications for the development of rural basic education.

### Acknowledgments

This research was supported by the open fund project of Hubei Teacher Education Research Center, a key research base of humanities and social sciences in Hubei universities in 2022, entitled “Research on the Influencing of Rural Teacher Loss and Psychological Anxiety: an Empirical Study based on Province H” (Project No. jsjy202203).

## LHMM23153 THE PSYCHOLOGICAL ADJUSTMENT EFFECT OF SHORT VIDEO IN URBAN-RURAL INTEGRATION BASED ON SCENE THEORY

Linwen Xiao^a^, Naikao Wang^b^

*^a^ School of Economics and Management, Nanning Normal University, Nanning, Guangxi 530001, China, ^b^ School of Journalism and Communication, Nanning Normal University, Nanning, Guangxi 530001, China*

**Background:** In traditional China, the gap between urban and rural areas is large, so is the psychological distance between urban and rural areas. There is a two-way stereotype between urban people and rural people. In the process of rapid urban-rural integration, they need to break the psychological barrier quickly. If this problem is not solved well, the social group will appear psychological sub-health state.

**Subjects and Methods:** In recent years, China’s urban-rural integration has accelerated. However, neither urban nor rural people showed any of these collective mental health problems. In order to find out how to adjust urban and rural psychological contradictions in the process of accelerated integration, the research group selected Gangwei Town, Longhai District, Zhangzhou City, Fujian Province, China, as the research object, and conducted research using the grounded theory research method.

**Results:** The results show that: (1) In the process of urban-rural integration, the melting speed of urban-rural cultural and psychological boundary is greater than that of urban-rural material space boundary. (2) Short video plays an important role in psychological adjustment during the process of psychological boundary ablation. (3) In the creation, dissemination and consumption of short videos, urban people and rural people participate in the process of cultural production, forming a psychological adjustment mechanism of cultural communism and cultural sharing. (4) Under this psychological adjustment mechanism, the cultural boundary between urban and rural people is eliminated, and the psychological gap is crossed, and no mental health problems caused by the rapid integration of urban and rural areas appear.

**Conclusions:** Short videos satisfy the psychological needs of mutual lookout between urban and rural areas, and content creators actively promote the cultural communization and sharing mechanism between urban and rural areas, avoiding the group mental health problems caused by the rapid integration of urban and rural areas. The Chinese government should pay full attention to the psychological adjustment role of short video and other new media in the process of urban-rural integration, guide people’s new understanding, new attitude and new behavior of the new urban-rural relationship through new media, and then actively promote the rural revitalization and urban-rural development. This study is a case study, and this mechanism may be more complicated, which needs further discussion.

### Acknowledgements

This work was supported by a project grant from The 14th Five-Year Plan Education Project of Fujian Province, China “Research on the Training Mechanism of College Students’ Short Video Content Entrepreneurial Talents”(Grant No.FJKBK21-135).

## LHMM23154 THE INFLUENCE OF THE DEVELOPMENT OF DIETETIC CULTURE HERITAGE ON THE MENTAL HEALTH OF RESIDENTS IN THE JIANGSU SECTION OF GRAND CANAL

Hao Wu^a,d^, Yan Duan^a^, Rubing Xia^a,d^, Jinwei Lv^c^, Peng Zhang^a^, Zhizhong Shen^a,d^, Yong Lu^a^, Mingku Ju^e^, Haoran Teng^a^, Yang Huang^b^

*^a^ College of Humanities and Social Development, Nanjing Agricultural University, Jiangsu, Nanjing 210095, China, ^b^ School of Cultural Heritage and Information Management, Shanghai University, Shanghai 200444, China, ^c^ Institute of History, Jiangsu Academy of Social Sciences, Jiangsu, Nanjing 210004, China, ^d^ College of Agricultural Civilization, Grand Canal Cultural Belt Construction Research Institute, Jiangsu, Nanjing 210004 China, ^e^ School of History and Culture, Henan Normal University, Henan, Xinxiang, 453007, China.*

**Background:** The dietetic culture heritage of the Jiangsu section of the Grand Canal has a very important impact on people’s physical and mental health. Diet is based on agriculture. In terms of traditional Chinese social form, China has always been an agricultural society. Agriculture is the main production sector of society, providing food services to the people, and the vast majority of social material wealth is provided by agriculture. Based on this natural ecological environment, the regional dietetic culture of the people in the Jiangsu section of the Grand Canal has been integrated into the cultural mechanisms of different cities in Jiangsu, with unique cultural symbolic significance and emotional memory. The resulting customs and habits show the local people’s understanding of the nature of food. Along the Grand Canal, people’s preferences for food taste, food material types and even cooking skills express the residents’ preferences for food mental health, and the resulting ideological, emotional and economic factors, which are the living inheritors of the sustainable development of dietetic culture’s dietetic culture heritage.

**Subjects and Methods:** This paper takes the operation, service and management concepts of the inheritors of dietetic culture heritage as the research entry point, systematically combs the dietetic culture related heritage in the Intangible Cultural Heritage List of Jiangsu Province, combs the number of national level projects, interviews the inheritors of dietetic culture heritage, investigates their attitude towards the future development of dietetic culture heritage, and analyzes their psychological and external reasons for this attitude. At the same time, it combed the dietetic culture inheritance bases related to Grand Canal in Jiangsu Province, discussed the relationship between base construction and inheritance protection, and the resulting impact on the mental health of residents along the Grand Canal.

**Results:** Through the above investigation and analysis, the development of dietetic culture heritage of Grand Canal in Jiangsu has a positive impact on the mental health of surrounding residents. We will find that a specific diet structure will produce a specific diet psychological structure, while the dietetic culture heritage of Grand Canal in Jiangsu has a long history, healthy diet, strong cultural genes, relatively perfect diet hygiene conditions and other characteristics. It is no longer just a simple satisfaction of appetite, but increasingly displays aesthetic psychological characteristics. At the same time, the dietetic culture heritage of Grand Canal in Jiangsu still has four problems: lack of strategic thinking and specific development goals, lack of talent training mechanism, imperfect support system, and unclear inheritance and protection standards. Although the existence of these problems does not have a great impact on the mental health of residents, in the long run, these problems will lead to negative effects on the dietetic culture heritage of Grand Canal in Jiangsu. In order to prevent these negative problems, this paper puts forward some suggestions, such as formulating comprehensive strategic goals, strengthening the psychological health intervention of inheritors, improving food hygiene policy support, highlighting the development of key dietetic culture heritage, strengthening academic support of universities, so that the dietetic culture heritage of Grand Canal in Jiangsu can develop healthily and orderly, and continue to adhere to the principle of the unity of truth and innovation in the process of inheritance, And incorporate the concepts of food tradition, food hygiene, and food innovation.

**Conclusions:** The formation of dietetic culture heritage in the Jiangsu section of the Grand Canal is closely related to the mental health of residents along the Grand Canal. What to eat is not only a phenomenon, but also a psychological behavior of people, which is inseparable from the psychological factors behind it. Therefore, we adhere to the principle that the dietetic culture heritage of Grand Canal in Jiangsu should be global, national, scientific and historic. Only in this way can the dietetic culture heritage of Grand Canal in Jiangsu maintain its vitality in the new historical period, thus having a positive impact on the residents along the Grand Canal in its development process.

### Acknowledgments

This work was supported by a project grant from Grand Canal Cultural Belt Construction Research Institute (Jiangsu) think tank special research project “Research on Agricultural civilization resources and its Exploitation and utilization in Grand Canal” (DYH19ZXA1). Nanjing Agricultural University Central University operating funds project “Investigation and research on the Diet Cultural Heritage in the Grand Canal coastal area of Jiangsu Province” (SKYC2020008). Key project of Sichuan Cuisine Development Research Center, Philosophy and Social Science Key Research Base of Sichuan Education Department ”Study on Conservation of Jiangsu Traditional Diet Cultural Heritage Resources”(CC18W01)

## LHMM23155 THE IMPACT OF PSYCHOLOGICAL ANXIETY UNDER ENVIRONMENTAL REGULATION ON FDI AND THE TECHNOLOGICAL COMPLEXITY OF CHINA’S EXPORTS

Jianhua Zhu^a^, Ziyang Zhou^a^

*^a^ School of Economics and Finance, Zhanjiang University of Science and Technology, Zhanjiang, Guangdong 524094, China.*

**Background:** to summarize the effects of psychological anxiety under the intensity of environmental regulation on foreign direct investment FDI and the technological complexity of Chinese exports, and to analyze the regularity of such effects, in order to improve the technical complexity of China’s export products, we can alleviate the psychological anxiety caused by environmental regulation, improve the psychological expectation of FDI, optimize the psychological account and investment portfolio.

**Subjects and Methods:** Based on Chinese provincial panel data from 2010 to 2019, the GMM model was used to examine the relationship between export technology complexity, foreign direct investment anxiety and environmental regulation intensity, the degree of psychological anxiety was quantified by the size of the foreign direct investment, and the panel threshold model was used to examine the relationship between the export technological complexity and the foreign direct investment under different environmental regulatory intensities, and the foreign direct investment characteristics of export technological complexity and the variation of psychological anxiety under different environmental regulatory intensities.

**Results:** (1)environmental regulation had a significant positive effect on the psychological anxiety of FDI. Different environmental control intensity will lead to “Pollution halo” and “Pollution shelter”, but it will not change the psychological goal and investment direction of FDI. (2) there is a U-shaped relationship between environmental regulation and the increasing complexity of export technology. The increased complexity of exporting technology depends on the sum of the “Cost-offsetting” and “Innovation-offsetting” effects, as well as the degree of psychological anxiety of the foreign direct investment. (3)the psychological anxiety of the foreign direct investment has a significant effect on the increase of export technological complexity. With the increase of environmental regulation, the complexity of FDI and export technology shows the “N”-type double threshold characteristics affected by investment psychological anxiety. (4) while foreign direct investment are affected by the psychological anxieties arising from the strengthening of environmental regulations, and the complexity of exporting technologies is indirectly affected through the effective interaction of environmental regulations, the intensity of environmental regulations must be moderate, can only play a catalytic role in a certain range.

**Conclusions:** under the background of strengthening environmental supervision, the psychological anxiety of FDI will increase, and it will affect the technological complexity of export products, moreover, there is a significant positive effect between the intensity of environmental regulation, the degree of psychological anxiety of FDI and the technological complexity of export products, it is necessary to stabilize investment expectations for FDI, to control foreign direct investment anxiety and to increase the technological complexity of Chinese exports.

### Acknowledgments

The authors acknowledge support from Major Program of National Social Science Foundation of China [19XMZ089].

## LHMM23156 MENTAL HEALTH: THE KEY TO UNLOCKING INNOVATION POTENTIAL—MEASUREMENT OF URBAN INNOVATION EFFICIENCY IN THE YANGTZE RIVER DELTA URBAN AGGLOMERATION FROM THE PERSPECTIVE OF URBAN HAPPINESS INDEX

Donghua Zhang^a,b^, Yi Wang^c^, Youyang You^d^, Yufei He^d^, Yangyu Ou^d^, Qiuyue Zou^d^

*^a^ School of Economics, Yunnan University of Finance and Economics, Kunming 650221, China, ^b^ School of Economics and Management, Dali University, Dali, 671003, China, ^c^ School of Logistics and Management Engineering, Yunnan University of Finance and Economics, Kunming 650221, China, ^d^ International Business School, Yunnan University of Finance and Economics, Kunming 650221, China.*

**Background:** As an important part of our life, happiness has become an important indicator to measure a person’s quality of life, which helps to enhance personal and physical health, increase social stability, and promote personal and social development. People with high levels of well-being tend to be more able to experience positive emotions, such as optimism, self-confidence, satisfaction, happiness, etc., and are also more likely to cope with negative emotions and stress in life. Mental health can improve the quality of life and happiness of individuals, promote physical health, and also contribute to social stability and development. Among the many factors that affect residents’ sense of well-being and happiness index, the impact of urban innovation level cannot be ignored. As a key path to improve the city’s happiness index and residents’ sense of well-being, technological progress has become a hot spot in academic research.

**Subjects and Methods:** This paper takes the Yangtze River Delta urban agglomeration as the research object, and explores the innovation efficiency of the Yangtze River Delta urban agglomeration and its influencing factors by constructing a three-stage DEA model and a K-means cluster analysis model. The advantages of using the three-stage DEA model to measure the innovation efficiency of the Yangtze River Delta urban agglomeration lie in the following aspects: First, the weights of input variables and output variables are generated by mathematical models through data, and do not need to be set in advance, which reduces the error of subjective weight setting; The second and third stages of DEA eliminate the influence of environmental variables and random noise in the second stage, making the results more accurate. K-means clustering further expands the research of this paper.

**Results:** The research found that: (1) After excluding environmental factors and random factors, the innovation efficiency of cities in the Yangtze River Delta urban agglomeration has been significantly improved; (2) Urban innovation efficiency is affected by the level of economic development, the degree of advanced industrial structure, government support, and the intensity of innovative human capital. (3) The innovation efficiency level of the Yangtze River Delta urban agglomeration can be roughly divided into innovation lagging type, innovation development type and innovation leading type.

**Conclusions:** This study has important guiding significance for improving the innovation efficiency of urban agglomerations and enhancing the mental health and happiness of residents.

## LHMM23157 STUDY ON THE INFLUENCE OF FINANCIAL MODEL INNOVATION OF RURAL REVITALIZATION ON FARMERS’ MENTAL HEALTH IN ZHEJIANG PROVINCE

Yijie Tang^a^, Songyan Zhang^a^

*^a^ School of Economics and Management, Zhejiang University of Science and Technology, Hangzhou, Zhejiang 310000, China.*

**Background:** Healthy mental state needs to be based on good material conditions. The economic level and material conditions in rural areas are lower than those in urban areas, which may lead to adverse development of rural household mental health. As one of the important factors to promote regional economic development, financial model can affect the mental health of farmers by changing material conditions. Therefore, the innovation and development level of financial model deserve high attention. At present, there are some financial problems such as brain drain, low financing demand, few mortgage loans, poor infrastructure, imperfect credit investigation system and diversified demand in the process of rural revitalization in Zhejiang Province. In rural areas with low economic level, there are some mental health problems such as spiritual poverty, lack of confidence, lack of motivation for poverty alleviation and incorrect concept of development. This paper aims to study the operation mechanism of innovative financial model in rural areas of Zhejiang Province and its impact on farmers’ mental health, and put forward relevant suggestions for promoting rural revitalization, sustainable development of agricultural health and cultivation of farmers’ mental health in Zhejiang Province.

**Subjects and Methods:** The innovative financial model in rural areas of Zhejiang Province is selected as the research object. First, look up and read a large number of case materials, and have an in-depth understanding of the rural revitalization strategy, the concept of financial model, the situation of rural revitalization in Zhejiang Province and the mental health status of rural households. Second, the paper investigates the innovative financial models generated in the process of rural revitalization in Zhejiang Province and makes a preliminary analysis. Third, select influential and representative innovation models such as “insurance + futures” model, supply chain finance model, “clean energy + green building” model as the main research object. This paper analyzes how different financial models support rural revitalization and affect the mental health status of farmers, and obtains the operating mechanism, key content and main impact from actual cases.

**Results:** The three innovative financial models have a good effect on the development of financial level in rural areas, effectively solve the financial problems and farmers’ mental health problems in rural areas of Zhejiang Province, and significantly improve the economic level, material conditions and farmers’ mental health degree in rural areas.

**Conclusions:** “Insurance + futures” mode can affect the psychology of farmers and promote the development of their mental health by improving their material conditions. Supply chain finance model plays a significant role in solving the psychological poverty problem of farmers. The model of “clean energy + green building” can promote farmers to shape the concept of sustainable development and form healthy psychology.

### Acknowledgments

This research was supported by a project grant from National College Student Innovation and Entrepreneurship Training Program.

## LHMM23158 ANALYSIS ON THE CURRENT SITUATION AND INFLUENTIAL FACTORS OF CHINESE COLLEGE STUDENTS’ PSYCHOLOGICAL SENSE OF GAIN IN THE COURSE OF IDEOLOGICAL AND THEORETICAL EDUCATION

Wenjun Zhang^a,b^, Qing Zhang^c^

*^a^ School of Marxism, Chongqing Technology and Business University, Chongqing, China, ^b^ People’s Government of Oitak Town, Aketao County, Xinjiang, China, ^c^ Primary School of Xinli Town, Zhongxian County,Chongqing, China.*

**Background:** This paper aims to investigate the current situation and influencing factors of Chinese college students’ psychological Sense of Gain in the process of Ideological and Theoretical Courses, so as to provide reference for the implementation of curriculum teaching reform for China and other countries.

**Subjects and Methods:** This paper uses the “survey method” to conduct the relevant research, and uses SPSS software to sort out the questionnaire data, and adopted descriptive statistics and correlation analysis to carry out empirical analysis of the status quo and influencing factors of the Sense of Gain of more than 800 Chinese college students.

**Results:** From the investigation, the psychological Sense of Gain of Chinese college students from Ideological and Theoretical Courses is between “average” and “relatively strong”, which is not strong enough. From the viewpoint of the factors influencing students’ learning psychology in the Ideological and Theoretical Courses, the “Sense of Gain” is mainly influenced by the learning psychological stress and learning participation, and the teaching objectives, contents and methods, teachers’ emotional care and affinity also has a relatively influence. Moreover, the students’ learning environment also has some influence on learning psychology in Ideological and Theoretical Courses.

**Conclusions:** In view of the current situation of college students’ psychological Sense of Gain in Ideological and Theoretical Courses and the existence of these influencing factors. First of all, Ideological and Theoretical Courses teachers and other course teachers need to increase the teaching affinity between teachers and students in the Ideological and Theoretical Courses, close the psychological distance between teaching and students, ease students’ negative emotion. Second, teachers need consider the learning psychology needs of college students when formulating the objectives, content and methods, reduce students’ psychological stress of learning. Third, teachers need give college students enough emotional care in the course to create a heart warming study environment, so that to inspire college students’ produce positive learning motivation, so as to stabilize their will to learn. Those methods could promote the “emotional resonance” and “psychological integration” between teachers and students, making college students feel the positive emotional and psychological impact in course. Moreover, it isn’t only helps college student continuously increase the knowledge in the learning process,but also achieves the purpose of further enhancing college students’ psychological Sense of Gain in Ideological and Theoretical Courses.

## LHMM23159 RESEARCH ON THE DANCE TEACHING EFFECTIVENESS EMPOWERED BY MENTAL HEALTH IN GUANGDONG UNIVERSITIES FROM THE PERSPECTIVE OF THE 5C KEY COMPETENCIES

Chang Yu^a,b^, Haijian Hu^c^

*^a^ School of Music, Guangdong Polytechnic Normal University, Guangzhou 510665, Guangdong, China, ^b^ School of Education, City University of Macau, Macau 999078, China, ^c^ School of Marxism, Guangdong Polytechnic of Science and Technology, Zhuhai 510640, Guangdong, China.*

**Background:** Dance education in China has experienced new development since the turn of the century, and the evaluation of the effectiveness of dance teaching in colleges and universities has expanded after the “Overall Plan for Deepening Educational Evaluation Reform in the New Era” was issued by the State Council. During the process of teaching dance, it is essential to effectively understand students’ psychological response to dance and the positive psychological effects of learning dance. It is important to cultivate self-awareness, self-control, self-efficacy, and adaptive thinking in students, which will help them handle challenges and stress in learning, achieve higher quality learning outcomes, and increase satisfaction with their dance education. Such psychological qualities are also integral to the development of a student’s dance performance, and can greatly enhance the effectiveness of dance teaching.

**Subjects and Methods:** Based on the theory of the 21st Century 5C Key Competencies Model and the maturity scale of domestic classroom teaching effectiveness, starting in2020 this study adapted and verified the 5C key competencies and dance teaching effectiveness scale specifically for college dance majors. Descriptive, correlation, and regression analyses were used to analyze 634 valid questionnaires from Guangdong universities and explore the correlation between 5C key competencies and the effectiveness of dance teaching in universities. Thus, this study explores the path from the 5C key competencies to dance psychology to ensure the teaching effectiveness of “Heart-Dance in Harmony.”

**Results:** The results of data analysis showed a significant correlation between the 5C key competencies and the effectiveness of dance teaching in colleges; the 5C key competencies had predictive power for the effectiveness of college dance teaching.

**Conclusions:** Dance education has a positive impact on reducing students’ stress and negative emotions from multiple aspects. In dance teaching, students can cultivate their understanding and appreciation of culture, which helps them feel a sense of self-identity and belonging in dance while enhancing their cultural confidence. Improving critical thinking skills can reduce loneliness and self-doubt, enabling students to face challenges and stress more confidently. Developing innovative skills can enhance students’ dance thinking ability and creative ability, increase self-efficacy and belief. In collective teaching and training, effective communication and cooperation can build stronger and healthier interpersonal relationships, fostering students’ cooperation spirit, trust, sharing, and sense of responsibility. the 5C key competencies are bestowed upon educational and psychological research, enabling students to deal with stress, alleviate pressure, and promote their mental and physical health more effectively. This study aimed to explore the effectiveness of university dance education in light of the 5C key competencies. It adhered to the principles of combining scientificity and practicality in education, focusing on positive psychological effects. In this modern age, university educators must encourage the physical and mental development of their students in line with the 5C standards while also possessing the 5C key competencies themselves. Through focused efforts, they can enhance the reach, depth, and orientation of dance instruction within university classrooms. A key aspect of this endeavor is the exploration of individual potential and strengths. This involves honing the creativity, communication, cooperation, critical thinking, and cultural awareness of students to aid in their attainment of success and personal fulfillment.

### Acknowledgments

This work was supported by a project grant from 2022 Annual Project of the “14th Five-Year Plan” for the Development of Philosophy and Social Sciences in Guangzhou; “Research on developing Guangzhou as a model city for educational reform and development in the Guangdong-Hong Kong-Macao Greater Bay Area: from the perspective of aesthetic education empowering a humanistic Bay Area development” (Project grant No.2022GZGJ289).

## LHMM23160 RESEARCH ON THE DEVELOPMENT PATH OF FRUIT TOURISM INTEGRATION IN GLOBAL TOURISM DESTINATIONS BASED ON CONSUMER BEHAVIOR AND PSYCHOLOGICAL ANXIETY

Aizhong Wang^a^, Qiong Wang^a^

*^a^ College of Tourism, Chongqing University of Arts and Sciences, Chongqing 402160, China.*

**Background:** Under the background of all-for-one tourism, the integration of fruit tourism is becoming an important driving force for the development of rural tourism. This paper studies the consumer psychological needs and consumption behavior of rural tourism consumers in Wulong District, Chongqing municipality, analyzes the problems existing in the integrated development model of fruit tourism in Wulong District and the integrated development of fruit tourism in the whole district, and puts forward targeted countermeasures to provide a basis for policy formulation and long-term planning of the government and rural tourism enterprises.

**Subjects and Methods:** Based on the theory of consumer behavior, this paper takes consumer behavior and consumer psychology research as the breakthrough, adopts the methods of stratified sampling and random sampling, divides the tourist population into existing consumers and potential consumers, selects several typical rural tourism scenic spots, important fruit production areas, leisure picking intensive areas as the research area, and investigates the demographic characteristics of tourists, the characteristics of tourists’ consumption behavior, the psychological characteristics of tourists, and the development status, mode and problems of fruit tourism. A total of 1,000 questionnaires were distributed, and 988 were recovered, with a recovery rate of 98.8%, of which 926 were valid. After the survey, the data were statistically analyzed using SPSS20.0 software.

**Results:** Relaxation, leisure and entertainment, psychological anxiety, melancholy getting rid of, emotional regulation, nostalgia, perception and show-off, personality traits, etc. have become the main motivations for people to choose fruit tourism. Among them, relaxation, leisure and entertainment are the traditional influencing factors of rural tourism development, and the importance of nostalgia, personality traits and psychological anxiety in the integration of fruit tourism is becoming more and more prominent. The survey results of tourist satisfaction show that there is a serious phenomenon of psychological homogenization and inferiority among tourists caused by the lack of fruit tourism products and projects is serious, and the lag of public service facilities, the lack of human resources, and the lack of integration mechanism aggravate tourists’ sense of disappointment and psychological identity. According to the psychological characteristics and behavioral habits of tourism consumers, the integrated development model of fruit tourism in Wulong District is divided into government intervention type, collective loose type and peasant household freedom type.

**Conclusions:** Under the background of all-for-one tourism, the consumer factors of the integrated development of fruit tourism in Wulong District should not only consider the consumption needs of traditional tourists such as relaxation and leisure, but also pay attention to psychological motivations such as psychological anxiety, depression and desolation, emotional regulation, nostalgia, personality traits and perception of show-off. Relevant parties can regulate and control by studying the actual needs of tourism consumers, tapping regional advantages and characteristics, optimizing public infrastructure construction, and building an innovative mechanism for the linkage development of fruit tourism.

### Acknowledgments

This work was supported by the Humanities and Social Sciences Project of the Ministry of Education“ Research on the Global Development Mode and Realization Mechanism of Non-Optimal Tourism District in Wuling Mountain Area under the Strategy of Rural Revitalization” (19YJC630161)

## LHMM23161 A COMPARATIVE STUDY OF DETECTION RESULTS OF TRANSFUSION-TRANSMISSIBLE INFECTIONS MARKERS APPLYING DIFFERENT TESTING STRATEGIES

Xingxiang Xing^a^, Lu Zhang^a^, Qichao Mao^a^, Miaoyu Wang^a^

*^a^ Shaoxing Central Blood Station, Shaoxing City 312000, China.*

**Background:** To understand and evaluate the detection efficacy of different testing strategies for transfusion-transmissible infections markers in blood donations, to choose appropriate testing strategies, and to effectively prevent transfusion-transmitted infections.

**Subjects and Methods:** The detection results of transfusion-transmissible infections markers in 214,474 blood donation samples collected between 2019 and 2022 were retrospectively analyzed and compared using three testing strategies (designated as A, B, and C) based on relevant specifications.

**Results:** The total nonconformity rate of blood tests under testing strategy A was 1.25%, and the nonconformity rates of ALT, HBsAg, Anti-HCV, Anti-HIV, Anti-TP, HBV-DNA, HCV-RNA, and HIV-RNA were 0.42%, 0.17%, 0.15%, 0.08%, 0.18%, 0.19%, 0.01%, and 0.01%, respectively, indicating a relatively low level of nonconformity. Among 17 confirmed HIV-infected donors, NAT detected 1 case first. The consistency rates of positive results for HBV, HCV, and HIV infection-related markers were 16.53%, 27.09%, and 20.43% under testing strategies A, B, and C, respectively. The total nonconformity rates of HBV, HCV, and HIV infection-related markers were 0.52%, 0.40%, and 0.39%, respectively, with statistically significant differences in nonconformity rates between HBV and HIV infection-related markers. Compared with testing strategy A, the relative detection rates of HBV, HCV, and HIV infection-related markers were 77.69% and 76.06% under testing strategies B and C, respectively. Testing strategy B detected 247 fewer cases compared to strategy A, with 246 fewer cases of HBV infection, no difference in HCV infection, and 1 fewer case of HIV infection. On the other hand, testing strategy C detected 265 fewer cases compared to strategy A, with 113 fewer cases of HBV infection, 46 fewer cases of HCV infection, and 106 fewer cases of HIV infection.

**Conclusions:** Testing strategies with more comprehensive detection items ensure a higher level of blood safety. Multiple testing methods with different types of markers are necessary in practice. Testing strategies with fewer detection items need to be cautiously applied. NAT is essential for blood screening, and the combination of multiple testing methods can improve detection rates.

### Acknowledgements

We would like to express our gratitude to Mr. Sang Liyong, Director of Shaoxing Central Blood Station, and Mr. Fu Liqiang, Deputy Director, for their valuable guidance, help, and support during this study.

## LHMM23162 MULTI-LEVEL ANALYSIS OF LABOR COST UNDER THE BACKGROUND OF PSYCHOLOGICAL ANXIETY

Zhiying Liu^a^

*^a^ Jiang Su Urban and Construction Vocational College, Changzhou city, Jiangsu Province 213000, China.*

**Background:** According to the research report of China Academy of Fiscal Science on the labor cost of China labor market, in the first half of 2018-2021, the average monthly wage of sample enterprises increased from 4,182 yuan to 5,367 yuan, with an average increase of 9.44%, especially in the first half of 2021, the average monthly wage increased by 12.63%. Enterprises are facing the pressure of rising costs, and job seekers are facing a state of job anxiety, how to solve the adverse effects and provide a positive signal for job seekers is the focus of this paper. Under the difficulty of rising labor costs, enterprises have psychological anxiety; job seekers face the increasing problems of rising living and housing costs. If the psychological anxiety problem is not paid attention to, and cannot be alleviated, it will develop into anxiety disorders, which may lead to changes in people’s body, and cause social problems.

The accurate distribution and analysis of labor cost realize reasonable control and provides a feasible path for enterprises.

**Subjects and Methods:** This paper studies the multi-level analysis of labor cost difference in standard cost method; In the paper the comparative analysis and case analysis are used. In the article a manufacturing company is chosen as a typical case.

**Results:** The labor cost problem needs the joint efforts of internal and external parties, such as external policy support, but it is also very important for enterprises to adopt appropriate cost methods. At present, there are two systems for the treatment of labor costs, which are the actual cost allocation method based on working hours and the standard cost method based on standards. Both methods have their shortcomings.

**Conclusion:** The key to the traditional standard based accounting and distribution method lies in the determination of standard working hours and wage rate standards. Besides the strong subjectivity in the formulation of standards, the reference factors are single and the labor level and the nature of jobs are not considered, which is also a major problem. The multi-level analysis of standard cost method proposed in this paper ensures the stability and systematic of cost indicators. This method can explain the causes of differences in detail and provide data support for later cost control and provide positive signal and relief the mental pressure relative to labor.

## LHMM23163 THE EFFECTS OF LOGO DESIGN CHARACTERISTICS’ CHANGES ON CONSUMERS’ RESPONSES: FROM THE PERSPECTIVE OF CONSUMERS’ EMOTION REGULATION

Ying Zhao^a^, Qinhai Ma^a^

*^a^ School of Business Administration, Northeastern University, Shenyang 110819, China.*

**Background:** This research aims to investigate the impact of brand logo change on consumers’ emotion regulation by exploring the effects of logo design characteristic changes. Enterprises often modify their logos to maintain their brand image, which requires continuous planning and adaptation to consumers’ emotion regulation. Therefore, brands aim to keep up with the times and maintain a positive image by redesigning their logos in ways that satisfy consumers’ emotional needs. Such needs include familiarity for loyal customers, meaning similarity with the old logo for the target audience, and the ability to evoke positive affect and good surprise. Consumers’ emotional needs are critical for brand logo changes to be effective, as they influence how consumers respond to and engage with logos.

**Subjects and Methods:** Our research involves conducting empirical experiments to investigate the relationship between logo design characteristics and consumer responses. Firstly, we identified eight distinct logo design characteristics. Secondly, we identified four different types of consumer responses to logo changes. Thirdly, we identified four underlying design dimensions of logo changes, which are natural, harmonious, elaborate, and integral, and four response dimensions. Finally, we conducted an empirical analysis of 100 logos to identify those that meet three different strategic objectives.

**Results:** Our empirical research has led us to propose design guidelines for logo changes that take into account consumers’ emotion regulation. Our analysis revealed that for minor logo changes in a mature market, the change should be medium in terms of naturalness and elaboration, low in terms of harmony, and high in terms of integrity. Conversely, major logo changes in a mature market should involve a high level of natural change transitioning to medium, a positive and substantial change in harmony, a low to medium level of elaboration, and a negative and low change in integrity. For logo changes in a young market, the guidelines suggest a low to medium level of natural change, a positive and substantial change in harmony, a low to medium level of elaboration, and a negative and low change in integrity.

**Conclusions:** Our findings indicate that any change in brand logos can have a significant impact on consumers, highlighting the importance of catering to their emotion regulation. The design guidelines for logo changes provide marketing managers with a reliable theoretical basis for minimizing or avoiding potentially negative effects, such as reduced market competitiveness, increased costs, or the risk of losing brand assets. Our research contributes to the design guidelines of brand logo change in several ways. First, it offers a new perspective on the relationship between a series of design characteristics of brand logos and their impact on consumers’ response, which is closely linked to their emotion regulation. Second, it proposes design guidelines for logo changes with respect to three different strategic objectives. Third, we provide purely illustrative recommendations for different types of logo changes, taking into account the impact on consumers’ emotion regulation.

## LHMM23164 THE EFFECT OF RISK PERCEPTION ON DEPRESSION AMONG COLLEGE STUDENTS DURING THE COVID-19 PLATEAU: THE PROTECTIVE EFFECT OF PSYCHOLOGICAL CAPITAL

Yifan Wang^a^, Hongmei Li^b^, Lizhang Dong^b^

*^a^ School of Education, Baoshan University, Yunnan 678000, China, ^b^ School of Psychology and Cognitive Science, East China Normal University, Shanghai 200062, China.*

**Background:** Since the start of the epidemic, people across the country have experienced a “lockdown period” in early 2020, when all places are closed down and travel restricted, and a “plateau period” when the epidemic is relatively stable after the epidemic is brought under control and the lockdown is lifted. The plateau period is a period of continuous response, reflection and adjustment to the epidemic situation. It is of great value and significance to explore the mental health status of college students during the plateau period, which can provide a new direction of thinking for the subsequent mental health education of college students. Depression is a condition to be concerned about; Psychological capital is an effective mediating variable of the psychological stress response of college students under the epidemic. For the mental health education of colleges and universities, it is helpful to reduce the impact of the major epidemic on the mental health of college students, promote the self-growth of college students, and promote the scientific psychological education model of colleges and universities. The impact of major epidemic on the psychological capital of Chinese college students has both advantages and disadvantages Positive environment and support resources such as effective prevention and control of the government, concerted efforts of the people and family companionship are conducive to the growth of optimism and hope factors of psychological capital. At the same time, the major epidemic has a negative impact on the self-efficacy and psychological resilience of college students with little stress experience, weak personality foundation, high psychological pressure and little social support. In order to explore the influence of college students’ risk perception level on their depressive state and the protective role of psychological capital in the stable period of the epidemic, this study investigated freshmen and seniors of Baoshan University in Yunnan Province.

**Subjects and Methods:** Totally 5138 college students were surveyed using the COVID-19 Risk Perception Scale (PRCPS), Depression Angst Stress Scale (DASS-21) and Compound PsyCap Scale (CPC-12)

**Results:** (1) Risk perception positively predicts depression; (2) Hope, efficacy and optimism in psychological capital can negatively predict depression; (3) The efficacy dimension of psychological capital moderates the relationship between risk perception and depressive state, that is, the predictive power of risk perception on depressive state decreases with the increase of efficacy.

**Conclusion:** High risk perception tends to lead to depression in college students, while psychological capital can reduce the occurrence of depression, and the efficacy dimension of psychological capital plays a protective role in the relationship between risk perception and depression.

## LHMM23165 THE POSITIVE EFFECT OF THE BEAUTY OF THREE GENERATIONS OF THE QING DYNASTY DECORATION ON RELIEVING VIEWERS’ PSYCHOLOGICAL ANXIETY IN THE PERSPECTIVE OF PSYCHOLOGICAL NEEDS

Dan Long^a^, Aihong Wang^a^

*^a^ School of Design and Art, Jingdezhen Ceramic University, Jingdezhen 333000, China.*

**Background:** “It is one of the important varieties of glaze decoration of traditional Chinese ceramics, and is an artistic expression with ceramics as the carrier. The development of pastel has a certain transitional period. This paper studies the beauty of ceramic pastel decoration in the three Qing dynasties (Kangxi, Yongzheng, and Qianlong) from the perspective of “psychological needs” and explores the role of its “unique” painting language in psychological healing. The purpose of this study is to examine the psychological development of the human psyche and the psychological role of aesthetics.

**Subjects and Methods:** Using Maslow’s Theory of Needs as a theoretical guide and a combination of qualitative and quantitative and comparative research methods, the study aims to analyze the role and healing of the “aesthetic expression” of the painting language of pastel decoration in the Qing and Third Dynasties on mental health.

**Results:** The analysis focuses on the meaning of the lines, composition and craftsmanship in relation to psychological needs and psychological health, and the interrelationship between the “artistic characteristics” and “psychological needs” of Qing Dynasty pastels. The analysis shows that the Qing Dynasty pastel decorations were effective in relieving people’s stress and anxiety at the level of “practical needs”, in healing people’s aesthetic psychology at the level of “aesthetic needs”, and in relieving people’s negative emotions at the level of “value needs”.

**Conclusions:** From this, it is concluded that the pastel decoration of the Qing Dynasty has a unique aesthetic of art, both psychological regulation and compensatory dynamic function, but also can make the artistic literacy and artistic inculcation of people to facilitate the maintenance of psychological health, art from life and above life, so that art into real life, so that people maintain a positive and healthy psychological needs of the development of the situation is particularly necessary.

### Acknowledgements

This research was supported by the [(Jinshu Class [2022] General YB2022144)] project.

## LHMM23166 RESEARCH ON PERSUASIVE TECHNOLOGY AND DESIGN FOR HEALTHY LIFESTYLE THROUGH SYSTEMATIC LITERATURE REVIEW (SLR)

Kang Ji^a^, Xiaoshu Li^b^, Ruhaizin Sulaiman^a^, Khairul Manami Kamarudin^a^, Rosalam Che Me^a^

*^a^ Faculty of Design and Architecture, Universiti Putra Malaysia, Serdang, 43400, Malaysia, ^b^ School of art & design, Zhengzhou University of Aeronautics, Zhengzhou, 450046, China.*

**Objective:** Maintaining a healthy lifestyle plays a vital role in personal health and social development. However, in reality, many people have various problems maintaining healthy behaviors and are unable to maintain a healthy lifestyle. Most of the existing healthy lifestyle-related products on the market are designed based on the principle of persuasion, and intervene in behavior through various persuasive strategies, so as to achieve the purpose of changing behavior. In order to provide designers and researchers with more effective design solutions in product design in the future, this study systematically analyzes and gives an objective assessment of the persuasive principles and strategies used in product persuasive design.

**Methods:** Through the method of systematic literature review, search the persuasion technology and application of persuasion strategies in healthy life and the Fogg behavior model (FBM) from the two search engines of Scopus and Connected Paper, then strictly follow the PRISMA flow chart to provide relevant screening and evaluation according to the requirements of eligibility, inclusion or exclusion of articles, 23 eligible articles were finally selected for systematic analysis.

**Results:** This study found that the Fogg Behavior Model and persuasive strategy used in the existing products can effectively promote the formation of healthy behavior and improve people’s healthy lifestyle. In addition, the effectiveness of persuasive technology will also be affected by a variety of other factors, such as the influencing factors of target behavior, social environment, intervention stage of persuasive strategy, intervention duration, etc. In order to improve the effectiveness of products, these factors should be considered by researchers and designers.

**Conclusions:** The systematic literature review provides an important theoretical basis for designers and researchers in the process of product design related to healthy lifestyle through the study of persuasive principles, persuasive strategies and Fogg Behavior Model. This study emphasizes the importance of persuasive technology and design in the process of healthy lifestyle intervention and summarizes the advantages of persuasive design from four aspects: orientation, friendliness, interactivity and ethics.

## LHMM23167 SAFETY AND EFFICACY OF 980 NM DIODE LASER ENUCLEATION FOR TREATMENT OF NON-MUSCLE-INVASIVE BLADDER CANCER

Xiaolei Ren^a^, Haiju Liu^b^, Chunsheng Li^a^

*^a^ Urology Department, Affiliated Hospital of Chifeng University, Institute of Urology, Chifeng University 024000 Chifeng, China, ^b^ Cancer Screening Department, Chifeng Cancer Hospital, 024000 Chifeng, China.*

XR and HL contributed equally to this study.

**Background:** To evaluate the safety and efficacy of 980 nm diode laser enucleation for treatment of non-Muscle-invasive bladder cancer.

**Subjects and Methods:** The data of the patients with non–muscle-invasive bladder cancer treated by either 980 nm diode laser enucleation (n=56) or conventional transurethral resection of bladder tumor (n=52) were analyzed retrospectively. The preoperative characteristics and intraoperative complications were compared in the 2 groups.

**Results:** No significant differences in the sex, age, mean tumor number, mean tumor max. size, tumor multiplicity, tumor location, tumor stage, and recurrence risk of the 2 groups were observed. All patients accepted the successful 980 nm diode laser enucleation or TURBT, respectively. No obturator nerve reflex and bladder perforation occurred during surgery in the 980 nm diode laser group. However, 11 patients in the conventional transurethral resection of bladder tumor group encountered obturator nerve reflex, and 2 of them encountered bladder perforation. There were no significant differences between the 2 groups in transfusion rate. Patients in the 980 nm diode laser group had shorter the mean duration time of the operation, duration postoperative continuous bladder irrigation time, and catheterization time than that in the conventional transurethral resection of bladder tumor group. There was no significant difference in the overall 1-year recurrence rate between the 2 groups during the follow-up periods. Also, no statistical difference was found when the comparison was made among every risk subgroups between the two groups.

**Conclusions:** 980 nm diode laser enucleation for treatment of non–muscle-invasive bladder cancer demonstrates an advantage over conventional transurethral resection of bladder tumor in reducing intraoperative complications, shortening the mean duration time of the operation, shorting the time of duration of postoperative continuous bladder irrigation, and shorting the time of catheterization.

### Acknowledgments

This research was supported by Inner Mongolia Autonomous Region Higher Education Science Research Project: NJZY21140.

## LHMM23168 RESEARCH ON THE COMMUNICATION MODEL OF CHINESE CALLIGRAPHY EDUCATION FOR INTERNATIONAL CHINESE EDUCATION: FROM THE PERSPECTIVE OF MENTAL HEALTH

Guirong He^a^,Tong Zhao^b^

*^a^ Department of China International Language and Culture College, Krirk University, Bangkok10220, Thailand, ^b^ Department of Culture Industry, College of Humanities and Social Sciences, Kwangwoon University, Seoul, 01897, Republic of Korea.*

**Background:** With the acceleration of the process of globalization, more and more people have a strong interest in learning Chinese language and culture. In international Chinese education, Chinese calligraphy, as a unique cultural and artistic form, has profound historical origin and cultural connotation, and has become an effective way and means to attract foreigners to learn Chinese. However, in the actual teaching process, due to the different cultural background and learning style, different psychological pressure, psychological anxiety and other bad emotions that are not conducive to physical and mental health will be produced in the learning process. How to effectively teach Chinese calligraphy in the cross-cultural background has become an urgent problem to be solved. In order to improve the effect of Chinese calligraphy education, promote the common development of international Chinese education and mental health education. Based on the mental health perspective, this paper studies the development of the communication mode of Chinese calligraphy education for international Chinese education.

**Subjects and Methods:** Based on this, in order to improve the effect of integrating mental health education into Chinese calligraphy education in international Chinese education, and promote the integrated development of international Chinese education and mental health education. This paper first discusses the basic situation of international Chinese education; then it analyzes the basic connotation of Chinese calligraphy education. Then, from the perspective of psychology, this paper discusses and analyzes the psychological health factors of the communication mode of Chinese calligraphy education. Finally, based on the investigation of the audience of international Chinese education, this paper analyzes the specific mode of the future development of Chinese calligraphy education in China.

**Results:** This paper mainly adopts the form of questionnaire survey to study Chinese calligraphy education under international Chinese education. The results show that the highest proportion of male study time is less than one year, accounting for about 35%. While the highest proportion of female study time is 3-4 years, accounting for about 23%. With the growth of age, the study time of participants under the age of 20 was less than one year, accounting for about 77% of the total; the study duration of 20-30 year-old participants is less than one year, accounting for about 48%; the study duration of participants over 30 years old is the highest of 2-3 years, accounting for about 36%. At the same time, the most popular way of learning is an online course, which accounts for about 40% of women. The largest number of Chinese calligraphy communication carriers that participants are interested in is painting, which accounts for about 34% of participants aged 20-30.

**Conclusions:** This research not only provides a reference for improving the effectiveness of Chinese calligraphy education, but also makes an objective contribution to the future sustainable development of international Chinese education.

### Acknowledgements

This work was supported by a project grant from the Connotations and Values of Local Calligraphy Heritage in Zhongshan City, which used Xiaolan Town in Zhongshan City as an example. (Grant No.202357).

## LHMM23169 THE EFFECTS OF LOGO COLOR CHARACTERISTICS’ CHANGES ON CONSUMERS’ RESPONSES FROM THE PERSPETIVE OF CONSUMERS’ PSYCHOLOGICAL NEEDS

Ying Zhao^a^, Qinhai Ma^a^

*^a^ School of Business Administration, Northeastern University, Shenyang 110819, China.*

**Background:** In the digital age, online reviews have become a crucial factor in shaping consumer purchase decisions. As such, understanding the factors that influence the credibility of online reviews is essential for businesses that seek to attract and retain customers. Previous research has identified several factors that affect online review credibility, such as review valence, length, and source credibility. However, little is known about how the psychological needs of consumers may impact their perceptions of review credibility. This study aims to investigate the influence of psychological needs, such as the need for social validation and the need for self-enhancement, on consumers’ perceptions of online review credibility.

**Subjects and Methods:** A survey was conducted with 500 participants who had recently made an online purchase. Participants were asked to rate the credibility of a fictitious online review, which varied in terms of review valence, length, and source credibility. Participants also completed a questionnaire that measured their psychological needs, such as the need for social validation and the need for self-enhancement. Structural equation modeling was used to analyze the data and determine the extent to which psychological needs influenced consumers’ perceptions of online review credibility.

**Results:** The results of the study indicate that psychological needs play a significant role in consumers’ perceptions of online review credibility. Specifically, the need for social validation was found to positively influence consumers’ perceptions of review credibility, while the need for self-enhancement was found to negatively influence consumers’ perceptions of review credibility. Furthermore, the results suggest that the influence of psychological needs on review credibility may vary depending on the source credibility of the review.

**Conclusions:** This study provides important insights into the factors that influence consumers’ perceptions of online review credibility. The findings suggest that businesses can enhance the credibility of their online reviews by appealing to consumers’ psychological needs for social validation, while avoiding tactics that may trigger consumers’ need for self-enhancement. By understanding the psychological needs that underlie consumers’ perceptions of online review credibility, businesses can better tailor their review management strategies to attract and retain customers.

## LHMM23170 SUBJECTIVE WELL-BEING, ORGANIZATIONAL IDENTITY AND JOB PERFORMANCE OF CHINESE TERTIARY TEACHERS OF FOREIGN LANGUAGES IN THE CONTEXT OF INNOVATION AND ENTREPRENEURSHIP EDUCATION

Xianli Gao^a^, Li Li^b^

*^a^ School of Foreign Languages, Guangdong Pharmaceutical University, Guangzhou, Guangdong 510006, China, ^b^ School of Foreign Studies, Guangzhou University, Guangzhou, Guangdong 510006, China*

**Background:** With the development of positive psychology, well-being, as a healthy psychological mechanism, has become an important factor to explain employees’ behavioral attitudes and improve their job performance, which gradually enriches the relevant researches on teachers’ subjective well-being. Predicting job performance based on subjective well-being has always been a major concern in the field of organizational behavior. In view of this, based on the perspective of positive management, the purpose of this study was to explore the internal mechanism of the impact of tertiary teachers’ subjective well-being on job performance in the context of innovation and entrepreneurship by introducing organizational identity as a moderating variable.

**Subjects and Methods:** Two hundred and sixty-seven Chinese tertiary teachers of foreign languages in innovation and entrepreneurship education from 10 universities were chosen through random sampling. Questionnaires were employed to measure job performance, subjective well-being and organizational identity in this study, and the data was analyzed by the descriptive statistics, correlation and regression analyses to examine the internal mechanism of the impact of tertiary teachers’ subjective well-being on job performance in the context of innovation and entrepreneurship by introducing organizational identity as a moderating variable.

**Results:** The findings obtained from the study are as follows: (a) Subjective well-being and organizational identity were important factors affecting job performance of tertiary teachers of foreign languages, (b) Subjective well-being of tertiary teachers of foreign languages had a significant positive impact on job performance and organizational identity, (c) Organizational identity had a significant positive impact on job performance of tertiary teachers of foreign languages. Conclusions: The moderating mechanism between teachers’ subjective well-being and job performance is also a problem worthy of in-depth study. The propulsive relationship of organizational identity on subjective well-being and job performance showed that Chinese tertiary teachers of foreign languages could improve subjective well-being and job performance through organizational identity. Therefore, urging Chinese tertiary teachers of foreign languages to identify with institutions of higher learning and motivating teachers’ enthusiasm for innovation and entrepreneurship education was one of the effective ways to improve teachers’ job performance.

### Acknowledgements

This work was supported by Guangdong Provincial Undergraduate Teaching Reform and Quality Engineering Construction Project grants (No. GDJG2021295); Planning Subject for the 13^th^ Five Year Plan of National Education Sciences grants (No. 2020GXJK468); The 2^st^ Batch of Industry-University Cooperative Education Projects in 2022 by the Ministry of Education of China grants (NO. 220802193315018); Higher Education Special Funds (2021) of Department of Education of Guangdong Province.

## LHMM23171 A MULTIDIMENSIONAL STUDY ON THE INFLUENCING FACTORS OF INTERNET WORD-OF-MOUTH QUALITY BASED ON CONSUMER MOOD

Yu Wang^a^, Yanrui Jia^b^, Xiaoyan Xin^b^

*^a^ School of Management, Guangdong University of Science & Technology, Dongguan, Guangdong 523083, China, ^b^ School of Business Administration, Jimei University, Xiamen, Fujian 361021, China.*

**Background:** With the rise and development of e-commerce, the speed and scope of IWOM have spread more rapidly and extensively than traditional word-of-mouth. IWOM affected consumers’ shopping mood and decision-making behavior. The quality of IWOM is increasingly becoming a primary consideration in consumer shopping choices.

**Subjects and Methods:** The influencing factors of IWOM quality were analyzed from four dimensions such as IWOM recipients, channels, merchants and IWOM itself. The influencing factors of consumers mainly included consumers’ gender, personality, mood and the type of products they bought. The influencing factors analyzed from the communication channels were the type and authority of websites. From the merchants’ perspective, their influencing factors were the merchants’ reputation, after-sales service, payment methods and measures such as cashback to consumers in order to get good reviews, as well as the merchants’ handling of poor reviews given by consumers. The influencing factors of IWOM itself were mainly the directionality and the level of detail of IWOM. The questionnaire was designed according to the above four dimensions, and the proposed hypotheses were argued one by one through the empirical analysis method of questionnaire survey.

**Results:** The study found that the stronger the trust tendency in consumers’ mood, the higher the credibility of IWOM. Consumers’ gender has no effect on IWOM quality. Consumers are more influenced by IWOM quality for highly involved products. Website authority, merchants’ credibility and guarantee measures positively affect IWOM quality. Merchants’ means of misleading implementation negatively affect IWOM quality. The higher the level of detail of IWOM, the higher its IWOM quality. The type of online platform and the positive or negative nature of IWOM itself do not have significant effects on IWOM quality.

**Conclusions:** Based on the research results, certain reference suggestions are made for enterprises or businesses, consumers, governments, and communication channels. Enterprises should encourage and guide consumers to publish more detailed IWOM as much as possible. They should focus on and influence consumer mood and try to target their marketing to consumers with lower expertise and higher trust tendency. Consumers, as publishers or disseminators of IWOM information, should adhere to the principle of truthfulness and truthfulness, and publish true and effective IWOM. The government can formulate relevant legal norms and take measures to punish merchants to reduce false IWOM and enhance the objective accuracy of IWOM. Websites, as the main communication channels, should avoid the spread of false IWOM on websites by strengthening supervision.

## LHMM23172 TGF-BETA IS A KEY GENE IN THE OCCURRENCE OF HASHIMOTO’S THYROIDITIS COMBINED WITH OBESITY AND TYPE 2 DIABETES

Rui Yang^a^, Xinzhe Zhai^b^, Yi Wu^b^, Jianli Han^a^

*^a^ General Surgery Department, Shanxi Bethune Hospital, Shanxi, Taiyuan 030032, China, ^b^ Third Hospital of Shanxi Medical University, Shanxi Bethune Hospital, Shanxi Academy of Medical Sciences, Tongji Shanxi Hospital, Shanxi, Taiyuan, 030032, China.*

**Background:** Metabolic diseases caused by obesity are diverse, and many large population studies have reported a direct correlation between obesity and the occurrence of various diseases, which can affect the function of multiple endocrine organs including thyroid function. The relationship and mechanism between Hashimoto’s thyroiditis (HT) and type 2 diabetes mellitus (DM) as two common obesity-related complications remain yet unclear.

**Subjects and Methods:** The National Center for Biotechnology Information (NCBI) database was searched for GEO datasets (https://www.ncbi.nlm.nih.gov/geo), and datasets GSE18470 and GSE138198 were selected for data analysis. After analyzing the differentially expressed genes between the groups, the results were submitted to the DIANA TOOLS website and the MirPath tool was used for pathway enrichment analysis. The KEGG pathway enrichment results were selected as the data for further analysis, and key genes were identified.

**Results:** Compared with simple obesity, there are more associated genes in obese patients with type 2 diabetes (DM) and HT, suggesting that type 2 DM may increase the chance of obese patients developing HT. The TGF-beta signaling pathway and Proteoglycans in cancer pathways contained the most genes, with 10 genes each. The TGF-beta signaling pathway (p=1.776067e-06) ranked higher than the Proteoglycans in cancer pathway (p=8.47584e-05), suggesting that the TGF-beta signaling pathway may be the key pathway for obese patients with type 2 DM to develop HT.

**Conclusions:** This study not only found that there are more associated genes in obese patients with type 2 DM and HT, but also suggested that the TGF-beta signaling pathway is the key bridge for the occurrence of these two conditions. The 10 associated genes in the TGF-beta signaling pathway identified in this study can be the focus of future research. Identifying the possible mechanisms leading to the occurrence of HT in obese patients with type 2 DM can provide feasible treatment options for subsequent HT treatment.

### Acknowledgments

This study was funded by Shanxi Province “136 Revitalization Medical Project Construction Funds” (2021YZ05).

## LHMM23173 ANALYSIS OF FACTORS INFLUENCING CONSUMERS’ WILLINGNESS AND MOOD TO USE FACE SWIPE PAYMENT IN THE CONTEXT OF DIGITIZATION

Yanrui Jia^a^, Weiwei Fan^a^, Qingqing Ma^a^

*^a^ School of Business Administration, Jimei University, Xiamen, Fujian 361021, China.*

**Background:** With the rapid development of Internet technology, a wide variety of digital information technologies have gradually integrated into people’s lives and effected consumers’ usage willingness and mood. Consumers have begun to expect payment methods to become more secure, convenient, and intelligent. Then various payment methods based on human biometric for identity verification have gradually emerged.

**Subjects and Methods:** This study combined initial trust theory and perceived risk theory to investigate the effects of a total of five factors such as structural assurance, trust propensity, company reputation, initial trust, and perceived risk on consumers’ psychological willingness and mood to use face swipe payment. Structural assurance is the guarantee of consumers’ environmental security when using face payment. Trust propensity is the degree of consumers’ personal tendency to believe in something new. Company reputation refers to the reputation of the backend company of face payment. Initial trust is the degree of consumers’ trust in face payment in the beginning. Perceived risk refers to consumers’ psychological expectation of unknown negative effects brought by using face payment. With the above five factors as independent variables and consumers’ willingness and mood as dependent variables, the research model was constructed and the hypotheses were verified by using questionnaires and SPSS data analysis.

**Results:** Structural assurance, trust propensity, company reputation, and initial trust significantly and positively affect consumers’ willingness and mood to use face payment. Perceived risk significantly and negatively influenced consumers’ willingness and mood to use. The details are as follows specifically. Higher structural assurance enhances consumers’ initial trust in face payment and thus consumers’ willingness to use it. The characteristic of being more likely to believe in something new positively drives consumers’ initial trust in face payment. The good reputation of the company makes consumers more willing to use the company’s face payment technology. The higher the initial trust, the stronger consumers’ willingness to use face payment. The higher the perceived risk level of face payment, the lower the willingness to use.

**Conclusions:** Valuable reference suggestions were proposed for enterprises to influence consumers’ willingness and mood to use face swipe payment. They need to develop consumer-related protection mechanisms and establish comprehensive information security to maximize the safety of consumers’ personal information and property. Enterprises should pay attention to maintaining their good reputation. Providing consumers with high-quality products and good services is the basis for enterprises to establish good reputation. They should emphasize the advantages of payment technology to attract consumers. When promoting face payment, enterprises should emphasize its convenient and safe functional advantages. They need to strictly comply with the laws and regulations related to face payment and protect consumers’ rights and interests, which is the way to long-term development of enterprises.

## LHMM23174 RESEARCH ON THE ADJUSTMENT OF WORK STRESS AND PSYCHOLOGICAL ANXIETY OF CHINESE ENTREPRENEURS

Lingling Tong^a^

*^a^ School of Social Service and Development, Zhengzhou Normal University, Zhengzhou, Henan, 450000, China.*

**Background:** Chinese entrepreneurs are known as the world’s most hardworking, indomitable and persistent qualities. They not only have the ability to withstand tremendous pressure and deal with complex practices, but also able to keep a balance between their career, family and self-growth. In fact, many Chinese entrepreneurs have serious psychological and health problems due to their work stress and psychological anxiety.

**Subjects and Methods:** From January 2022 to February 2023, 100 entrepreneurs and executives from different fields and industries were selected in Beijing, Shenzhen, Hubei, Xi ‘an, Zhengzhou and other places for case interviews, observation, visits and questionnaires.

**Results:** In recent years, with the integration of the world economy, the embedding of the Internet economy and the vigorous development of the digital economy, the pace is getting faster and faster, which makes the work pressure and physical and mental health condition of Chinese entrepreneurs cannot be ignored. Many entrepreneurs have physical health problems due to the work pressure and psychological anxiety cannot be well alleviated, such as sleep disorder, fatty liver, chronic gastritis, migraine, alcohol paralysis, low performance, depression, Job burnout, etc.

**Conclusions:** This paper analyzes the influencing factors of entrepreneur’s stress on individual psychological anxiety and establishes a structural model of the influence of entrepreneur’s stress on emotion. Based on the empirical research data, this paper proposes the adjustment strategies and means of entrepreneur’s job stress.

**Key words:** Chinese entrepreneur; psychological anxiety; work pressure; affective disorder; health indicators

### Acknowledgements

1.This paper is one of the key research results of the undergraduate Teaching Engineering and Teaching Reform Research project of Zhengzhou Normal University, Research on the Cultivation Model and Standard Evaluation of Undergraduate Excellent Teachers (No.:[JSJYZD-001211573].

2. This paper is a general project of Humanities and social sciences in colleges and universities of Henan Provincial Department of Education, “Research on the impact of digital Economy on High-tech Industry Innovation” phased results.

## LHMM23175 THE APPLICATION OF MIXED TEACHING BASED ON QQ GROUP+TENCENT CONFERENCE IN THE MENTAL HEALTH EDUCATION OF POOR STUDENTS IN THE CONTEXT OF COVID-19

Fei Sun^a^, Ziwei Xu^a^

*^a^ Yuzhang Normal University, Nanchang 330103, China.*

**Background:** Poor students will encounter many problems on their development path, such as psychological pressure, life pressure, learning pressure, employment pressure, and so on. Self regulation of poor students in the face of these pressures could effectively promote the development of poor students. In 2022, affected by the COVID-19, the competent education department successively issued the requirement of “suspending teaching and learning”. The impact of mental health education on poor students was particularly evident during the epidemic therefore, it is necessary to carry out modernization, reform and innovation of mental health education for poor students.

**Subjects and Methods:** Constructing online teaching courses for mental health education through the mixed teaching method of QQ group + Tencent conference is significant for the development of poor students. This teaching mode is conducive to mental health course education and innovation and is conducive to cultivating qualified high-quality talents. The blended teaching using QQ Group + Tencent Meeting was conducive to solving the problem of not being able to carry out traditional classroom teaching during the epidemic, helped to ensure the health of students and reduced the risk of epidemic contact and crossover. For poor students, online mental health education was even more necessary during the epidemic.

**Results:** Online mixed teaching includes four parts: course introduction, teaching new courses, classroom summary and consolidation of exercises after class. After the courses, it is necessary to reflect on the teaching effect of the course, summarize and accumulate experience. Online mixed teaching is an important guarantee for curriculum teaching in the context of the epidemic. Online hybrid teaching plays an important role in improving teaching quality. Online hybrid teaching strategies can play an important role in stimulating students’ interest in learning.

**Conclusions:** Under the background of the epidemic, using the mixed teaching method of QQ group + Tencent conference can achieve good results in the education and teaching of poor students, and this teaching method can improve the teaching quality and stimulate students’ interest in learning.

### Acknowledgments

The 2022 Special Task Project for Humanities and Social Sciences Research of the Ministry of Education (Research on University Counselors) “Research on the Impact of University Financial Assistance on the Development of Poor Students and Its Influencing Factors” (Project host: Sun Fei, Project Number:22JDSZ3125).

## LHMM23176 MASS MEDIA, BODY CONSUMPTION AND PSYCHOLOGICAL ANXIETY IN MARTIN AMIS’S MONEY: A SUICIDE NOTE

Wenju Han^a^

*^a^ College of Foreign Languages and Literature, Northwest Normal University, Lanzhou, China.*

**Background:** This paper studies Martin Amis’s masterpiece Money: a Suicide Note, aims to reveal the socioeconomic and psychological impact of mass media’s consumption of body, such as the psychological anxiety.

**Subjects and Methods:** This paper studies the novel from the perspective of media ecology, Baudrillard’s social theories on the consumer society, body in the consumer culture, psychological anxiety and etc.

**Results:** By focusing on the under-examined correlation between mass media, body consumption and anxiety in Amis’s Money, this paper finds out that unlike orality and typography which produce abstract image of body in the mind of different people, the visual media such as TV produce concrete standards of body images, which highlight the importance of appearance, wiping out the uniqueness in people’s appearance. By presenting a vast amount of visual images of the body through make-up, plastic surgery, lights, dressing and etc, the visual media construct ideal and standard images of male and female body, which foreground forever youth, beauty and health that are in fact impossible, and body image is associated with masculinity, femininity, self-discipline and social status, arousing people’s consciousness of their body and anxiety, creating their desire of consumption to maintain their body, thus the mass media reach the goal of promoting consumerism and selling the goods. In order to meet or approximate the criteria of beauty set by visual media, people take various instrumental means to build their body. In this process, they accept the capital and power’s discipline of their body willingly.

**Conclusions:** In conclusion, this paper argues that the male and female body is commodified by mass media, causing great anxiety. In Amis’s Money, under the discipline of media culture, John Self, the protagonist, is preoccupied with his appearance and full of anxiety. Self is so willing to do everything to preserve his body that he would rather quit reading because it hurts his body, which causes anti-intellectualism. And the male characters model after the film stars in their body image and even undergo plastic surgery. So in the age of visual media and high technology, body is oppressed and damaged to a deeper degree. The female characters in Money such as Selina, Caduta self-monitor their body to meet the standards of beauty set by the mass media. Therefore, mass media strengthen the commodification of male and female body, which reduces the value of female to sex and consumption. Instead of being liberated, the human body is still oppressed by aesthetic violence in the contemporary consumer society.

### Acknowledgments

This work was supported by the [Northwest Normal University] under Grant [number 2916]

## LHMM23177 RESEARCH HOTSPOTS AND FRONTIER TRENDS IN HUMAN ENHANCEMENT TECHNOLOGIES: BIBLIOMETRICS AND VISUAL ANALYSIS

Hong Chang^a^, Chuanping Lei^b^, Qingda Li^a^, Junling Ma^c^, Li Jia^d^

*^a^ School of Marxism, Zhongkai University of Agriculture and Engineering, GuangZhou, Guangdong 510550, China, ^b^ School of Marxism, Sun Yat-sen University, GuangZhou, Guangdong 510275, China, ^c^ School of Humanities and Management, Guangdong Medical University, Dongguan, Guangdong 523808, China. ^d^*

HC and CL contributed equally to this study.

**Background:** Human enhancement technology can help people solve better social, economic, and medical problems and improve the social and medical environment. Human enhancement technology aim to improve human productivity through retinal implants, cochlear implants, and other audio-visual enhancement technologies, brain enhancement technologies such as drugs, brain implants, and brain-computer interfaces, and physical enhancement technologies such as exoskeletons and quality of life, thus promoting the progress of society.

**Subjects and Methods:** This study aims to explore the research hotspots and cutting-edge trends of human enhancement technologies in the past 20 years. This study collected literature on human enhancement technologies from the Web of Science database. This study used bibliometric and visual analysis methods to conduct the study. The main paths of knowledge evolution of human enhancement technologies were mapped using CiteSpace visualization software. One of the main ways to perform word contribution analysis is to extract keywords, abstracts, and other relevant information from the dataset and draw a visual knowledge map to determine the relationships between topics in the disciplines represented in the dataset. The literature and the degree of deviation from the current knowledge structure are studied according to the structural transformation model in the citation network.

**Results:** In the last 20 years of human enhancement research, this study identified 159 highly productive scholars and ten collaborative teams with large research sizes. Cluster analysis revealed that the Research was focused on “cognitive enhancement,” “ethical enhancement,” “emotional enhancement,” and “disease and technology enhancement ethics”. There are three shifts in the research themes and directions of human augmentation research, from disease treatment research to general human augmentation research; from medical examination of human cognitive augmentation to ethics of moral and emotional augmentation research; and from human drug augmentation research to biotechnological augmentation research under convergence technology. The frontier literature on human enhancement technology research involves multiple disciplines such as medicine, neuroscience, molecular biology, psychology, ethics, and sociology, and future Research is bound to focus on the convergence and innovation of multiple technologies, multiple topics, and multiple fields.

**Conclusions:** The results of this paper can provide necessary references for researchers engaged in human genetic engineering, drugs, biomedical technology and other aspects of human enhancement technologies, and provide necessary guidance for future research directions.

### Acknowledgements

This work was supported by a project grant from “Human Enhancement Technology Risk Research: Causes, Models and Protocols”, Ministry of Education, Humanities and Social Sciences Research Youth Project (Grant No. 22YJCZH012); “A Philosophical Hermeneutic Study on Emerging Human Enhancement Technologies”, Guangdong Philosophy and Social Sciences Planning Project (Grant No. GD20CZX01); Teaching reform Project of Guangdong Medical University “Research on Humanistic Quality Training Path of Medical Students in the Vision of Collaborative Education” (Grant No.1JG21083); Guangdong Provincial University characteristic innovation project “Construction of Humanistic Medicine from the Perspective of Life Culture” (Grant No.2021WTSCX034).

## LHMM23178 BIBLIOMETRIC ANALYSIS OF MENTAL HEALTH LITERATURE OF FEMALE TEACHERS IN CHINESE UNIVERSITIES BASED ON CITESPACE

Cui Shan^a^, Qin Zhou^a^, Wenchao Gao^b^

*^a^ College of Civil Engineering, Putian University, Putian, Fujian 351131, China, ^b^ CNNC Huachen Construction Engineering Co., Ltd, Putian, Fujian 351100, China.*

**Background:** With the rapid rise of China’s social economy from 1998 to 2022, university teachers constantly face dual challenges from society and profession. Their mental health is constantly affected, especially female teachers in Chinese universities. This paper which analyzes the research results on the mental health of female teachers in universities from 1998 to 2022, reveals the development law of the research, summarizes and analyzes the research contents.

**Subjects and Methods:** On the platform of China National Knowledge Network (CNKI), a total of 93 valid literatures were obtained, through the advanced search of the two subject words “female teachers in universities” and “mental health”. CiteSpace software is used to cluster analysis for the sample literature. It mainly contains the cluster analysis of literature authors, research institutions and keywords.

**Results:** (1) From the annual trend of research results, the research development trend in the field of mental health of female teachers in universities can be roughly divided into three periods. First, the initial exploration period, paying attention to mental health investigation. Second, the rapid growth period, focusing on the psychological characteristics and influencing factors of female teachers in universities. Finally, the slow growth period, caring on the analysis of coping strategies in mental health problems and the improvement of career happiness. Moreover, the number of published papers peaked in 2008. (2) From the perspective of literature sources, key authors and research institutions, the research results in the field of mental health of female teachers in universities have been published in a wide range of journals, especially in mental health, education and teaching, higher education research field. (3) From the perspective of research contents, hotspots, frontiers and trends, the research contents in the field of mental health of female teachers in universities include the investigation of mental health status, influencing factors of mental health, and strategies for improving mental health. (4) Research hotspots focused on the female teachers in universities mental health status, and strategies to improve mental health problems. (5) Research frontiers can be roughly divided into three research periods.

**Conclusions:** (1) From 2014 to now, the research popularity in the field of mental health of female teachers in universities has declined, and the quantity and quality of research results in this field have great room for improvement. (2) In the future, new technologies and methods, such as simulation prediction model and big data, can be introduced into the research methods in oder to improve the practicability and innovation of the research results in this field. (3) China’s first-tier cities have more developed economies, and more students, higher school standards, and greater pressure on teachers’ life and career. Therefore, the future research area can be further focused on China’s first-tier cities. (4) At present, most researchers and research institutions have a lot of room for future cooperation. The further expansion of cooperative relations is conducive to the overall development of China’s higher education.

### Acknowledgments

This work was supported by a project grant from Education and Research Project for Young and Middle-aged Teachers of Fujian Province (Social Science) (Grant No.JAS21249); Education Science Planning Project of Fujian Province (Grant No.FJJKC2020-029); University-level Education Reform Project of Putian University (Grant No. JG2022068).

## LHMM23179 ANALYSIS OF SU SHI’S PRACTICE IN ANTI EPIDEMIC MEDICINE

Limin Zhang^a^, Xiaofeng Luo^b^

*^a^ Sichuan University Jinjiang College, Pengshan, Sichuan, 620860, China ^b^ Sichuan Police College, Luzhou, Sichuan, 646000, China.*

**Background:** At the beginning of 2020, the COVID-19 began to sweep across China from Hubei, and the public health safety of Huanggang in Hubei was the first to bear the brunt. Huanggang was known as Huangzhou in ancient China. In 1079 AD, Su Shi, who was selected by the French newspaper Le Monde as one of the “heroes of the millennium” who influenced the world, was demoted to Huangzhou due to the Wutai poetry case. When the plague broke out, he announced the anti epidemic prescription “Sheng San Zi Fang” to the public, and countless people survived!

**Subjects and Methods:** During his demotion, Su Shi encountered plagues many times. Due to his rich epidemic prevention experience and effective public health management measures, he repeatedly saved people from distress. In terms of Su Shi’s anti epidemic practice, effective public health management measures are as follows: During the initial stage of the epidemic, go deep into the epidemic area, truthfully report to the superior, and bought time for the government’s timely rescue; Pay attention to drinking water hygiene and prevent accidents before they occur; During the epidemic, he actively promoted the use of prescription drugs to treat the epidemic, while donating funds to establish the “Anle Fang” equivalent to the prototype of a modern square cabin hospital to carry out isolation and self rescue, And integrate civil forces to assist in combating the epidemic; After the epidemic, it is recommended that the government exempt taxes and provide relief to restore people’s livelihood. He also requires that officials who fail to perform their duties in the prevention and control of the epidemic be held accountable, and rectify the official atmosphere to ensure the normal operation of state power in the future.

**Results:** In the early stages of the epidemic, due to the implementation of timely and effective public health prevention measures, the source of infection of the epidemic was quickly cut off; During the epidemic, Public health management measures are timely and effective, fully mobilizing the forces of all sectors of society, saving countless people, and minimizing the harm of the epidemic; After the epidemic ended, economic remedial measures were taken to appease the people’s livelihood, and the lives of the people and government functions gradually returned to normal operation.

**Conclusion:** Su Shi’s series of anti epidemic practices from prevention to treatment are also scientific and reasonable, and still have important reference significance for the prevention and treatment of contemporary epidemic situations; As a great scholar, he also had a profound understanding of traditional Chinese medicine; Whether selflessly donating anti epidemic prescriptions, compiling medical books, or generously donating money to build a shelter hospital to fight the epidemic, these fully reflect his people-oriented, benevolent and pragmatic medical feelings.

## LHMM23180 THE ROLE OF TRADITIONAL CHINESE OPERA INHERITANCE ON RELIEVING STUDENTS’ PSYCHOLOGICAL ANXIETY

Xinxia Fang^a^

*^a^ Department of Music, Yancheng Teachers University, Yancheng, 224007, China.*

**Background:** China’s National Mental Health Development Report (2021-2022) shows that the primary factors affecting depression and anxiety levels are age and income, and among which young people are the high-risk groups. According to the report and some relevant statistics, young people are faced with many troubles and challenges such as academic pressure, employment pressure, economic pressure, emotional pressure and so on. Mastering the ability to adjust emotions or words and deeds such as self-regulating depression, dissatisfaction and irritability, and learning to maintain an optimistic and peaceful attitude is a necessary quality to relieve psychological anxiety. What’s more, traditional Chinese culture contains rich and profound psychological values, among which the representative works, such as Jung’s theory of analytical psychology, are greatly inspired by traditional Chinese culture. The Chinese government attaches great importance to the inheritance of traditional Chinese opera, and has issued relevant policies such as “Opinions on the Implementation of traditional Chinese Opera on Entering the Campus”, “Opinions on Comprehensively Strengthening and Improving School aesthetic education in the New Era”, “Compulsory Education Curriculum Plan and Curriculum Standards (2022 Edition Curriculum Standard)” and so on to further encourage the inheritance and development of traditional Chinese opera, which promotes the mental health development of students to a large extent. In the era of new media, it should be remembered that only through continuous innovation can the inheritance methods and means of traditional Chinese opera achieve more remarkable results.

**Subjects and Methods:** Relying on its advantages of low cost, immediacy, fast interaction, etc., the new media has become an important communication platform for traditional Chinese opera. Taking TikTok and Bilibili as an example, the author uses a combination of quantitative analysis and qualitative research to carry out research, and then analyzes the characteristics and trends of people’s cognition, aesthetics and other aspects of traditional Chinese opera, so as to put forward targeted plans for primary and secondary school students to improve their cultural spiritual literacy, enhance the sense of national cultural identity and strengthen their mental health.

**Results:** According to some relevant surveys, there are still insufficiency in netizens’ awareness of traditional Chinese opera inheritance and knowledge reserve. In order to promote the physical and mental health of the general public through the dissemination of traditional Chinese opera, it is really necessary to cultivate their appreciation ability of traditional Chinese opera from an early age, strengthen the promotion of aesthetic education infiltration practice activities on primary and medium-sized campuses, and realize the whole process of aesthetic education for the whole people.

**Conclusions:** The peaceful, harmonious, tenacious and optimistic aesthetic characteristics embodied in the traditional Chinese opera will not only have a positive guidance to the audience’s psychology, but also strengthen their sense of national cultural identity and enhance their national cohesion. Additionally, while inheriting traditional Chinese culture, we should realize that the personality development and mental health of students and always bear in mind that the cultivation of aesthetic consciousness should be cultivated from an early age. What counts most is that, rich and colorful traditional Chinese opera aesthetic education activities should be designed according to the psychological characteristics and learning ability of students of different ages.

## LHMM23181 APPLIED RESEARCH IN ART THERAPY FOR MENTAL HEALTH

Shuhan Hu^a^

*^a^ School of Visual Communication, Lu Xun Academy of Fine Arts, Liaoning Province, China.*

**Background:** Art therapy is a separate discipline born in Europe in the 1930s and 1940s from the fusion of art and psychology as a form of creative expression for therapeutic purposes. The art therapy process consists primarily of the creation of art as therapy itself, and the resulting interaction between the practitioner and the subject. Art therapy has been widely used in Europe and the United States and has been shown to have a positive effect on a person’s mental health and to alleviate many mental illnesses through art therapy, while art therapy is still in its infancy in theory and practice in China.

**Subjects and Methods:** This paper first discusses the relationship between art creation and mental health, and then goes through the applications of art therapy in life, medicine and society respectively, pointing out through case studies that art creation can release psychological stress, relieve pain, alleviate illness and make a prominent contribution to mental health, followed by a brief overview of the current state of development of art therapy in China.

**Results:** Artistic creation in psychological healing is not only an expression of one’s emotions, but also a clear contribution to physical and mental health.

**Conclusions:** Art therapy is an art of communication methods that has made an outstanding contribution not only to the psychological meaning of visual symbolism, to the relationship between creativity and health, but also to alternative therapeutic approaches. In the future this discipline will play an even greater role in building the field of mental health and has even greater potential to be explored. It has a role to play in human understanding of the self, healing and healthy living that cannot be ignored.

## LHMM23182 A STUDY ON THE INFLUENCE OF NETFLIX ON CONSUMERS’ PURCHASE INTENTION AND MOOD BASED ON SOCIAL MEDIA COMMUNICATION

Yanrui Jia^a^, Luxia Lin^a^

*^a^ School of Business Administration, Jimei University, Xiamen, Fujian 361021, China.*

**Background:** With the rapid development of Internet and information technology, a number of “net celebrities” with distinctive features and economic benefits have emerged on social media platforms. Through social media platforms, net celebrities conveyed information about corporate brands or products to consumers, which improved their visibility and profoundly affected people’s online consumption intention and mood. It is an emerging trend for companies to rely on social media platforms to influence consumers’ shopping behavior through the Netflix model.

**Subjects and Methods:** This paper firstly explained the theoretical foundation of social media and the current situation of Netflix, and analyzed the influencing factors of consumers’ online purchase intention and mood, then deduced the influencing factors of consumers’ purchase behavior under the Netflix model. Secondly, through the empirical study of quantitative analysis, the model of the influence of Netflix communication on consumers’ purchase intention was constructed, with consumers’ purchase intention as the dependent variable and with the product factor, the Netflix factor and the perception factor as independent variables. Finally, relevant conclusions were drawn by using questionnaires, SPSS data correlation analysis and regression analysis.

**Results:** The factors that positively influence consumers’ purchase intention and mood under Netflix social communication include product quality, users’ product evaluation, interactive attitude of Netflix, multi-channel publicity of Netflix, and consumers’ perceived value of the product. The specific descriptions are as follows. The higher the product quality, the better the consumer’s shopping experience. The better the user’s product evaluation, the stronger the consumer’s purchase intention. The good interactive attitude can meet the consumer’s demand. The multi-channel publicity both reduces the cost and expands the effect of Netflix. The higher the consumer’s perceived value of the product, the stronger the consumer’s purchase intention. In contrast, product information and perceived risk have no positive influence on consumers’ purchase intention, i.e., specific and accurate product information does not have a significant positive influence on consumers’ purchase intention, and consumers’ perceived risk of the product has no significant negative influence on purchase intention.

**Conclusions:** Marketing suggestions for enterprises to effect consumers’ purchase intention and mood are presented. Firstly, enterprises can use differentiated Netflix groups to improve consumers’ perceived value of their products. Secondly, focus on interactive marketing. Net celebrities should publish content according to consumers’ interests, share their own real feelings of using the products, and actively interact with consumers on platforms such as video, live streaming or social. Thirdly, improve product quality. Companies can enhance consumer participation, so that consumers can participate in the process of designing and improving products. Finally, establish channel advantages. Enterprises should choose the right social media platform for cooperation according to their own product and industry characteristics to establish their long-term channel advantages.

## LHMM23183 SPEECH REHABILITATION: RESPIRATORY AND PROSODIC FEATURES IN READING LITERATURE IN MANDARIN

Feng Yang^a^

*^a^ College of Chinese Language and Culture, Zhejiang Yuexiu University, Shaoxing, Zhejiang 312000, China.*

**Background:** This paper combines respiratory and acoustic signals from a physiological and acoustic perspective to write a program that divides the prosodic hierarchy based on the magnitude of respiratory reset amplitude, explores the corresponding relationship between the respiratory reset amplitude and the prosodic hierarchy, also explores the respiratory physiological mechanisms of acoustic features such as pauses, pitch dips, and amplitude dips in speech flow. To provide methods for speech rehabilitation training, improve patients’ speech naturalness and fluency, enhance the effect of speech rehabilitation training. It provides a new method and thought for speech rehabilitation.

**Subjects and Methods:** In this paper, the acoustic analysis method is used to record the voice and respiratory signals of 80 short passages read by four announcers. Write a Matlab program to analyze the four signals. Firstly, automatically mark the various respiratory reset positions of the chest and abdomen respiratory signals, calculate parameters such as respiratory reset amplitude, inhalation and exhalation duration, and then divide the prosodic hierarchy based on the magnitude of respiratory reset amplitude, mark the boundaries of prosodic units, and finally output parameters such as the duration, fundamental frequency, and amplitude of each prosodic unit.

**Results:** The results show that there is a corresponding relationship between the repositioning amplitude of respiration and the prosodic boundary in discourse reading. (1) In discourse reading, the amplitude of respiratory reset can be divided into three levels. The first level respiratory reset corresponds to paragraph or complex sentence boundaries, the second level respiratory reset corresponds to clause boundaries, and the third level respiratory reset corresponds to prosodic phrase boundaries. (2) The pause coincides with the inspiratory segment, and the reason for the pause is to inhale for pronunciation, while the phonetic segment coincides with the expiratory segment. (3) Along with the pitch dip, the amplitude dip occurs simultaneously within a breathing cycle. The dip is caused by the continuous exhalation of air flow from the lungs during the exhalation pronunciation stage, resulting in a decrease in air pressure. The focus stress is mainly manifested as an increase in pitch. Phonological fluency is related to respiratory reassignment, which can be used for phonological rehabilitation training.

**Conclusions:** The physiological reason for the pause is the completion of inspiratory movement, and the downward pitch and amplitude decline are caused by the attenuation of the airflow pressure in the expiratory segment of the lungs. In the process of language rehabilitation training, patients should master the correct breath control method, and adopt different sizes of breath resets according to the pause position of the discourse, so as to improve the effect of language rehabilitation training and the naturalness of the pronunciation of reading training.

### Acknowledgments

This work was supported by Major Projects of National Social Science Foundation ‘Research on the contact and integration of Chinese languages’ (Grant No.22&ZD213).

## LHMM23184 DIAGNOSTIC VALUE OF SERUM SAA, IL-6, AND HS-CRP IN PATIENTS WITH INFLUENZA A (H3N2)

Bin Liu^a^, Huiqiong Hu^b^

*^a^ Jingzhou Central Hospital, Jingzhou 434020, China, ^b^ Hubei College of Chinese Medicine, Jingzhou 434020, China.*

**Background:** To provide reference for the diagnosis and treatment of influenza A (H3N2) in clinic and to reduce the mortality in patients with influenza A (H3N2) through the study on the level change of serum amyloid A (SAA), interleukin-6 (IL-6) and hypersensitive-C-reactive protein (hs-CRP) in patients with influenza A (H3N2).

**Subjects and Methods:** 177 patients who had the clinical manifestations of influenza and were positive for H3N2 subtype influenza virus in the nucleic acid testing of the nasopharyngeal swab or sputum samples in Jingzhou Central Hospital from January 1, 2022 to December 30, 2022 were selected as the influenza A (H3N2) group. Another 169 subjects who underwent health examinations during the same period were selected as the healthy control group. The venous blood samples were collected in the influenza A (H3N2) group and healthy control group to detect the levels of SAA, IL-6, and hs-CRP in peripheral blood. The levels of the SAA, IL-6, and hs-CRP in the influenza A (H3N2) group and healthy control group were analyzed, and area under the curve (AUC), cutoff value, sensitivity, specificity, and Yoden index of single detection of SAA, IL-6, and hs-CRP and joined detection (SAA + IL-6+ hs-CRP) were compared to analyze the diagnostic value of single detection and joint detection on the diagnosis and treatment of influenza A (H3N2).

**Results:** The levels of SAA, IL-6 and hs-CRP in influenza A (H3N2) group were 46.49 ± 29.08 mg/L, 22.03 ± 11.59 pg/mL and 14.88 ± 7.80 mg/L, respectively, significantly higher than those in healthy control group. The differences of SAA, IL-6 and hs-CRP between the two groups were statistically significant in test (all P<0.05). The area under the curve (AUC), cutoff value, sensitivity, specificity and Yoden index of single detection of SAA, IL-6 and hs-CRP in influenza A (H3N2) group showed that the cutoff value of the serum SAA was 12.40 mg/L with AUC, sensitivity, specificity, and Yoden index of 0.952, 0.819 and 0.988, respectively, significantly higher than that of IL-6 and hs-CRP; the AUC, sensitivity, and Yoden index of the joint detection (SAA + IL-6+ hs-CRP) were 0.964, 0.938, and 0.820, respectively, higher than those in the single detections; however, the specificity of the joint detection (SAA + IL-6+ hs-CRP) in influenza A (H3N2) group was 0.882, lower than that in the single detection.

**Conclusions:** Markedly increased level of serum SAA can be found in patients with influenza A (H3N2). The joint detection of of SAA, IL-6 and hs-CRP is helpful for the diagnosis and evaluation of conditions of patients with influenza A (H3N2), which can be used as the reference for the diagnosis and treatment of influenza A (H3N2) in clinic with significant clinic value.

## LHMM23185 THE RESEARCH OF INTEGRATING PHYSICAL EDUCATION INTO SPORTS PSYCHOLOGY HEALTH EDUCATION IN COLLEGES AND UNIVERSITIES

Liqiu Lin^a^, Longhui Zhou^b^

*^a^ College of Physical Education and Health, Guangdong Vocational College of Science and Technology, 519090, Zhuhai, Guangdong, China, ^b^ Physical Education, Guangdong Jiangmen China Medicine College, Jiangmen 529000, Guangdong, China.*

**Background:** To briefly discuss related strategies of integrating sports psychology into physical education teaching in colleges and universities, so as to provide experience reference and help for practitioners in related industries, and promote the development of sports literacy of students in colleges and universities. This paper discusses the significance of integrating physical education into sports psychology health education in colleges and universities, and puts forward relevant countermeasures accordingly, in order to provide reference for physical education teachers in colleges and universities.

**Subjects and Methods:** Questionnaire survey, logical analysis, expert interview and empirical research were used to explore the significance of integrating physical education into sports psychology health education in colleges and universities, and corresponding countermeasures were put forward.

**Results:** In the physical education teaching practice of colleges and universities, it is necessary to infiltrate and integrate the related content of sports psychology, so as to continuously enhance the mental health quality in the teaching process and achieve excellent results in sports training. Constantly improve the ability of thinking control and self-regulation control of college students, to improve the physical psychology of college students to lay the foundation. In order to realize the cultivation of mental health quality of young people in colleges and universities in sports, the organization and development of sports should be guided by the law of physical and mental development of young people and the law of physical education teaching, take positive emotion, experience success, democratic autonomy and individual guidance as the basic principles, and take the organic, moderate and flexible penetration in the process, content and method of sports as the main strategy, so as to be practical. Training design and effective classroom management as process control to construct the physical sports youth mental health quality training path.

**Conclusions:** By analyzing the correlation between the discipline orientation of sports psychology and the connotation of sports core literacy, the promoting effect of sports psychology research content on sports core literacy, and how to cultivate sports core literacy in sports psychology teaching, this paper tries to clarify the role of sports psychology curriculum in promoting students’ sports core literacy, so as to better cultivate students’ knowledge of sports psychology. Ability to solve problems related to sports practice.

## LHMM23186 RESEARCH HOTSPOTS AND TRENDS IN MEDICAL HUMANITIES BASED ON BIBLIOMETRICS

Li Jia^a^, Manyi Chen^b^, Junling Ma^a^, Cheng Yu^c^, Chuanping Lei^d^

*^a^ School of Humanities and Management, Guangdong Medical University, Dongguan, Guangdong 523808, China, ^b^ Nanling Corridor Country Revitalization Research Institute, Xiangnan University, Chenzhou 423000, China, ^c^ School of Medicine, Sun Yat-sen University, Shenzhen 518107, China, ^d^ School of Marxism, Sun Yat-sen University, GuangZhou, Guangdong 510275, China.*

LJ and MC contributed equally to this study.

**Background:** The frequent occurrence of doctor-patient conflicts and medical disputes in the process of healthcare system reform has made medical humanities research a critical issue in public and scientific research. Despite the increasing number of studies on medical humanities, there is a lack of comprehensive and systematic combing based on bibliometrics.

**Subjects and Methods:** This study uses author collaboration, institutional collaboration, keyword co-occurrence, cluster analysis, Structural Variation Analysis, bipartite graph overlay, and country and keyword bimodal mapping construction based on medical humanities-related research literature data from 1974-2022 in the Web of Science database to comprehensively and systematically sort out medical humanities.

**Results:** The study found that the authors in the field of medical humanities research have been working on the following topics: scientific cooperation, research hotspots, and frontiers, knowledge base evolution trends, and prediction of potential, influential literature. It was found that author co-location and institutional cooperation in medical humanities research are relatively close, forming a complex cooperative network. Institutional research is more active in countries such as the United States, Canada, and the United Kingdom. The knowledge base and significant fields in medical humanities research show diversified development, and the intersection of subject areas is becoming stronger. Hot research issues represented by medical humanities education, medical humanities care, doctor-patient communication, and medical ethics have been formed. Moreover, 11 pieces of potentially influential literature have been mined.

**Conclusions:** This study uses bibliometrics to systematically sort out the development trend, research hotspot and potentially influential literature in the field of medical humanities research since 1974, so as to provides relevant theoretical references and references for research in medical humanities and even medical humanities education.

### Acknowledgements

This work was supported by a project grant from National Social Science Fundation: Research on China’s Experience and Global Sharing in Building a Human Health Community (Grant No. 20&ZD122); Teaching reform Project of Guangdong Medical University “Research on Humanistic Quality Training Path of Medical Students in the Vision of Collaborative Education” (Grant No.1JG21083); Guangdong Provincial University characteristic innovation project “Construction of Humanistic Medicine from the Perspective of Life Culture” (Grant No.2021WTSCX034).

## LHMM23187 EXPLORATION OF PSYCHOLOGICALLY THERAPEUTIC SERVICES FOR VISITORS TO SMART MUSEUM EXHIBITIONS

Xiao Zhang^a^, Deling Yang^a^, Feng Feng^a^, Jianhua Yang^b^, Xijun Huang^a^, Shiqi Chen^a^

*^a^ Guangzhou Academy of Fine Arts, Guangzhou, China, ^b^ Qingdao University of Science and Technology, Qingdao, China.*

**Background:** To explore the main technical applications for increasing the effectiveness of smart museum exhibitions in providing psychologically therapeutic services for visitors in the aftermath of the COVID-19 pandemic, with the goal of investigating the development of museum services for psychological treatment of visitors.

**Subjects and Methods:** From the perspectives of art therapy and cognitive psychology theory, this paper provides a literature review of relevant papers and monographs on social psychological therapy in smart museums. Focusing on museums’ psychological therapeutic services, the analysis examines trends in the development of smart museum exhibition technologies for visitors’ psychological therapeutic services.

**Results:** The psychological problems caused by the pandemic are similar to those of post-traumatic stress disorder (PTSD), making museums an ideal platform for artistic experiences and psychological healing. First, museums provide visitors with an experience environment that reflects a beautiful society and life, helping them to recover from negative emotions and addressing issues related to intrusive memory affecting psychological health. Second, museums offer creative exhibition activities to enhance social communication, helping visitors overcome fear and anxiety, while cultivating confidence and hope for a better future, addressing psychological health problems related to excessive pessimism leading to a loss of willpower. Third, museums provide an enjoyable experience that helps to heal the soul, addressing psychological health problems related to social anxiety.

**Conclusions:** Firstly, smart museums can provide more open and inclusive services to the public by integrating online and offline exhibitions through the use of virtual reality and mobile communication technologies. By breaking down social barriers and alleviating post-pandemic social anxiety, smart museums can attract visitors to return to the museum environment. Secondly, by closely integrating artificial intelligence, the Internet of Things and collection resources in the planning of museum exhibitions, smart museums can create distinctive features that provide more targeted healing solutions for diverse groups of people with various psychological disorders. Thirdly, smart museums can explore scientific methods for treating psychological problems through exhibitions by leveraging big data, cloud computing and collaborating with relevant institutions, thereby making the focus on visitors’ psychological health an important service content of museums. This study aims to contribute new technological applications to the development and construction of enhancing visitors’ psychological health in museum exhibitions.

### Acknowledgement

This research partially funded by Ministry of Education of China under the Humanities and Social Sciences with the project “Research on the normal form of personalized interpretation of smart museum exhibition” (project number 21YJA760093). The research is partially supported under and the 2021 Guangdong province graduate education innovation plan with the project “Guangdong joint postgraduate training demonstration base” (project number: 6040121010). Thanks should go to Ministry of Culture and Tourism of China under the Art Research Key Project Support Plan of 2022 Social Science Fund with the project “Comparative Study of Chinese, Japanese and Hollywood Animation Film Aesthetic and Cross-Cultural Communication Strategy” (project number 22AC003). Special thanks to Guangzhou Academy of Fine Arts for the support of project on Film and Television Special Effects (project number: 6040321054), Science and Technology Art Experimental Class (project number: 6040321002), Research on the Teaching Method of Cultivating Graduate Students’ Cross Media Art Creation Practice (project number: 6040122012LHKY), Research on the Design Method of Digital Exhibition in Smart Museums Based on Technology and Art (project number: 23XSC32).

## LHMM23188 EMPIRICAL STUDY ON THE DIFFERENTIATION OF FOREST FARMERS AND ITS IMPACT ON PSYCHOLOGICAL WELL-BEING UNDER THE LIVELIHOOD CAPITAL FRAMEWORK OF DFID

Xuan Song^a^, JinQi Song^a^

*^a^ Central South University of Forestry and Technology of Business, Changsha 410000, Hunan, China.*

**Background:** Based on the sustainable livelihood analysis framework, this paper conducts in-depth research on the impact of livelihood capital and mental health on the differentiation of farmers in mountainous areas. This is important as industrial differentiation provides a reference for promoting multi-channel income generation for farmers and achieving rural revitalization in mountainous areas.

**Subjects and Methods:** A comparative analysis and multivalue selection model were used to explore the occupational differentiation of 180 farmers in mountainous areas of Hunan Province under different influencing factors.

**Results:** The research results indicate that social capital differentiates pure and non-agricultural farmers into part-time farmers. Rich natural capital can attract farmers to differentiate into pure farmers or part-time farmer enterprises. Moreover, increased financial capital will help part-time farmers focus on agricultural production and encourage non-agricultural operators to engage in agricultural operations. The impact of physical capital will transform farmers into pure or non-agricultural full-time farmers. However, farmers with work experience are likelier to choose part-time agricultural forms. Providing agricultural technology training to farmers can help them immerse themselves in agricultural production by improving their production and management abilities. Additionally, mental health positively impacts the differentiation of pure farmers into part-time farmers and the livelihood capital differentiating the farmers depending on the proximity to the central market.

**Conclusions:** The following suggestions are proposed. Increase psychological health training, develop farmers’ enthusiasm, implement “endogenous” and “exogenous” industrial assistance, and ensure employment. Additionally, attention should be paid to optimizing and combining various livelihood capital and increasing immigrant livelihoods’ sustainable endogenous driving force. Moreover, increase opportunities for immigrants to engage in non-agricultural businesses and encourage the development of ethnic minority characteristic tourism industries and online operation of “characteristic products”. While increasing support for non-agricultural skill training, it is necessary to assist the psychological health construction of migrant workers’ families mainly engaged in agricultural production. Land asset transfer and appreciation work for contracted land for relocation, mountainous forest land, and homestead land are also important. Finally, restoring social networks, cultural protection, and development for ethnic minority immigrants should be considered.

## LHMM23189 CONSTRUCTION AND DISCUSSION OF CARBON AUDIT EVALUATION SYSTEM UNDER CARBON PEAKING AND CARBON NEUTRALITY TARGETS: BASED ON A PSYCHOLOGICAL HEALTH PERSPECTIVE

Changping Yin^a^, Li Zhong^a^, Qinghong Hou^a^, Yunning Tan^b^

*^a^ School of Accounting, Chongqing University of Technology, Chongqing, China, ^b^ School of Accounting, Chongqing Technology and Business University, Chongqing, China.*

**Background:** “Mitigating and adapting to global warming and reducing carbon dioxide emissions” has become the political consensus of all countries in the world, and is also a common challenge for the world. Greenhouse gas emissions, such as carbon dioxide, not only affect political, economic and cultural development, but also have a significant negative impact on the psychological health and happiness of the general public. In order to reduce carbon emissions, the countries of the world have signed and introduced the Kyoto Protocol, the Paris Agreement, the Glasgow Climate Convention and other international conventions. In September 2020, China proposed at the 75th session of the United Nations General Assembly that “China should strive to reach peak carbon dioxide emissions by 2030 and achieve carbon neutrality by 2060” (hereinafter referred to as “carbon peaking and carbon neutrality” target).

**Subjects and Methods:** On February 28, 2022, the United Nations Intergovernmental Panel on Climate Change (IPCC) issued a bulletin stating that greenhouse gas emissions such as carbon dioxide can lead to a series of psychological health problems. In order to better reduce carbon emissions, this paper constructs a carbon audit index system under the carbon peaking and carbon neutrality target based on the hierarchical analysis method with the perspective of the audit and the carbon peaking and carbon neutrality target as the guide.

**Results:** This paper constructs a carbon audit index system based on the main line of “carbon emission policy formulation and implementation → carbon emission reduction governance investment → carbon emission project management → carbon emission governance output → carbon emission management effect”, in an attempt to comprehensively, systematically and scientifically evaluate and authenticate the carbon reduction and emission reduction behaviors of each region and unit, and suppress the negative impact of carbon emission on people’s psychological health.

**Conclusions:** Carbon emission is a global issue, closely related to human economic and social development and physical and psychological health. As the “inspector” of policy implementation and the main national governance system, we should give full play to the role of audit in promoting the “double carbon” work, strengthen the audit supervision of carbon emission policies, projects, funds and benefits, and help achieve the national carbon peaking and carbon neutrality goals.

### Acknowledgements

This work was supported by a project grant from China Postdoctoral Science Foundation (Grant No. 2020M683255), and the National Social Science Fund Item (No. 22BJY033), and the Humanities and Social Sciences Project of the Ministry of Education (No. 19YJC630205).

## LHMM23190 PSYCHOLOGICAL THERAPY RESEARCH OF ART HEALING IN IMMERSIVE GARDEN EXHIBITION

Xiao Zhang^a^, Deling Yang^a^, Feng Feng^a^, Jianhua Yang^b^, Shiqi Chen^a^, Xijun Huang^a^

*^a^ Guangzhou Academy of Fine Arts, Guangzhou, Guangdong Province, People’s Republic of China, ^b^ Qingdao University of Science and Technology, Qingdao, Shandong Province, People’s Republic of China.*

**Background:** Contemporary social work and life stress bring about psychological issues such as depression and anxiety. However, due to limited medical resources, it is necessary to explore the use of art healing as a means to provide psychological therapy services to the general public.

**Subjects and Methods:** This study explores the application of traditional immersive garden exhibition art healing as a method of psychological therapy through natural scenery and humanistic art therapy for the general public. Specific research includes the ways in which exhibitions soothe the emotions of the general public, the use of immersive experiential innovative art healing in exhibitions, and the integration of individuals undergoing art healing into society through exhibitions. Through the analysis of psychological therapy cases and qualitative analysis, taking the “Three Mountains and Five Gardens Exhibition” as an example, the application of art healing in exhibitions for psychological therapy is analyzed and studied from the aspects of communicating audience emotions, allowing audiences to immerse themselves in the art field, and creating a panoramic discourse space for artistic symbols to project new emotions from the audience.

**Results:** First, the “Three Mountains and Five Gardens Exhibition” constructs artistic discourse and achieves emotional resonance and psychological comfort for the audience. Second, virtual reality immersive exhibitions encourage audience participation and artistic experience, achieving emotional healing. Third, the exhibition integrates artistic symbols, recreates cultural symbol representations through various media, and forms a panoramic discourse space constituted by artistic symbols, projecting the audience’s emotions into a new cultural imaginary space for psychological healing.

**Conclusions:** First, art healing fulfills the social responsibility of exhibition updates, reflects the exhibition’s deeper care for the public, and extends the psychological therapy services of traditional immersive garden exhibitions. Second, exhibitions innovate the psychological therapy methods of art healing. Through the panoramic discourse space created by the exhibition’s artistic symbols, the audience can project new emotions and achieve physical and mental healing. Third, the healed audience can better express their thoughts and release emotions through exhibitions to enhance communication. Therefore, the exhibition of innovative art therapy can serve the mental health of the public in a new way.

### Acknowledgement

This research partially funded by Ministry of Education of China under the Humanities and Social Sciences with the project “Research on the normal form of personalized interpretation of smart museum exhibition” (project number 21YJA760093). The research is partially supported under and the 2021 Guangdong province graduate education innovation plan with the project “Guangdong joint postgraduate training demonstration base” (project number: 6040121010). Thanks should go to Ministry of Culture and Tourism of China under the Art Research Key Project Support Plan of 2022 Social Science Fund with the project “Comparative Study of Chinese, Japanese and Hollywood Animation Film Aesthetic and Cross-Cultural Communication Strategy” (project number 22AC003). Special thanks to Guangzhou Academy of Fine Arts for the support of project on Film and Television Special Effects (project number: 6040321054), Science and Technology Art Experimental Class (project number: 6040321002), Research on the Teaching Method of Cultivating Graduate Students’ Cross Media Art Creation Practice (project number: 6040122012LHKY), Research on the Design Method of Digital Exhibition in Smart Museums Based on Technology and Art (project number: 23XSC32).

## LHMM23191 THE RELATIONSHIP BETWEEN THE USE OF SMART DEVICES AND HEALTH MANAGEMENT OF OLDER ADULTS - A THEORY BASED ON A PLANNED BEHAVIOR PERSPECTIVE

Longchao Xu^a^, Menghua Liu^b^, Qian Du^a^, Xianglei Zhu^c^

*^a^ Department of Social policy & Social work, School of Humanities, Taiwan National Chi Nan University, Taiwan, China, ^b^ Graduate School, University of Perpetual Help System DALTA, Las Piñas City, Philippines, ^c^ Department of Health Sociology, School of Humanities and Management, Guangdong Medical University, Dongguan, China.*

LX and ML contributed equally to this study.

**Background:** In recent years, the widespread use of smart devices for the elderly has also triggered more and more attention to how the elderly use smart devices and the importance of smart devices for the health management of the elderly. To this end, this study will investigate the relationship between the use of smart devices and the health management of the elderly to provide a scientific reference basis for optimizing the use of smart devices and implementing health management in the elderly.

**Subjects and Methods:** This paper mainly uses the ordered Probit model to study the effects of smart device use on physical health and mental health of older adults. This paper examines the effects of attitudes, subjective norms, and perceived behavioral control on the physical and mental health of older adults based on planned behavior theory using CGSS 2017 data on older adults aged 60 years and older as a research sample, and analyzes the effects of educational attainment on the relationship between them.

**Results:** It was found that more positive attitudes towards smart device use were associated with higher levels of physical and mental health. However, the moderating effect of educational attainment on the relationship between smart device use and physical and mental health was not significant. In addition, this paper makes the following recommendations: (1) communities increase education and training on the application of smart device for older adults. (2) Family as the smallest unit of support system, with intergenerational interaction to promote digital feedback, can effectively drive the digital integration of older adults.

**Conclusions:** This study found that in China, older adults have more positive attitudes toward smart device use. Moreover, the more positive the attitude toward smart device use, the higher the level of physical and mental health. However, the moderating effect of education level on the relationship between smart device use and physical and mental health was not significant, but the regression coefficient was negative. This paper to provide more practically meaningful guidance and suggestions for the use of smart devices in the elderly, and to provide better support and assistance for health management and smart device use in the elderly.

### Acknowledgements

This article is the research output of the introduced talent scientific research launch fund project of Guangdong Medical University--“Application and improvement research of innovative technology of social work service for the Elderly”.

## LHMM23192 THE INFLUENCE OF RURAL INDUSTRIAL INNOVATION AND SYMBIOTIC DEVELOPMENT ON ALLEVIATING THE PSYCHOLOGICAL ANXIETY OF RURAL RESIDENTS

Jianping Liu^a,b^, Yongtian Wu^b^, Peitao Yang^c^, Xuekai Wei^b^

*^a^ College of Civil and Environmental Engineering, Hunan University of Science and Engineering, Yongzhou, Hunan 425199, China, ^b^ Bama Hetai Longevity Industry Co., Ltd., Bama, Guangxi 547500, China, ^c^ College of Economics, Central South University of Forestry and Technology, Changsha, Hunan 410004, China.*

**Background:** The psychological recognition level of rural residents is the basis and prerequisite for choosing and promoting innovative development of rural industries. However, for a long time, the relatively weak foundation of rural industrial development, insufficient competitive advantages in industries, and relatively low output return rates have led to deep doubts among rural residents about whether they can promote innovative development of rural industries. It is urgent to alleviate the psychological anxiety of rural residents in both theoretical and psychological aspects, this has important theoretical and practical significance in promoting innovation and sustainable development of rural industries, achieving coordinated development between urban and rural areas and regions. However, there has been no research exploring the correlation and impact of rural industrial development and residents’ psychological interaction from the perspective of psychological health and symbiosis theory.

**Subjects and Methods:** Using the research method of symbiosis theory, an in-depth analysis and summary of the common anxiety problems in villagers’ psychology were conducted from the perspective of mental health management. A comprehensive and systematic study in Some aspects of the construction and improvement of villagers’ psychological health, improvement of psychological cognition level, composition of rural industrial symbiosis system, symbiotic connection mechanism, symbiotic interface, symbiotic mode, symbiotic ecosystem and other aspects has been researched on the interactive mechanism between the psychological logic and motivation of villagers’ psychological anxiety and the innovative development of rural industries.

**Results:** The research results indicate that villagers’ psychological anxiety is the fundamental obstacle that restricts the innovative development of rural industries. The level of villagers’ psychological health is the fundamental prerequisite for determining and influencing the innovative development of rural industries. Insufficient or biased psychological cognition of villagers is the primary obstacle that hinders the development of rural industries, and the lag in development awareness and thinking is the internal reason that hinders the development of rural industries, The insufficient level and depth of integration are the main practical problems faced by the development of rural industries, and the weak linkage mechanism is the fundamental reason that restricts the development of rural industries.

**Conclusions:** There is an urgent need to build a sustainable development path for rural industries in some aspects of alleviating villagers’ psychological anxiety, improving their mental health level, improving their psychological cognition level, strengthening the concept of innovative symbiotic development, constructing a connecting carrier platform, constructing efficient symbiotic mode, smoothing symbiotic interfaces, creating a positive symbiotic environment, constructing symbiotic connection mechanism, and strengthening co-effectiveness, in order to create a symbiotic ecosystem for innovative development of rural industries, To provide theoretical and practical references for promoting the sustainable development of rural industries.

### Acknowledgments

This work was supported by a project grant from Natural Science Foundation of Hunan Province(Grant No.2019JJ40545) and Hunan University of Science and Technology Applied Characteristic Discipline Construction Project(Grant No.18TSXK-001).

## LHMM23193 A STUDY ON STRATEGIES TO IMPROVE THE MENTAL HEALTH OF FARMERS FROM THE PERSPECTIVE OF METAVERSE

Chunyi Cao^a^, Shanshan Niu^a^, Yijian Wang^a^

*^a^ Zhejiang Institute of Economics and Trade, Zhejiang Province, Hangzhou, China.*

**Background:** To achieve rural revitalization more quickly and effectively, it is necessary to enhance the quality of the vast peasantry, as farmers are an important human resource for rural revitalization. However, there are still issues such as low mental health level of farmers and long-term neglect of farmers’ psychological problems. Therefore, it is imperative to conduct research on improving the mental health level of farmers in the context of rural revitalization.

**Methods:** Based on Maslow’s Hierarchy of Needs theory, research and analyses of farmers’ actual needs can be conducted. With the support of metaverse technologies, artificial intelligence and virtual reality technologies, an all-in-one agricultural assistance platform can be built to provide innovative digital content applications for farmers’ production, sales, and livelihood.

**Results:** Based on the agricultural assistance platform, 3D virtual sports interaction with fun elements can be utilized to address the issues of inadequate sports facilities and farmers’ weak awareness of fitness, thereby improving farmers’ physical fitness and meeting their physiological needs. Through online and offline e-commerce skills training and the agricultural assistance platform, the problem of farmers lacking e-commerce skills and sales channels can be solved, thus fulfilling farmers’ safety needs. By leveraging metaverse technologies to allow users to understand the original production process of products, communication between farmers and consumers can be promoted, satisfying farmers’ social needs. Through big data and AI technologies, brand planning and design for agricultural products can be carried out, thus meeting farmers’ self-actualization needs and promoting the holistic development of rural residents’ physical and mental health.

**Conclusions:** As a research hotspot in the digital economy, the integration and development of metaverse with the agricultural field holds great potential. Particularly, the innovative approach of incorporating metaverse into farmers’ mental health in this article is noteworthy. Improving the overall mental well-being of rural areas is crucial for ensuring sustainable development in rural areas, and there is still a long way to go.

## LHMM23194 EFFECT OF HEAT TREATMENT TEMPERATURE ON THE SYNTHESIS AND PROPERTIES OF NALA4(SIO4)3F: EU3+ RED PHOSPHORS SUITABLE FOR MEDICAL TESTING

Yanhua Zhou^a^, Weifeng Xie^b^, Guo Feng^b^, Pengpeng Yu^b^, Xianhui Duan^a^

*^a^ College of Urban Construction, Jiangxi University of Technology, Nanchang 330098, China, ^b^ Department of Material Science and Engineering, Jingdezhen Ceramic Institute, Jingdezhen 333000, China.*

*YZ and WX contribute equally to this work.*

**Background:** The rapid development of phosphor application materials and technology provides a very important platform and means for the research and development of medical detection, and the key to effective control and treatment of diseases is that accurate detection can be carried out at an early stage, so the development of new, stable, simple preparation conditions, practical value of red phosphor is a very meaningful work. Near-infrared light has important applications in security monitoring, biomedicine, plant lighting, spectral detection and other fields. Traditional near infrared light source cannot meet people’s demand for fast and convenient use of near infrared equipment. Near-infrared phosphor conversion LED can make up for the deficiency of the traditional near-infrared light source, and it is the most promising new near-infrared light source to replace the traditional near-infrared light source.

**Subjects and Methods:** Red phosphor has excellent optical properties, can emit red fluorescence, has the advantages of high stability and high efficiency, and can be used for biomedical detection. NaLa4(SiO4)3F: Eu3+ red phosphor was prepared by solid-phase method, and the influence of preparative process parameters on performance was systematically studied.

**Results:** The study found that: the minimum temperature for the synthesis of NaLa4(SiO4)3F phase by solid-phase method was 750℃, the minimum temperature for the preparation of NaLa4(SiO4)3F pure phase was 850℃, and the optimal heat treatment temperature is 950℃. The optimal dosage of sodium fluoride (multiple of relative stoichiometric ratio) x = 1.3. The use of heat treatment and heterogeneous methods to strengthen the conversion of fluorescent materials in disease and biomedical detection has great significance and considerable application prospects. It is believed that the thermal stability regulation and design of phosphor will become an important research hotspot of phosphor research, will be widely concerned by researchers, and will become a kind of very potential phosphor matrix materials.

**Conclusions:** The phosphor particles prepared by the solid phase method are spherical-shaped, with clear edges and surfaces, narrow particle size distribution, and are suitable for LED packaging. NaLa4(SiO4)3F: Eu3+ red phosphor will become an important tool to assist related medical testing, and is also widely used in portable and real-time related testing, providing important support and guarantee for medical testing.

### Acknowledgements

This work was supported by the Higher Education Teaching Reform Project of Jiangxi Provinc China (JXJG-22-24-5), the National Natural Science Foundation of China [grant numbers 52072162, 51962014], Jiangxi Provincial Natural Science Foundation [grant numbers 20202ACBL214008, 2020ZDI03004, 20202BABL214013], Open fund project of the Key Laboratory of Inorganic Coating Materials, Chinese Academy of Science (ICM-202001), the Projects of Jiangxi Provincial Department of Science and Technology (20202BABL214013), the Science Foundation of Jiangxi Provincial Department of Education (GJJ201324, GJJ201345).

## LHMM23195 A STUDY ON AUDIENCE IN THE COMMUNICATION OF URBAN IMAGE FROM THE PERSPECTIVE OF MENTAL HEALTH

Shaoyun Yang^a^

*^a^ Business Administration School, Zhuhai Technology Colleg, Zhuhai City 516000, China.*

**Background:** In recent years, with the acceleration of urbanization and economic development, the sense of happiness and belonging brought by cities has not been satisfactory. Cities gather population and various resources, but at the same time, the oppression and decadence caused by living issues such as wages, childbirth, education, transportation, and prices are becoming increasingly severe. Citizens’ impression of the city has changed from a warm home to ubiquitous work pressure. This phenomenon constantly reminds us that there have been social and psychological problems in China’s urbanization process. A series of popular communication trends such as “fleeing Beijing, Shanghai, and Guangzhou” have shown us that the main body of urban image shaping should be understanding the happiness of life, reflecting more on the psychological health and spiritual satisfaction of citizens. Nowadays, people have created increasingly diverse forms of communication through the communication platforms provided by modern technology, and their recognition of regional culture is becoming increasingly strong. The increasing pressure and anxiety in urban life are psychological issues in the development of Chinese cities, but these issues also provide soil for the construction and development of urban culture and image.

**Subjects and Methods:** Urban image refers to various perceptual factors presented by a city in the public’s mind, which can be the influence of various aspects such as architecture, culture, history, environment, cultural activities, etc., as well as people’s understanding and interpretation of the symbols and meanings generated by these factors.

Audience psychology refers to the thinking, emotions, and behavioral reactions that occur during the process of receiving and processing information. In communication activities, understanding the characteristics and changing trends of audience psychology can effectively formulate communication strategies and creativity, thereby improving communication effectiveness.

Literature research and data analysis methods are used in this article. In the current era of information explosion, all kinds of communication media are using different communication strategies to attract the attention of the audience. In fact, they are trying to break through the audience’s psychological system: make appropriate information according to the criteria of the audience’s psychological selection, so that the information transmitted can enter the audience’s psychological system faster and better through the psychological selection of the audience. Therefore, the psychological selection process of the audience has become the “invisible axis” of various media production content today.

Understanding the psychological characteristics of the audience is the foundation for developing effective communication strategies. Communicators need to deeply understand the characteristics and needs of the audience, design and optimize communication content and methods based on the audience’s psychology, in order to maximize the effectiveness of communication.

**Results:** The attractiveness of a city’s image to its audience is not only an external manifestation of its comprehensive strength, but also a symbol of its soft power. People come with “dreams” and strive for their dreams in the city. However, when the development of the city encounters bottlenecks or lags behind, people’s dreams will also be shattered, leading to a series of social psychological effects such as weakened individual psychological feedback and increased social exclusion. When regional economic development lags behind, people tend to focus more on material interests rather than emotional needs, but at the same time, the motivation to participate in social or economic activities decreases, entering a vicious cycle. Due to the motivating effect of urban image internally and the attractive effect externally, the dissemination of urban image will expand the space for urban development: the combination of the creativity of foreign residents and new spaces provides vitality for urban development, while also providing a path to realize dreams and spiritual support for citizens. Urban popularity is an important indicator of urban development. Through urban image promotion, more people can understand the city, generate interest and favoritism towards the city, and attract more people to travel, study, work, and live in the city.

**Conclusion:** In recent years, with the acceleration of urbanization and economic development, the sense of happiness and belonging brought by cities has not been satisfactory. Cities gather population and various resources, but at the same time, the oppression and decadence caused by living issues such as wages, childbirth, education, transportation, and prices are becoming increasingly severe. Citizens’ impression of the city has changed from a warm home to ubiquitous work pressure. This phenomenon constantly reminds us that there have been social and psychological problems in China’s urbanization process. A series of popular communication trends such as “fleeing Beijing, Shanghai, and Guangzhou” have shown us that the main body of urban image shaping should be understanding the happiness of life, reflecting more on the psychological health and spiritual satisfaction of citizens. Nowadays, people have created increasingly diverse forms of communication through the communication platforms provided by modern technology, and their recognition of regional culture is becoming increasingly strong. The increasing pressure and anxiety in urban life are psychological issues in the development of Chinese cities, but these issues also provide soil for the construction and development of urban culture and image.

## LHMM23196 M6A MODIFICATION LNCRNA FOXP4-AS1 CONTRIBUTE TO OVARIAN CANCER DEVELOPMENT AND PROGNOSIS BY REGULATING TME

Zhanghui Li^a^, Yuxing Shang^a^, Qiaoyi Lv^a^, Yangjing Zhao^a^, Hui Wang^a^, Weili Cai^b^, Hui Zhang^c^,Yanling Zhu^d^, Xiuting Liang^d^, Qixiang Shao^a,b^

*^a^ Reproductive Sciences Institute, Jiangsu Key Laboratory of Medical Science and Laboratory Medicine, Department of Immunology, School of Medicine, Jiangsu University, Zhenjiang 212013, Jiangsu, P. R. China, ^b^ Institute of Medical Genetics and Reproductive Immunity, School of Medical Science and Laboratory Medicine, Jiangsu College of Nursing, Huai’an 223002, Jiangsu, P. R. China, ^c^ Department of Clinical Laboratory, The Affiliated Hospital of Jiangsu University, Zhenjiang 212001, Jiangsu, P. R. China, ^d^ Department of Gynaecology and Obstetrics, The Xuzhou Hospital Affiliated to Jiangsu University, Xuzhou 221005, Jiangsu, P. R. China.*

**Background:** Ovarian cancer (OC) is one of the three malignant tumors with the highest mortality rate in the female reproductive system. Owing to the special anatomic location and the lack of typical clinical symptoms, the early onset is insidious. Due to drug resistance and easy recurrence, the 5-year survival rate of ovarian cancer remains has not improved significantly in the past thirty years, and mortality rate is also at a very high level, despite the great progress in chemotherapy, surgery and immune therapy. However, the mechanism of oncogenesis, progression, recurrence, and drug resistance of ovarian cancer have not been fully elucidated. Recently, it has been reported that N6-methyladenosine (m6A) and long non-coding RNAs (lncRNAs) play pivotal roles in ovarian cancer initiation, progression, drug resistance and recurrence. Whereas, the regulatory role and its mechanisms of m6A modification on lncRNAs in ovarian cancer are still unclear. Therefore, it is important to elucidate the regulatory role of m6A modification of lncRNAs and its effects on the development and prognosis of ovarian cancer.

**Subjects and Methods:** We comprehensively evaluated the m6A modification patterns of 29 m6A modification-related genes in 374 samples from the TCGA ovarian cancer database, constructed an independent prognostic model based on m6A modification-related lncRNAs. ROC analysis was performed to verify the reliability of the prognostic model based on the age, stage and type of the patients. The data of the patients were divided into high-risk group and low risk group. TMB analysis, GO and KEGG pathway analysis of immune cells, immune infiltrate analysis and drug sensitivity analysis were performed in the prognostic model. QRT-PCR was used to detect the expression levels of 5 m6A modified lncRNAs in ovarian cancer tissues, and the changes of immune checkpoints in peripheral blood of ovarian cancer patients were analysed by flow cytometry.

**Results:** The prognosis model that we constructed was reliable, through the prognosis model, we found that the prognostic characteristics of patients with ovarian cancer are related to the prognostic model, Naive B cell was necessary and played an important role in ovarian cancer, TIGIT had high value in immune checkpoint analysis in prognostic models, and Pazopanib might be a targeted medicine for ovarian cancer. In tumor tissues and peripheral blood of patients, we further verified that FOXP4-AS1 played a m6A dependent role in ovarian cancer and TIGIT was abnormally elevated in the tumor microenvironment of patients with ovarian cancer.

**Conclusions:** The results demonstrated that m6A modification-related lncRNAs participated in malignant progression, recurrence, and drug resistance of ovarian cancer by regulating the tumor immune microenvironment. FOXP4-AS1 may will serve as a potential biomarker for ovarian cancer.

### Acknowledgments

This work was supported by the National Natural Science Foundation of China (82071738). Institutional Review Board Statement: The study was conducted in accordance with the guidelines of the Declaration of Helsinki, and was approved by the Ethics Committee of the Affiliated Hospital of Jiangsu University (KY2022K0913) and the Ethics Committee of Xuzhou Hospital Affiliated of Jiangsu University (2019-02-010-K01), respectively.

## LHMM23197 FEASIBILITY STUDY ON ACCOUNTING PSYCHOLOGICAL BARRIERS, PROBLEMATIC BEHAVIOR, AND PSYCHOLOGICAL ADJUSTMENT

Wenlong Zhou^a^, Wei Cui^a^, Lisheng Chen^a^

*^a^ Shandong Youth University of Political Science, Shandong, China.*

**Background:** With the development and progress of the economy and society, accounting work is playing an increasingly important role. Accounting psychological barriers can easily lead to unethical accounting behavior. How to provide psychological adjustment is currently the focus of accounting work. This article explores the relationship between accounting psychological barriers and unethical accounting behavior from the perspective of accounting psychology, focusing on the specific manifestations of accounting psychology and unethical accounting behavior, as well as feasible suggestions for psychological adjustment of the psychological barriers that may arise. This article provides theoretical support and practical guidance for the psychological adjustment of unethical accounting behavior, and summarizes its application experience.

**Subjects and Methods:** This research will primarily use normative analysis, supplemented by qualitative analysis, logic, and other methods. The main focus of this paper is to explore the relationship and influence between accounting psychological barriers, accounting misconduct, and psychological adjustment from the perspective of accounting psychology. Due to the difficulty of obtaining relevant research samples and data from public sources in both behavioral accounting research and accounting psychology research, this paper conducts a review of relevant literature and develops a survey questionnaire. Through theoretical analysis of the generation and change of psychological barriers in accounting subjects, as well as the relationship with accounting misconduct, this paper provides explanations and rationalization suggestions for the psychological adjustment of accounting psychological barriers.

**Results:** This paper expounds on the historical development and current situation of accounting, analyzes the content, composition, and influencing factors of accounting psychology, explains the reasons and influencing factors of accounting psychological barriers and accounting misconduct. The relationship between accounting misconduct and accounting psychological barriers is discussed, and the process of accounting misconduct is emphasized. By referencing literature, questionnaire surveys, and other materials, this paper reveals the internal relationship between the subjective and objective environment and personal characteristics of accounting practitioners and the factors that contribute to the occurrence of psychological barriers and accounting misconduct. Finally, from the perspective of how to adjust psychological barriers, suggestions are made to standardize accounting behavior.

**Conclusions:** The psychological barriers of accounting subjects engaged in accounting work are the key factors that lead to accounting misconduct. The two are closely related and influence each other. The psychological barriers of accounting subjects are the main reasons that affect the occurrence of accounting misconduct, while accounting misconduct is the manifestation of accounting psychological barriers. By studying the relationship between accounting psychological barriers and accounting misconduct, exploring the reasons for the emergence of accounting psychological barriers, and implementing reasonable and effective psychological adjustment measures, can we fundamentally find methods and strategies to standardize accounting behavior.

### Acknowledgments

Shandong Province Education Science “the thirteenth Five-Year Plan” project: research on the construction of “big accounting” professional practice teaching system in the era of digital intelligence (project number: 2020YB005).

## LHMM23198 EFFECT OF CHILDHOOD MALTREATMENT ON SOCIAL ANXIETY AMONG HEARING-IMPAIRED STUDENTS: THE MEDIATING ROLE OF REGULATORY EMOTIONAL SELF-EFFICACY

Leyi Shao^a^, Hongyu Lan^a^, Xinyu Lao^a^, Guangning Luo^b^, Xiaolan Ma^a^

*^a^ School of Education Science, Nanning Normal University, Nanning, China, ^b^ Nanning School for the Special Education, Nanning, China.*

**Background:** Early childhood experience has a profound impact on an individual’s lifelong development, and the quality of development is influenced by many factors of biological and social environment, among which the traumatic experience of children in early childhood is closely related to their later psychological problems and anxiety disorders, traumatic experiences mainly include physical abuse, physical neglect, emotional abuse, emotional neglect, sexual abuse, etc. Social anxiety disorder will directly affect social skills, which is not conducive to the development of individuals’ social functions and the construction of good interpersonal relationships. Compared with normal students, Hearing-impaired students tend to perceive certain prejudice and hostility, so they have a strong defensive psychology and often exaggerate such prejudice. This hostility and distrust towards others can have a negative impact on the establishment of their interpersonal relationships, thus making them prone to anxiety when communicating with others. So they are more prone to emotional problems such as social avoidance and social anxiety. Emotional regulation self-efficacy, as a kind of self-efficacy with emotional regulation characteristics, actually refers to a sense of confidence formed by individuals based on their subjective evaluation of their own emotional regulation ability, which is a key factor to reduce social anxiety in adolescents. Under the dynamic effect of emotional regulation self-efficacy, individuals can take effective emotional regulation strategies and properly deal with emotional events, especially negative emotional events. Therefore, it is of great significance to explore the mechanism of emotional regulation self-efficacy between childhood maltreatment and social anxiety.

**Subjects and Methods:** To investigate the influence mechanism of childhood maltreatment on social anxiety of hearing-impaired students, 135 hearing-impaired students were investigated by the Childhood Maltreatment Questionnaire (CTQ-SF), the Emotional Regulation Self-efficacy Scale (SR-ESE), and the Social Interaction Anxiety Scale (SIAS).

**Results:** The results indicated that: (1) childhood maltreatment significantly positively predicted social anxiety; (2) childhood maltreatment and emotional regulation self-efficacy showed a significant negative correlation, and there was a significant positive correlation between emotional regulation self-efficacy and social anxiety; (3) emotional regulation self-efficacy played a mediating role between childhood maltreatment and social anxiety.

**Conclusions:** It suggested that childhood maltreatment may have a long-term negative influence on hearing-impaired students’ social anxiety, which is partially mediated through emotional regulation self-efficacy.

### Acknowledgments

This study is the phased research achievement of 2023 Guangxi Education Science “14th Five-Year Plan” Topic: Mental Health Status and Service System Construction of Sound healthy siblings in Families with Special Children, Project No.: 2023B145.

## LHMM23199 NETWORK PHARMACOLOGICAL MECHANISM OF MONGOLIAN MEDICINE NUAN GONG QI WEI PILL IN TREATING DIABETIC KIDNEY DISEASE

Guizhi^a^, Yuanyuan Ma^a,b^, Zhiqiang Bao^c^, Narenchaoketu^a^, Huan Wang^d^, Hua Li^a^, Huhemuren^a^, Shui Ling^a^, Siqin^a^, Naqin^a^, Yingsong Chen^a,d^

*^a^ Inner Mongolia Medical University Inner Mongolia, Hohhot 010010, China, ^b^ Affiliated Hospital of Inner Mongolia Medical University, Inner Mongolia, Hohhot 010010, China, ^c^ Inner Mongolia Hospital of International Mongolian Medicine, Inner Mongolia, Hohhot 010065, China, ^d^ Inner Mongolia Minzu University, Tongliao 028000, China.*

Guizhi and YY Ma contributed equally to this study.

**Background:** This research aims to examine the molecular mechanism of Mongolian medicine Nuan Gong Qi Wei Pill (NGQW) in treating diabetic kidney disease (DKD) based on network pharmacology and molecular docking and analyze the molecular regulatory mechanisms of its major active ingredients docking with the significant targets of DKD.

**Subjects and Methods:** The active ingredients and their targets of NGQW were acquired from the Traditional Chinese Medicine Systems Pharmacology Database and Analysis Platform (TCMSP), Encyclopedia of Traditional Chinese Medicine (ETCM), and a high-throughput experiment- and reference-guided database of traditional Chinese medicine (HERB). DKD-related targets were founded by searching four databases: DisGeNET, Gene Cards Human Gene Database (GeneCards), Online Mendelian Inheritance in Man (OMIM), and Therapeutic Target Database (TTD). The STRING database was used to construct a protein-protein interaction (PPI) network to search for major targets. Additionally, enrichment analysis involving main biological processes and pathways was carried out using the Database for Annotation, Visualization, and Discovery (DAVID) and Metascape databases. Cytoscape software was used to construct and analyze networks. AutoDock Vina and PyMOL were used to validate molecular docking between significant active ingredients and major key targets.

**Results:** Collectively, 174 NGQW active ingredients, 492 potential targets, and 4227 DKD targets were screened. After the intersection procedure, 295 potential targets of NGQW for DKD were obtained. In addition, quercetin, beta-sitosterol, oleic acid, isoliquiritigenin, and apigenin were the possible key active ingredients of NGQW, Signal transducer and activator of transcription 3 (STAT3), Nuclear factor NF-kappa-B p65 subunit (RELA), Mitogen-activated protein kinase 1 (MAPK1), Heat shock protein 90 alpha family class A member 1 (HSP90AA1), and AKT Serine/Threonine Kinase 1 (AKT1) were important targets in the PPI network. The PI3K/AKT signaling pathway, the lipid and atherosclerosis signaling pathway, the AGE-RAGE signaling pathway in diabetic complications, and the MAPK signaling pathway were all involved in the enrichment analyses along with biological processes including the cellular response to oxidative stress, hormones, and nitrogen compounds. Furthermore, this study highlighted a good binding activity between key active ingredients and key targets.

**Conclusions:** NGQW has the potential to treat DKD through multiple pathways, targets, and compounds. The main active ingredients in this medicine, such as quercetin, isoliquiritigenin, beta-sitosterol, oleic acid, and apigenin interfere with multiple signaling pathways including cellular response to nitrogen compounds, hormones, and oxidative stress by regulating crucial targets such as STAT3, RELA, HSP90AA1, MAPK1, and AKT1.

### Acknowledgments

This work was supported by a project grant from the word-class discipline“ construction project of Traditional Chinese Medicine (Mongolian medicine) of Inner Mongolia Medical University, (Grant No.myxylxk2022017).

## LHMM23200 RESEARCH ON THE IMPACT OF CUSTOMER INVOLVEMENT ON NEW PRODUCT DEVELOPMENT PERFORMANCE: BASED ON INDIVIDUALS’ MENTAL HEALTH PERSPECTIVE

Xiaobo Tao^a^, Chao Fan^b^

*^a^ School of Economics and Management, North China University of Technology, Beijing 100144, China, ^b^ School of Business, Central University of Finance and Economics, Beijing 100098, China.*

**Background:** With the continuous advancement of technology and the ongoing innovation of business models, companies are facing an increasingly competitive business environment. In this context, successful development of new products has become one of the key strategies for companies to cope with competition. In reality, practices led by companies and inviting customers to participate in new product development are becoming quite common, and as a result, many forms of customer involvement have emerged. However, the issue of how to effectively improve the satisfaction and mental health of customers, and encourage them more proactive and engaged in the company’s new product development process, has received little attention. Mental health refers to a person’s overall psychological well-being, including their emotional, cognitive, and social functioning. It encompasses how an individual thinks, feels, and behaves, as well as their ability to cope with life’s challenges and adapt to changes. The promotion and preservation of mental health contributes to individuals’ overall quality of life, productivity, and social integration, and it reduces the burden of mental illness on individuals, families, and society. Therefore, favorable levels of mental health among customers are conducive to enhancing their efficiency in participating in new product development.

This requires companies to choose more appropriate forms of customer involvement in the new product development process, so as to not only improve customers satisfaction and mental health, but also further enhance company performance and put new product development on a healthier and more sustainable track. Based on this, the study will conduct a systematic exploration of how different forms of customer involvement affect new product development performance.

**Subjects and Methods:** This study conducted a questionnaire survey on 287 high-tech companies with independent R&D departments in five provinces and cities including Beijing, Tianjin, Shandong, Shanghai, and Jiangsu. Finally, a set of 267 valid questionnaires was obtained. These questionnaires were mainly distributed across industries such as electronic information technology, biotechnology and new pharmaceutical technology, and aerospace technology.

**Results:** Based on the regression analysis, the results suggested that customer involvement had a significant positive impact on new product development performance. Furthermore, the study revealed that the stronger the incremental innovation capability, the stronger the impact of customer involvement as an information source on new product development performance, while the impact of customer involvement as innovators was weaker. In comparison, the stronger the radical innovation capability, the stronger the impact of customer involvement as innovators on new product development performance, and the weaker the impact of customer involvement as an information source. However, the interaction effect of incremental or radical innovation capability and customer involvement as co-developers was not significant.

**Conclusions:** When a company possess strong incremental innovation capability, customer involvement as an information source is more advantageous for enhancing new product development performance. Conversely, when companies possess strong radical innovation capability, customer involvement as innovators is more conducive to enhancing new product development performance. During this process, the mental health of customers plays a crucial role. The participants’ good mental health level triggered by appropriate customer involvement strategies not only strengthens their ability to cope with stress, challenges, and maintain positive interpersonal relationships at work, but also further enhances their initiative and work efficiency in participating in new product development. Therefore, improving the mental health of participants is of significant importance in enhancing the performance of new product development. The study enriches the understanding of the mechanisms through which customer involvement influences new product development performance and offers empirical evidence for companies with diverse innovation capabilities to determine suitable customer involvement strategies for new product development.

## LHMM23201 THE INFLUENCE OF ADVERSITY QUOTIENT THEORY ON EXERCISE PERSISTENCE AND RELIEF OF STUDENTS’ ANXIETY

Jinggang Li^a^, Lijun Wang^b,c,d^, Yuepeng Sun^e^, Hyoung-Kil Kang^f^

*^a^ Sports Department, Jilin International Studies University, Changchun 130000, Jilin, China, ^b^ College of Physical Education and Health, Yulin Normal University, Yulin 537000, Guangxi, China, ^c^ International College, Krirk University, Bangkok 10220, Thailand, ^d^ Wuchang University of Technology, Wuhan 43000, Hubei, China, ^e^ Sports Department, Dalian Maritime University, Dalian, Liaoning 116026, China, ^f^ Graduate School of Education (Physical Education Major), Kyungnam University, Changwon, Republic of Korea.*

**Background:** This study aims to investigate the impact of Adversity Quotient Theory on the exercise persistence of Chinese college students and to develop an Exercise Adversity Coping Scale to explore the relationship between exercise adversity coping ability and student anxiety alleviation.

**Subjects and Methods:** The present study aimed to develop an Exercise Adversity Coping Scale based on the Adversity Quotient Theory. The scale includes three dimensions and twelve items, and has been shown to have good reliability and validity. In addition, the study sought to validate the effect of the Adversity Quotient Theory on exercise persistence and the alleviation of student anxiety, through exercise behavior surveys and student interviews.

**Results:** Based on data collected from interviews and questionnaires, typical exercise adversities were identified using NVivo12 software. Next, Exploratory Factor Analysis (EFA) was conducted on a sample of 345 Chinese college students to evaluate the psychometric properties of 54 items and develop a 15-item Exercise Adversity Coping Scale with three dimensions: Exercise Adversity Responsibility, Exercise Adversity Control, and Exercise Adversity Influence. This scale explained 63.54% of the cumulative variance, and its internal consistency reliability and split-half reliability were found to be 0.909 and 0.874, respectively. Additionally, Confirmatory Factor Analysis (CFA) was conducted on another sample of 300 college students, and the remaining 12 items were validated. According to the results of exercise behavior surveys, college students who consistently exercised had an average score of 3.63 on the exercise adversity coping ability scale. Moreover, interviews conducted with students who had high exercise adversity coping abilities revealed that they considered physical exercise to be an effective means of relieving stress, which is supported by related literature.

**Conclusions:** The Exercise Adversity Coping Scale developed in this study has demonstrated acceptable reliability and validity. Moreover, the findings suggest that developing exercise adversity coping ability can enhance exercise persistence, which in turn may help alleviate student anxiety.

## LHMM23202 EFFECT OF PROBLEMATIC MOBILE SOCIAL MEDIA USAGE ON DEPRESSIVE SYMPTOMS OF CHINESE COLLEGE STUDENTS

Yan Jiang^a^, Xiang Wang^b^

*^a^ Psychological Counseling Center, Shanghai University, Shanghai, 20444, China, ^b^ Department of Nursing, Zhuhai Campus of Zunyi Medical University, Zhuhai 519041, China.*

**Background:** The lifestyles and mental health of numerous college students were affected by the outbreak of the novel coronavirus disease. However, no study has examined the impact of subjective evaluations of exposure to the pressure on mental health. The current study was conducted and examined the relationship between problematic mobile social media usage and depressive symptoms, and the moderating effect of perception of the novel coronavirus disease.

**Subjects and Methods:** A cross-sectional online questionnaire study of 3123(63.3% female and 36.7% male) Chinese college students in the Shanghai was conducted in March and April 2020. Problematic mobile social media usage was assessed by the Problematic Mobile Social Media Usage Assessment Questionnaire and depressive symptoms were assessed using the Patient Health Questionnaire-9 (PHQ-9). 2056 students reported that they were affected by COVID-19, and 1067 students formed the “unaffected group.” Structural equation modeling (SEM) in Mplus 8.0 was used to examine whether the hypothesized relationship between problematic social network usage and depressive symptoms was affected by whether the respondents reported being affected by COVID-19.

**Results:** The effect of problematic mobile social media usage on depressive symptoms was significant. The level of problematic mobile social media usage and depressive symptoms in the affected group was significantly higher than that in the unaffected group. The interaction between problematic mobile social media usage and perceived influence of COVID-19 was statistically significant. The effect of problematic mobile social media usage on depressive symptoms was moderated by perceived COVID-19 influence. Specifically, compared with students who were unaffected by the novel coronavirus disease, those affected showed more severe depressive symptoms.

**Conclusions:** The findings suggest independent and interactive effects of problematic mobile social media usage. The results highlighted the underlying mechanisms in the relationship between problematic social media usage and depression. These findings provide practical insights into the development and implementation of psychological interventions when facing pressure.

## LHMM23203 AN INVESTIGATION OF THE MODERATING EFFECT OF DIGITAL DIVIDE ON THE RELATIONSHIP BETWEEN FINANCIAL INCLUSION AND MENTAL HEALTH OF POOR RESIDENT

Anzhong Huang^a^, Qiuxiang Bi^b^, Zhouyin Zhang^c^, Luote Dai^d^

*^a^ School of Economics and Management, Nanchang Institute of Science and Technology, Nanchang, 330108, China, ^b^ School of Management, Guangzhou Xinhua University, Dongguan, 523133, China, ^c^ School of Accounting and Finance, Anhui Xinhua University, Hefei, 230088, China, ^d^ School of Digital Economy and Trade, Wenzhou polytechnic, Wenzhou City, 325035, China.*

**Background:** Based on the hypothesis of the positive relationship between poverty and its vulnerability and residents’ mental health, this paper aims to test whether the digital divide affects the efficiency of poverty alleviation in inclusive finance, so as to affect people’s mental health.

**Subjects and Methods:** Poverty is the main acoustic factor of people’s mental health. Whether inclusive finance can improve poverty and finance to alleviate people’s mental health has been debated in academic circles. Based on econometric analysis, this paper holds that the digital divide has a downward effect on the poverty alleviation effect of general finance, which in turn affects people’s mental health.

With the development of digital technology, digital inclusive finance is becoming the mainstream. However, the digital divide significantly affects the inclusiveness of financial development.

Based on the measurement of China’s digital financial inclusion and digital divide, this paper studies the moderating effect of digital divide’s financial inclusion.

**Results:** The test results show that family health, labor, the dependency ratio, per capita income levels, and the influence of the level of cash deposits are significantly negative, family average age for the action of increasing poverty vulnerability, and the influence of family size is may be due to the large family, more than in the case of more than three generations would cohabit stroke young family in pension spending is higher, This burden increases the vulnerability of households to poverty. The development of digital inclusive finance weakens the poverty vulnerability of households on the whole. The existence of digital divide does negatively affect the poverty alleviation effect of digital inclusive finance.

**Conclusions:** The development of digital financial inclusion has a significant negative effect on the vulnerability to poverty and the digital divide significantly weakens the mitigation effect of digital financial inclusion on poverty vulnerability. Through poverty and poverty fragility, the digital divide has a negative impact on residents’ mental health. The digital divide has a huge impact on families’ mental health by affecting financial inclusion. The digital divide has a huge impact on families’ mental health by affecting financial inclusion. So addressing the digital divide can not only improve financial inclusion, but also address the mental health pressures that families face. There is a significant negative correlation between the digital divide and the mental health of the poor resident. Therefore, addressing the digital divide can improve mental health of the poor resident.

### Acknowledgments

This study was supported by the 13th Five-Year plan research project of philosophy and social sciences of Guangdong province (GD17YGL03); the Project of Philosophy and Social Sciences Research in Jiangsu Universities (2019SJA1932); the Youth Fund Project of Humanities and Social Sciences Research of Ministry of Education (21YJC790086); the key project of Humanities and Social Sciences (SK2021A0776) and the Anhui Philosophy and Social Science planning Project (AHSKY2020D53).

## LHMM23204 TEACHING INTANGIBLE CULTURAL HERITAGE AND TOURISM MANAGEMENT FROM THE PERSPECTIVE OF STUDENT MENTAL HEALTH

Chunlu Gao^a^

*^a^ Chongqing Aerospace Polytechnic College, Chongqing, China.*

**Background:** With the improvement of people’s living standards, contemporary individuals are constantly pursuing higher levels of experiential satisfaction in their daily lives. Travel demand, to a large extent, is inspired by the diverse and colorful cultural heritage found throughout different destinations, providing opportunities to experience and explore the diversity and uniqueness of culture. Intangible cultural heritage embodies the civilization of human society and represents the diversity of global cultures. Introducing intangible cultural heritage-related courses to students majoring in tourism management at vocational colleges is beneficial for enhancing their sense of pride in traditional Chinese culture, cultivating their interest in further learning and researching intangible cultural heritage, and encouraging them to find their life goals and build self-confidence, thereby promoting their mental health development.

**Subjects and Methods:** The paper analyzed the impact of integrating intangible cultural heritage into tourism management education on enhancing students’ self-efficacy, promoting cultural identity, and fostering respect and dedication. Further, it explored specific intangible cultural heritage elements that can be integrated into the teaching process, and proposed targeted strategies for incorporating these elements into the curriculum.

**Results:** The findings indicate that integrating intangible cultural heritage into tourism management education allows students to gain knowledge and insights about intangible cultural heritage and its inheritors. Incorporating intangible cultural heritage into the growth process of cultivating personal character, developing professional skills, and fostering ethical values can produce positive emotions and fulfill students’ psychological needs, mitigating the negative impact of emotions such as depression, low self-esteem, and sensitivity. This integration produces a significant positive effect on students’ mental health and contributes greatly to their growth and development in the future.

**Conclusions:** Based on the findings, it is recommended that the teaching curriculum of tourism management should be developed based on real-world contexts, with a focus on the theme of “protection and inheritance of intangible cultural heritage”. It is crucial to recognize the significant role that tourism plays in safeguarding intangible cultural heritage and to undertake education reform and practical exploration to promote the simultaneous development of intangible cultural heritage preservation and tourism management education towards a “win-win” outcome.

### Acknowledgements

This work was supported by the General Project of Higher Education Science Research of Chongqing Higher Education Institute from 2019 to 2020 “Research on Teaching Reform of ‘1+X’ Certification System in Vocational Tourism Management Program” under Grant number CQCJ19B142.

## LHMM23205 QUESTIONNAIRE SURVEY ON CURRENT ANXIETY SITUATION OF PARENTS OF CHILDREN WITH SPECIAL NEEDS AND ANALYSIS OF RELATED FACTORS

Leyi Shao^a^, Xinyu Lao^a^, Yexiao Liang^a^, Xia Chen^a^, Rongchao Gong^a^, Liting Liu^a^

*^a^ School of Education Science, Nanning Normal University, Nanning 530299, China*

**Background:** As direct participants in the education and rehabilitation of children with special needs, the anxiety of parents of children with special needs affects the rehabilitation process of children with special needs to a certain extent. In this study, we investigated the anxiety of parents of children with special needs and assessed the relevant factors affecting parents’ anxiety levels.

**Subjects and Methods:** In this study, a questionnaire was administered to 176 parents of children with special needs using the Self-Assessment Scale for Anxiety (SAS) and the results were statistically analysed using SPSS 20.0 software to investigate the anxiety status of parents of children with special needs, including basic personal information of the parents, basic information of the children, educational status of the children, monthly household income and educational status. At the same time, in order to ensure the completeness and richness of the information required for the study, a foundation was laid for the subsequent data collation and analysis. The researcher also conducted interviews with the parents of special children.

**Results:** This study investigates the anxiety of parents of children with special needs and analyses the relevant factors that influence the level of parental anxiety, which consists of basic information about the family and the basic situation of the child. The anxiety index of parents of special children was shown to be (56.16 ± 12.55) points, which are at a mild level of anxiety. Among these factors, parental gender, age, type of family of the special child, parental status of the special child, occupation, education, monthly household income, number of children, gender of the child, type of disability of the child, degree of disability of the child, pattern of rehabilitation expenditure, special school and family placement had no effect on the anxiety level of the parents of the special child. The total anxiety score of parents of exceptional children was positively correlated with parental status, occupation, degree of impairment and the institution attended, and negatively correlated with the placement of the child in a regular kindergarten.

**Conclusions:** By establishing a sound education system for children with special needs and increasing employment opportunities for parents of children with special needs, as well as conducting regular parent training and parent group support activities, these measures can effectively reduce the psychological pressure on parents of children with special needs.

### Acknowledgments

This study is the phased research achievement of 2023 Guangxi Education Science “14th Five-Year Plan” Topic: Mental Health Status and Service System Construction of Sound healthy siblings in Families with Special Children, Project No.: 2023B145.

## LHMM23206 THE APPLICATION AND RESEARCH OF DATA GOVERNANCE TECHNOLOGY TO MEET PATIENTS’ PSYCHOLOGICAL NEEDS AND WELL-BEING AND PROMOTE HOSPITAL DEVELOPMENT

Wangwang Zeng^a^, Kai Li^a^, Haonan Zheng^a^, Zhihu Ding^a^

*^a^ Information Center, The First People’s Hospital of Yunnan Province, Kunming 650032, China.*

WZ and KL contributed equally to this study.

**Background:** With the continuous development of information technology and the continuous improvement of people’s living standards, more and more people have put forward higher requirements for physical and mental health. While information technology has promoted the rapid development of hospitals, it has also produced a large number of redundant data. Patients have put forward higher requirements for the construction of hospital informatization. This paper will improve the quality of hospital data through a series of data governance measures, Improve the clinical business processes of hospitals to ensure their efficient operation. Through various national assessments such as interconnectivity ratings, three-level public hospital performance evaluations, and electronic medical record ratings, we aim to meet patients’ requirements for hospital informatization construction to the greatest extent possible, enhance people’s sense of medical service happiness, and ultimately promote the high-quality development of modern hospitals.

**Subjects and Methods:** A special team for data governance was set up, a series of data governance rules and regulations were formulated, and software tools of the multi-party data governance platform were introduced. The team members regularly investigated the psychological needs of patients during diagnosis and treatment, and obtained specific opinions of patients on the hospital’s diagnosis and treatment process and information system. Then, the diagnosis and treatment process was optimized, and the information system was transformed to meet the psychological needs of patients to the greatest extent, and improve the happiness of patients during the medical treatment process.

**Result:** The outpatient volume and operational data of discharged patients in the hospital have significantly improved. The outpatient volume of the hospital has increased significantly for five consecutive years, and the average waiting time of patients has decreased for five consecutive years. In the questionnaire survey, it was found that the satisfaction of patients who came to the hospital for medical treatment has been greatly improved, their psychological needs have been met, and their sense of happiness has significantly improved. The hospital has made two consecutive breakthroughs in the performance evaluation ranking of tertiary public hospitals, and ranks first in the province through the interconnection of Class IV and Class A

**Conclusion:** Through data governance, various data indicators of the hospital have been significantly improved, and the hospital has developed at a high speed. Every year, the hospital can provide more patients with better quality diagnosis and treatment services. When patients come to the hospital for treatment, they always have a relaxed and happy diagnosis and treatment experience. While diagnosing and treating patients’ physiological diseases, the psychological needs of patients have also been more satisfied, and the happiness of patients in the area has been greatly improved.

### Acknowledgements

This work was supported by a project grant from Yunnan Provincial Basic Research Program (Grant No.202201AY070001-258).

## LHMM23207 THE DEVELOPMENT TRAJECTORY AND MOTIVATION OF THE ROOKIE NETWORK: BASED ON THE PSYCHOLOGICAL NEEDS AND EMOTIONAL BEHAVIOR OF CONSUMERS

Hui Liu^a^, Hanteng Liao^b^, Shaohe Zhang^c^

*^a^ Business School, Nanfang College GuangZhou, Guangzhou 510970, China, ^b^ Higher Education Impact Assessment Center, Nanfang College GuangZhou, Guangzhou 510970, China, ^c^ School of Economics, Guangzhou College of Commerce, Guangzhou 511363, China.*

**Background:** To realize the practical theoretical innovation of industrial Internet combining modern service industry and advanced manufacturing industry, it is necessary to integrate the existing mainstream theories of sustainable transformation and update the meaningful case studies. Satisfying consumers’ psychological needs and emotional behavior is key to promoting consumer upgrading under the new development pattern of “dual circulation”. The innovative practice theory of industrial Internet can provide more personalized, convenient, and efficient products and services, enabling consumers to enjoy pleasure and satisfaction in the purchasing process. This further enhances consumer loyalty and reputation for brands, bringing greater commercial value to businesses.

**Subjects and Methods:** This paper uses theoretical analysis and case study methods. Based on the practical achievements of Rookie Network, this study firstly analyzes the development process of Rookie Network, as a logistics link under the digital economy, from the earliest information platform to intelligent logistics system with the mainstream theory of Industry 4.0 “Cyber-Physical System” (CPS). As the mainstream multi-perspective sustainable transformation “social-technical system” theory of sustainable transition, CPS is used to demonstrate and analyze the transition process of rookie network from the earliest odd-job economic platform to the “low-carbon” social-technical system that provides consumers with high-level emotional value and upgrades consumer experience.

**Results:** The construction of intelligent logistics based on consumer psychological needs of intelligent logistics construction from C-end consumers and consumption scenarios must include the overall theoretical view of Industry 4.0 CP Logistics and multi-perspective sustainable transformation “social-technological system”. Only in this way can logistics companies meet consumers’ increasingly high demands on logistics. Help them to get logistics information quickly and accurately. Enjoy convenient and efficient logistics services, and improve the sense of experience and service quality in the logistics process. Get a sense of pleasure and satisfaction over good logistics services.

**Conclusions:** It can ensure that the digitalization and digital transformation capabilities at the consumer side can be extended to the working side of commerce and industry, and drive the industrial transformation and upgrading path of advanced manufacturing industry with the deep integration of digital capacity building. Thus, enterprises can better understand consumer needs and launch products and services that meet their psychological needs and emotional behavior more accurately, and sustain development.

### Acknowledgements

This work was supported by two projects grant one from Nanfang College, Guangzhou A study on the influence of hog futures on hogs and the industry chain price of hogs (Grant No. 2021XK25), the other from The reform of digital teaching mode of Internet finance based on project management, a general project funded by the 14th Five Year Plan of China Association of Higher Education - A case study of Theory and Practice of Internet Finance (Grant No.21SZYB29).

## LHMM23208 ANALYSIS OF STANDARDIZED MODE OF AGRICULTURAL PRODUCT SUPPLY CHAIN FROM THE PERSPECTIVE OF PSYCHOLOGICAL NEEDS - A CASE STUDY OF TEA INDUSTRY

Jie Li^a^

*^a^ MBA Center, Shanghai University, Shanghai, 200444 China.*

**Background:** To alleviate the problems of extensive production, insufficient investment in cold chain, high transportation loss, and asymmetric information in China’s agricultural product supply chain, regulate the management of the agricultural product supply chain, meet consumers’ psychological needs for high-quality agricultural products, and guide suppliers to optimize the agricultural product supply chain mode based on consumers’ attitudes, emotions, and hobbies.

**Subjects and Methods:** Based on consumers’ psychological needs for tea product quality, this paper took the agricultural standardization policies issued by 16 provinces in China as the basis and applied the panel data from 31 provinces between 2000 and 2020. Multiple-period difference-in-difference (DID) method was used to study the growth effect of the agricultural standardization work plan on the tea industry in China.

**Results:** The results showed that the promotion of agricultural standardization work and the support of relevant policies can promote the growth of local tea production and also have a promoting effect on the growth of local tea value. Furthermore, it can effectively meet the psychological needs of consumers for high-quality tea, and influence consumers’ purchasing motivation and behavior.

**Conclusion:** Agricultural standardization has a positive impact on the yield and output value of agricultural products, is an important guarantee for promoting the development of the agricultural industry, and is also an important prerequisite for the standardization construction of the agricultural supply chain. It is recommended that China accelerate the research on the standardization of agricultural product supply chain, and take specific measures such as “continuously strengthening the standardization of agricultural product supply chain, developing universal agricultural product supply chain standards; strengthening the construction of organizational guarantee measures to ensure the implementation of agricultural product standardization plans; strengthening the construction of logistics infrastructure to enhance the responsiveness of agricultural product inventory and distribution”. This provides new ideas, new methods, and new channels for China to carry out agricultural product standardization construction, meeting consumers’ psychological needs for agricultural products’ freshness. Additionally, this has important theoretical and practical implications for the quality upgrading of agricultural product circulation.

## LHMM23209 AUTISM COMORBIDITIES OF MENTAL RETARDATION AND EPILEPSY WITH CISTERNA MAGNUM OCCIPITALIS: A RARE CASE REPORT

Cunbo Wu^a^, Kexiang Cheng^a^, Xiaolin Tan^a^

*^a^ Chongqing Mental Health Center, Gele Mountain, Shapingba District, Chongqing,400036, China.*

**Background:** Autism comorbidities of mental retardation and epilepsy with cisterna magnum occipitalis in children are rare, Early recognition by clinicians is difficult, which led it easy to be neglect and difficult to thrapy that affect the children’s mental health and physical health and increased the burden on the family

**Subjects and Methods:** A child with autism comorbidities of mental retarded development and epilepsy with cistercius macroccipitalis was promptly treated with oxazepine for antiepilepsy from Chonging mental health center, he was emotional unstable, irritable, impulsive wounding, accompanied by obvious mental symptoms and anxiety, repeated toilet visits, could not speak human language, could not take care of his own life. We used Risperidone oral liquid for antipsychosis, Quetiapine combined with Ziprasidone injection for mood stabilization, completed relevant auxiliary examination, supplemented by rehabilitation training such as psychotherapy, behavior modification, hypnotherapy, biofeedback therapy to promote the rehabilitation of the child.

**Results:** The child became emotional stability, not irritable, impulsive, hurtful and anxious feelings any more, mental symptoms gradually disappeared, could cooperate with simple educational training, although discharged automatically.

**Conclusions:** Autism comorbidities with severe mental retardation and epilepsy are rare and difficult for clinicians to treat due to communication difficulties. Autism and mental retardation mainly rely on education training and psychotherapy, while epilepsy and primary disease caused by mood disorders, clinical psychiatry mainly medicated to solve. The patient is accompanied by large cisterna, whether it is the cause of the disease or coincidence, and whether it is used as the auxiliary basis for diagnosis, needs a large number of clinical samples and data to study. For such patients, it is also necessary to be cautious in drug use. Due to the retarded neurological development, the drug sensitivity is reduced and the drug tolerance is easy, so the choice of drugs should be more careful. Early identification, timely antiepileptic treatment and appropriate drug sedation therapy are very necessary for clinicians to pay attention to Comorbidities of mental retardation and epilepsy in children.

## LHMM23210 RESEARCH AND PRACTICE ON TRANSLATION STRATEGIES OF FILM AND TELEVISION STYLISTICS FROM THE PERSPECTIVE OF TRANSLATORS’ MENTAL HEALTH

Weiwei Liu^a^

*^a^ Basic Teaching Department Shandong Huayu University of Technology, Dezhou, Shandong, 253000, China.*

**Background:** From the perspective of mental health, this paper uses cognitive psychology, personality psychology and Nida’s functional equivalence theory to further explore the translation strategies of English film and television drama language, so that film and television art can better play the role of film and television art in people’s mental health and personality. The positive role of shaping clarifies the linkage relationship between the translation of film and television texts in promoting people’s mental health and all-round development, in order to provide reference for the development of film and television style translation.

**Subjects and Methods:** In order to promote the healthy development of people’s mental health, this paper discusses the principles, methods and strategies of film and television translation on the basis of analyzing and summarizing the characteristics of film and television stylistic language - colloquial, easy to understand, cross-cultural and immediacy. According to the different contexts, semantics, and discourse conditions of specific translation examples, the principles and effects of the application of translation, free translation, abbreviated translation, and compilation strategies are emphasized.

**Results:** In the process of translating film and television scripts, the translator must face the audience and give more consideration to the audience’s objective needs, aesthetic taste and acceptance level, so as to finally achieve harmony and unity between the audience’s expectations and the original intention of the translation, and improve the expression and integration of individual emotions. Control ability, promote the formation of human personality and the overall development of mental health. At the same time, we must strive to:

1. Make good use of cognitive psychology theories and laws of cognitive psychology to complete the understanding and expression of the original text, actively mobilize the translator’s psychological activities in the translation process, and improve the quality of translation.

2. Make good use of the positive influence of personality factors in the translation process of film and television style, determine the translation objects and expressions, maintain the original style, and improve the translation quality.

**Conclusions:** The translation of film and television style should not only pay attention to the expression of artistic techniques and stylistic characteristics, but also be people-oriented. It is absolutely necessary to promote people’s mental health. Carrying out the translation strategy research of film and television style from the perspective of mental health can not only make the translator’s personality tendency and film and television text realize two-way adaptation, but also make the correlation between the translator’s cognitive activity law. The people-oriented concept has been deeply integrated, and the translator’s emotional activity rules have been fully reflected in the translation examples, which has opened up a new path worth trying for the translation of film and television genres.

### Acknowledgments

This article is the phased research result of the 2019 scientific research platform research project of Shandong Huayu University of Technology - Applied Translation Research Center (No. 09) and College English Curriculum Construction.

## LHMM23211 THE IMPACT OF IMPROVING MISCELLANEOUS TUNES IN FUJIAN AND TAIWAN GEZI OPERA BASED ON TRADITIONAL CHINESE OPERA ON MENTAL HEALTH

Chin-niang Chan^a^

*^a^ School of Art, Minnan Normal University, Zhangzhou, Fujian, China.*

**Background:** As a product of artistic development, traditional Chinese opera is associated with human emotions through the process of visual and spiritual experience, thereby having a profound impact on human mental health. Through in-depth analysis of how traditional Chinese opera art affects emotions through visual experience, this study explains the close relationship between traditional Chinese opera art and mental health from a micro perspective, using the way that traditional Chinese opera art affects mental health.

**Subjects and Methods:** This study examines the relationship between art and mental health in art therapy, using art based techniques such as traditional Chinese opera music as evidence based interventions for mental health issues such as anxiety and depression. More and more evidence suggests that art can be used in non-therapeutic environments to promote mental health, such as performing arts to understand the core subject areas of a school or to maintain a sense of mental health. In other words, practicing art can be used to establish the ability to manage a person’s mental and emotional health.

**Results:** The research results show that art affects mental health, and the healing effect of art works needs to be carried out to the extent that the audience can understand. If opera has a psychological relief and healing effect on the audience, then the art works or performers of this opera are effective for these audiences. The direction of this study is to focus on the artistic works themselves, as well as the psychological world and healing function of opera performances, in terms of art and mental health.

**Conclusions:** The impact of traditional Chinese opera art on mental health is mainly produced through aesthetic experience. In the process of creating traditional Chinese opera art, individuals tend to pay attention to the expression of their physical and emotional tendencies in the actual inner experience of the artistic work; In the process of appreciating traditional Chinese opera art, individuals experience emotional flow and emotional release through re-experiencing the emotions expressed in artistic and technical works. The integration of artistic experience and artistic techniques can enable an individual to gain respect for their own feelings, thereby achieving the release and sublimation of the surrounding environment within. Show the artistic style characteristics of Gezi Opera and its healthy body and mind.

## LHMM23212 RESEARCH ON THE PRACTICAL LOGIC AND PSYCHOLOGICAL ANXIETY OF ARTIFICIAL INTELLIGENCE EMBEDDED IN SOCIAL SECURITY GOVERNANCE

Tuo Shi^a,b^, Jie Fu^c^, Chi Zhang^a^

*^a^ Beijing Police College Department of Public Security Management, Beijing 102202, China, ^b^ Institute of Scientific and Technical Information of China, Beijing 100038, China, ^c^ Beijing Miyun District Administration Bureau, Beijing 100050, China.*

**Background:** With the increasing application of artificial intelligence (AI) in the field of public security and law enforcement, its integration into the practice of law enforcement is no longer a one-dimensional technical innovation, but a multidimensional empowerment that includes promoting the formation of multi-subject collaborative governance models, forcing upgrades of supporting institutional frameworks for public security, and driving the technological transformation of law enforcement methods. While AI has effectively improved law enforcement efficiency, it has also brought risks such as discrimination, privacy infringement, and deprivation of rights, causing psychological anxiety about technology.

**Subjects and Methods:** This study discusses the negative effects of law enforcement caused by technology defects. It is supported by the theoretical framework of comprehensive public security and law enforcement, and integrates methods such as situational analysis and systematic deconstruction. The study systematically analyzes the types of psychological anxiety risks caused by AI technology integration into law enforcement, and investigates the inducements and impacts of psychological anxiety from the multidimensional perspectives of structural imbalances of the law enforcement subject, ineffective governance systems, and technological alienation.

**Results:** From the perspective of the relationship structure of governance subjects, the monopoly of AI technology resources undermines the original intention of promoting the participation of multiple subjects in governance, and can even cause risks such as social disorder and psychological imbalance caused by the feeling of injustice among subjects. It becomes a huge obstacle to the modernization of governance capacity, deviating from the governance goal of “joint construction, joint governance, and sharing”. From the perspective of the role of governance subjects, it causes further polarization and even group isolation and confrontation among social subjects, and algorithm-driven social polarization and even group isolation and confrontation causes more serious psychological disorder. From the perspective of technological alienation, over-reliance on data algorithms and the risk of uncontrolled application bring insurmountable psychological anxiety to subjects.

**Conclusions:** The study results show that the main ways to effectively alleviate the psychological anxiety of subjects at the technical level include: the government’s comprehensive coordination of the structural relationship among multiple subjects under the embedding of AI, promoting the formation of a multi-subject collaborative governance pattern, following up and improving warning decision-making, information resource sharing, and public security and crime prevention and control systems, and accelerating the construction of an “AI governance system”.

### Acknowledgements

This work was supported by a project grant from National Social Science Foundation Youth Program (Grant No.21CSH05).

## LHMM23213 THE IMPACT OF MAJOR SHAREHOLDERS’ SHAREHOLDING STRUCTURE ON THE PSYCHOLOGICAL ANXIETY OF SHAREHOLDERS, BASED ON THE PERSPECTIVE OF STOCK PRICE CRASH RISK

Weichao Cheng^a^, Shi Zheng^b^, Feng Hu^a^

*^a^ School of Economics and Management, West Anhui University, Luan 237012, China, ^b^ School of Business, Sichuan University Jinjiang College, Meishan 620860, China.*

**Background:** The collapse of stock price will quickly evaporate the wealth of shareholders, greatly increasing their anxiety. Anxiety can have a dual impact on people’s physical and mental well-being, Anxiety can bring complex and unpleasant emotions such as tension, unease, worry, annoyance, Insomnia and dreaminess to people, thereby affecting family harmony and happy life. Due to the late development of China’s capital market, there is room for improvement in China’s market regulatory mechanism and legal system, and the stock price crash risk are higher risk.

**Subjects and Methods:** This paper using the Shapley-Shubik power index, which is based on cooperative game theory, and investigates the impact of major shareholders’ shareholding structure on the psychological anxiety of shareholders, based on the perspective of stock price crash risk. stock price crash risk by examining a sample of A-share listed companies in China from 2003 to 2018. This study uses regression methods such as least squares to empirically test the research hypotheses proposed in this paper.

**Results:** The study finds that: (1) Cash flow rights significantly reduce stock price crash risk, alleviated the anxiety of shareholders. The separation of cash flow rights and control rights significantly increases such risk, increased the anxiety of shareholders. While the control rights have no significant effect on the stock price crash risk; (2) More supportive behaviors and fewer hollowing-out ones play a partially intermediary role in the suppression of stock price crash risk by the cash flow rights, whereas fewer supervisory and more hollowing-out behaviors play a partially intermediary role in the crash effect of separation degree of control and cash flow rights.(3) The more powerful a firm’s management and the weaker its external supervision, the more significant the role of the cash flow rights in suppressing stock price crash risk, and the more conspicuous the crash effect of separation degree of control and cash flow rights.

**Conclusions:** Cash flow rights significantly alleviate the anxiety of shareholders, while the separation of cash flow rights and control rights significantly increases the anxiety of shareholders. The findings of this paper could provide useful theoretical and practical guidance for optimizing firms’ shareholding structure, suppressing the risk of share price collapse, improving the quality of listed companies, promoting the stability of capital markets, and alleviating the anxiety of shareholders.

### Acknowledgments

This research was funded by West Anhui University Talent Introduction School Research Start-up Fund Project (No. 00701092302) and Youth foundation of SCUJJ (No. QNJJ-2022-A07).

## LHMM23214 EMPIRICAL ANALYSIS AND PROPERTY RIGHTS INTERPRETATION OF MASS LABOR AND CAPITAL INCIDENT UNDER THE PERSPECTIVE OF EMOTIONAL BEHAVIOURS

Ling Miao^a^, Yun Yang^b^

*^a^ The CPC Henan Provincial Party Committee Party School, Zhengzhou 451464, China, ^b^ School of Economics and Management, Zhongyuan University of Technology, Zhengzhou 450007, China.*

**Background:** As one of the most important social relationships in market economy, the labor-capital relationship is “the axis of rotation around all modern social systems” (Engels). Nowadays, this relationships under China’s “national integration model” has basically completed the market-oriented transformation, but the labor-capital dilemma has become increasingly severe. According to the Chinese Law Blue Book, labor-capital incidents have accounted for 36.5% of China’s mass incidents, which become major threats to China’s economic and social construction, possible solutions thereby are needed. And in the current era where emotional health is becoming increasingly important, using emotional behavior as a research entry point can more intuitively reflect internal mechanisms and problems.

**Subjects and Methods:** Based on the statistical data from 2000 to 2015, this paper concludes that (i) the number of mass labor disputes is proportional negative correlated to the labor compensation, while (ii) the number of mass labor disputes is proportional positive correlated to the capital property income with the cointegration theory and the generalized impulse response function. This paper studies the endogenous relationship between the loss of labor property rights, the decline of labor compensation proportion and the “outside institutional struggle” under property rights theory. Combined with data validation, it verifies the relationship between emotional behavior and large-scale industrial events, and verifies that emotional behavior can have an impact on industrial events.

**Results:** We propose to (i) increase the proportion of labor compensation at the initial distribution stage as the reform direction; (ii) improve the “three rights of labor and capital”; (iii) establish a long-term mechanism for wage growth; and (iv) develop the redistribution and adjustment mechanism of compensation to solve the problems mentioned above. And attention should be paid to the prevention and guidance of negative emotions and unhealthy behaviors caused by negative emotions.

**Conclusions:** The decline in the proportion of labor compensation does not necessarily lead to mass labor incidents, but in a long run, labor compensation and capital property income have a causal relationship with mass labor disputes. That is, the continuous decline in the proportion of labor compensation makes workers fall into a psychology of disadvantage and their “deprivation feeling” become solidified. An accidental event may lead to the mass labor-capital incidents. Furthermore, the consequence of the generalized impulse response function indicates that the number of mass labor disputes is negative correlated to labor compensation, while positive correlated to capital property income, which presents a crowding effect on labor compensation. Obviously, the long-term crowding effect of the labor compensation and the continuous decline of the labor compensation ratio have become the inherent reasons for mass labor and capital incidents. The prevention and guidance of emotions and behaviors can effectively solve the current practical problems in enterprises or society. Therefore, the prevention of negative emotions and the guidance of unhealthy behaviors caused by negative emotions are urgent.

### Acknowledgements

The period research results of the National Social Science Fund General Project “Research on the Fire-retardant Mechanism and Governance of Massive Labor Incidents Network” (No. 18BGL237).

## LHMM23215 TIME SERIES PREDICTION METHOD OF INFECTIOUS DISEASES BASED ON SCALED DOT-PRODUCT ATTENTION-LSTM AND CNN-FILM

Jinyang Liu^a,b^, Boping Tian^a^

*^a^ School of Mathematics, Harbin Institute of Technology, Harbin 150006, China, ^b^ School of Statistics, Chengdu University of Information Technology, Chengdu 610103, China.*

**Objective:** It is common knowledge that in the history of the world’s medical development, various infectious diseases have been the most dangerous and deadly diseases for human health. Considering that the virus has not been fully explored and the scale of contagion is becoming more and more widespread, there is an urgent need for timely prediction of the spread of infectious diseases. With the continuous development of artificial intelligence in recent years, deep learning techniques have been used to predict infectious diseases in addition to traditional prediction methods. Therefore, proposing prediction models with better performance based on depth techniques is the main objective of this study.

**Methods:** Long short-term memory (LSTM) and convolutional neural networks (CNN) are deep learning models, both of which perform well in predicting time-series data. Based on summarizing previous studies, the LSTM based on scaled dot-product attention and the CNN combined with feature-wise linear modulation (FiLM) were proposed. These methods improve the ability to capture key information about infectious diseases, which in turn improves the predictive performance of the model. To evaluate the fitting performance of those models, LOSS, RMSE, Pearson coefficient, and R^2^ of models were calculated in this study.

**Results:** The fitting results of Scaled Dot-product Attention-LSTM and CNN-FiLM on time series data of infectious diseases from four countries were obtained based on Python. In conjunction with the calculated results of the evaluation metrics for model fitting, it can be seen that the two methods proposed in this study, based on Scaled Dot-product Attention-LSTM and CNN-FiLM, are suitable for the effective prediction of infectious disease time series, and both methods have improved to some extent in terms of model fit. Compared with Japan and Armenia, the two improved methods were relatively better fits for the epidemic data from China and India.

**Conclusions:** To ensure effective prediction of infectious diseases, it is necessary to actively explore the establishment of excellent methods and models, strengthen the ability of models to be applied to different data, and minimize the error rate in prediction. Scaled Dot-product Attention-LSTM and CNN-FiLM can be easily adapted to many different temporal data analysis tasks, which greatly increases the scalability of time series prediction models. This study makes some attempts at the prediction methods and models of infectious diseases, and the research results may play a certain role in the effective response to similar infectious diseases that may occur in the future.

### Acknowledgements

This work was supported by a project grant from the Natural Science Foundation of China(NSFC) (No.91646106).

## LHMM23216 EXPLORATION ON THE IMPLEMENTATION STRATEGY OF THE OVERALL DEVELOPMENT OF POSTGRADUATES FROM THE PERSPECTIVE OF STUDENT MENTAL HEALTH

Xihuang Lai^a^, Zhihui Yang^b^, Jing Cao^c^, Zhi Fang^d^

*^a^ Graduate School, North China University of Technology, Beijing 100144, China, ^b^ Brunel London School, North China University of Technology, Beijing 100144, China, ^c^ School of Electrical and Control Engineering, North China University of Technology, Beijing 100144, China, ^d^ School of Humanity and Law, North China University of Technology, Beijing 100144, China.*

**Background:** As the fresh troops in national innovative talents cultivation, postgraduate education shoulders the important mission of building China into a world leader in science and technology. However, the increasingly complex international environment and fierce competition of employment prospects make postgraduates bear great pressure. Some students even have mental problems. In order to alleviate the anxiety of the postgraduates nowadays, we put forward the goal of all-round development of talent cultivation on the basis of mental health with the corresponding assessment indicator system, trying to cultivate the sound personality and comprehensive quality of graduate students from the macro level of management and the micro level of personal development.

**Subjects and Methods:** Firstly, through the method of literature survey, this paper analyzes in detail the practical dilemma and implementation bottleneck of mental health education based on physical, aesthetic and labor development at the graduate level. Then it combined with the management characteristics of our school, as well as the mental education, to establish a cultivation framework for the all-round development of graduate students. Secondly, according to the requirements of the comprehensive development in moral, intellectual, physical, aesthetic and labor aspects, and the demand of innovative-applied talents, we designed a relatively systematic questionnaire about postgraduate learning experience and mental health status, and carried out long-term data survey of our graduate students. On this basis, we further optimized the process of talent cultivation by putting forward the implementation strategy of “moral and intellectual education as the foundation, mental health education as the connection, practice as the way, team as the body part and multiple educations working together”.

**Results:** Under the influence of the reform of the postgraduates’ comprehensive development mechanism of our university, the self-assessment survey of postgraduates’ learning experience and mental health in recent two years shows that their degree of satisfaction with the work of multi-education integration carried out by our university is on the rise. We have successfully diverted students’ attention and relieved their psychological problems caused by employment pressure, academic pressure, classmate relationship and emotion factors through conducting physical, aesthetic and labor education. And we have gradually shaped students’ healthy psychology and sound personality as well as guide their all-round development in the process of education.

**Conclusions:** The mental health of graduate students has been paid more and more attention, and universities are focusing on solving students’ mental problems. We promote the organic integration of five educations with mental education, continue to improve the assessment system of graduate talent cultivation and optimize the implementation approach, urge the graduate management department to enhance the working methods through the way of assessment, and guide the graduate students to form good research habits and healthy mental state, to further improve the quality and effectiveness of graduate education. It is believed that the implementation strategies will provide a strong support and guarantee for cultivating high-level talents with excellent professional quality, noble character and mental health.

### Acknowledgements

This work was supported by the ideological and political work research project of Beijing universities (support project): Construction and implementation of the comprehensive development of postgraduates oriented by ability cultivation (Grant No. BJSZ2021ZC63), the Beijing higher education association project (general project): Exploration and research on the quality evaluation of graduate practice in the new era (Grant No. MS2022291).

## LHMM23217 A STUDY OF DEMOTIVATING FACTORS AMONG CHINESE COLLEGE STUDENTS IN EFL CLASSROOMS AND POSSIBLE MOTIVATIONAL STRATEGIES FOR REDUCING PSYCHOLOGICAL STRESSORS AND CLASSROOM ANXIETIES

Xuemin Zhu^a^

*^a^ School of Foreign Languages, Sichuan University Jinjiang College, Pengshan, Sichuan 620860, China.*

**Background:** L2 motivation research has already attracted much attention in L2 field and this paper mainly focuses on the explorations of possible causes of low motivation in English learning in colleges of China and L2 motivational strategies in English Teaching of Chinese Context. Under the condition of maintaining and improving students’ mental health, the investigation of the demotivation in EFL classrooms also takes psychological factors into consideration, which would probably help Chinese students ease academic stress and relieve anxiety in EFL classrooms, with the purpose of building students’ confidence in L2 learning. Motivational problem also could be part of the contributions to promoting more effective language learning and teaching.

**Subjects and Methods:** On the basis of related literature review, a qualitative research method is adopted in this study and therefore observation is chosen to be employed as the major method in the research. Meanwhile a case study is conducted among the college students. The discussing objects are mainly confined to the non-English majors who are studying in some colleges of the southwest city of China, in order to make the topic more clearly defined and elaborated.

**Results:** Motivation to learn a foreign language depends on how much the individuals would like to learn L2 and how much satisfaction they would gain during the process of learning. In observational studies, the target learners have low motivation in EFL classrooms and the demotivating factors involves a series of problems, among which negative attitudes towards language learning have detrimental influence on the success of acquiring L2. Negative attitudes towards EFL classrooms may be caused by some psychological factors, including classroom anxiety and academic stress. Teaching methods, classroom atmosphere, teacher-and-student relationship and facilities problem are also taken into account.

**Conclusion:** Negative attitudes are common and unavoidable in the discussed context, however, there is a more general recognition that suitable strategies may offer good chances to convert negative attitudes towards L2 learning into positive ones and even promotes the enjoyable process of language learning in some interesting ways. Moreover, this paper examines the teachers’ role of setting up rapport with students in the motivational perspective and how it works as a spur to make L2 learners more confident and engaging in English learning. It also illustrates how important it is to create a pleasant and supportive environment for L2 learners to ease the pressure and anxiety in the process of language learning.

## LHMM23218 APPLICATION OF TRADITIONAL CHINESE MEDICINE EMOTIONAL THERAPY IN IMPROVING ALZHEIMER’S DISEASE

Ruiyue Zhu^a^, Chunting Diao^b^

*^a^ Clinical College of Traditional Chinese Medicine, Hubei University of Chinese Medicine, Wuhan, Hubei 430061, China, ^b^ School of Medical Humanities, Hubei University of Chinese Medicine, Wuhan, Hubei 430070, China.*

**Background:** Alzheimer’s disease has become one of the epidemic diseases in contemporary human society. In recent years, the number of elderly people in China has increased year by year, and the aging process has obviously accelerated. Among them, people with Alzheimer’s disease in China have occupied a large proportion of the population. Alzheimer’s disease, as an elderly disease that will lead to serious consequences and extensive effects, should attract people’s extensive attention. Emotional therapy of Traditional Chinese Medicine is a kind of therapy based on the theory of Traditional Chinese Medicine, which is simple to operate and rarely has side effects. This paper discusses the effect of emotional therapy of Traditional Chinese Medicine on improving Alzheimer’ s disease, and provide the application path.

**Subjects and Methods:** Research subjects are elderly patients with Alzheimer’s disease, generally considered to be 45 years old and above. Patients receive treatment from the emotional theory of Traditional Chinese Medicine, mainly including transforming emotions, meeting demand, dispelling doubts, diverting attention, hinting to switch ideas. This paper uses the method of literature review to sort out and analyze the literature related to emotional therapy of Traditional Chinese Medicine and Alzheimer’s disease in recent 20 years.

**Results:** The theory of emotion of Traditional Chinese Medicine emphasizes that the human body can help repulse the influence of negative emotions by mobilizing the positive emotions of the whole body, restore the body to a peaceful state, and balance the function of viscera thereby relieving or treating various diseases. Depending on the patient’s condition, different Traditional Chinese Medicine emotional therapies can be selected. When the patient is emotional, transforming emotions therapy can be selected. When the patient has requirements, meeting demand therapy can be selected. In addition, the patient can also be enlightened by dispelling doubts therapy firstly. If the effect is unsatisfactory, hinting to switch ideas therapy can be continued. Based on the above, Traditional Chinese Medicine treatment mainly based on psychotherapy can effectively improve the symptoms of Alzheimer’s disease.

**Conclusions:** Traditional Chinese Medicine emotional therapy can effectively regulate the abnormal emotions of patients, help patients to release themselves, obey their wishes, and promote physical and mental health. It has a positive effect on improving the symptoms of patients, and is worthy of comprehensive promotion.

### Acknowledgements

This work was supported by a project grant from the Hubei Provincial Department of Education’s Philosophy and Social Science Fund Project in 2020 (Grant No.20Q075).

## LHMM23219 THE IMPACT OF NEW MEDIA DEVELOPMENT ON BEHAVIORAL ABILITY AND MENTAL HEALTH CHANGES OF THE ELDERLY

Qi Chen^a^, Nur Syuhada binti Mat Sin^a^, Afeez Nawfal bin Mohd Isa^a^, DuoBao Chen^b^

*^a^ Sultan Idris Education University, 35900, Tanjong Malim, Perak, Malaysia, ^b^ Anhui University of Science and Technology, 232001, Huainan, Anhui, China.*

**Background:** The development of new media has brought significant changes in the way people communicate and engage with the world. However, elderly people may struggle to keep up with the fast-paced technological advancements due to changes in their cognitive and physical capabilities. The prolonged exposure to new media may have adverse effects on senior mental health, such as depression, anxiety, and loneliness. Moreover, seniors’ decreased ability to concentrate and process information may lead to difficulty in navigating complex digital interfaces, resulting in low adoption rates. It is crucial to consider the changing behavior and mental health of elderly individuals when designing digital technology to ensure inclusivity and accessibility for all age groups.

**Subjects and Methods:** In this research, by analyzing the consumption demand and usage status of new media among elderly people, as well as the factors affecting their sense of happiness in life. we propose measures to optimize the new media platform, establish a comprehensive system for the elderly to enhance their living quality and mental health.

**Results:** By analyzing a quantity of files, the elderly prefer simplicity and may become more conservative in their thinking. They still require social interaction, particularly spiritual support from loved ones. A user-friendly media application could help bridge the digital divide for them. In this research, conducting several points to elaborate key direction for improving the elderly’s living experience and mental health.

**Conclusions:** The proposed measures not only aim to improve the usability and accessibility of new media platforms for seniors but also promote their digital literacy, social engagement, and overall well-being. Through technology training classes and user-friendly interfaces, seniors can enhance their digital skills and feel more confident and connected while using new media. Additionally, optimizing new media platforms to meet the specific needs of the elderly, such as larger fonts, simpler navigation, and audio instructions, can significantly improve their user experience. Ultimately, promoting the adoption of new media among seniors can bring substantial benefits, including improved mental health, social connectedness, and quality of life.

## LHMM23220 RESEARCH ON INFLUENCING FACTORS OF PUBLIC RISK PERCEPTION UNDER MAJOR PUBLIC HEALTH EMERGENCIES

Ying Zhang^a^, Yuewu Zheng^a^, Rumeng Tian^a^, Lei Peng^b^, Mushan Li^a^

*^a^ School of Emergency Management, Henan Polytechnic University, Jiaozuo 454003, China, ^b^ School of Energy Science and Engineering, Henan Polytechnic University, Jiaozuo 454003, China.*

**Background:** In recent years, frequent occurrence of public health emergencies posed a serious threat to social public security. After the occurrence of public health emergencies, people concern about their own health and the unstable situation, along with media and public opinion out of control, emotion focused coping and irrational behavior happen frequently. As the important factor of responding to public health emergencies, public risk perception has important practical value. Therefore, studying the influencing factors of public risk perception under public health emergencies can help the government guide to respond to public health emergencies, so the public’s risk perception ability will improve, and minimize the serious consequences of public health emergencies.

**Subjects and Methods:** First of all, based on the PSR (Press-State-Response) model this article identifies and analyzes the influencing factors of public risk perception. It is concluded that the inherent risk of public health emergencies, the actual progress of the event, the government response, and the media communication as influencing factors and the degree of reaction of the public’s own response behavior as dependent variable to interpret public risk perception level. Secondly, integrating previous research achievements, a theoretical model is constructed based on the research hypothesis of influencing factors of public risk perception under public health emergencies. Thirdly, data are obtained through questionnaire survey, and empirical analysis is conducted using structural equation models. Finally, this article proposes countermeasures and suggestions for scientifically optimizing the level of public risk perception.

**Results:** The inherent risk of events and media communication have a positive impact on public risk perception; The actual progress of events and government response have a negative impact on public risk perception.

**Conclusions:** Improving the public’s risk perception ability, optimizing existing emergency management policies, strengthening the government’s risk communication ability, and improving the media’s risk information dissemination can improve the public’s risk perception ability, and provide a decision-making basis for guiding the public to respond scientifically to public health emergencies.

### Acknowledgments:

This study was supported by the General Project of Humanities and Social Sciences Research in Henan Universities (2021-ZDJH-0139), the Key Science Research Project in Universities of Henan (21A630015), Ph.D. Fund of Henan Polytechnic University (B2017-62), China Engineering Science and Technology Development Strategy Henan Research Institute Strategic Consulting Research Project (2023 HENZDB 05) and the Teaching Reform Research Project of Henan Polytechnic University “Exploration and Practice of Safety System Engineering Teaching Reform in the Perspective of New Engineering”.

## LHMM23221 THE REALITY BREAKTHROUGH OF THE PYRAMID CLASS: THE COMPLEX SOCIAL LANDSCAPE AND PHYSICAL AND MENTAL DILEMMAS IN THE FILM “WRATH OF SILENCE”

Mingyue Sun^a^

*^a^ Nanchang Institute of Technology, Nanchang, 330000,China.*

**Background:** In the film *Wrath of Silence*, due to illegal mining and urbanization, the dual contradiction between urban and rural areas becomes increasingly prominent, forming a completely different social landscape between rural and urban areas. Under the influence of psychological pressure, people living here adopt different ways of making a living, forming a distinct pyramid shaped social structure. In the psychological context, the “silence” of the lower class is a passive and ineffective struggle, while the “silence” of mining owners from the emerging upper class and lawyers from the middle elite class is an active choice. The film intuitively displays the negative psychology of anxiety, inferiority, depression, mania, cowardice, suspicion, greed, indifference, etc. among the pyramid class population. The dual dilemma of physical and psychological forces people belonging to different classes to implement a breakthrough in reality.

**Subjects and Methods:** By talking about the film semiotics analysis, this paper deconstructs the structure of the story hidden in the text and the expression method of the text from the perspective of story, narrative time, narrative space and expression techniques. The details considered from a psychological perspective are as follows. First, through the dual tone of the film’s realism and surrealism (magic) coexistence, we examine how the film establishes the style and tone of the entire film. Secondly, it deconstructs the pyramid shaped social structure through the gaze perspective in Freud’s psychoanalysis. This tower shaped display image symbol is in line with the contemporary Chinese landscape of class opposition and interest conflicts. The collapsed and imbalanced hierarchical structure makes the tragedy presented in the film more profound and powerful. Third, by reconstructing the time sequence of the film, the deeper meaning of metaphors in each image symbol is deciphered. The film examines the different desires pursued by people of different classes.

**Results:** Different from other crime suspense genre narrative films, to find the child is the starting point of the film, but not around the crime committed or crack crime to narrate. Instead, from the Gestalt film theory of mind, a large number of plots are left blank. Through a large number of symbols and plot blank, the audience can think and feel who is the real villain. The film seems to be looking for children, but in reality, it is the pursuit of different desires by people from different social classes.

**Conclusions:** This study focuses on the imbalance of urban-rural development through the film *Wrath of Silence*, and explores the reality of the physical and mental plight of people in China’s classes, especially in the rural areas, exploring the social symptoms of the upper class’s loss of attitude, the middle class’s loss of morality, the bottom class’s loss of speech, and the earthly disorder. And the complexity and diversity of mental health issues will also lead to diverse discussions on the desires, difficulties, and psychology of different social classes in rural China. Furthermore, it brings positive psychological thinking to the audience, in order to alleviate negative psychology such as anxiety, stress, and suspicion.

## LHMM23222 THE IMPACT OF BIOPHILIC DESIGN IN THE WORKPLACE ON THE HEALTH, COMFORT AND WELLBEING OF OFFICE WORKERS

Kexin Jiang^a^; Zhongyan Lin^b^

*^a^ The Bartlett School of Environment, Energy and Resources, University College London, London, WC1H 0NN, UK, ^b^School of International Digital Economy, Minjiang University, Fuzhou, China.*

**Background:** A person spends most of their time indoors. The quality of the indoor environment (IEQ) has a direct impact on people’s comfort and health. In this circumstance, IEQ becomes the professional’s main concern. For many years, offices have been one of the main topics of research. A variety of aspects that affect office occupants’ health, wellbeing, and comfort have been researched and listed by many researchers, including indoor air quality, thermal comfort, and biophilic design.

**Subjects and Methods:** This study focuses on the comfort, health, and wellbeing of office workers. Using Alker et al.‘s structure of office design to produce the literature review structure, the study first develops the overall picture of office design variables with health, welfare, and comfort. Then, a variety of studies are succinctly analyzed and organized to demonstrate the most recent findings in the area. Then, biophilic design as the main focal point in this study is analyzed. With the help of the biophilic design framework, a literature analysis is conducted to summarize the most recent research and offer recommendations for workplaces to improve the health, wellbeing and comfort status of employees.

**Results:** There is information available on every aspect of workplace design, along with their recommendations. The table of research demonstrates the validity of the framework by Alker et al. and the relationships between various elements and the comfort, wellbeing, and health of occupants. The research on biophilic design then presents an ongoing process in the field and the significance of having the biophilic design in workplaces based on the framework for biophilic design. Some of the recommendations, meanwhile, have flimsy supporting data, necessitating more investigation. There is a need to do additional quantitative research on the relationships between biophilic components and health as a result of the limited research approaches in this study and existing research gaps.

**Conclusions:** Based on the findings, stakeholders should take the office design structure into consideration to enhance the working environment. The compiled biophilic design recommendations can help design teams and building owners further optimize their workplaces for the comfort, health, and welfare of their employees. Additionally, a larger variety of constructed surroundings should emphasize the value of biophilic design in order to enhance the relationship between humans and nature.

### Acknowledgments

This work was supported by project grants from The National Key R&D program, the Intergovernmental International Science, Technology and Innovation Cooperation Project (EU Horizon Project-No.2017YFE0118800); Central Financial support for Local Science and Technology Development Special (No.2018L3007); Foreign cooperation project of Fujian Provincial Science and Technology Plan (NO.2020I0040).

## LHMM23223 THE POSITIVE EFFECT OF TRADITIONAL CHINESE HEALTH PRESERVATION AND SELF CULTIVATION CULTURE ON PSYCHOLOGICAL ANXIETY OF COLLEGE STUDENTS

Hua Wang^a^

*^a^ School of Marxism, Xi’an International Studies University, Xi’an 710119, China.*

**Background:** Starting from the value of traditional Chinese health preservation and self-cultivation culture, analyze the main manifestations of current college students’ psychological anxiety, and interpret the positive role and broad value of traditional Chinese health preservation and self-cultivation culture in alleviating college students’ psychological anxiety.

**Subjects and Methods:** The article mainly analyzes and summarizes the combined methods and practical significance of alleviating college students’ psychological anxiety based on traditional Chinese health preservation and self-cultivation cultural concepts and practical practices. In the process of research, the methods of literature research and induction were mainly used. The article summarized and organized the content and characteristics of traditional Chinese self-cultivation culture, as well as the manifestations of college students’ psychological anxiety, including relevant concepts and practices in traditional Chinese culture, as well as the methods to solve college students’ psychological anxiety summarized through practical implementation and exploration.

**Results:** Based on the analysis of the connotation of traditional Chinese health and self-cultivation culture and the main manifestations of college students’ psychological anxiety, this article explores the role of traditional Chinese health and self-cultivation culture in positively alleviating college students’ psychological anxiety, the specific implementation methods, and its broader practical and world significance in the future. This provides new ideas for the management of psychological anxiety among college students, and also provides a model for applying Chinese health preservation and self-cultivation culture to a wider range in the future.

**Conclusions:** The concepts of calmness, harmony, and self consistency contained in traditional Chinese health and self-cultivation culture, as well as traditional practice methods such as traditional Chinese medicine and Tai Chi, have a very significant effect on positively relieving the psychological anxiety of college students. From a broader perspective, China’s traditional health and self-cultivation culture has more practical and extensive value in relieving psychological anxiety for a wider population in the future.

## LHMM23224 RESEARCH ON BLOCKCHAIN COPYRIGHT PLATFORM FOR PSYCHOLOGICAL NEEDS OF INTANGIBLE CULTURAL HERITAGE INHERITORS

Jingtong Zhao^a^, Lintao Fu^b^

*^a^ Department of Electronic Commerce and Law, Beijing University of Posts and Telecommunications, Beijing City 100000, China, ^b^ Department of Software engineering, Beijing University of Posts and Telecommunications, Beijing City 100000, China.*

**Background:** This paper aims to study the intellectual property protection platform for intangible cultural heritage digital works based on blockchain technology. With the development of digital technology, intangible cultural heritage (ICH) works face the challenge of intellectual property protection in digital dissemination. This study constructs a new type of intellectual property protection platform by utilizing the decentralized, transparent, and tamper resistant characteristics of blockchain. The platform adopts smart contract technology to ensure the confirmation, authorization, and supervision of intellectual property rights, and ensures the traceability and tamper resistance of works through the distributed storage and timestamp functions of blockchain. At the same time, the platform also provides a convenient authorization management and copyright trading mechanism, providing a fair, transparent, and efficient intellectual property protection solution for artists and rights holders. Through empirical research and case analysis, this paper verifies the feasibility and effectiveness of a blockchain based intellectual property protection platform for intangible cultural heritage digital works. This platform can not only promote the inheritance and protection of intangible cultural heritage works, but also promote the healthy development of digital art market. In order to protect the copyright of intangible cultural heritage works, assist in the standardized collection, digital protection and dissemination of intangible cultural heritage. In terms of mental health, intangible cultural heritage has a unique therapeutic and soothing effect. Participating in the inheritance and practical activities of intangible cultural heritage can help individuals reduce stress, alleviate anxiety, and promote emotional balance and self adjustment. The aesthetic experience and artistic creation of intangible cultural heritage can also stimulate people’s creativity and imagination, enhance individual self-esteem and self-confidence. In addition, intangible cultural heritage activities can promote social interaction and group cohesion, provide social support and emotional security, and help prevent and respond to mental health issues.

**Subjects and Methods:** This article proposes a blockchain based intellectual property protection platform for intangible cultural heritage digital works to address the copyright issues encountered in the current digitization process of intangible cultural heritage works. Provide a more humane and easy to understand operation process on the platform, better fit the psychological needs of non-genetic heirs.

**Results:** By integrating blockchain technology into the intangible cultural heritage creation and exchange platform, blockchain stores the information of intangible cultural heritage creators and works. Blockchain, as a decentralized, reliable, and secure storage technology, can provide copyright protection for digital works, as well as new ideas and methods for digitizing intangible cultural heritage and IP construction. At the same time, at the level of mental health, the use of blockchain increases the security of data, so that creators can store data more securely, so as to form a platform with a credible degree that meets the psychological expectations of the authors.

**Conclusions:** Taking the IP digitization process of the intangible cultural heritage of Buyi people “Maple fragrans Dyeing” in Daihua Town, Changshun County, Guizhou Province as an example. Non-genetic heirs have a good experience on the platform, which is in line with psychological expectations. This case verifies the effectiveness of using blockchain to confirm the rights of digital works of intangible cultural heritage and further implement the copyright transformation mode.

## LHMM23225 AN ANALYSIS OF COLLEGE STUDENTS’ MENTAL HEALTH AND ITS INFLUENCING FACTORS DURING ONLINE LEARNING

Yingli Guo^a^, Yunying Shi^b^, Fangmei Liang^a^

*^a^ School of Teacher Education, Hechi University, Hechi 546300, China, ^b^ School of Big Data and Computer, Hechi University, Hechi 546300, China.*

**Background:** In schools, issues related to mental health among students have been a significant concern for several years. Before the outbreak, statistical sources indicated a significant and increasing rate of depressive disorders, suicidal behavior, and various other issues in educational institutions among students. This includes issues such as the possibility of being depressed and worst committing suicide. Thus, this qualitative study aimed to explore the Hechi University students’ lived experiences with online learning during the COVID-19 pandemic. Specifically, this analyzes college students’ mental health and it is influencing factors during online learning. Based on the findings recommendations were proposed and the implications were presented.

**Subjects and Methods:** This research utilized a qualitative method of research, particularly descriptive phenomenology. The participants of the study were ten (10) students studying using the online learning platforms, they were selected using convenient sampling. The criteria for the participants’ selection were students from all levels, male or female, and those using the online learning modality. Thus, they are the most suitable participants of this study as they can best give their responses and testimonials based on what they have experienced while using the new learning platform. The data gathering for this study commenced from November to December 2022. The researcher conducted in-depth one-on-one interviews in gathering the data.

**Results:** The overall results suggested that most students expressed a wide variety of unfavorable emotions they believed they had encountered throughout the course of the global outbreak. Throughout the lockdown, several pupils expressed feelings including “frustration,” “constriction,” “anxiety,” and/or “depression.” After then, a few of them discussed why these feelings affected their day-to-day performance, often in significant ways. Thus, it was revealed that distance learning can be strongly demanding for the mental health of the students. Thus, assistance for student’s mental health is needed during online classes since virtual learning exacerbates pupils’ anxiousness throughout a disease outbreak.

**Conclusions:** Online education was deemed to have a severe effect on the student’s mental health. It was recommended that the results of this study shall be presented to the Ministry of education to provide information, plan activities, and strategies, and create innovations to provide sufficient intrinsic and extrinsic motivation to excel and improve their academic achievement and attain quality and relevant education during the pandemic.

### Acknowledgments

This work is supported by Guangxi Natural Science Foundation Project (2020GXNSFAA159172, 2021GXNSFBA220023) and Research Basic Ability Improvement Project for Young and Middle-aged Teachers of Guangxi Universities (2023KY0633, 2021KY0604).

## LHMM23226 EFFECTS OF RURAL TOURISM ON MENTAL HEALTH OF TOURISTS OF DIFFERENT AGES: A CASE STUDY OF SHAANXI PROVINCE, CHINA

Jing Zhao^a,b,c^, Peiyun Guo^a^, Linbo Zhu^a^

*^a^ Institute of Human Geography, School of Tourism, Xi’an International Studies University, Xi’an 710128, China, ^b^Shaanxi Tourism Research Institute, Xi’an International Studies University, Xi’an 710128, China, ^c^ Research Center for Tourism Cooperation and People-to-people Exchange among SCO Nations, Xi’an International Studies University, Xi’an 710128, China.*

**Background:** Growing volume of research suggests mental health benefits of natural environments. Psychological needs and motivations of tourists of different age groups and how to promote mental health of tourists remain relatively unexplored. This research was aimed to examine association between rural tourism and mental health among tourists of different age groups.

**Subjects and Methods:** Analyzing psychological needs and features of tourists of different ages, this study constructed a table to summarize. In addition, this research analyzed the existing resources of rural tourism in Shaanxi, China, and explored how to use these resources to meet the different psychological needs of tourists, relieve their psychological anxiety, and promote their mental health.

**Results:** Rural tourism can promote the mental health of tourists of different ages. Adolescents face a variety of pressures, such as difficulties of interpersonal communication, heavy academic burden, excessive expectations of parents and so on, which can trigger psychological anxiety of varying degrees. Middle-aged people bear the pressure from emotion, home, work, and physical health, in the face of high-intensity work tasks in the workplace, at the same time, bear the responsibility to take care of the elderly and children in the family, is the most anxious stage of life. Unlike adolescents and middle-aged people, loneliness may cause anxiety for elderly people, especially empty-nesters who live alone. Different types of rural tourism can meet different psychological needs. Wildlife tourism can meet psychological needs of seeking novelty and knowledge of tourists. Living in the countryside with beautiful scenery, tourists can experience the natural, simple and sincere lifestyle and cultural expression of the countryside, and meet the nostalgia psychological needs. Forest tourism can enable tourists to appreciate natural scenery, enjoy quiet time, relax in fresh air, relieve the pressure of life, and promote mental health of tourists.

**Conclusions:** People of different ages groups in modern society are under great pressure and prone to psychological anxiety. The natural environment, wild animals and homestays in rural areas can meet the psychological needs of tourists from different angles, so that tourists can obtain happiness and satisfaction, produce a positive tourism experiences, and promote the mental health of tourists.

### Acknowledgements

This research was supported by the Innovation Capability Support Program of Shaanxi (Program No.2022KRM038), Shaanxi Higher Education Teaching Reform Research Project (No.21BY092), Research and Practice Project on Comprehensive Reform of Postgraduate Education of Xi’an International Studies University (No. 22XWYJGA08). The authors also wish to thank the editor and the reviewer for their valuable comments.

## LHMM23227 RESEARCH ON THE PATH OF INFANT CARE AND NURSING FRIENDLY SERVICE UNDER INTEGRATED SERVICE MODEL

Juanmiao Shi^a^

*^a^ Department of Social Work, Zhejiang University of Finance Economics Dongfang College, Jiaxing 314000, China.*

**Background:** With the development of the infant care service industry, all sectors of society gradually focus on how to create a friendly environment for infants and children. Especially the concept of “child-friendly community” advocated in recent years, all sectors of society gradually focus on how to create a friendly environment for infants and children. The independent infant care service model cannot fundamentally solve the parenting pressure of young families and solve the problem of gradually decreasing child ratio. An appropriate service model is urgently needed to relieve the physical and mental pressure of infant families, promoting the healthy growth of infants and their family members. This study combined with the social organization in the process of service integration community resources to assist the practice of infant family nursery demand experience, put forward the appropriate infant nursery service intervention path-integrated service mode.

**Subjects and Methods:** Selecting “z” social organization as research object, this paper expounds how “z” social organization integrates resources of community health service centers, voluntary service organizations and other community social organizations to provide physical, psychological and social services for infants in the community, so as to promote the healthy growth of infants and young children.

**Results:** In this study, social organizations provide services for infants relying on child-friendly community platforms, and an integrated service model suitable for infant care needs is proposed. The integrated infants nursery care model mainly takes baby-friendly service projects as samples, integrates the resource advantages of women’s federations, grass-roots civil affairs departments, medical and health organizations and medical volunteers, provides social support for infants in the community, and creates a child-friendly community atmosphere of “system-friendly, environment-friendly and service-friendly”, responding to the needs of infants and families and promoting the healthy growth of infants.

**Conclusions:** According to the practical experience of social organizations using the integrated service mode to provide services for infants in the community, it is concluded that the integrated service mode has realized the needs of infants in the community to a certain extent, and according to the practical experience of the integrated service mode, it clarifies the roles and tasks of each resource subject in the infant care and nursing. The integrated service model of infant service plays an important role in the healthy development of infant’s physical, psychological and social ability.

### Acknowledgments

This work was supported by a project grant from the 2021 Philosophy and Social Science Planning project of Zhejiang Province “Research on the Improvement of Children’s Psychosocial Ability from the Perspective of Community Governance- -Taking the Children’s Friendly (Grant No.21NDQN301YB), 2023 Zhejiang Province Civil Policy Theory Research Project “Research on the Path of Social Organizations Participating in Children Friendly Services from the Perspective of Community Governance” (Project No.: ZMKT202388) and the key topics of Zhejiang University of Finance Economics Dongfang College “Research on the Construction of Early Development Service System of 0-3 Year Old Children in Zhejiang Province”(Grant No.2020dfy001).

## LHMM23228 THE INFLUENCE OF EMPLOYEE EMOTIONAL BEHAVIOR AND SYSTEM FACTORS ON THE ORGANIZATIONAL EFFICIENCY OF TROUSER AUTOMATIC HANGING SYSTEM

Yarui Huo^a^, Faridah binti Sahari^a^

*^a^ Faculty of Applied and Creative Arts, Universiti Malaysia Sarawak, Sarawak, Malaysia.*

**Background:** The trouser production industry is in the transformation and upgrading of the attack period. Intelligent manufacturing is an important strategic direction for the transformation and upgrading of China’s manufacturing industry, the emotional behavior of employees affects the organizational efficiency of the trouser production line, and the study of organizational efficiency is the core of automated production in the face of the new pattern of industry development, the trouser production line managers fully consider the emotional behavior of employees, the introduction of automated equipment to help improve the organizational efficiency of the production line It is imperative to do so.

**Subjects and Methods:** This paper explores the factors influencing the introduction of an automated hanging system in trouser production lines in four dimensions: employee emotional behavior, i.e., intention to use, user satisfaction, and system quality and organizational efficiency, and constructs a theoretical model of organizational efficiency of an automated transfer hanging system. This study was conducted with 268 front-line employees in a Chinese trouser manufacturing company and analyzed by structural equation modeling (SEM).

**Results:** The results showed that (i) system quality had a significant positive effect on employees’ emotional behavior, i.e., intention to use and user satisfaction; (ii) user satisfaction had a significant positive effect on intention to use. (iii) Employee’s emotional behavior, i.e., intention to use and user satisfaction, positively affects organizational efficiency.

**Conclusions:** This study addresses the results of the study, fully considers the direct and positive impact of employees’ emotional behavior on the organizational efficiency of the automatic hanging system, and puts forward practical and feasible guiding suggestions for trouser manufacturing enterprises to introduce automated equipment and enhance their automation and intelligent development capabilities based on meeting employees’ emotional needs.

## LHMM23229 APPLICATION OF COGNITIVE THERAPY IN REHABILITATION TREATMENT AND NURSING OF DEPRESSION IN ADOLESCENTS IN ETHNIC REGIONS

Xiaolan Huang^a,b^, Deying Lin^a^, Zhaomin Sang^a^, Xin Zhang^a^, Jinhua Wu^a^, Luxiang Hou^a^

*^a^ Department of Psychology, Third Affiliated Hospital of Guizhou Medical University, Duyun 558000, Guizhou China, ^b^ School of Nursing, Guizhou Medical University, Guiyang 550000, Guizhou, China.*

**Background:** To study the positive effect of cognitive therapy in rehabilitation treatment and nursing of depression in adolescents in the southern ethnic regions of Guizhou.

**Subjects and Methods:** 1. The study subjects were adolescents with depression who were officially admitted to our hospital for rehabilitation treatment in the southern ethnic regions of Guizhou from July 2020 to May 2022. 2. They were randomly divided into a clinical pathology research experimental group (referred to as the experimental group) and a clinical pathology control group (referred to as the control group). The control group was given symptomatic conventional drug treatment and routine nursing measures for psychiatric and psychological departments; while the experimental group was given cognitive therapy intervention on the basis of the conventional rehabilitation treatment and nursing measures of the control group. The Hamilton Depression Scale (HAMD) scores of the two groups of depressed patients before and after the intervention were compared. 3. Firstly, analyze and study the gender, age, education level, ethnicity, living environment, and other factors of the control group and experimental group of adolescent depression. 4. Use SPSS 21.0 statistical software for statistical analysis and data processing. The basic data is presented as mean ± standard deviation. Single-factor ANOVA test and post-hoc multiple comparisons (LSD method) are used for statistical analysis of the experimental and control groups.

**Results:** The research results show that: 1. Adolescent depression is mainly concentrated in female Bouyei ethnic middle school students in rural areas. This is mainly due to the lack of care from parents for their female children while working outside the home. This should be highly valued by the school, society, and family, and proper care and frustration education should be given to left-behind children to enhance the life confidence and ability to withstand pressure of female students.2. After rehabilitation treatment intervention, the HAMD scores of the experimental group were significantly reduced compared to those of the control group. The clinical comprehensive evaluation index was significantly better in the experimental group compared to the control group, and the difference had important clinical statistical guidance significance (P<0.05).

**Conclusion:** In the rehabilitation treatment and nursing of depression in adolescents in the southern ethnic regions of Guizhou, cognitive therapy can be effectively applied to improve the degree of depression and enhance the ability of emotional self-control. It is worth promoting in the clinical rehabilitation treatment and nursing of depression in adolescents.

### Acknowledgments

This research was supported by:

1. 2020 Special Scientific Research Project of Nursing Discipline of Guizhou Medical University (Project No. YJ20070);

2. 2023 Science and Technology Fund Project of Guizhou Provincial Health Commission (Project No. gzwkj2023-146).

## LHMM23230 THE INFLUENCE MECHANISM OF SOCIAL MEDIA ON THE COMMUNICATION OF AGRICULTURAL BRANDS AND THE PSYCHOLOGICAL NEEDS OF PRACTITIONERS: AN EXPLORATION BASED ON GROUNDED THEORY

Zhichao Song^a^, Wenlu Wei^b^

*^a^ School of Economics and Management, North China University of Technology, Beijing 100000, China, ^b^ School of Communication, Weifang University, Weifang 261000, China.*

**Background:** With the development and maturity of social media power, the influence of social media on the communication effect of agricultural brands is becoming increasingly apparent. Relevant practitioners feel more pressure on how to meet the psychological needs in the competition of the new industry. Some pathological competition also affects the psychological health of practitioners, such as disorderly competition and industry anxiety. The industry and society need to find answers to the problems based on development to meet individual psychological needs, solve industry pathologies, and sustainable development of agricultural brands. Research on the mechanism of agricultural products’ brand communication in the social media environment will be one of the solutions. It is a generalization and summary of communication methods and effects, and a universal and replicable communication mechanism is refined based on communication practice, which can also meet psychological needs, solve anxiety problems and protect psychological health.

**Subjects and Methods:** This article uses grounded theory and coding analysis through theoretical studies, policies, news, activity reports, and in-depth interviews on the communication of agricultural brands on various social media platforms. Through the coding analysis, the research finds the causes of industry anxiety, the conditions to meet psychological needs, and the solution ideas of pathological competition, and the article constructs a model of the influence mechanism of social media on the communication of agricultural brands.

**Results:** Based on grounded theory, this paper sorted out 58 open codes, 26 spindle codes, and six selective codes in interviews and data screening. This paper describes and analyzes the selective codes, and the extensive data ensures the rigor and credibility of the result sources. Finally, it proposes a mechanism for agricultural brand communication that satisfies psychological needs and alleviates industry anxiety.

**Conclusions:** Research and analysis based on grounded theory show that the influence mechanism of social media on agricultural brand communication will effectively meet psychological needs, relieve industry anxiety and improve mental health. This requires government authorities, industry associations or enterprises, and brand users to think and discuss various aspects when formulating communication strategies and developing more three-dimensional communication strategies to realize the benefits of transformation of agricultural brand communication and ultimately help the industry and practitioners to grow healthily and sustainably.

## LHMM23231 A principle submatrix ALGORITHM FOR MEDICAL ASSIGNMENT PROBLEM

Zhitao Xiao^a^

*^a^ Department of General Education, Guangzhou Huali College, Guangzhou 511325, China.*

**Background:** With the progress of society and the development of medical technology, people need to integrate a large amount of medical resources, allocate medical tasks reasonably, and achieve higher efficiency and less wastage in completing tasks. This article presents a principle submatrix algorithm for the assignment problem where the number of medical tasks exceeds the number of people.

**Subjects and Methods:** Firstly, we present the theory of same solution transformation for n persons and kn tasks assignment problems, followed by a principle submatrix algorithm for this type of problem. Then, we transform the medical assignment problem with more tasks than the number of people into a n persons and kn tasks assignment problem with the same solution, and finally use the principle submatrix method to find the optimal assignment of the problem. The characteristic of this principle submatrix algorithm is that it does not need to consider the overall assignment matrix of the assignment problem, only needs to perform operations locally on the assignment matrix, starting from the first order principle submatrix, and finding the optimal assignment of the problem step by step in a regular manner.

**Results:** For medical assignment problems with more tasks than the number of people, we do not need to transform it into a balanced assignment problem where the number of tasks equals the number of people, this assignment problem is first transformed into an n persons and kn tasks assignment problem with the same solution, and then the principle submatrix algorithm is used to obtain the optimal allocation of the original medical assignment problem. The principle submatrix algorithm for this medical assignment problems with more tasks than the number of people proposed in this article is more regular and operable compared to the commonly used Hungarian method for solving assignment problems.

**Conclusions:** The principle submatrix method proposed in this article is a polynomial algorithm with a computational complexity of. The algorithm starts from a row minimum group of the principle submatrix of the first order of the assignment problem, finds the row minimum groups of the principle submatrix of each order step by step, and finally obtains an optimal assignment of the original medical assignment problem. Through this method, it is convenient to find the optimal assignment of medical tasks that exceed the number of people, and efficiently complete the reasonable assignment of medical resources.

## LHMM23232 THE IMPACT OF GUANGXI OCEAN ECONOMY DIGITAL TRANSFORMATION ON PEOPLE’S MENTAL HEALTH

Hua Ouyang^a^

*^a^ Guangxi University of Finance and Economics, Nanning, Guangxi 530003, China.*

**Background:** The 21st century is the ocean century, the ocean is rich in resources, social and economic development is an important support. Guangxi is located in the Southwest China, with an area of about 40,000 square kilometers in the Gulf of Tonkin Sea, and has huge potential for marine economic development. But at present, the development of Guangxi’s marine economy has encountered a bottleneck. Especially in the digital transformation of the marine economy, the development of digital economy and intelligentization has also brought about the problems of machine dependence, loss of skills, infatuation with the network, separation from reality and spiritual loss, so that people’s mental health has been affected.

**Subjects and Methods:** By analyzing the background and current situation of Guangxi Marine Economy Digital Transformation, this paper probes into the painful and difficult problems in the process of Guangxi Marine Economy Digital Transformation. In particular, the paper deeply analyzes the mental health problems brought by the digital transformation of marine economy, and puts forward feasible solutions.

**Results:** Through the research, the paper finds that although the progress of Guangxi’s marine economy digitization is steady and good, it still faces many problems and challenges, mainly as follows: the marine economy digitization process lags behind, the marine digital economy enterprises lack vitality, the marine digital talents are seriously lacking. The digital development of marine medicine is absent, and the digital transformation affects people’s mental health.

**Conclusions:** We should break the shackles of the traditional economic model on the development of marine industry, and promote the digital development of Guangxi Marine Economy, and finally realize the transformation from “Digital ocean” to “Smart Ocean”. First, to build a strong digital marine area and a modern digital marine economy; second, to accelerate digital innovation and promote a high degree of integration between the digital economy and the marine industry; and third, to formulate preferential policies, fourth, to construct the system of marine medicine to alleviate the mental illness brought by digitalization. We should actively carry out psychological intervention, find out problems as early as possible and try to adapt to the development process of digital economy, so as to fully enjoy the changing modern civilization.

### Acknowledgements

This work was supported by The 2018 National Social Science Foundation Project (Grant No.18BJL049), Guangxi First-class Discipline Applied Economics Construction Project Fund (Grant No.2022YBD06) and the 2022 Annual scientific research project of land-Sea Economic Integration Collaborative Innovation Center (Grant No.2022YB10).

## LHMM23233 EXAMINING THE MEDICAL AND HEALTH IMPLICATIONS OF LONG JUMPERS’ MUSCLE PERFORMANCE DURING TAKE-OFF: A BIOMECHANICAL ANALYSIS USING MULTI-SOURCE INFORMATION INTEGRATION

Yue Ren^a^, Bingquan Luo^a^, Huayu Zhao^b^, Jun Chu^a^, Zihao Li^a^, Jiwen Zhang^a^, Wenrui Zhang^a^

*^a^ Capital University of Physical Education and Sports, Beijing 100191, China, ^b^ Northeastern University at Qinhuangdao, Qinhuangdao 066004,China.*

**Background:** Multi-information sensing technology has gained prominence in various industries, including sports. Long jump, an ancient sport, requires a deep understanding of muscle contraction methods and characteristics during each stage. Previous studies have primarily focused on movement patterns and joint muscle activity, without exploring the anatomical function of muscles and joints or the role of individual joint muscle groups in exercise techniques. By incorporating multi-information fusion technology in long jump research, this study aims to investigate the muscle-specific capabilities of long jumpers and contribute to the medical and health aspects of sports performance.

**Subjects and Methods:** Utilizing multi-source information fusion technology, this study examined the biomechanical attributes of muscle-specific capabilities during the take-off phase of long jumpers, aiming to effectively identify changes in muscle vitality of the take-off leg. The research employed the widely-accepted multi-source information fusion technology to explore the specific conditions of long-jumpers’ leg muscles during the take-off action, which accurately reflects the athletes’ physical condition and has potential implications for injury prevention, athlete health, and performance enhancement.

**Results:** The findings demonstrate that the hip joint of the take-off leg generates greater hip extensor torque during the long jump take-off action, highlighting the importance of active contraction ability of the knee flexor group for successful take-off and avoidance of damage to the posterior femoral muscle group. The range of pressure center change lies between 51.26% and 74.35%, suggesting valuable applications in actual training for improving athletes’ well-being, overall health, and performance in the long jump. Additionally, these results can contribute to the development of personalized training and rehabilitation programs that minimize the risk of overuse injuries and muscle imbalances, ultimately promoting better overall health and longevity in athletes’ careers.

**Conclusions:** This study provides valuable insights into the muscle-specific abilities of long jumpers during the take-off phase using multi-source information fusion technology, which has significant implications for medical and health-related aspects of sports performance. The results emphasize the importance of hip extensor torque and knee flexor group contraction in injury prevention and optimal performance, with potential applications for enhancing training regimens, promoting athlete health, and prolonging careers. Furthermore, the knowledge of these biomechanical characteristics can assist in the early detection and intervention in cases of suboptimal performance, thereby allowing timely adjustments to training or rehabilitation programs and ensuring the athletes’ well-being.

## LHMM23234 RESEARCH ON RESIDENTS’ PSYCHOLOGICAL ANXIETY LEVEL AND LANDSCAPE PATTERN CHANGE OF MINQIN COUNTY SETTLEMENT SYSTEM BEFORE AND AFTER KEY MANAGEMENT OF SHIYANG RIVER BASIN

Youxi Yan^a^, Yubiao Li^b^, Wenqi Gong^b^

*^a^ Department of International Applied Technology, Yibin University, Yibin, Sichuan 644000, China, ^b^ Department of Resources and Environment, Wuhan University of Technology, Wuhan, Hubei 430070, China.*

**Background:** Minqin settlement system located in the downstream of Shiyang River Basin is surrounded by Tengger Desert in the northeast and surrounded by Badain Jaran Desert in the northwest, with strong ecological sensitivity. At the end of the last century, with the increase of settlement population, excessive reclamation and wood-cutting activities resulted in lack of ecological barrier of settlement system and deterioration of human settlement environment, and the residents were in deep anxiety. In order to solve people’s psychological anxiety and repair the residential environment of settlement system, the state and local governments have successively launched the key governance projects of Shiyang River Basin after 2000, and completed the treatment in 2015 and carried out the acceptance of the treatment results smoothly.

**Subjects and Methods:** In order to objectively analyze the restoration effect of key governance on the ecological environment of the settlement system and regulate the anxiety of residents in Shiyang River Basin, the TM remote sensing images of Minqin County in 1980, 2000 and 2015 are taken as the main data sources, and the landscape pattern of Minqin County Settlement System is interpreted through image fusion, geometric correction, image enhancement, data registration and sampling verification by referring to the geospatial data cloud of Chinese Academy of Sciences.

**Results:** In 1980, 2000 and 2015, the area of settlement patches continued to increase, the patch area of cultivated land first increased and then decreased, the patch area of grassland decreased first and then increased, the patch area of forest land was flattened first and then increased, the patch area of water area first decreased and then increased, and the area of desert patches decreased in turn, and the key governance effect did not appear, and the adjustment effect of residents’ anxiety was unsatisfactory.

**Conclusions:** The reason is that the key governance measures of Shiyang River Basin are improper, and the water conveyance mode in the settlement is still artificial lined channels, and the natural water transportation mode of Daxi River and Dadong River is abandoned. The east and west ecological barriers of settlement system cannot be restored naturally due to water shortage, and the residents are still anxious about ecological problems.

### Acknowledgments

This work was supported by Yibin University “Sailing” plan project (2020QH02), General Project of Ministry of Education Foundation on Humanities and Social Sciences in 2021 (21XJA630008) and the fourth round key discipline of Yibin University “Resources and Environment”.

## LHMM23235 EFFECT EVALUATION OF INFECTIOUS DISEASE INTERVENTION BASED ON THE SIR MODEL

Jinyang Liu^a,b^, Boping Tian^a^, Guangqiang Teng^a^

*^a^ School of Mathematics, Harbin Institute of Technology, Harbin 150006, China, ^b^ School of Statistics, Chengdu University of Information Technology, Chengdu 610103, China.*

**Objective:** Infectious diseases are often fierce, dangerous, widely spread, and have a huge social impact. Countries have developed different prevention and control strategies according to the epidemiological status, disposal capacity, and social characteristics of their infectious diseases. Few quantitative studies have been conducted to compare and analyze the intervention effects of infectious disease epidemics in multiple countries. This study aims to use scientific models and methods to evaluate the effect of infectious disease intervention in different countries, sum up experience and lessons, and provide some suggestions for minimizing or effectively controlling infectious diseases that may occur in the future.

**Methods:** This study used the classical epidemiological SIR model and calculates its basic regeneration number to compare the effectiveness of infectious disease epidemic control in four countries: China, America, Italy, and India. By solving the SIR model with a system of differential equations, the infectious disease epidemic curves can be fitted relatively accurately based on available data. It is only necessary to estimate the model parameters to predict the spread of infectious diseases, and then evaluate the intervention effect of infectious diseases.

**Results:** In this study, the epidemic trends of infectious disease in four countries, China, America, Italy, and India, under natural conditions and government control, were obtained by simulation and the basic regeneration numbers were calculated. The study shows that the spread of the epidemic was effectively interrupted earlier in China under control, while the epidemic control was not more effective in America, Italy, and India.

**Conclusions:** As transportation levels increase and the number of mobile populations grows, prevention of infectious diseases is no longer simply a matter of prevention in a particular region or country, but has expanded to a global level. In this study, the SIR model was used to compare and analyze the prevention and control effects of infectious disease outbreaks in four countries. The findings of the study deepen the understanding of infectious diseases and their intervention measures to a certain extent, which plays a role in the common and effective response to similar outbreaks of major infectious disease epidemics in the future. China’s infectious disease intervention measures and related experiences are worthy of active reference by countries around the world.

### Acknowledgments

This work was supported by a project grant from the Natural Science Foundation of China(NSFC) (No.91646106).

## LHMM23236 THE SIGNIFICANCE OF ART THERAPY IN ADDRESSING PSYCHOLOGICAL ISSUES

Tian Li^a^, Ji Guo^a^

*^a^ College of Arts, Gansu University of Political Science and Law, Lanzhou 730070, China.*

**Background:** Art therapy, also known as art psychotherapy, is an important intervention method for treating psychological issues. It spans multiple disciplines, including art, psychology, and medicine, and has gained increasing attention as society continues to develop. Painting therapy is one of the methods of expressive art therapy, which allows painters to use nonverbal tools through the creative process of painting to guide chaotic psychological states and feelings into a clear and orderly state. It can present the suppressed emotions and conflicts within the subconscious and obtain relief and satisfaction during the painting process, thus achieving diagnostic and therapeutic effects.

**Subjects and Methods:** A certain number of college students with psychological issues were selected, and interviews, questionnaires, and analysis were conducted to summarize their psychological problems. Different treatment strategies were employed based on the specific psychological issues of the college students. There are various forms of painting therapy strategies, including free painting, theme painting (ordinary theme painting: trees, self portraits, and house tree people; special theme painting: fairy tales, animals, the opposite sex, troubles, and dreams), gestalt painting (providing certain stimulation points, lines, points, etc. for college students to supplement), and so on.

**Results:** Painting therapy is a service-oriented profession that presents individual development, abilities, personality, interests, concerns, and conflicts through the use of painting media or materials, artistic imagery, creative artistic activities, and patient feedback on the work. Art therapy is generally suitable for individual psychotherapy. Through art therapy, various tense emotions and pressures can be released during the creative process, inherent experiences can be clarified, thoughts can be concretized, and certain psychological needs can be conveyed. Art creation opens a pathway to the subconscious, facilitating the expression, reflection, integration of individual development, abilities, interests, personality, thoughts, and inner emotional states, making it more accessible to the public.

**Conclusion:** Through painting therapy, the suppressed emotions and conflicts in the patient’s subconscious are presented. At the same time, during the process of painting therapy, the patient releases negative energy in their mind, emotions, and thoughts, decompresses, releases emotions, adjusts emotions and attitudes, repairs spiritual wounds, fills gaps in their inner world, and obtains a sense of satisfaction, achievement, and confidence, thereby achieving good diagnostic and therapeutic effects. Art therapy has vast prospects, but due to various objective factors and the low attention given to this therapy in China, there is limited research, insufficient studies, and a lack of prominent case studies, making it difficult to conduct specific and effective art therapy. This article mainly explains the implementation, directions, and unique significance of art therapy, aiming to provide a detailed introduction to the entire process of art therapy, its advantages, and development prospects.

### Acknowledgments

This study was supported by the 2021 Young Doctor Fund Project of Gansu Province (Project No.: 2021QB101).

## LHMM23237 EFFECTS OF SOMATOSENSORY GAMES ON THE MOTOR ABILITY AND ON THE PSYCHOLOGICAL STATUS OF CHILDREN WITH AUTISM

Chenxi Lu^a^, Borhannudin Bin Abdullah^b^, Hazizi Bin Abu Saad^c^

*^a^ Faculty of Educational Studies, Universiti Putra Malaysia, Serdang 43400, Selangor, Malaysia, ^b^ Faculty of Educational Studies, Universiti Putra Malaysia, Serdang 43400, Selangor, Malaysia, ^c^ Faculty of Medicine and Health Sciences, Universiti Putra Malaysia, Serdang 43400, Selangor, Malaysia.*

**Background:** Intelligent somatosensory interactive exercises are the primary focus of this study because of their potential to improve motor skills and effect and on the psychological status in autistic children.

**Subjects and Methods:** The present issues of hand movement training for children with autism, as well as the impact of somatosensory games on psychological status as well as on rehabilitation training for autism, are explored via the use of case studies. Somatosensory activities for teaching kids with autism how to use their hands effectively were studied. The impact of various variables on users’ immersion is investigated via data collecting, classification, and analysis. Functions, programme flow, main features, and implementation effects of the somatosensory game are displayed, and an introduction to the development platform and key technologies used in the design and development of the somatosensory game module are provided.

**Results:** Modelling, character management, task flow management, collision detection, an interactive interface, and natural interaction techniques including gesture and voice interaction are all presented as part of the somatosensory interaction system’s development process. This research paper defines autism and describes its symptoms and features in youngsters. It lays the groundwork for future study by discussing the viability of using somatosensory games in the hand movement training of autistic children and by providing an in-depth analysis of the development status and use of somatosensory games. It also clarifies the product’s intended direction and design content for somatosensory interactive training products based on the defined study material.

**Conclusion:** Somatosensory games have been demonstrated to be useful in the improving the psychological status and rehabilitation of autistic youngsters. Children with autism showed improvements in their visual responses, anxiety responses, verbal communication, and interpersonal relationships after participating in a healthcare intervention based on intelligent interactive gesture technology.

## LHMM23238 ANALYSIS OF MENTAL RESILIENCE OF COLLEGE STUDENTS MAJORING IN PHYSICAL EDUCATION AND NON-PHYSICAL EDUCATION FROM THE PERSPECTIVE OF MENTAL HEALTH

Heshuang Ye^a^, Yuanyuan Dai^a^

*^a^ Physical Education Institute, University of the Chizhou, Chizhou, Anhui 247100, China.*

**Background:** With the rapid development of society, college students often face various pressures, such as study, employment, love and so on. These pressures will seriously affect the mental health of college students. Only strong mental toughness can help them overcome difficulties and promote the good development of mental health.The purpose of this study is to explore the characteristics of mental toughness of college students majoring in physical education and non-physical education, and to provide ideas for further enhancing the mental health level of students of different majors.

**Methods:** The psychological toughness of 788 students of different majors in Chizhou University was investigated by using the Adolescent Mental Toughness Scale and questionnaire survey. Among them, 340 students (217 boys and 123 girls) majored in physical education and 448 students (177 boys and 271 girls) majored in non-physical education. The collected scales and questionnaires were classified, and the data were recorded into Excel for statistics and SPSS 25.0 for independent sample T-test.

**Results:** (1) The total score of the respondents’ mental toughness was 81.02 ± 15.16. Among the dimensions of mental toughness, family support was the highest, followed by interpersonal assistance, goal focus and emotional control, and goal focus was the lowest. (2) For physical education major and non-physical education major, there were significant differences in goal focus, emotional control, family support and interpersonal assistance (P<0.01), and there were significant differences in total score of mental toughness and positive cognition (P<0.05); (3) There were significant differences in total score of mental toughness, emotional control, family support and interpersonal assistance between male physical education major and non-physical education major (P<0.01), and there were significant differences in goal concentration (P<0.05). (4) There were significant differences in goal focus, emotion control, positive cognition and interpersonal assistance between females majoring in sports and females not majoring in sports (P<0.01). (5) Male and female physical education major: The total score of mental toughness and each dimension had no statistical significance (P>0.01).

**Conclusion:** (1) Family support had a significant impact on the psychological resilience of the respondents; (2) The overall mental toughness of physical education major is significantly better than that of non-physical education major; (3) In terms of total score of mental toughness, emotional control, family support and interpersonal assistance, male physical education major are better than female physical education major. In terms of goal focus and positive cognition, male physical education major are weaker than female physical education major. Tips: (1) When improving the mental toughness of college students, it is necessary to unite with their families. Through the joint intervention of family and school, the mental toughness of college students can be effectively promoted; (2) Physical exercise can effectively promote the level of mental toughness. Schools should strengthen physical education and create a strong physical exercise atmosphere. (3) In the work of mental health education, we should pay attention to gender differences and pay attention to teaching students according to their aptitude.

### Acknowledgments

This work was supported by 3 project grant from Key Humanities Project of Chizhou University(Grant No.CZ2021RWZ18); Key Research Project of Philosophy and Social Sciences in Higher Education Institutions of Anhui Province(Grant No.2022AH051811); Key Teaching Research Project of Chizhou University (Grant No.2021XJYXM03).

## LHMM23239 TO EXPLORE THE MECHANISM OF POLYGONATUM IN THE TREATMENT OF RHEUMATOID ARTHRITIS BASED ON NETWORK PHARMACOLOGY AND MOLECULAR DOCKING

Ying Chen^a,b,c,d^, Hao Tian^a,b,c,d^, Qin Ma^a,b,c,d^, Lunzhi Yang^a,b,c,d^, Xue Zhou^a,b,c,d^, Ting Xiao^a,b,c,d^, Ling Tao^a,b,c,d^, Linjing Wu^a,b,c,d^

*^a^ School of Pharmacy, Key Laboratory of Optimal, Utilizaiton of Natural Medicine Resources, Guizhou Medical University, Guiyang 550025, Guizhou, China, ^b^ State Key Lab of Functions and Applications of Medicinal Plants, Guiyang 550025, Guizhou, China, ^c^ Key Laboratory of Natural Medicine Pharmacology and Drug Evaluation of General Colleges and Universities in Guizhou Province, Guiyang 550025, Guizhou, China, ^d^ Guizhou Provincial Efficient Utilization Engineering Center of Featured Natural Medicine Resource, Guiyang 550025, Guizhou, China.*

YC and HT contributed equally to this work.

**Background:** Rheumatoid arthritis is a type of inflammatory arthritis characterized by joint inflammation, swelling, pain and potential damage. Western medicine primarily focuses on controlling inflammation and regulating immune function in the treatment of RA, with commonly used clinical drugs including non-steroidal anti-inflammatory drugs, glucocorticoids, antirheumatic drugs and biological agents. However, prolonged use of these medications may lead to liver and kidney dysfunction as well as gastrointestinal complications. *Polygonatum* exhibits characteristics of “multicomponent, multitarget, and multipathway” which provide potential advantages in the treatment of rheumatoid arthritis (RA). Network pharmacology integrates network biology with various pharmacological approaches based on existing databases. By investigating multiple targets, a “component-target-disease” network can be constructed, providing a novel approach to explore the mechanism and synergistic effects of *Polygonatum* in treating rheumatoid arthritis.

**Subjects and Methods:** This study aims to investigate the mechanism of rheumatoid arthritis treatment using network pharmacology and molecular docking techniques. Through literature mining and database retrieval, the compound components of *Polygonatum* were obtained, the potential targets of the compounds were predicted using the TCMSP database, and the related targets of rheumatoid arthritis were obtained through GeneCards, OMIM and other databases, and the intersection targets of *Polygonatum* for the treatment of rheumatoid arthritis were obtained. Based on the STRING database, Cytoscape software was used to construct the interaction network of the common target protein of *Polygonatum* and Rheumatoid Arthritis, and the key targets were selected for molecular docking with the chemical components of *Polygonatum* to verify and analyze the interaction between them.

**Results:** The final screening results revealed the identification of 12 active components, 65 potential targets, and 4797 potential targets for rheumatoid arthritis. Additionally, a total of 48 common targets were identified. GO and KEGG enrichment analysis led to the discovery of 343 signal pathways related to the main components found in Polygonum japonicum. The core components β-sitosterol and baicalein exhibited strong binding affinity with key targets VEGFA and RELA.

**Conclusions:** Through the application of TCM network pharmacology, this study revealed that *Polygonatum* may play a multi-component, multi-target and multi-pathway therapeutic effect on rheumatoid arthritis by regulating cell apoptosis and cholesterol-like hormone receptors, participating in cancer pathways and other mechanisms, which may provide a reference for the screening of clinical efficacy evaluation indicators for the treatment of rheumatoid arthritis.

### Acknowledgement

The research was supported by Guizhou Provincial Basic Research Program (Natural Science, Nos. ZK [2022]389 and ZK [2023]303) and Guizhou Provincial Key Technology R&D Program (No. [2020]4Y104).

## LHMM23240 REPORTING INFECTIOUS DISEASES TRUTHFULLY TO REDUCE PUBLIC PSYCHOLOGICAL PANIC: THEORY FROM PUBLIC HEALTH EVENTS

Chong He^a^

*^a^ Department of Economics, Zhejiang University, Hangzhou 310030, China.*

**Background:** Study the incentives of the country for reporting emerging infectious disease outbreak, in order to reduce public panic and herd behavior during public health events.

**Subjects and Methods:** This paper expands the existing single signal game on the motivation of national reporting epidemic to the infinite signal game. In the model, the main benefit of reporting infectious disease outbreak is to reduce casualties, while the main loss is the decrease in income caused by trade and travel disruptions. The model incorporates the loss of mental health of the population due to panic in an epidemic and the losses caused by disrupting social order due to herd behavior such as rushing to buy household goods. In addition, the model takes into account the accuracy of medical detection of infectious disease outbreak as a way to distinguish the behavior of the country under different parameters.

**Results:** The country is more willing to truthfully report the diagnostic test of the disease outbreak in the infinite game than in a one-shot game in order to win the trust of the public and reduce social psychological panic. When trade and travel disruption intensifies, the country will tend to adopt an ‘oscillating’ strategy: conceals or adopts a mixed action when the volume of trade and travel is above the threshold, but truthfully reports the diagnostic test when the volume is below the threshold. The rapid growth of trade and travel may inhibit the willingness of the country to truthfully report the detection information and increases the risk of disease transmission.

**Conclusions:** When making decisions, the government should not only consider economic losses, but also consider the psychological health issues among the public caused by the epidemic and lack of information, which helps the government truthfully report infectious disease outbreak. It is not better for the partner to impose trade and travel disruption as harshly as possible. The increase in assistance from international organizations such as WHO can help the country to report truthfully, but blindly improving the detection accuracy may only lead to the waste of funds. What’s more, a financial mechanism led by the World Bank should be set up to compensate national and local governments for economic losses associated with outbreaks, especially in areas with the greatest lack of health resources.

## LHMM23241 EXPERIMENTAL STUDY ON THE INTERVENTION OF BA DUAN JIN TO THE SUB-HEALTH OF UNIVERSITY STUDENTS

Kaiwen Li^a^, Dongyi Huang^b^, Wu Liu^c^

*^a^ Wenshan University, Yunnan Wenshan 633099, China, ^b^ School of Sports Science, Guangzhou College of Applied Science and Technology, Guangzhou 511370, China, ^c^ Yunnan Vocational College of Mechanical and Electrical Technology, Yunnan Kunming 650203, China.*

**Background:** With the rapid development of China’s economy, more and more college students are in the state of sub-health, among the types of exercise used in the clinical management of sub-health, some are related to the ancient Chinese traditions as Baduanjin. Despite the growing interest of the scientific community in Baduanjin, which has the theoretical basis of traditional Chinese medicine, its research is still in its infancy. It is noteworthy that, to the best of our knowledge, there are no studies that verified the influence of Baduanjin on sub-healthy college students. Therefore, the main objective of the present study was to investigate the effects of the Baduanjin, a traditional Chinese Qigong, on the physical and mental health of sub-healthy college students.

**Subjects and Methods:** Explore the intervention effect of Baduanjin exercise on sub-health college students. The average age (males, n=112, 20.02 ± 0.85; females, n=108, 20.31 ± 0.9). Methods Comparative analysis method was used. Practicing baduanjin for 18 weeks, after the experiment, the improvement of physical, mental and fatigue sub-health was assessed by “Personal Health Status Questionnaire”. HK6000, G100, JGW-B and FCS-10000 were used to evaluate the physical forward bend, grip strength, Reaction time, and vital capacity.

**Results:** The 220 students were randomly allocated to the control or experimental group. Physical and mental health, fatigue status, and physical fitness were assessed in both groups before and after the experimental protocol. Compared with the control group, the physical subhealth and mental subhealth of the experimental group were significantly improved (P<0.05). The fatigue state of the experimental group was significantly relieved (P<0.05). Baduan jin intervention significantly improved sitting forward flexion ability and lung volume (P<0.05).

**Conclusion:** It is proved that baduanjin has significant therapeutic effect on sub-health of college students. It provides a new way for researchers and coaches to conduct research and intervention from the perspective of traditional medicine.

### Acknowledgments

Thanks to Professor Gu Yongkun, Professor Liu Wu and coach Lu Tianxue for their support to the sub-health intervention students.

## LHMM23242 RESEARCH ON THE DEVELOPMENT OF YANGQIN PERFORMANCE IN CHINA BASED ON THE HEALING OF MUSIC TO THE BODY AND MIND

Chin-niang Chan^a^

*^a^ School of Art, Minnan Normal University, Zhangzhou, Fuijian, China.*

**Background:** Music therapy is different from conventional instrumental music, as it has the ability to evoke every possible emotion. A melodious voice can heal a person, and music therapy is a constantly evolving concept that can promote healing. Mainly, it is a therapeutic method that utilizes the natural emotional enhancement characteristics of music. In addition, it can also help people improve their mental health and overall happiness.

**Subjects and Methods:** Music therapy includes developing music, writing songs, singing, dancing, listening to and discussing music. Especially, this form of treatment may be helpful for people with depression and anxiety. In addition, it may help improve the quality of life for people with physical health issues. The music of playing the yangqin can make us soar to unimaginable heights, comfort us in sadness or loneliness, help us release our anger or frustration in a way that does not harm anyone, make our bodies move, and bring peace and tranquility to our hearts.

**Results:** Music therapy is an expressive art therapy that uses music to improve and maintain an individual’s physical, psychological, and social well-being. In addition, it also involves a wide range of activities, such as listening to music, singing, and playing musical instruments. Music can also improve our health and happiness. Music heals, allowing wonderful music to improve our physical and mental well-being. After experiencing the baptism of hundreds of years of Chinese culture, the yangqin has achieved outstanding development in its performance form. In addition to solo performances, the yangqin is widely involved in various forms of performance such as traditional Chinese opera, Quyi accompaniment, ensemble, large, medium, and small ensemble, and has become an important component of Chinese ethnic instruments. A large number of literature references and field research are combined to obtain the original data, and the results can be obtained only after analysis and research.

**Conclusion:** The increasing evidence of music therapy suggests that music therapy is not just a great way to boost energy, but can improve medical outcomes and quality of life in various ways. Surprised by the magic of music and its benefits in treating mental illnesses. Not only that, music therapy can also cure some symptoms of physical problems. Of course, there are some types of music to enhance emotions and relieve stress. The yangqin has been continuously absorbed, integrated, transformed, and developed in the process of historical transformation, and its development requires the improvement of the content of the work. In the creation and performance of traditional and modern works, it is necessary to strive for innovation through change and progress through development, making contributions to enriching and improving the art of yangqin.

## LHMM23243 RESEARCH ON THE GROWTH OF TRADITIONAL CHINESE MEDICINE INDUSTRY FROM THE PERSPECTIVE OF NATIONAL HEALTH: BASED ON FACTOR ANALYSIS METHOD

Lingshan Li^a,b^, Bo Su^a,b^

*^a^ School of Management, Hubei University of Chinese Traditional Medicine, Wuhan, Hubei 430070, China, ^b^ Research Center for the Development of Traditional Chinese Medicine, Key Research Institute of Humanities and Social Sciences of Hubei Province, Wuhan, Hubei 430070, China.*

**Background:** With the great contribution of the traditional Chinese medicine (TCM), China has made significant breakthroughs in combating the epidemic. Moreover, China has released many relevant policies to support the development of the traditional Chinese medicine industry. For the public, more and more people are paying attention to personal health management. The development of the traditional Chinese medicine industry will further promote the health of the entire population.

**Subjects and Methods:** In order to further understand the degree of the development of Chinese medicine industry in China, this paper selects 62 listed companies in the traditional Chinese medicine (TCM) industry to research their growth in the future with their financial data in 2022. With reference to listed company performance evaluation index system, we constructed a index system of 12 indicators from 4 aspects using factor analysis for empirical analysis. These four indicators are profitability, debt paying ability, operational ability, and growth ability. These four abilities can comprehensively measure the overall situation of a company.

**Results:** According to the empirical results, it can be found that among the 62 listed TCM companies, 32 companies got a growth score of more than 1, accounting for 51.61%, indicating that these companies had a good development prospect. However, only 3 listed TCM companies scored more than 1 in all indicators, which suggests that there is still a lot of development space for other companies to explore. The top ten companies all have well-known products and operate well.

**Conclusions:** The TCM company needs to develop its own characteristics to enhance its growth potential. The TCM companies also need comprehensive development to be more competitive. The development of a company needs to meet the public’s health needs in order to have greater development space. The development of the traditional Chinese medicine industry plays an important role in disease prevention and can better assist people in health management.

## LHMM23244 THE IMPACT OF PSYCHOLOGICAL PROBLEMS ON IRRATIONAL INVESTING: EVIDENCE FROM PHARMACEUTICAL STOCKS

Xuecheng Wang^a^; Qiushuang Duan^a^

*^a^ School of Economics and Management, North China University of Technology, Beijing, China.*

**Background:** Investor’s mental health has a great impact on their investment behavior. Anxiety, autism and personality disorders may cause investors to loss their judgment, rely more on the herd effect, and make irrational investment behaviors. Frequent trading triggered by rumors and hot spots causes investors to ignore the value of stocks and lead to investment failure. The existing research shows that the digital transformation strategy has little impact on the profit of R&D-dependent industries as pharmaceutical and health industries. Will the hype about the concept of the digital economy lead to investors exhibiting irrational behaviour? Will listed companies in the pharmaceutical industry use the digital economy to push up their stock prices? This paper will examine the above issues.

**Subjects and Methods:** This paper have tested whether there is an irrationality investment behavior caused by unhealthy psychology with individual fixed panel regression models of 299 A-share listed companies in China’s pharmaceutical industry from 2010 to 2020.This paper uses abnormal trading volume to represent unassertive investment due to blindness, pressure and other negative psychology, uses the FGLS estimation method to estimate the parameters of the model, and use the two-step correction method for the coefficient correction of AR(1) and the PSCE method for the heteroskedasticity-robust covariance correction to produce more reliable regression results. We also performed robustness tests to avoid regression bias. All methods are based on theoretical deduction and empirical analysis of investment psychology.

**Results:** The hypothesis that unhealthy investment psychology attracted by investment hot-spots encourages blind investment is well tested. There is a herd effect attracted by the digital transformation strategy of pharmaceutical companies. Investors trade frequently and drive up the stock price severely than normal. On average, an increase of 1 unit in the frequency of digital economy related vocabulary (per thousand words) in the annual reports will increase stock trading volume by 1000 million in a whole year. This result is significant at the 1% level. This result proves that overinvestment due to psychological problems exists. The robustness test also shows that the stock price will be raised by about 23.89%.The results can be used to explain the collective psychological problems in investment, such as excessive profit pressure, choice anxiety, and excessive comparison.

**Conclusions:** Under the influence of herd mentality, investors will indeed make irrational investment behaviors. This unhealthy psychology is transmitted between groups, causing distortions in the value of the stock market. It can be seen that investors have high hopes for the digital transformation of pharmaceutical companies. But poor knowledge and overstress drive people to ignore risks. Before the digital economy strategy takes effect, investors should overcome anxiety and maintain a healthy mind. Pharmaceutical companies should increase corporate profits and promote the lasting development of the industry. The mechanism of individual psychological problems on market failure is the subject of further research.

### Acknowledgements

This research was supported by Beijing Social Science Foundation (Grant No.22JCC077) and Beijing Urban Governance Research Base of North China University of Technology.

## LHMM23245 TO EXPLORE THE PATHOGENESIS AND PROVIDE THE PREVENTION AND TREATMENT OF TIC DISORDER IN CHILDREN BASED ON THE THEORY OF “OCCURRENCE DUE TO ADDING CAUSATIVE FACTORS”

Hongyan Shen^a^, Dongmei Li^a^, Changqing Feng^a^

*^a^ Affiliated Hospital of Liaoning University of Traditional Chinese Medicine, Shenyang 110032, China.*

**Background:** In recent years, with the increasing incidence of tic disorder in children, the author observed children suffering from tic disorder accounted for nearly one fifth of the chronic disease patients in the pediatric outpatient department of our hospital. Ranking the second after spleen and stomach disease, tic disorder has witnessed a decreasing trend in its onset age. Due to the complex etiology of tic disorder, there is no specific treatment method at present.

**Subjects and Methods:** To explore the etiology and pathogenesis and provide favorable methods about prevention and treatment of tic disorder in children according to the theory of “occurrence due to adding causative factors”.

In the process of the clinical research on tic disorder, we consulted, collected and sorted out a lot of literatures, including classical TCM ancient books and medical journals, etc., combined with clinical data, and systematically analyzed the diagnosis and treatment of tic disorder. We collected and referred to huge amounts of information in TCM books and periodicals, in order to further clarify the pathogenesis of tic disorder, and to identity the focus of treatment and prevention for tic disorder. In this paper, the pathogenesis of tic disorders in traditional Chinese medicine is clarified from all aspects. This study is based on the theory of “occurrence due to adding causative factors”, which clarify the pathogenesis of tic disorder from the inside to the outside, from the point to the surface, from multiple perspectives, including the children’s constitution, body endowment and traditional Chinese medicine Yin and Yang, the five elements, and the visceral state.

**Results:** The etiology and pathogenesis of tic disorder in children are similar to the theory of “occurrence due to adding causative factors” in Canon, which is the most classical TCM ancient book. The pathogenesis of tic disorder in children can be explored from the theory of “occurrence due to adding causative factors “, so as to guide the treatment for children with tic disorders.

**Conclusions:** The treatment based on the theory of “occurrence due to adding causative factors” for children with tic disorder can better guide clinical treatment and differentiation of syndromes, so as to achieve “curing the disease to seek the root”, guide the clinical children to “prevent diseases before occurrence”, and thus has certain clinical practical value.

### Acknowledgments

This paper was supported by the basic research project of the education department of Liaoning province (2100221274).

## LHMM23246 APPLICATION RESEARCH OF 4S LYING POSITION REHABILITATION EXERCISE FOR AECOPD PATIENTS IN HOSPITAL-FAMILY INTEGRATED LUNG REHABILITATION NURSING

Wei Duan^a^, Huiling Lu^a^, Xiaopan Di^a^, Yan Li^a^, Bo Chen^a^, Danyang Li^a^, Long Zhang^a^, Yuping Zhang^a^, Hui Yao^a^

*^a^ Department of Medical, People’s Hospital of Ningxia Hui Autonomous Region, Yinchuan, Ningxia 750001, China.*

**Background:** To investigate the efficacy of 4S lying position lung rehabilitation exercise, in hospital-family integrated lung rehabilitation nursing of acute exacerbation of chronic obstructive pulmonary disease(AECOPD) patients,and to summarize the application experience.

**Subjects and Methods:** A total of 99 AECOPD patients who were hospitalized from March 2019 to June 2020 in People’s Hospital of Ningxia Hui Autonomous Region were enrolled in the study. According to the inclusion criteria, Patients were randomly divided into observation group (49 cases) and control group (50 cases). The observation group had 34 males and 15 females, ranging in age from 63 to 84 years, with an average of (72.19 ± 7.89) years; the control group had 33 males and 17 females, ranging in age from 66 to 83 years, with an average of (73.70 ± 5.32) years. There was no statistically significant difference in gender and constitutional indext (P>0.05).Both groups received the same drug treatment. The observation group received 4S lying position lung rehabilitation exercise, while the control group received routine lung rehabilitation program. mMRC scores, 6MWT, CAT scores and compliance of family lung rehabilitation were compared between the two groups before and after intervention for 12weeks.

**Results:** Comparison of the compliance of family lung rehabilitation between the observation group and the control group after 12 weeks of intervention was improved, but the improvement degree in the observation group was significantly higher than that in the control group(P < 0.05), with statistical significance. 12 weeks after intervention, the scores of mMRC, 6MWT and CAT in both groups were improved. The improvement of the observation group was significantly higher than that of the control group (P<0.05), with statistical significance.

**Conclusions:** 4S lying position rehabilitation exercise is safe, effective and simple.It can relieved patient’s dyspnea symptoms,promote exercise ability and quality of life, which has positive significance for patients’ self-care ability, maximum social function and is thus worthy to promote hospital-family integrated lung rehabilitation nursing mode for AECOPD patients.

### Acknowledgments

This work was supported by a project grant from Ningxia Hui Autonomous Region Health System Scientific Research Project (Grant No.2022-NWKY-006).

## LHMM23247 PREPARATION OF CLOBOPIRIDE BIOADHESIVE SUSTAINED RELEASE TABLETS & ITS EFFECTS ON EXPERIMENTAL GASTRIC ULCER & GASTROINTESTINAL DYSFUNCTION

Yang Nie^a^, Junfang Zhu^b^, Huifang Chen^c,d^, Haichao Huang^b^, Bo Li^b^, Yan Li^d^, Jianhua Yi^e^, Bing Liu^f^, Hua Cao^d^, Li Yao^g^

*^a^ School of Pharmaceutical Engineering, Guangdong Food & Drug Vocational College, Guangzhou 510520, China, ^b^ Experimental Training Center, Guangdong Food & Drug Vocational College, Guangzhou 510520, China, ^c^ College of Chemistry & Pharmacy, Guangxi Normal University, Guilin, 541004, China, ^d^ Guangdong Lingnan Institute of Technology, Guangzhou 510663, China, ^e^ Department of Orthopedics, East Hospital of the First Affiliated Hospital of Sun Yat sen University, Guangzhou,510700, China, ^f^ School of Health, Guangzhou Huaxia Vocational College, Guangzhou 510935, China, ^g^ School of Food Science and Biology, Guangdong Polytechnic of Science and Trade, Guangzhou, 510430, China.*

Y Nie and HF Chen contribute equally to this work.

**Background:** This project aims preferred clebopride bioadhesive sustained-release tablets of the prescription and preparation processes, and to study the effects of Clebopride (CBP) bioadhensive sustained-release tablets on experimental gastric ulcer and gastrointestinal motility disorder.

**Subjects and Methods:** Release tablets release as an indicator, the use of orthogonal design to optimize select clebopride prescription bioadhesive sustained release tablets. Gastric ulcer rat model was induced by ethanol and aspirin, and then divided into model group (normal saline), common tablet (CBP tablet 0.072 mg/kg) and sustained-release tablet high-dose and low-dose groups (CBP bioadhensive sustained-release tablet 0.072, 0.036 mg/kg); normal rats were included in normal control group (normal saline); they were given relevant medicine intragastrically, twice a day for sustained-release tablet, three times a day for other. Ulcer area were observed 2 and 4 days after medication to calculate healing rate of ulcer (n=6). Gastrointestinal motility disorder mice model was induced by atropine, and then divided into model group (normal saline), common tablet group (CBP tablet 0.1 mg/kg) and sustained-release tablet high-dose, medium-dose and low-dose groups (CBP bioadhensive sustained-release tablet 0.1, 0.05, 0.025 mg/kg); normal mice were included in normal control group (normal saline); they were given relevant medicine intragastrically, once a day, for consecutive 3 days. The rate of gastric emptying and small intestinal propulsion were detected (n=6).

**Results:** The optimized formulation consisting of hydroxypropyl methyl cellulose (HPMC 5.5g/100 tablets) and carbomer (CP 2.2g/100 tablets) bioadhesive and skeletal material, lactose as diluent (1.2g/100 film), starch as a filler and a disintegrant (2.0g/100 tablets), optimized in line with a sustained-release tablets prescription kinetic process, release meet the design requirements. Simultaneously with clebopride ordinary tablets measured and compared to the rat gastrointestinal tract adhesion. Compared with normal control group, ulcer area of rats increased in model group; compared with model group, that of rats decreased in common tablet group and sustained-release tablet high-dose, low-dose groups, with statistical significance (P< 0.01); healing rates of gastric ulcer were 32.35%-48.24% 2 days after medication, and those were above 70% 4 days after medication. Compared with normal control group, the rate of gastric emptying and small intestinal propulsion in mice decreased in model group; compared with model group, those of mice increased in common tablet group and sustained-release tablet high-dose, medium-dose, low-dose groups. The effects of sustained-release tablet high-dose and medium-dose groups were better than that of common tablet group; those difference had statistical significance (P< 0.01 or P< 0.05).

**Conclusions:** This study obtained clebopride bioadhesive sustained-release tablets have good slow-release effect, reasonable preparation process is simple, and significant adhesion performance to meet clinical needs, CBP bioadhensive sustained-release tablets have improvement effects against gastric ulcer of rats and gastrointestinal motility disorder of mice, CBP bioadhensive sustained-release tablet has good development prospects.

### Acknowledgements

This work was supported by the key project grant of Natural Science Foundation of Guangxi Province in 2022: mitochondrial autophagy inhibits aBelta deposition and tau phosphorylation to improve cognitive function of AD model animals (Grant No.2022GXNSFAA035447).This thesis is also supported by the following project funds: 1) 2019 Guangdong Medical and Health Education Steering Committee Project: Deep Research on the Teaching Reform of Biopharmaceutical Technology Course under the Background of “School enterprise Cooperation” (Project No. 2019LX025); 2)the project of Guangdong Provincial Health and Health Commission on the development, pharmacokinetics and pharmacodynamics of coagulation factor XIII microsphere long-acting injection (No. A2021203) In 2021; 3)Evaluation on the Introduction, Tissue Culture, Rapid Propagation and Planting Effect of Genuine Rhizoma Bletillariae of Guangdong Provincial Bureau of Traditional Chinese Medicine (No. 20191242) in 2020.

